# Reactivities of *N*-Nitrosamines against Common Reagents and Reaction Conditions

**DOI:** 10.1021/acs.oprd.4c00217

**Published:** 2024-09-18

**Authors:** George A. Hodgin, Michael J. Burns, Benjamin J. Deadman, Christopher S. Roberts, King Kuok Mimi Hii, Bao N. Nguyen

**Affiliations:** † School of Chemistry, University of Leeds, Woodhouse Lane, Leeds LS2 9JT, U.K.; ‡ Lhasa Ltd., Granary Wharf House, 2 Canal Wharf, Leeds LS11 5PS, U.K.; § Centre for Rapid Online Analysis of Reactions, Molecular Sciences Research Hub, Imperial College London, 82 Wood Lane, London W12 0BZ, U.K.

**Keywords:** *N*-nitrosamines, reactivity, cheminformatics, impurity

## Abstract

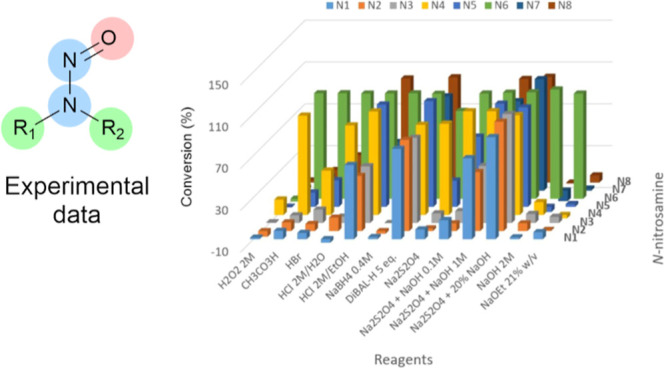

The knowledge of the reactivity of *N*-nitrosamines (NSAs) with common organic reagents in synthesis is essential in determining their presence in pharmaceutical products, if formed and retained during synthesis. In this study, we carried out a comprehensive survey of the Reaxys database for all reactions in which the NSA functional group is consumed. Very different reactivities for different classes of NSAs, e.g., *N*,*N*-dialkylnitrosamines and *N*,*N*-diphenylnitrosamine, were identified, suggesting substrates which should be included in any future reactivity screening. A classification of NSAs based on their reactivities, and corresponding reagents and transformations, was drawn up based on the data. Furthermore, the survey identified missing areas in the reported reactivities of NSAs with different reagents. This led to an experimental reactivity screening of 8 commercial NSAs with common synthetic reagents in the Mirabilis tool for purge assessment. The results showed Na_2_S_2_O_4_ in 1 M aqueous NaOH at 50 °C to be highly effective at destroying NSAs without damaging other organic compounds.

## Introduction

The presence of *N*-nitrosamines (NSAs) in pharmaceutical products has gained center stage from a regulatory perspective with the discovery of *N*-nitrosodimethylamine (NDMA) in valsartan and in other classes of drugs such as ranitidine.^1^ Updated guidance from health authorities required NSA risk assessments for all synthetic drug products on the market and, if required, to carry out confirmatory testing and make changes to the product manufacture or control strategy.^2,3^ The requirement also applies to biological products and medicines and marketing applications for new products. Thus, it is important for the chemistry community to develop complete understanding of how NSAs can be generated during active pharmaceutical ingredient (API) synthesis^4−6^ and how NSAs may be entirely removed during synthetic processes without affecting the final products. These are essential to help the pharmaceutical industry develop robust risk assessment processes and control strategies to ensure the safety of patients.

**Scheme 1:**
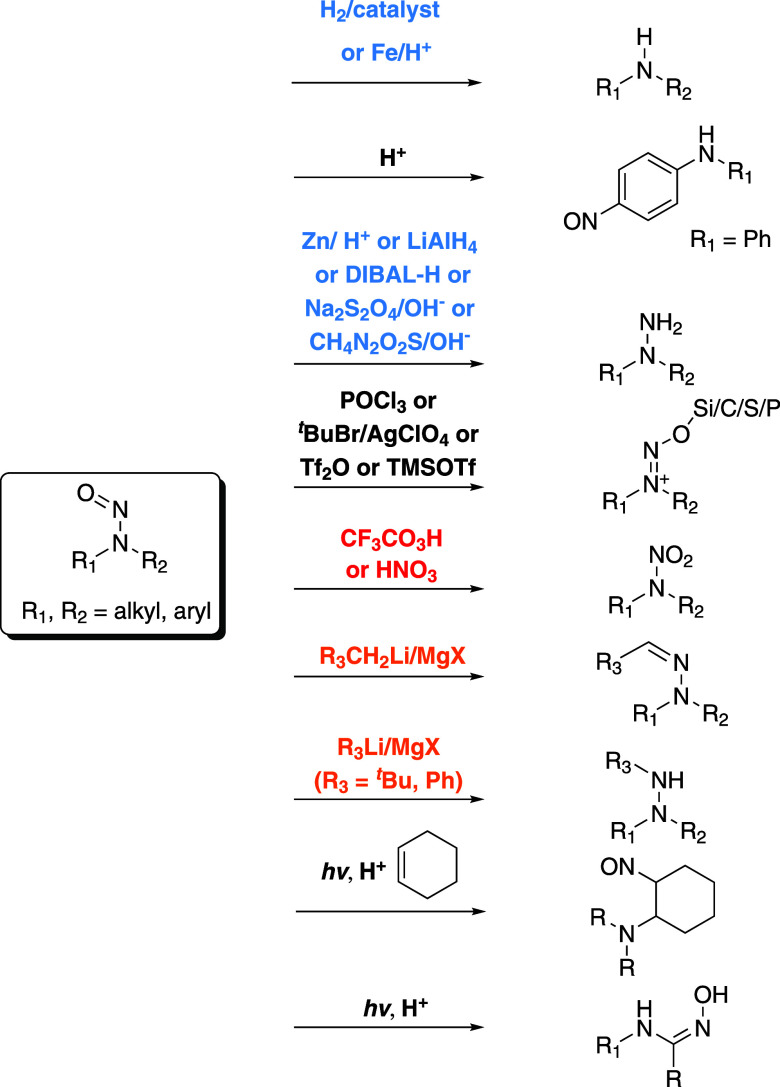
Classes of Reactions of Nitrosamines Summarized in Previous Reviews (**Red** for Oxidative Conditions, **Blue** for Reductive Conditions, and **Orange** for Organometallic Reagents)^8,9^

In this context, the reactivity of NSAs with common chemical reagents in synthetic reactions is part of the foundation of risk assessment and control strategies. Despite their long history,^7^ few quantitative measurements of reactivity, i.e., rate constants, are known and reactions of NSAs are few in the literature. Recent reviews by Swager and Borths showed that NSAs can undergo a small number of reactions under harsh conditions, e.g., strong acids, lithium bases, Grignard reagents, hydrogenation with Raney-Ni or 10% Pd/C, and metal reductants (Scheme 1).^8,9^ Milder reagents and conditions, such as thiourea dioxide (TDO)/NaOH/50 °C or Na_2_S_2_O_4_/NaOH,^10,11^ are fewer in number with a limited reported substrate scope. This scarcity of data is partially due to the hazards associated with NSAs, which necessitates stringent safety protocols and dissuades extensive studies by synthetic chemists.

In this article, we report our complete survey of reported reactions of NSAs using a cheminformatics and data science-based approach. The data highlighted the gaps in our knowledge of the reactions of NSAs and biased data toward dialkyl NSAs due to their role in food and drug contamination. This led to an experimental campaign to collect reactivity data of a set of 8 different NSAs with focus on dialkyl NSAs with common reagents and workup/purification conditions in organic synthesis, where both reactions and nonreactions are important in evaluating purge factors of NSAs in chemical syntheses.^12^ These highly valuable reaction data will inform medicinal and process chemists on the risks and mitigation strategies in drug design and development.

## Results and Discussion

### Reactions of NSAs in the Literature

#### Data Collection and Curation

**Figure 1:**
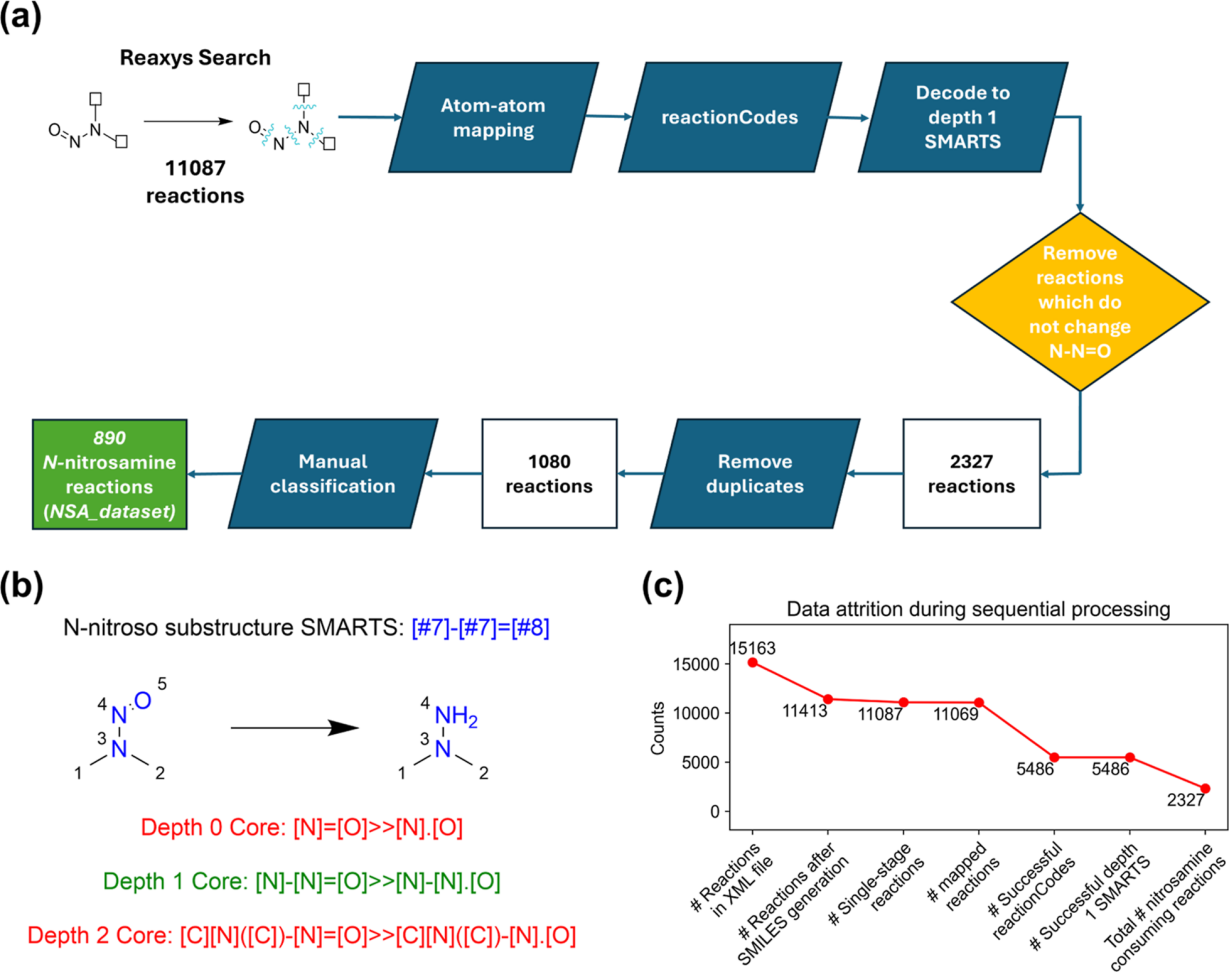
(a) Workflow for the collection and curation of the NSA reaction data set from Reaxys; (b) ReactionCode and SMARTS string at different depth cores for identifying reactions in which the NSA functional group is consumed; and (c) graph to show the data attrition during data collection.

A search of all reactions in Reaxys database containing NMDA as a substructure of the starting material resulted in 15,163 reactions, which are exported in XML format (Figure 1a). For each reaction, SMILES strings for reactants and products, and reaction conditions, which included solvents and catalysts, were extracted with a Python script. The Python package ***rdkit*** was used to convert MDL Molfiles in the exported XML file to SMILES strings. This resulted in 11,413 reactions with extractable information. After this, multistep reactions were removed, leaving 11,087 single-step reactions. In order to perform functional group analysis and reaction classification at the later stages, the Reaction Decoder Tool (RDT)^13^ was employed to perform atom–atom mapping (AAM) based on the computed reaction SMILES strings, giving 11,069 reactions. AAM is essential for reaction center generation,^14−16^ which will underpin automated processing of the collected reactions. The RDT was reported to achieve 76% accuracy in mapping reactions in a curated data set containing a mixture of balanced and unbalanced reactions.^17^ Against the unbalanced reactions from Reaxys, a 99.8% conversion rate was obtained due to the higher data quality of these curated reactions. AAM was followed by application of ReactionCode (version 1.2.2) software to encode the reactions into ReactionCodes. ReactionCodes are a layered string-based language which defines a reaction center as the atoms and bonds which change in a reaction, and the reaction can be portrayed at various depths.^18^ The depth was evaluated (Figure 1b) and a depth 1 core was found to be sufficient to capture the changes to the NSA functional group. A total of 5486 reactions successfully underwent these transformations. Reactions which were not successfully transformed were often not balanced, taking into account the recorded reagents, or included side products in the reaction SMILES strings, which also led to balancing problems. An analysis of these discarded reactions showed similar reagents to those carried forward (Supporting Information, Section S1.3), suggesting that few transformations of NSAs were lost at this stage. The only significant class of the reaction which was removed at this stage are oxidation reactions (e.g., with H_2_O_2_ 90%), which have been well-covered in previous reviews.^8,9^ The correctly assigned reaction centers were analyzed, and reactions in which the *N*-nitroso substructure was not consumed were removed, leaving 2327 reactions (Figure 1c). Duplicated reactions, based on starting materials, products and reagent/conditions, were removed, leaving a data set of 1080 unique reactions of NSAs. Finally, manual classification of these reactions was performed, and 890 reactions were found to have adequate information to determine the reaction class. These reactions form the ***NSA_data set***.

#### Analysis of Literature Reported Reactions of NSAs

**Figure 2:**
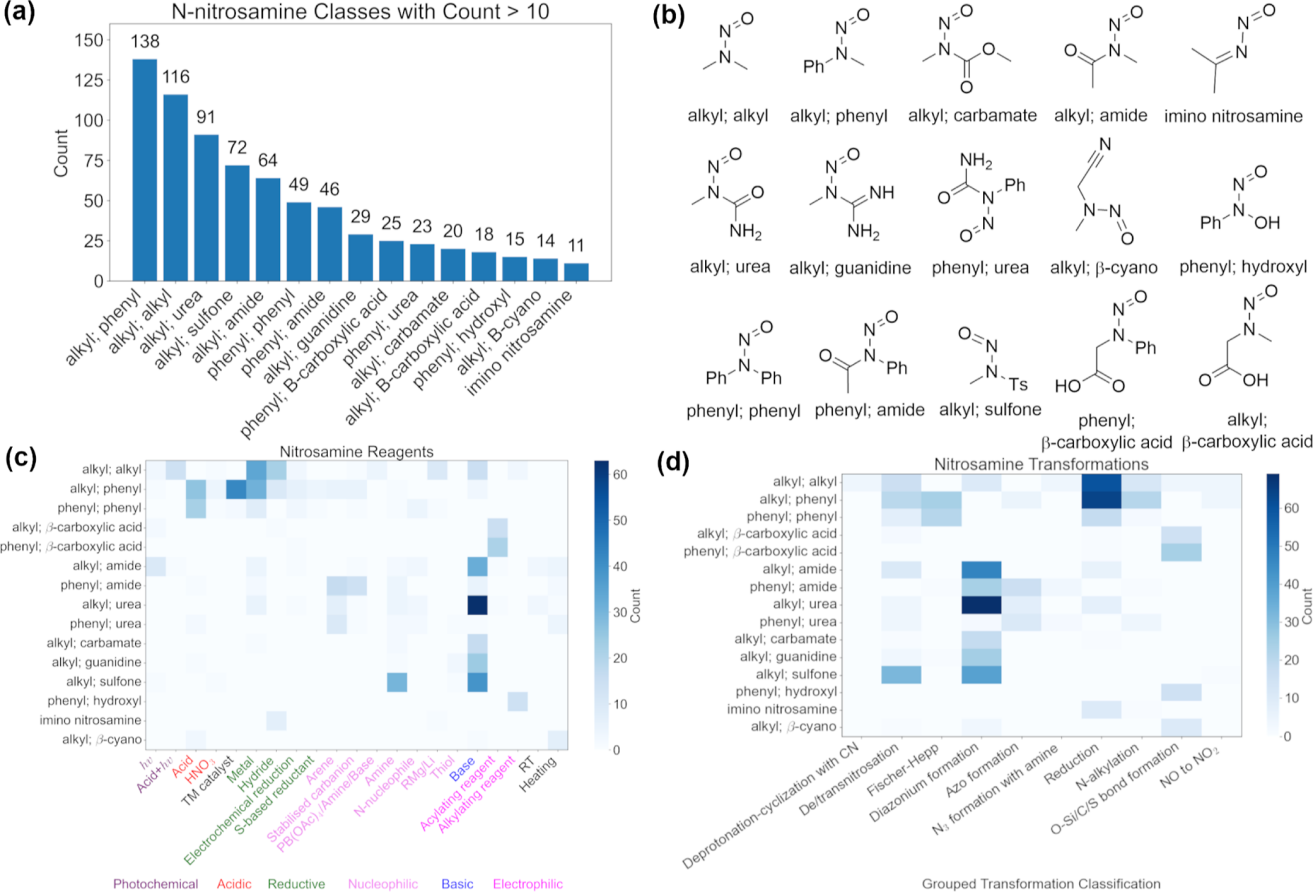
Literature reaction data analysis: (a) distribution of different classes of NSAs as starting materials in ***NSA_data set***, showing classes with 10 or more compounds; (b) representative structures of different classes of NSAs; (c) reaction counts (>3) between different reagents and classes of NSAs; and (d) transformation counts (>3) for different classes of NSAs.

**Scheme 2:**
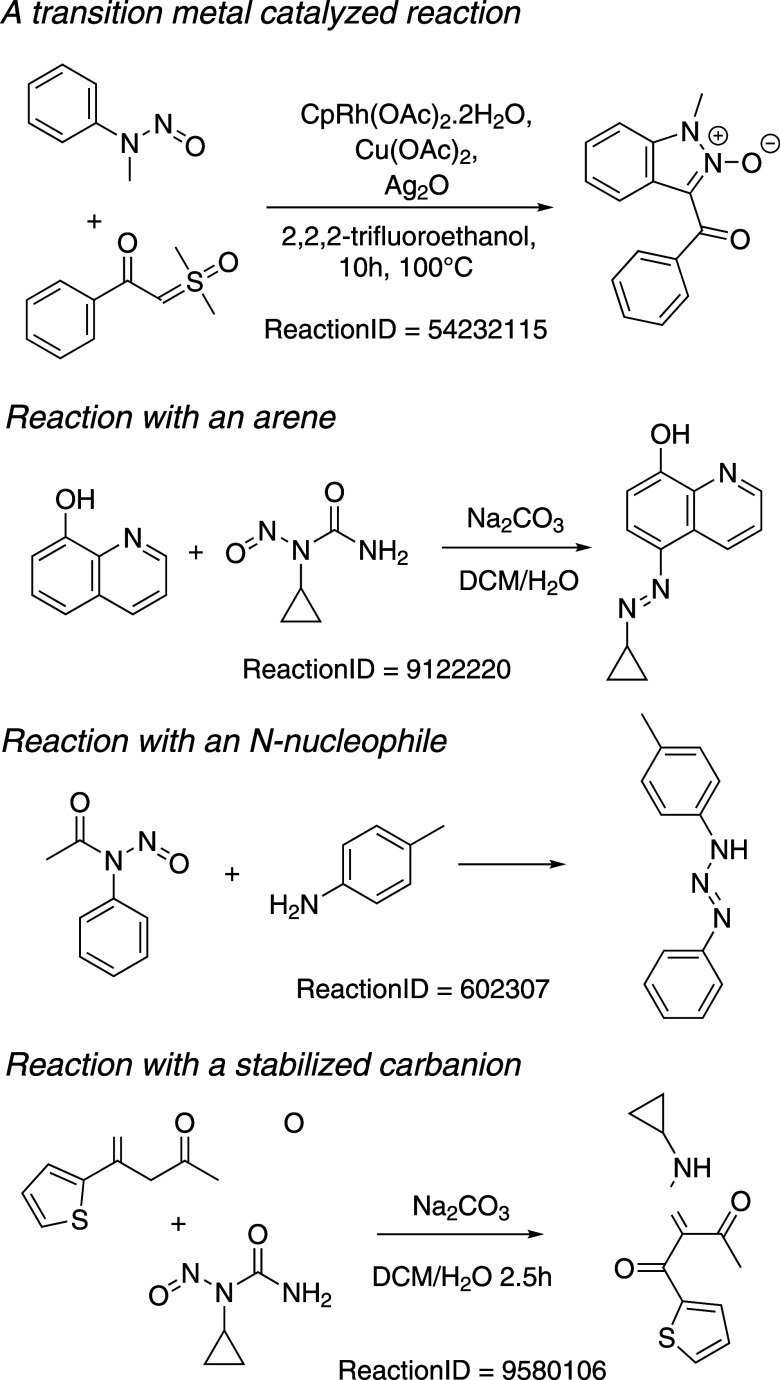
Examples of Reactions of Other Classes of NSAs Not Covered by Swager and Borths and their Reaxys Reaction ID Numbers

An analysis of the functional groups of the NSA starting materials is summarized in Figure 2a,b. While the regulatory important dialkyl-NSAs are significantly represented (116 reactions), there are many other classes of NSAs in the ***NSA_data set***, with alkyl,phenyl-NSAs being the most abundant (phenyl stands for any aryl groups). Thus, the data set represents a wide range of NSAs and their reactivity. Figure 2d shows the grouped transformations and their reactions with the NSAs in ***NSA_data set***. Many of these classes of NSAs have electron-withdrawing functional groups next to the N–N=O group, which enables class-specific reactions such as formation of diazonium salts with bases (32 reactions with alkyl,amide-NSAs, 16 reactions with alkyl,carbamate-NSAs, 24 reactions with alkyl,guanidine-NSAs, 39 reactions with alkyl,sulfone-NSAs and 62 reactions with alkyl,urea-NSAs (Supporting Information, Section S2.3, Figures S14, S17, S18, S20, and S21). While these are not strictly NSAs and have a different potential carcinogenic mechanism,^19^ they are referred to as NSAs in the article for simplicity. Most of the reported reactions of *N*,*N*-diphenylnitrosamine are Fischer–Hepp reactions (migration of the nitroso group to the phenyl ring) under strongly acidic conditions. In addition, there are a large number of literature precedents that employ reducing conditions including dissolving metal and hydride reagents. These classes of reagents are linked with reduction to amines and hydrazines and diazonium formation (most commonly done with alkyl,urea-NSAs)^20−22^ (Figure 2d). Although the documented reagents are reasonably diverse, the transformations that they achieve are limited. In-depth analysis of the formation of diazonium salts from NSAs under basic conditions showed that this transformation does not always require an electron-withdrawing group next to the −N–N=O functional group, e.g., Reaxys reaction ID = 240,906 (see Supporting Information, Section S2.4 and Scheme S4). This methodical and complete survey of the reported reactions of NSAs also identified a small number of reactions which were not covered in previous reviews by Swager and Borths, which focused on NSAs with alkyl and aryl substituents and did not include electrophilic NSAs:^8,9^ reactions with transition-metal catalyst, arenes, N-nucleophiles, and stabilized carbanions. Examples of these reactions are listed in Scheme 2.

### Reclassification of NSAs Based on their Reactivity in the Literature

**Figure 3:**
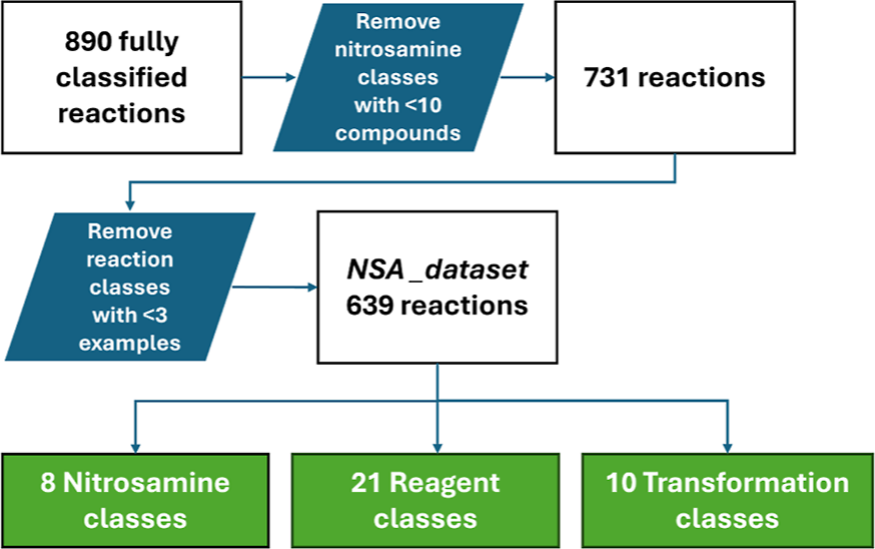
Workflow for reclassification and streamlining of the data set.

**Figure 4:**
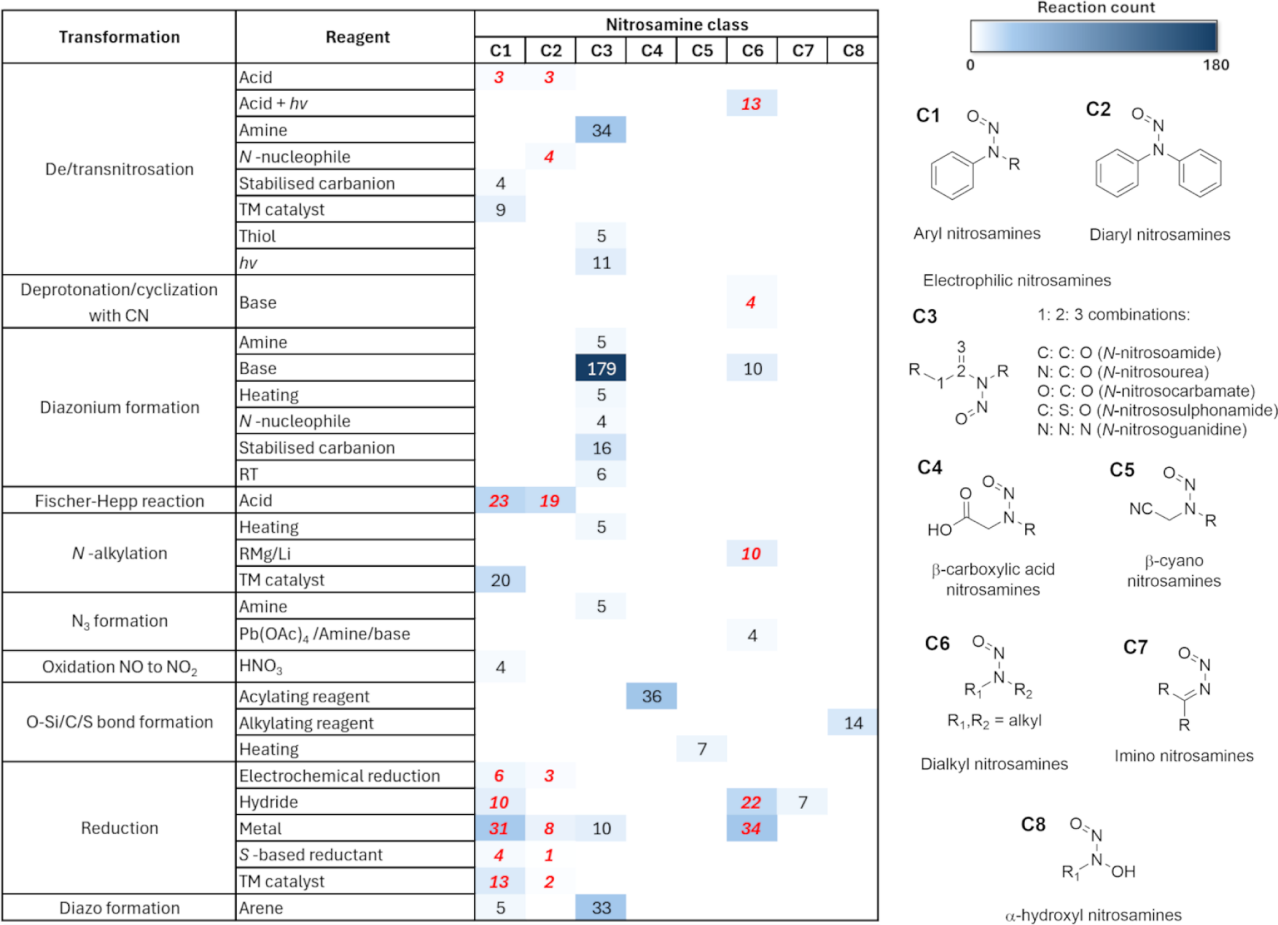
Classification table for major reactions of NSAs from Reaxys. The numbers represent the reaction counts in each case, with ***red***, italic numbers representing reactions which were listed in previous reviews.^8,9^ Reaction counts lower than 3 were included for class **C2** due to the lower total count for this class, these highlight the similar reactivity between **C1** and **C2**.

Based on the reactivity described in Figure 2, the classes of NSAs were rearranged to reflect their similar reactivity with common reagents in the ***NSA_data set***. The new classification and process are described in Figure 3. This was achieved by combining the nitrosamines into 8 classes (**C1–8**, Figure 4), reagents/conditions into 21 classes, and transformations into 10 classes. The streamlining of classification resulted in a loss of 251 reactions which belong to classes with fewer examples (<10 nitrosamines or <3 transformations) and a small number of reactions being included in more than 1 class of the substrate. On the other hand, it provides a straightforward identification of the most common reactions for each type of NSA through the use of Figure 4 at the cost of a small number of much less common transformations. The reaction classes which were reported by Swager and Borths are highlighted in red numbers^8,9^ and accounted for 19 entries out of 43 in Figure 4.

Importantly, the analysis of the reactions in ***NSA_data set*** showed that reported reactions in the literature do not distribute evenly among different classes of NSA, reagents, and transformations, and there are many gaps in-between, which one cannot confidently conclude whether they are due to lack of activity or lack of prior investigation (Figure 2a). The reported acidic and basic conditions are also typically harsh. Examples include (i) strong acids at high concentrations required for Fischer–Hepp reactions^23^ and (ii) strong bases for diazonium formation with NSAs which do not have an adjacent urea/carbamate functional group,^24^ H_2_/Raney-Ni, Zn/HCl, Zn/AcOH, and LiAlH_4_ for reduction of NSAs to hydrazines and amines.^25−28^ The most promising, and potentially general reaction, for destroying trace contamination of NSAs in APIs are reductions with inorganic S-based reductants, e.g., Na_2_S_2_O_4_ and TDO (acute toxicity by inhalation).^10,11^ Nevertheless, the reported substrate scopes with these reagents were somewhat limited: 6 NSAs with Na_2_S_2_O_4_ (dibenzyl-NSA and 5 aromatic-NSAs) and 23 NSAs with TDO (dibenzyl-NSA and 22 NSAs containing an aryl-NSA substructure). Finally, they did not include any alkyl,alkyl-NSA, which is the most important class of NSA from a regulatory point of view. Thus, there is a clear need for a systematic investigation on reactivity of a wide range of NSAs with common reagents in organic synthesis and workup/purification.

### Reactivity of NSAs with Common Reagents in Organic Reactions and Workup

**Figure 5:**
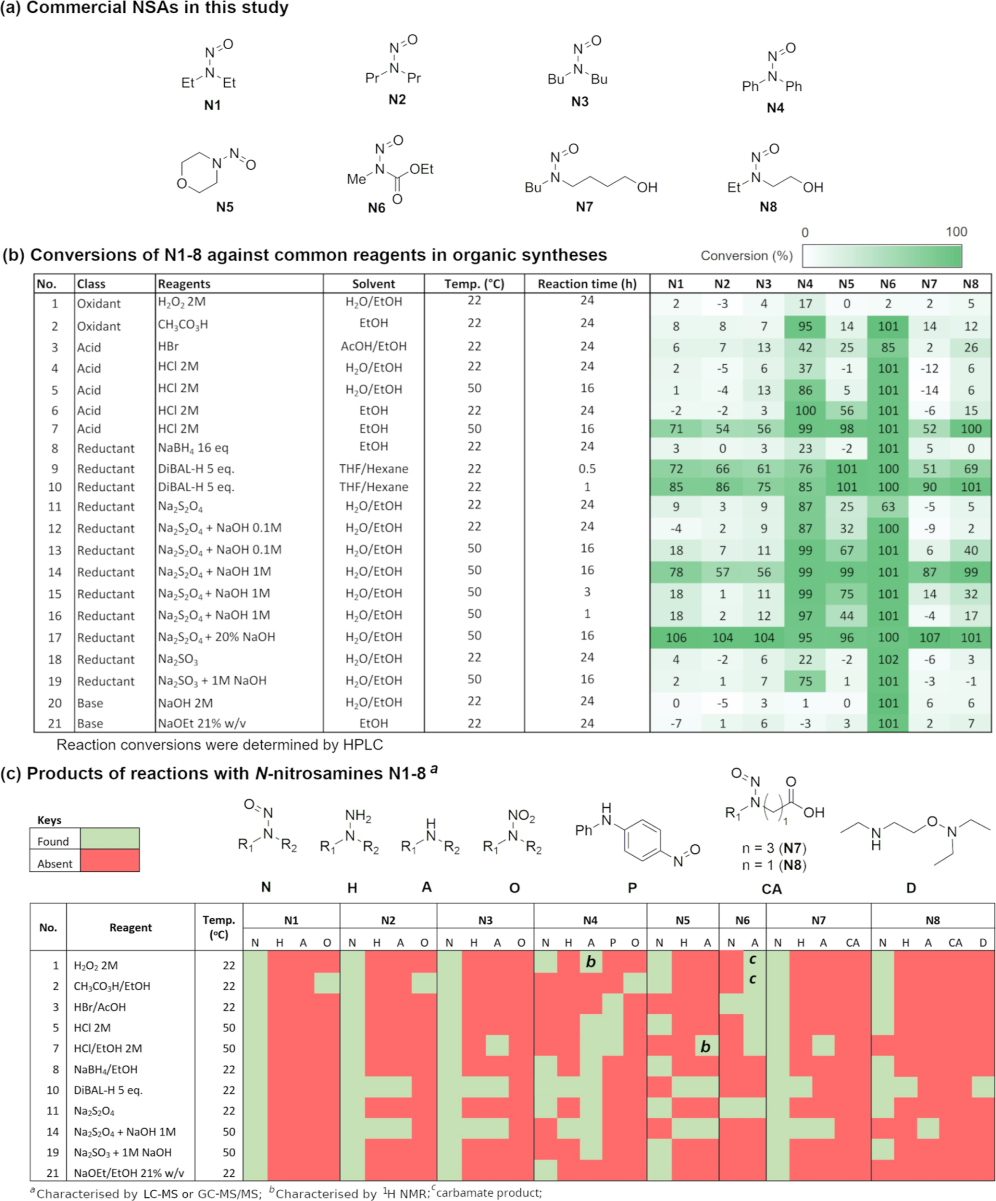
(a) Commercial NSAs employed in this study; (b) reaction screening results with common reagents in organic syntheses; and (c) products from reaction screening with common reagents in organic syntheses.

Eight commercial NSAs were sourced from common chemical suppliers for this study (Figure 5a). These are mainly the alkyl,alkyl-NSAs which are most important from a regulatory standpoint, with one alkyl,carbamate-NSA (**N6**) and diphenyl-NSA (**N4**). Three of these NSAs have hydroxyl groups at the β- or δ-positions. These substrates represent a different distribution of NSAs compared to that of the ***NSA_data set***, with a better representation of the electron-rich NSAs. Electron-rich NSAs are associated with carcinogenicity in the literature,^29^ although hydroxyl groups at the β-position are considered to reduce carcinogenic potency.^19^ A set of reaction conditions was initially selected based on those employed in the Mirabilis tool to estimate purge factor for contaminants in final APIs (Table 1).^12^ These common conditions were carefully examined and removed based on 4 criteria: (i) reaction conditions which are already well-represented in the ***NSA_data set*** (>5 reactions) or prior reviews,^8,9^ i.e., Grignard reagents, LiAlH_4_, NaOEt/EtOH, *n*-BuLi, and H_2_/Raney-Ni; (ii) reaction conditions for which rate constants have been reported, i.e., H_2_/10% Pd/C and H_2_/5% Pd/C; (iii) reaction conditions which are too harsh and indiscriminate for late-stage synthesis where many sensitive functional groups are present, i.e., NaClO_4_, BH_3_, and O_3_; and (iv) reaction conditions that are not commonly used in the later stage of API syntheses, e.g., TEMPO, which has only 8 examples in *Org. Process Res. Dev.* between 2010 and 2020 based on Reaxys. Given the potential chemoselectivity of sulfur-based reductants, Na_2_S_2_O_4_ and Na_2_SO_3_ were included, with and without NaOH and heating, in the reactivity screening in our study (Figure 5b).

**Table 1:** Reaction Conditions Based on the Mirabilis Tool for Reactivity Screening with Commercial NSAs

no.	reagents	solvent	reactivity screen	reason for removal	no reaction
1	BnSH		no	acute toxicity (inhalation)	
2	BnNH_2_	isooctane	no	literature data^a^	
3	EtMgBr	diethyl ether	no	literature data^31^	
4	DBU	DCM/THF	no	overlap with NaOEt/EtOH	
5	NaOH (10%)	H_2_O	yes		
6	NaHCO_3_ sat.	H_2_O	yes		yes
7	NaOEt (21%)	EtOH	yes		
8	*n*BuLi	hexane/THF	no	literature data^32^	
9	HCl 2 M		yes	literature data, limited scope^33^	
10	H_2_SO_4_ 95%	none	no	harsh conditions	
11	H_2_SO_4_ 2 M	H_2_O	yes	literature data^33^	yes^b^
12	HBr 33%	AcOH	yes		
13	HCl	EtOH	yes		
14	NaBH_4_	EtOH	yes		
15	LiAlH_4_	THF	no	literature data^34^	
16	DIBAL-H	THF	yes		
17	BH_3_		no	harsh conditions	
18	H_2_, 10% Pd/C	EtOH, iPrOH	no	literature data^35,36^	
19	H_2_, 5% Pd/C	EtOH, iPrOH	no	literature data^9^	
20	H_2_, Raney Ni		no	literature data^25^	
21	NaClO_4_		no	harsh conditions	
22	H_2_O_2_ 30%	H_2_O	yes		
23	AcOOH 40%	H_2_O	yes		
24	KMnO_4_	DCM	no	literature data^37^	
25	IBX/DMP	DCE/MeCN	yes		yes^b^
26	TEMPO	MeCN	no	less common conditions	
27	*m*CPBA	DCE/MeCN	yes		yes^b^
28	O_3_	THF	no	harsh conditions	

a Literature reactions with BnNHR resulting in transnitrosation.

b Reaction performed in the continuous mode using Vapourtec R-series with internal static mixers.

Initial reactivity screening was performed in flow, using a Vapourtec flow reactor with static mixers, in order to minimize and contain the inventory of hazardous NSAs. However, most of the reaction conditions are biphasic with a long reaction time, and continuous and efficient mixing is required to ensure fast and reproducible reactions. Thus, subsequent reactions, which are summarized in Figure 5b, were performed in the batch mode. The reaction mixtures were quenched (see Supporting Information, Section S3.3) and analyzed with HPLC, GC–MS/MS, and LC–MS. The reaction conversions were calculated based on the consumption of the NSAs. The solvents were selected based on common solvents for the desired reactions, their miscibility, and solubility of the NSAs in them.

In some cases, conversion was reported in Figure 5b and no product was found by either GC–MS/MS or LC–MS/MS (Figure 5c). For example, in row 7 (2 M HCl/EtOH, 50 °C, 16 h) and 14 [Na_2_S_2_O_4_ (1 M NaOH/EtOH), 50 °C, 16 h], the products of **N1**, **N2**, and **N8** were not observed. Where the products of higher molecular weight NSAs were successfully captured and assigned based on GC–MS/MS and LC–MS data, the unobserved products of the lower molecular weight NSAs were accordingly assumed. In some cases, particularly with **N1**, significant conversion was achieved under reaction conditions 7, 9, 10, 14, and 17, but no product was observed. This is attributed to the limitation of our analysis regime to capture and characterize volatile products and the loss of some products due to aqueous workup (described in Supporting Information, Section S3.2.3). Substrate **N6** displayed activity under all reaction conditions, except H_2_O_2_, but also no detected product. Our literature analysis above suggested that it is a highly activated NSA toward formation of the reactive methyldiazonium cation, leading to products with low molecular weights and significant aqueous solubility.

Treatment of the NSAs with aqueous 2 M HCl resulted in no reaction except with **N4** and **N6**. **N6** was completely converted to the amine product, while the conversion was only moderate (37%) with **N4** after 24 h at room temperature. Gentle heating at 50 °C improved the conversion to 86%, giving the expected mixture of amine and transnitrosation products (Figure 5c). Williams and co-workers suggested that a strong nucleophile would improve the reactivity of nitrosamine under acidic conditions.^33^ Thus, the solvent was changed from water to EtOH to reduce the level of solvation of the chloride anion and to improve its nuleophilicity. This led to an increase of conversion with **N4** to 100% at room temperature after 24 h. In addition, **N5** also gave 56% conversion to the amine product. Increasing the temperature to 50 °C led to reactions with all 8 NSAs, with higher reactivity observed for **N4**, **N5**, **N6**, and **N8** (Figure 4b, row 7). The more acidic reagent 33% HBr in AcOH did not show improved reactivity compared to HCl in EtOH with **N4**, **N5,** and **N8** at room temperature, and little product was observed with the rest of the NSAs (row 3).

Oxidative conditions were heavily under-represented in the ***NSA_data set***, with only 12 reported reactions using reagents such as H_2_O_2_ with Ac_2_O, organic peroxide, and NaClO_4_. Thus, two simple oxidants were included in the reactivity screening: aqueous 2 M H_2_O_2_ and AcOOH in EtOH. The latter is much more reactive with organic compounds,^38^ with a mechanism based on transfer of an oxygen atom instead of a hydroxyl radical or a peroxide anion.^39^ While little reactivity was observed with aqueous 2 M H_2_O_2_ at room temperature with all NSAs (Figure 5b, row 1). AcOOH showed near complete conversion with **N4** and **N6** after 24 h, and some conversion with other NSAs (Figure 5b, row 2). Substrates **N7** and **N8**, which contains oxidizable primary alcohol functional groups showed no reaction with aqueous 2 M H_2_O_2_ and low conversions with AcOOH, 14% and 12%, respectively. These results are consistent with the lack of report of noncatalytic oxidation of alcohols with H_2_O_2_ or AcOOH under ambient conditions in the literature. Heating was not applied given the potential hazards of oxidants at a high temperature. With **N4** as the substrate, AcOOH gave the expected *N*-nitroamine (O, Figure 5c) product, but only amine was observed with H_2_O_2_. This is attributed to the reagent solution being slightly acidic and therefore invoking denitrosation. **N6** gave the amine product with both peroxide reagents, highlighting its poor stability in acidic conditions (Figure 5c).

Strong reductants such as LiAlH_4_ and Zn/HCl are well documented as effective reagents for reducing the NSA group to hydrazines and amines.^8,9^ However, these are not mild reagents and may not be compatible with a wide range of functional groups at a late stage of synthesis. Thus, we included reaction screening for **N1–8** with NaBH_4_ and DIBAL-H in their usual solvents. NaBH_4_ showed no reactivity with most NSAs, low conversion with **N4,** and complete reduction of **N6** (Figure 5b, row 8). NaBH_4_ is a mild reductant and normally does not reduce a carbamate functional group. The electron-withdrawing group −N=O manifests increased reactivity of the carbamate group, consistent with the observed disappearance of the C=O stretching IR signal when **N6** was subjected to NaBH_4_ (see Supporting Information, Section S8). Hence, no product was observed for **N6** by GC–MS/MS with NaBH_4_ and the stronger reductant DIBAL-H. DIBAL-H was effective to react with all NSAs, giving high conversions after 30 min at room temperature (Figure 5b, rows 9 and 10), providing the corresponding hydrazine and amine in most cases. The lack of detected product for **N1**, despite evidence of consumption by HPLC, was attributed to the volatility of diethylamine and aqueous solubility of other products used during the workup. Interestingly, reduction of **N8** with DIBAL-H resulted in an unexpected product, which was tentatively assigned as **D** based on LC–MS and GC–MS data (Supporting Information, Section S9.5).

The sulfur-based reductants were included in addition to the original Mirabilis conditions (Table 1), due to their mild conditions and lack of activity with the most common organic functional groups. The aqueous Na_2_S_2_O_4_ reagent is of particular interest, due to its tunable redox potential, depending on the pH of the solution (eq 1).^40−42^ The standard redox potential *E*^0^ can vary from 0.07 to −1.12 V at pH = 0 to 14.
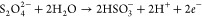
1

Consequently, the NSAs were treated with Na_2_S_2_O_4_ under both neutral and basic conditions (NaOH 0.1, 1 M, and 20%). The results are summarized in Figure 5b, row 11–17. Changing from Na_2_S_2_O_4_ to aqueous Na_2_SO_3_ (Figure 5b, row 18) led to no conversion with all NSAs but **N4** (22% after 24 h) and **N6** (100% conversion). The corresponding amines and hydrazines were observed as expected products (Figure 5c). To rule out the reactions mediated by basic conditions alone, all 8 NSAs were treated with aqueous 2 M NaOH and 21% NaOEt/EtOH (Figure 5b, rows 19 and 20). These resulted in no significant reaction with any NSAs other than **N6**. Compound **N6** contains an electron-withdrawing group next to the −N–N=O functional group, which activates it to produce diazonium salts under basic conditions.^43^ These control experiments confirm the treatment of NSAs with Na_2_S_2_O_4_ in aqueous NaOH 1 M at 50 °C as an effective method for removing NSA contamination. The only reported reactions of Na_2_S_2_O_4_ with organic compounds without a metal catalyst are reduction of an aromatic nitro or nitroso group to an amine,^44,45^ conversion of alkyl halides to sodium sulfonates,^46^ and reductive cleavage of N–S bonds,^47^ suggesting excellent functional group tolerance for this treatment. Base-sensitive functional groups, i.e., incompatible with NaOH, such as aldehydes and ketones, are also uncommon in APIs. It is worth noting that the products of the reactions between NSAs and Na_2_S_2_O_4_ are amines and hydrazines, which have their own hazards and toxicity issues, albeit of lesser concerns than those of NSAs.

Finally, a general trend in reactivity was observed across the 8 NSAs. Electron-rich NSAs, such as **N1–3** (dialkyl-NSAs) and **N7** (with a hydroxyl group at the δ position), displayed the lowest reactivity, while **N4** (*N*,*N*-diphenylnitrosamine) and **N6** (electron-poor NSA with a −COOMe group on the *N* −1 position) are the most reactive. This is important when it comes to extrapolating the reactivity of electron-rich NSAs based on the known reactivity of safer NSAs such as **N4**. The presence of an oxygen atom at the β-position to the −N–N=O group improves the reactivity of the NSA, as demonstrated with **N8** vs **N1–3**, and with **N5** which was the third most reactive NSA, after **N4** and **N6**. Computational investigation of the influence of this oxygen atom at the β-position is ongoing and will be reported in due course.

## Conclusions

We have carried out a comprehensive analysis of literature reaction data of NSAs using cheminformatics tools. The main reactivity trends were highlighted, and the NSAs were classified based on the analysis, with common transformations and reagents for each NSA class summarized in Figure 4. Importantly, the data show that *N*,*N*-dialkyl nitrosamines are the least reactive and often only react with highly reactive reagents or under harsh reaction conditions. Based on these insights, reactivities of a diverse range of eight NSAs with common organic reagents and workup conditions were measured. These are important in assessing the residual amount of possible NSAs in medicinal products. The results showed Na_2_S_2_O_4_ in aqueous NaOH 1 M at 50 °C to be highly effective at reducing NSAs to the parent amine with a limited alteration of other functional groups.

## Supporting Information Available

Reaction data of NSAs, experimental procedures, and characterization data of reactions and products (PDF)

## References

[ref1] Suspension of Ranitidine Medicines in the EU; EMA, 2020. https://www.ema.europa.eu/en/news/suspension-ranitidine-medicines-eu (accessed Nov 25, 2023).

[ref2] Guidance for Marketing Authorisation Holders; European Medicines Agency, 2022. https://www.ema.europa.eu/en/human-regulatory-overview/postauthorisation/pharmacovigilance-post-authorisation/referral-procedures-human-medicines/nitrosamine-impurities#guidance-for-marketing-authorisation-holders-13196 (accessed Oct 20, 2022).

[ref3] Control of Nitrosamine Impurities in Human Drugs: Guidance for Industry; U.S. Food and Drug Administration, 2020. https://www.fda.gov/regulatory-information/search-fda-guidance-documents/control-nitrosamine-impurities-human-drugs (accessed Oct 20, 2022).

[ref4] López-Rodríguez R.; McManus J. A.; Murphy N. S.; Ott M. A.; Burns M. J. Pathways for *N*-Nitroso Compound Formation: Secondary Amines and Beyond. Org. Process Res. Dev. 2020, 24, 1558–1585. 10.1021/acs.oprd.0c00323.

[ref5] Ashworth I. W.; Curran T.; Dirat O.; Zheng J.; Whiting M.; Lee D. A Consideration of the Extent that Tertiary Amines Can Form *N*-Nitroso Dialkylamines in Pharmaceutical Products. Org. Process Res. Dev. 2023, 27, 1714–1718. 10.1021/acs.oprd.3c00073.

[ref6] Murphy N. S.; O’Connor D. C.; Gavins G. C.; James L.; Lockett J. P.; McManus J. A.; Packer G.; Lopez-Rodríguez R.; Webb S. J.; Burns M. J. Identifying the Risk of Formation of Nitrosamines and Other Potentially Mutagenic Impurities during API Manufacture Using In Silico Risk Assessment. Org. Process Res. Dev. 2023, 27, 1812–1819. 10.1021/acs.oprd.3c00118.

[ref7] Martin R.; Tashdjian M. The Polarographic Reduction of *N*-Nitrosamines. J. Phys. Chem. 1956, 60, 1028–1030. 10.1021/j150542a002.

[ref8] Beard J. C.; Swager T. M. An Organic Chemist’s Guide to *N*-Nitrosamines: Their Structure, Reactivity, and Role as Contaminants. J. Org. Chem. 2021, 86, 2037–2057. 10.1021/acs.joc.0c02774. 33474939 PMC7885798

[ref9] Borths C. J.; Burns M.; Curran T.; Ide N. D. Nitrosamine Reactivity: A Survey of Reactions and Purge Processes. Org. Process Res. Dev. 2021, 25, 1788–1801. 10.1021/acs.oprd.1c00162.

[ref10] Chaudhary P.; Gupta S.; Sureshbabu P.; Sabiah S.; Kandasamy J. A Metal Free Reduction of Aryl-*N*-Nitrosamines to the Corresponding Hydrazines using a Sustainable Reductant Thiourea Dioxide. Green Chem. 2016, 18, 6215–6221. 10.1039/C6GC02444K.

[ref11] Overberger C. G.; Lombardino J. G.; Hiskey R. G. Novel Reductions of *N*-Nitrosodibenzylamines—A New Reaction. J. Am. Chem. Soc. 1958, 80, 3009–3012. 10.1021/ja01545a028.

[ref12] Burns M. J.; Teasdale A.; Elliott E.; Barber C. G. Controlling a Cohort: Use of Mirabilis-Based Purge Calculations to Understand Nitrosamine-Related Risk and Control Strategy Options. Org. Process Res. Dev. 2020, 24, 1531–1535. 10.1021/acs.oprd.0c00264.

[ref13] Rahman S. A.; Torrance G.; Baldacci L.; Martínez Cuesta S.; Fenninger F.; Gopal N.; Choudhary S.; May J. W.; Holliday G. L.; Steinbeck C.; et al. Reaction Decoder Tool (RDT): Extracting Features from Chemical Reactions. Bioinformatics 2016, 32, 2065–2066. 10.1093/bioinformatics/btw096. 27153692 PMC4920114

[ref14] Coley C. W.; Barzilay R.; Jaakkola T. S.; Green W. H.; Jensen K. F. Prediction of Organic Reaction Outcomes Using Machine Learning. ACS Cent. Sci. 2017, 3, 434–443. 10.1021/acscentsci.7b00064. 28573205 PMC5445544

[ref15] Segler M. H. S.; Waller M. P. Neural-Symbolic Machine Learning for Retrosynthesis and Reaction Prediction. Chem.—Eur. J. 2017, 23, 5966–5971. 10.1002/chem.201605499. 28134452

[ref16] Christ C. D.; Zentgraf M.; Kriegl J. M. Mining Electronic Laboratory Notebooks: Analysis, Retrosynthesis, and Reaction Based Enumeration. J. Chem. Inf. Model. 2012, 52, 1745–1756. 10.1021/ci300116p. 22657734

[ref17] Lin A.; Dyubankova N.; Madzhidov T. I.; Nugmanov R. I.; Verhoeven J.; Gimadiev T. R.; Afonina V. A.; Ibragimova Z.; Rakhimbekova A.; Sidorov P.; et al. Atom-to-Atom Mapping: A Benchmarking Study of Popular Mapping Algorithms and Consensus Strategies. Mol. Inf. 2022, 41, 2100138 10.1002/minf.202100138. 34726834

[ref18] Delannée V.; Nicklaus M. C. ReactionCode: Format for Reaction Searching, Analysis, Classification, Transform, and Encoding/Decoding. J. Cheminf. 2020, 12, 72 10.1186/s13321-020-00476-x. PMC7713369 33292568

[ref19] Carcinogenic Potency Categorisation Approach for N-Nitrosamines; EMA, 2023. https://www.ema.europa.eu/system/files/documents/other/appendix_2_carcinogenic_potency_categorisation_approach_for_n-nitrosamines_en.pdf (accessed Aug 07, 2024).

[ref20] Wu Y.; Feng L. J.; Lu X.; Kwong F. Y.; Luo H. B. Palladium-Catalyzed Oxidative C–H Bond Acylation of *N*-Nitrosoanilines with Toluene Derivatives: A Traceless Approach to Synthesize *N*-Alkyl-2-Aminobenzophenones. Chem. Commun. 2014, 50, 15352–15354. 10.1039/c4cc07440h. 25348462

[ref21] Yang W.; Lu X.; Zhou T.; Cao Y.; Zhang Y.; Ma M. Selective Reduction of *N*-Nitroso Aza-Aliphatic Cyclic Compounds to the Corresponding *N*-Amino Products using Zinc Dust in CO_2_–H_2_O Medium. Chem. Heterocycl. Compd. 2018, 54, 780–783. 10.1007/s10593-018-2349-0.

[ref22] Berhe S.; Slupe A.; Luster C.; Charlier H. A. Jr.; Warner D. L.; Zalkow L. H.; Burgess E. M.; Enwerem N. M.; Bakare O. Synthesis of 3-[(*N*-Carboalkoxy) Ethylamino]-indazole-dione Derivatives and their Biological Activities on Human Liver Carbonyl Reductase. Bioorg. Med. Chem. 2010, 18, 134–141. 10.1016/j.bmc.2009.11.011. 19959367 PMC2821159

[ref23] D’Amico J. J.; Tung C. C.; Walker L. A. Nitrosoanilines. J. Am. Chem. Soc. 1959, 81, 5957–5963. 10.1021/ja01531a029.

[ref24] Lee P. A Method for the Preparation of Diazoalkanes. WO 2013110932 A1, 2013.

[ref25] Grillot G. F. The Reduction of Diphenylnitrosamine in the Presence of Raney Nickel Catalyst and Platinum Catalyst1. J. Am. Chem. Soc. 1944, 66, 2124 10.1021/ja01240a503.

[ref26] Denmark S. E.; Chang W.-T. T.; Houk K.; Liu P. Development of Chiral Bis-hydrazone Ligands for the Enantioselective Cross-Coupling Reactions of Aryldimethylsilanolates. J. Org. Chem. 2015, 80, 313–366. 10.1021/jo502388r. 25494058 PMC4285162

[ref27] Sanaa P.; Savelli F.; Cignarella G. Synthesis of Cis- and Trans-1,3-Dicarbomethoxy-2-aminoisoindoline. J. Heterocycl. Chem. 1981, 18, 475–478. 10.1002/jhet.5570180307.

[ref28] Fusco R.; Sannicolo F. Rearrangement of Arylhydrazones of .Alpha., Beta.-Unsaturated Carbonyl Compounds in Polyphosphoric Acid. 6. J. Org. Chem. 1984, 49, 4374–4378. 10.1021/jo00197a009.

[ref29] Schlingemann J.; Burns M. J.; Ponting D. J.; Martins Avila C.; Romero N. E.; Jaywant M. A.; Smith G. F.; Ashworth I. W.; Simon S.; Saal C.; et al. The Landscape of Potential Small and Drug Substance Related Nitrosamines in Pharmaceuticals. J. Pharm. Sci. 2023, 112, 1287–1304. 10.1016/j.xphs.2022.11.013. 36402198

[ref30] Garcia-Rio L.; Leis J. R.; Pena M. E.; Iglesias E. Transfer of the Nitroso Group in Water/AOT/Isooctane Microemulsions: Intrinsic and Apparent Reactivity. J. Phys. Chem. 1993, 97, 3437–3442. 10.1021/j100115a057.

[ref31] Farina P. R.; Tieckelmann H. Reactions of Grignard Reagents with Nitrosamines. J. Org. Chem. 1975, 40, 1070–1074. 10.1021/jo00896a016.

[ref32] Vazquez A. J.; Rodriguez C.; Nudelman N. S. Convenient Methodology for the Synthesis of Trialkylhydrazines. Synth. Commun. 2009, 39, 3958–3972. 10.1080/00397910902738120.

[ref33] Hallett G.; Johal S.; Meyer T.; Williams D. Reactions of Nitrosamines with Nucleophiles in Acid Solution; IARC Scientific Publications, 1980; pp 31–41. 7228262

[ref34] Golubev P.; Krasavin M. *N*-Isocyanodialkylamines Generated In Situ for the Joullié–Ugi Reaction with Indolenines. Tetrahedron Lett. 2018, 59, 3532–3536. 10.1016/j.tetlet.2018.08.025.

[ref35] Grošelj U.; Bevk D.; Jakše R.; Meden A.; Stanovnik B.; Svete J. Stereoselective Additions to the Exocyclic C=C Bond of Some α-Alkylidene-(+)-Camphor Derivatives. Tetrahedron: Asymmetry 2006, 17, 1217–1237. 10.1016/j.tetasy.2006.04.014.

[ref36] Kakeya H.; Imoto M.; Takahashi Y.; Naganawa H.; Takeuchi T.; Umezawa K. Dephostatin, a Novel Protein Tyrosine Phosphatase Inhibitor Produced by Streptomyces II. Structure Determination. J. Antibiot. 1993, 46, 1716–1719. 10.7164/antibiotics.46.1716. 8270494

[ref37] Saavedra J. E.; Temu C. T.; Farnsworth D. W. Oxidation of β-Hydroxynitrosamines to β-Ketonitrosamines with KMnO_4_-Metal ·SO_4_·XH_2_O in Non-Aqueous Media. Synth. Commun. 1989, 19, 215–220. 10.1080/00397918908050972.

[ref38] Kim J.; Huang C.-H. Reactivity of Peracetic Acid with Organic Compounds: A Critical Review. ACS ES&T Water 2021, 1, 15–33. 10.1021/acsestwater.0c00029.

[ref39] Targhan H.; Evans P.; Bahrami K. A Review of the Role of Hydrogen Peroxide in Organic Transformations. J. Ind. Eng. Chem. 2021, 104, 295–332. 10.1016/j.jiec.2021.08.024.

[ref40] Mayhew S. G. The Redox Potential of Dithionite and SO_2_ from Equilibrium Reactions with Flavodoxins, Methyl Viologen and Hydrogen plus Hydrogenase. Eur. J. Biochem. 1978, 85, 535–547. 10.1111/j.1432-1033.1978.tb12269.x. 648533

[ref41] McMillan W. Jr.; Roberts J. D.; Coryell C. D. The Thermodynamic Constants of the Dithionite (Hydrosulfite) Ion. J. Am. Chem. Soc. 1942, 64, 398–399. 10.1021/ja01254a047.

[ref42] Latimer W. M. The Oxidation States of the Elements and their Potentials in Aqueous Solutions; Prentice Hall: New York, 1952; p 76.

[ref43] Muller E.; Petersen S. R. Aliphatische Diazo- und Azoverbindungen in der Kunststoffchemie. Angew. Chem. 1951, 63, 18–20. 10.1002/ange.19510630105.

[ref44] Espinosa S.; Solivan M.; Vlaar C. P. Synthesis and Redox-Enzyme Modulation by Amino-1,4-Dihydro-Benzo[*d*][1,2] Dithiine Derivatives. Tetrahedron Lett. 2009, 50, 3023–3026. 10.1016/j.tetlet.2009.03.209. 20161292 PMC2697396

[ref45] Sanofi Antagonist Derivatives of the Vitronectin Receptor. U.S. Patent 20,040,225,111 A1, 2001.

[ref46] JSR Acid Generator, Sulfonic Acid, Sulfonic Acid Derivative, Halogen-Containing Bicyclooctane Compound and Radiation-Sensitive Resin Composition. J.P. Patent 2,005,112,724 A, 2003.

[ref47] AstraZeneca Sulfur Containing Diamides and Antimicrobial Use. U.S. Patent 5,224,980 A, 1993.

