# Supplementary material for: Reactivities of N-Nitrosamines against Common Reagents and Reaction Conditions
Source: Org Process Res Dev. 2024 Sep 18;28(10):3837–46. doi: 10.1021/acs.oprd.4c00217 (PMC11494645; doi:10.1021/acs.oprd.4c00217)
Supplement: Supplementary file 1 — op4c00217_si_001.pdf [file op4c00217_si_001.pdf]

Reactivities of N-nitrosamines against common reagents and reaction conditions

George A. Hodgin,[†] Michael J. Burns,[‡] Benjamin J. Deadman,[¶] Christopher S. Roberts,[¶] King Kuok (Mimi) Hii,[¶] and Bao N. Nguyen[*,†]

†*School of Chemistry, University of Leeds, Woodhouse Lane, Leeds, LS2 9JT, United Kingdom*

‡*Lhasa Ltd., Granary Wharf House, 2 Canal Wharf, Leeds, LS11 5PS, United Kingdom*

¶*Centre for Rapid Online Analysis of Reactions, Molecular Sciences Research Hub, Imperial College London, 82 Wood Lane, London, W12 0BZ, United Kingdom*

E-mail: b.nguyen@leeds.ac.uk

Phone: +44 (0)113 3430109

Electronic supporting information

Contents

1 Data collection and curation **5**

1.1 Atom-atom mapping 5

1.2 ReactionCodes 6

1.3 Analysis of the reactions which failed reactionCode generation 7

1.4 Filtering irrelevant reactions and duplicates 10

2 **Literature reaction data analysis** **16**

2.1 Initial classification . . . 16

2.2 Re-classification based on the analysis of literature reaction data . . . 19

2.3 Heat maps for transformations and reagents of each class of *N*-nitrosamines . 24

2.4 Examples of organic transformations of *N*-nitrosamines . . . 39

3 **Reactivity screen of *N*-nitrosamines N1-8** **53**

3.1 Safety . . . 53

3.2 General procedures . . . 54

3.2.1 Flow reaction protocol . . . 54

3.2.2 Flow reaction analysis workflow . . . 56

3.2.3 Batch protocols . . . 57

3.3 Experimental conditions . . . 58

4 **Reaction screening results** **70**

4.1 Conversion calculation method and information . . . 70

4.2 Conversion tables . . . 72

4.3 Conversion summary table . . . 93

5 **Calibrations** **95**

5.1 HPLC Calibrations . . . 95

5.1.1 HPLC method . . . 95

5.1.2 HPLC calibration curve equations . . . 95

5.1.3 HPLC calibration curves . . . 96

5.2 GC Calibrations . . . 106

5.2.1 GC methods . . . 106

5.2.2 GC calibration curves . . . 107

6 **GC-MS/MS References** **115**

7 LC-MS/MS References **138**

8 Flow reaction results **157**

9 Reaction products by LC-MS/MS and GC-MS/MS **158**

9.1 GC-MS/MS and LC-MS/MS analysis 158

9.1.1 LC-MS/MS method 158

9.2 Oxidative conditions (1-2): 159

9.3 Acidic conditions (3-7): 166

9.4 Hydride reductant conditions (8-10): 179

9.5 Analysis of the products of N8 in conditions 9 and 10 187

9.6 Sulfur-based reductants conditions (11-19): 194

9.7 Basic conditions (20-21): 214

10 Spectroscopic data of reaction products **217**

10.1 NMR spectra 217

10.1.1 Diphenylamine ^{1}H NMR 218

10.1.2 Diphenylamine ^{13}C NMR 219

10.1.3 *N*-nitrosodiphenylamine (N4) ^{1}H NMR 220

10.1.4 *N*-nitrosodiphenylamine (N4) ^{13}C NMR 221

10.1.5 Condition 1 N4 ^{1}H NMR overlay 222

10.1.6 Condition 1 N4 ^{13}C NMR overlay 223

10.1.7 Condition 1 N4 ^{1}H NMR (Full) 224

10.1.8 Condition 1 N4 ^{1}H NMR (Zoomed) 225

10.1.9 Condition 1 N4 ^{13}C NMR (Full) 226

10.1.10 Condition 1 N4 ^{13}C NMR (Zoomed) 227

10.1.11 Morpholine ^{1}H NMR analysis 228

10.1.12 Morpholine ^{13}C NMR analysis 229

10.1.13 *N*-nitrosmorpholine (N5) ^{1}H NMR analysis 230

10.1.14 *N*-nitrosomorpholine (N5) ^{13}C NMR analysis 231

1 Data collection and curation

This section outlines the retrieval of the initial dataset of reactions, and the cheminformatics workflow used to filter the reactions to leave only those reactions which consumed N-nitrosamines.

A search of all reactions in the Reaxys database containing NMDA as a sub-structure of the starting material resulted in 15163 reactions, which were exported in XML format (Figure S1). For each reaction, SMILES strings for reactants and products, and reaction conditions, which included solvents and catalysts, were extracted with a Python script. The Python package ***rdkit*** was used to convert MDL Molfiles in the exported XML file into SMILES strings. This resulted in 11413 reactions with extractable information. After this, multi-step reactions were identified using the "RXD.STP" tag in the XML file. Reactions with RXD.STP > 1 were removed, leaving 11087 single-step reactions.

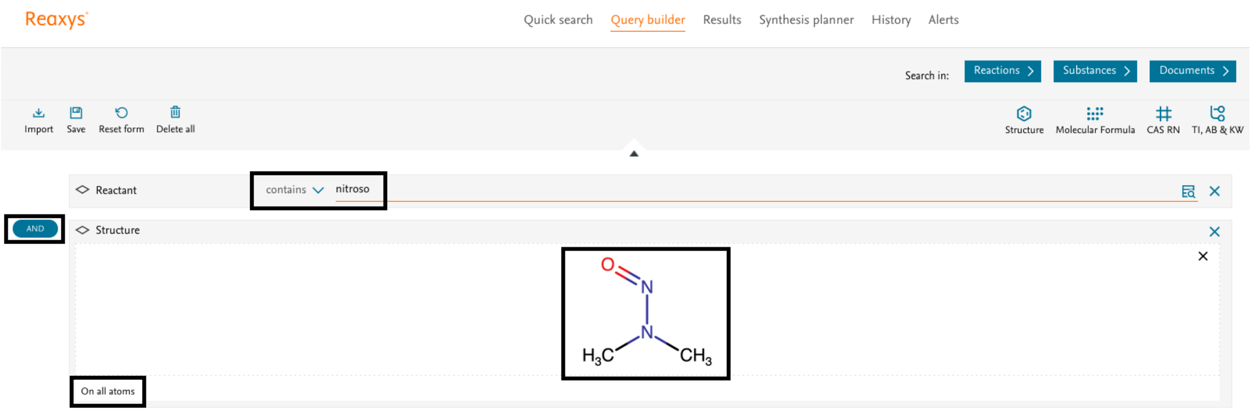

Figure S1: The search terms entered into Reaxys to retrieve the initial dataset.

1.1 Atom-atom mapping

In order to identify those reactions which consumed nitrosamines in some way, a cheminformatics workflow employing reaction centre extraction was employed. This began with the use of the Reaction Decoder Tool (RDT)[1] to perform atom-atom mapping (AAM) based on

the computed reaction SMILES strings. To perform the mappings, the RDT command line interface was supplied with the SMILES strings of all the unique reaction SMILES via a .bat file which was constructed in python (Listing S1).

Listing S1: Python code to generate RDT .bat script

```
# Construct AAM .bat file
with open('rdt.bat', 'w') as outfile:
    # Assign the file path for reaction decoder tool
    outfile.write("set rdtpath=%~dp0\n")
    # loop through every reaction string
    # using the Reaxys Reaction ID
    # as reference for file names
    for index, reaction_string in zip(reaction_index,
    reaction_smiles_Reaxys):
        outfile.write(
            # Make a new directory named after the Reaxys ID
            'mkdir "%rdtpath%rdt_1\\'
            + str(index)
            # Change into this directory
            + '" && cd "%rdtpath%rdt_1\\'
            + str(index)
            # Run the RDT CLI passing the reaction SMILES as input
            + r'" && java -jar "%rdtpath%rdt_2.5.0.jar" -Q SMI -q "'
            + reaction_string
            + '" -g -c -j AAM -f TEXT'
            # Start a new line for the next reaction
            + '\n'
            )
```

11069 reactions gave successful mappings. The .rxn output file from the RDT process contained a representation of the atom-mapped reaction, and this was used as the direct input to the ReactionCode software used in the next step of the cheminformatics workflow.

1.2 ReactionCodes

AAM was followed by application of the ReactionCode (version 1.2.2) software to encode the reactions into ReactionCodes which is a string-based language in which the reaction

centre can be portrayed at various depths from the atoms and bonds that change during a reaction.[2] The .rxn output file from the RDT AAM process containing the mapped reaction was accessed and passed as input to ReactionCode 1.2.2 to generate ReactionCodes for the 11069 reactions using another .bat file which was again generated in python (Listing S2).

Listing S2: Python code to generate ReactionCodes .bat script

```
# Construct .bat file for ReactionCode generation
with open('ReactionCode.bat', 'w') as outfile:
    # Assign the file path for ReactionCode.jar
    outfile.write("set reactioncodepath=%~dp0\n")
    # loop through every reaction string
    for index, reaction_string in zip(reaction_index,
    reaction_smiles_Reaxys):
        outfile.write(
            # Make a directory to save the results
            'mkdir "%reactioncodepath%ReactionCode_1\\'
            # Named after the Reaxys ID of the reaction
            + str(index)
            # Access the atom mapped .RXN file for that reaction
            + '" && cd "%reactioncodepath%rdt_1\\'
            + str(index)
            # Encode the reaction into a ReactionCode
            + r'" && java -jar "%reactioncodepath%ReactionCode_1.2.2.jar" '
            + ' -q ECBLAST_smiles_AAM.rxn -o '
            # Store output in the results folder
            + '"%reactioncodepath%ReactionCode_1\\'
            + str(index)
            # New line for next reaction
            + '\n'
            )
```

1.3 Analysis of the reactions which failed reactionCode generation

There were 5601 reactions which failed reactionCode generation. Analysis of the failed reactions revealed that unbalanced reactions were the main cause of reactionCode failure. The atom-atom mapping output images from the RDT are shown for some examples of failed reactions in Figures S2 and S3.

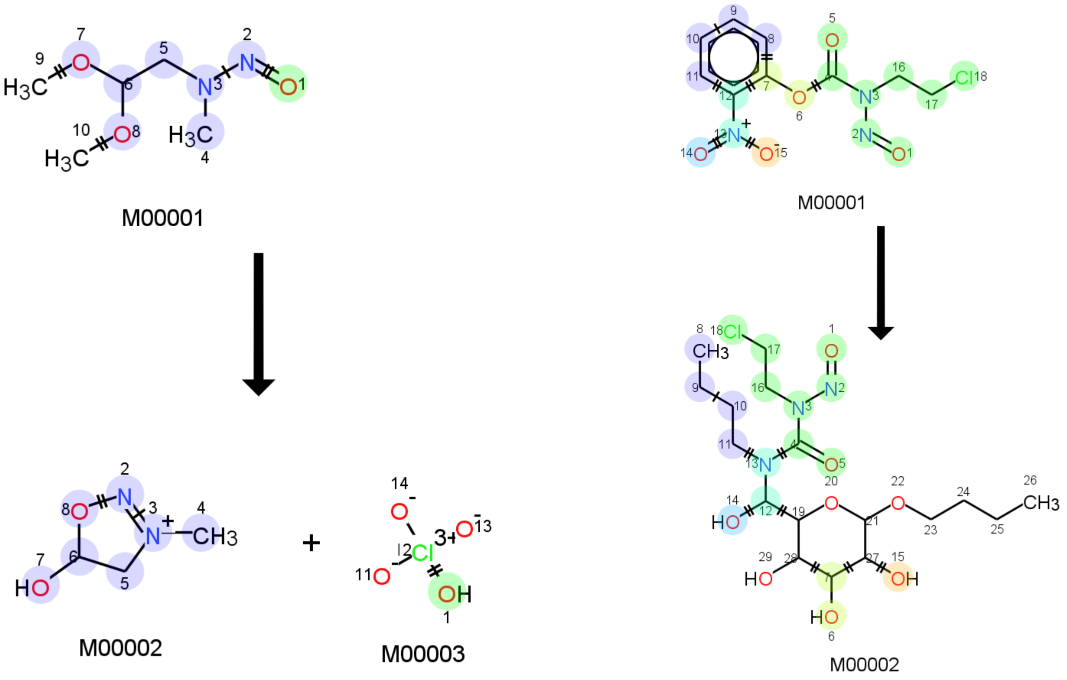

Figure S2: Reactions from the set of reactions that failed to generate reactionCodes which have unbalanced stoichiometry.

Reactions which produce salts, like the leftmost reaction in Figure S2 often did not include the starting acid on the reactant side of the reaction SMILES string. Furthermore, it was often the case that the reactants were actually buried within the reagents, with the reactants side of the reaction SMILES only containing the starting nitrosamine. Several factors, including those mentioned, lead to incorrect in AAM. These inaccuracies subsequently result in the failure of reactionCode generation due to incorrect bond formation/breakage and atom count in the condensed graph, which reactionCode heavily relies on. Furthermore, we highlight one specical case of incorrect reaction SMILES, where a reactant molecule gained one atom e.g. a nitroso to nitro oxidation or aromatic substitution reactions (hydrogens implicit) (Figure S3).

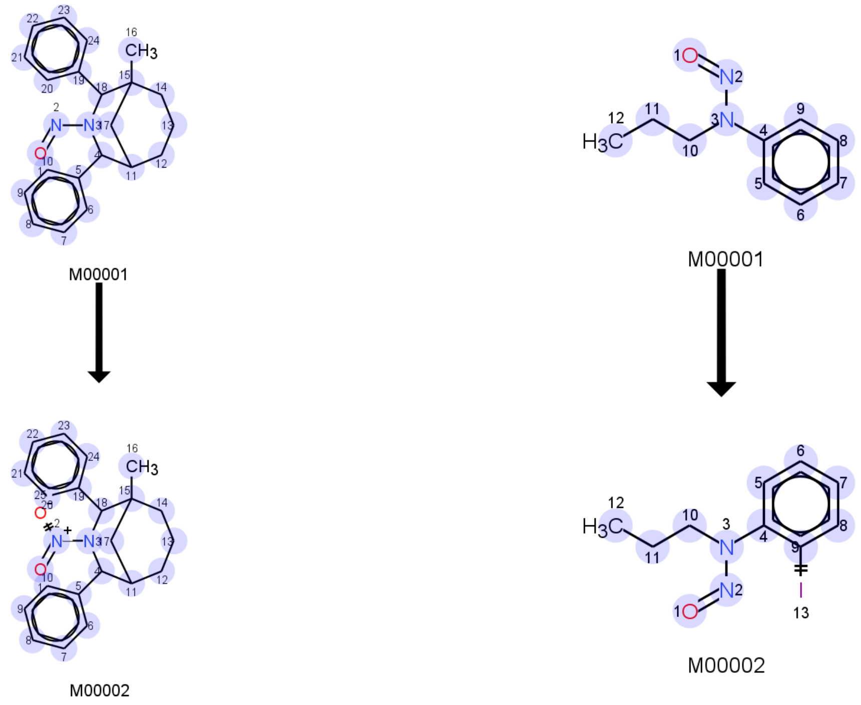

Figure S3: Reactions from the set of reactions that failed to generate reactionCodes which have product atoms which are buried in reagents.

This failure case indicated that examples of nitroso to nitro oxidation may have been missed by our analysis. A search of the reagents column for the set of reactions which failed to generate reactionCodes yielded the oxidation conditions listed in Table S1. Analysis of the reaction conditions indicated that the transformations that were missed as a result of this was oxidation of nitrosamines to N-nitro compounds by hydrogen peroxide/water (ca. 90%), and combinations of this with acetic anhydride and trifluoroacetic anhydride, leading to the peroxyacid oxidation described in the review by Borths *et al.*.[3–5] Further, more complicated reaction conditions included electrochemical oxidations of N-nitrosamines to N-nitramines using air in non-degassed acetonitrile as the oxidant, and highly specific examples of photooxidation of to N-nitramines in the presence of air, oxygen and nitrogen dioxide.[6,7]

Table S1: Missed oxidation reaction conditions by ReactionCodes

**Reagent**	**Count**	**Notes**
air, oxygen, Nitrogen dioxide[7]	19	Photochemical oxidation with nitrogen dioxide, *N*-nitroso to *N*-nitro, multiple side products and tars
dihydrogen peroxide[8]	16	*N*-nitroso to *N*-nitro with 90% H_2O_2
oxygen[6]	15	Electrochemical oxidation, *N*-nitroso to *N*-nitro
oxygen, tetraethylammonium perchlorate[6]	10	Electrochemical oxidation, *N*-nitroso to *N*-nitro
methanol, air, Nitrogen dioxide[7]	8	Photochemical oxidation with nitrogen dioxide, *N*-nitroso to *N*-nitro, multiple side products and tars
sodium periodate[9]	8	S to S=O, no reaction with nitroso
bromine, iodine, acetic acid[10]	8	Reagents not applied to nitrosamine
trifluoroacetyl peroxide[11]	7	*N*-nitroso to *N*-nitro, ca. 90% H_2O_2 and acetic anhydride
lithium hydroxide, dihydrogen peroxide[5]	7	Nitrosamine affected by LiOH - diazonium formation
oxygen, Nitrogen dioxide[7]	7	Photochemical oxidation with nitrogen dioxide, *N*-nitroso to *N*-nitro, multiple side products and tars
3-chloro-benzenecarboperoxoic acid[12]	7	S to S=O, no reaction with nitroso
air[7]	7	Photochemical oxidation with air, *N*-nitroso to *N*-nitro
hydrogen, phosphorus tribromide, 3-chloro-benzenecarboperoxoic acid[12]	5	Multistep, *m*-CPBA illicits S to S=O
oxygen, *N,N,N,N*-tetraethylammonium tetrafluoroborate, sodium carbonate[6]	5	Electrochemical oxidation, *N*-Nitroso to *N*-nitro
sodium tert-butyl hydroperoxide[13]	4	Diazonium formation using (-)O-OR
potassium permanganate, acetic acid[9]	4	S to SO_2, no reaction with nitroso

1.4 Filtering irrelevant reactions and duplicates

A total of 5486 reactions successfully underwent the encoding process into ReactionCodes. The successfully encoded ReactionCodes were decoded into reaction SMARTS strings at the depth 0 and depth 1 levels to produce 2 sets of reaction centres with varying specificity. Upon analysis, it was found that the depth 0 reaction codes did not provide enough clarity as to whether the reaction occurred at the nitrosamine functional group or not (Figure

S4). Therefore, for the next processing step, the depth 1 Reaction SMARTS codes were considered.

N-nitroso substructure SMARTS: [#7]-[#7]=[#8]

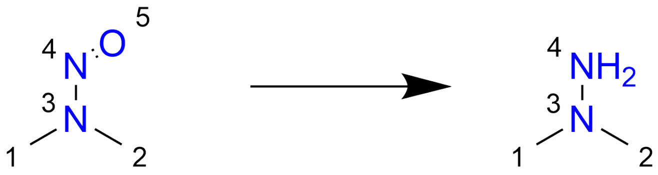

Depth 0 Core: [N]=[O]>>[N].[O]

Depth 1 Core: [N]-[N]=[O]>>[N]-[N].[O]

Depth 2 Core: [C][N]([C])-[N]=[O]>>[C][N]([C])-[N].[O]

Figure S4: Analysis of depth 0, 1 and 2 ReactionCodes to show that depth 1 codes are sufficient to identify reactions that consume *N*-nitrosamines.

With the appropriate sized reaction centre selected, the next step in the cheminformatics workflow was to filter out reactions that did not occur at the N−N=O substructure. The transformation to SMARTS strings enabled parsing the resulting reaction centre SMARTS strings in python. Using a combination of pybel and rdkit to loop through the atoms in the reactants, the atom map numbers of the N−N=O substructure in the reactants were identified, and these atoms were searched for in the products (Listing S3). If the atom map numbers of N−N=O were found in the product SMARTS of the reaction centre, this indicated that the N−N=O groups was part of the reaction centre, and was transformed in some way during the reaction. After these filering steps, 2327 reactions remained.

Listing S3: Python code to find reactions which consume nitrosamines

```
# Need to remove the reactions which have the nitrosamine as part of the leaving group
# i.e. need to make sure N-N=O are accounted for in the products.
# To do this, find atom map numbers of N-N=O and check if they
# exist in the product atoms
# Any reactions which no not meet this criteria will be discarded.

consumes_nitrosamine = []
# loop through the reactants and products of the depth 1 reaction cores
for reactants, products in zip(full_added["Depth_1_reactants"],
                               full_added["Depth_1_products"]):
    try:
       # Read molecule using Pybel
        reactant_mol = pybel.readstring("smi", reactants)
        # Convert the SMILES to RDKit mol format
        rdkit_reactant_mol = Chem.MolFromSmarts(reactants)
        # Get RDKit product molecules
        product_mols = Chem.MolFromSmarts(products)
        # Perform SMARTS matching on the reactants using Open Babel
        smarts = pybel.Smarts("[#7]-[#7]=[#8]") # SMARTS Pattern for N-N=O
        matching_atoms = smarts.findall(reactant_mol)
        # If there is a match for the N-N=O
        if matching_atoms != []:
            # Get the atom map numbers from the matched atoms using RDKit
            atom_map_nums = []
            for atom_indices in matching_atoms:
                for atom_index in atom_indices:
                    # RDKit atom indices start from 0
                    atom = rdkit_reactant_mol.GetAtomWithIdx(atom_index - 1)
                    atom_map_num = atom.GetAtomMapNum()
                    if atom_map_num != 0:
                        atom_map_nums.append(atom_map_num)
            # check if the nitrosamine atoms are present in the products
            if set(atom_map_nums).issubset([atom.GetAtomMapNum() for
                                            atom in product_mols.GetAtoms()]) == True:
                consumes_nitrosamine.append(True)
        else:
            consumes_nitrosamine.append(False)
    except:
        consumes_nitrosamine.append(False)
full_added["Consumes_Nitrosamine_depth_1"] = consumes_nitrosamine
```

Within these 2327 reactions, there were many duplicates. With a view to manually classifying the reactions, an attempt to standardise the reaction conditions was made in order to reduce the number of duplicate reactions. The Reaxys data was extremely inconsistent as to which columns solvents and reagents were assigned to, this made the number of unique values for the reaction conditions much higher than the true number of uniques due to the ordering of the reagents. To combat this, a list of common solvents was constructed, and solvents in this list were removed if they were present in the reagents column, and added to the solvents column (Listing S4).

Listing S4: Cleaning up the reagents column by removing common solvents.

```
# PREPARE DATA FOR MANUAL CLASSIFICATION

# Define a list of common solvents to remove from All Reagents column
common_solvents = ["1,4-dioxane", "water", "methanol", "DMSO", "dichloromethane",
↪ "dichloromethane-d2", "tetrahydrofuran", "benzene", "tetrachloromethane", "diethyl
↪ ether", "ethanol", "xylene", "petroleum ether", "Acetone", "chloroform",
↪ "n-heptane", "acetonitrile", "chloroform-d1", "neat (no solvent)",
↪ "1,2-dichloro-ethane", "toluene", "n-heptane", "N,N-dimethyl-formamide",
↪ "cyclohexane", "ethoxyethoxyethanol", "butan-1-ol", "2-methoxy-ethanol", "pentane",
↪ "Petroleum ether", "1,1-dichloroethane"]

# Extract the reagents column from the pre-parsed dataset
allreagents = []
allsolvents = []
for i in nitrosamines_consumed['All Reagents']:
    reagents = i.split(', ')
    solvents = []
    # Remove solvents from the reagents
    for reagent in reagents[:]:
        if reagent in common_solvents:
            reagents.remove(reagent)
            solvents.append(reagent)
    allreagents.append(reagents)
    allsolvents.append(solvents)

```

With the new list of solvents and reagents in hand, the set function in python was applied

to each item in the new solvents and reagents list to reorder the items in the sub lists in a consistent manner. The ordered reagents and solvents lists were transformed into strings with the join function in python. To find unique entries, the homogenised reagents and depth 0 transformation codes were considered. Duplicates were dropped based on these two columns leaving 1080 reactions which were then manually classified (Listing S5).

Listing S5: Removing duplicate entries based on homogenised reagents and depth 0 reaction core.

```

# We need to homogenise the list of allreagents so that all similar sublists are
↪ identical.
# This is done by reading in each sublist and reorder the terms with the function set().
# Then we dump the reordered sublist into a new list.
allreagents_homogenised = []
allsolvents_homogenised = []
for sublist1, sublist2 in zip(allreagents,allsolvents):
    item1 = set(sublist1)
    item2 = set(sublist2)
    allreagents_homogenised.append(item1)
    allsolvents_homogenised.append(item2)

# Now we combine each sublist into a string and recreate the reagent list as a simple
↪ list of strings
allreagents_list = []
allsolvents_list = []
separator = ', '
for sublist1, sublist2 in zip(allreagents_homogenised,allsolvents_homogenised):
    item1 = separator.join(sublist1)
    item2 = separator.join(sublist2)
    allreagents_list.append(item1)
    allsolvents_list.append(item2)
# Now we can add these as a new columns in our dataframe "Homogenised Reagents" and
↪ "Homogenised Solvents"
nitrosamines_consumed["Homogenised Reagents"] = allreagents_list
nitrosamines_consumed["Homogenised Solvents"] = allsolvents_list

# Add a cat code column to make it easier to filter in excel
nitrosamines_consumed['Reagents_Cat_Codes'] = nitrosamines_consumed['Homogenised
↪ Reagents'].astype("category").cat.codes
nitrosamines_consumed['Depth_0_Cat_Codes_unmapped'] =
↪ nitrosamines_consumed['Depth_0_Core_unmapped'].astype("category").cat.codes

# Make a new code which combines the unique reagent combinations and the unique depth 1
↪ transformations
nitrosamines_consumed["Reagent_Transformation_Code"] =
↪ nitrosamines_consumed["Reagents_Cat_Codes"].astype(str) +
↪ nitrosamines_consumed["Depth_0_Cat_Codes_unmapped"].astype(str)

nitrosamines_consumed.to_csv("nitrosamine_consumed_reagents_homogenised.csv")
```

2 Literature reaction data analysis

2.1 Initial classification

The classification was carried out on 3 defining features of the reactions: the structural class of N-nitrosamine in the reaction, the type of transformation taking place, and the reaction conditions. In some cases, the reactions and reference simply did not have enough information to determine all of the 3 sub-classes required for complete classification, this was true for 190 reactions. In total, 890 reactions were fully classified. Attempts to retrieve pseudo reaction kinetics information from temperature, reaction time and yields/conversions were halted by the low number of reactions with all these information (Figure S5).

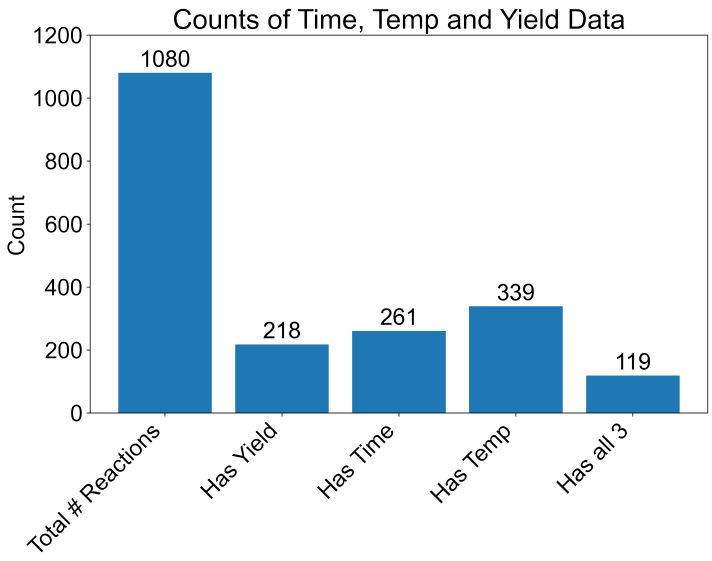

Figure S5: Bar chart to show the lack of pseudo-kinetic data in the curated dataset of N-nitrosamine consuming reactions.

Where Reaxys data were insufficient, the original publications was referred to, this exemplifies the need for manual classification over automated approaches. Furthermore, while it was often the case that the product atoms were represented by the reactant atoms, only ca. 10% of reactions in the dataset were stoichiometrically balanced (Figure S6). This re-

stricts the use of automated reaction classification by reaction centres due to the predicted uncertainty in the authenticity of the computed reaction centres.

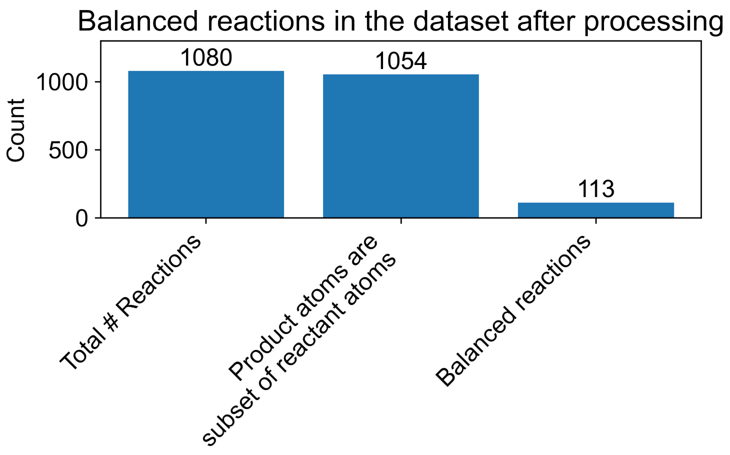

Figure S6: Number of balanced reactions in the dataset.

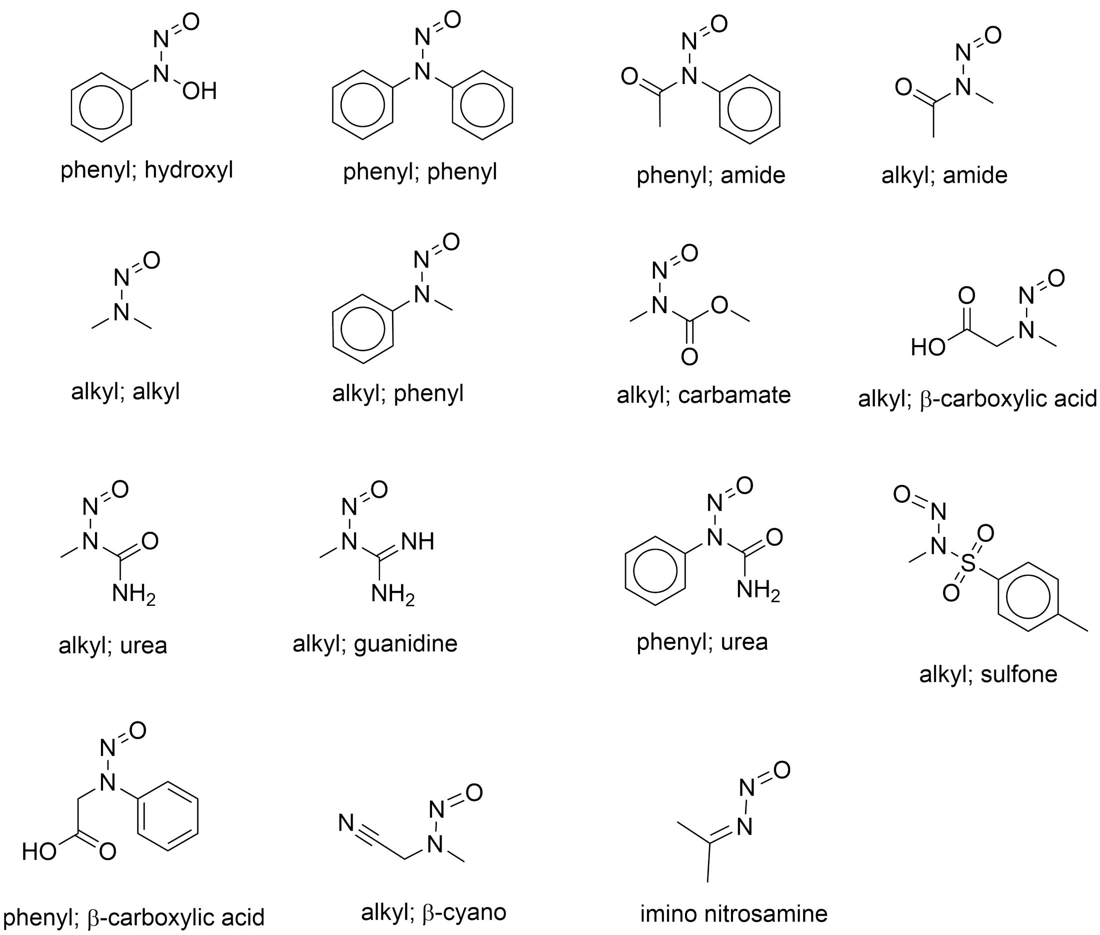

Scheme S1: Structural examples of nitrosamine classes.

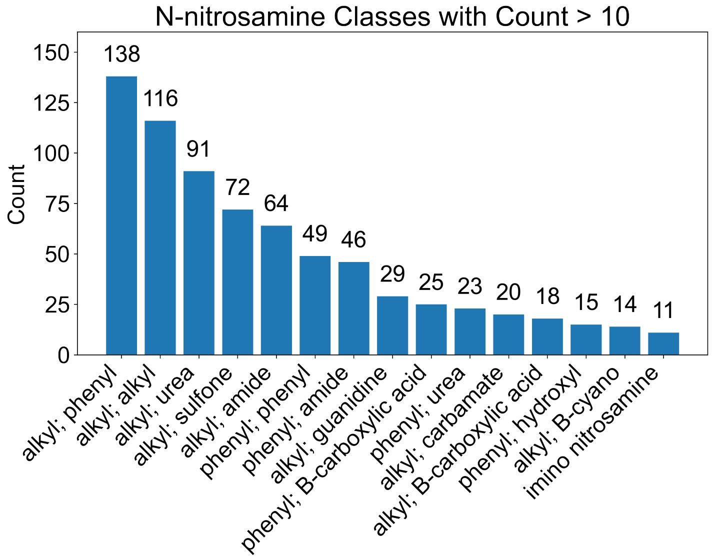

.

Figure S7: Counts of the nitrosamines in Scheme S1.

2.2 Re-classification based on the analysis of literature reaction data

The classification workflow is detailed in Figure S11. Initially, nitrosamine classes were assigned based on the R-groups adjacent to the N−N=O substructure common to all *N*-nitrosamines. Using this method, it was evident that there were a number of classes which were poorly represented (less than 10 examples), and so to ensure confidence in the reactivity examples described in this work, these classes were removed, leaving 731 reactions. Example structures and the counts of the nitrosamine classes with count above 10 in the 731 fully classified reactions are provided in Scheme S1 and Figure S7. These were plotted against the 10 transformation classes and the 21 reagent classes from Figure S12. Heatmap plots are shown in Figures S8 and S9.

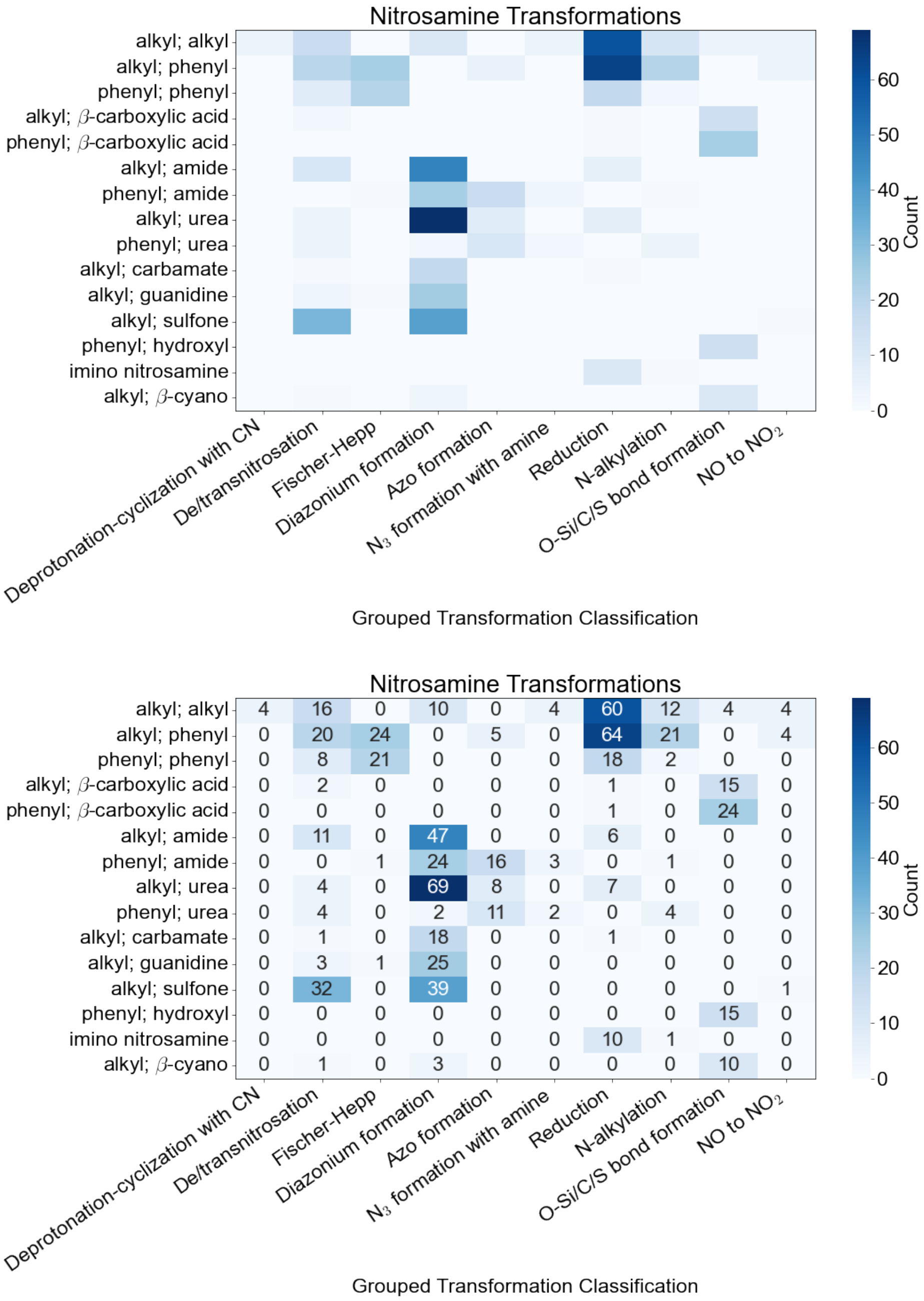

Figure S8: Initial nitrosamine classes and their transformations.

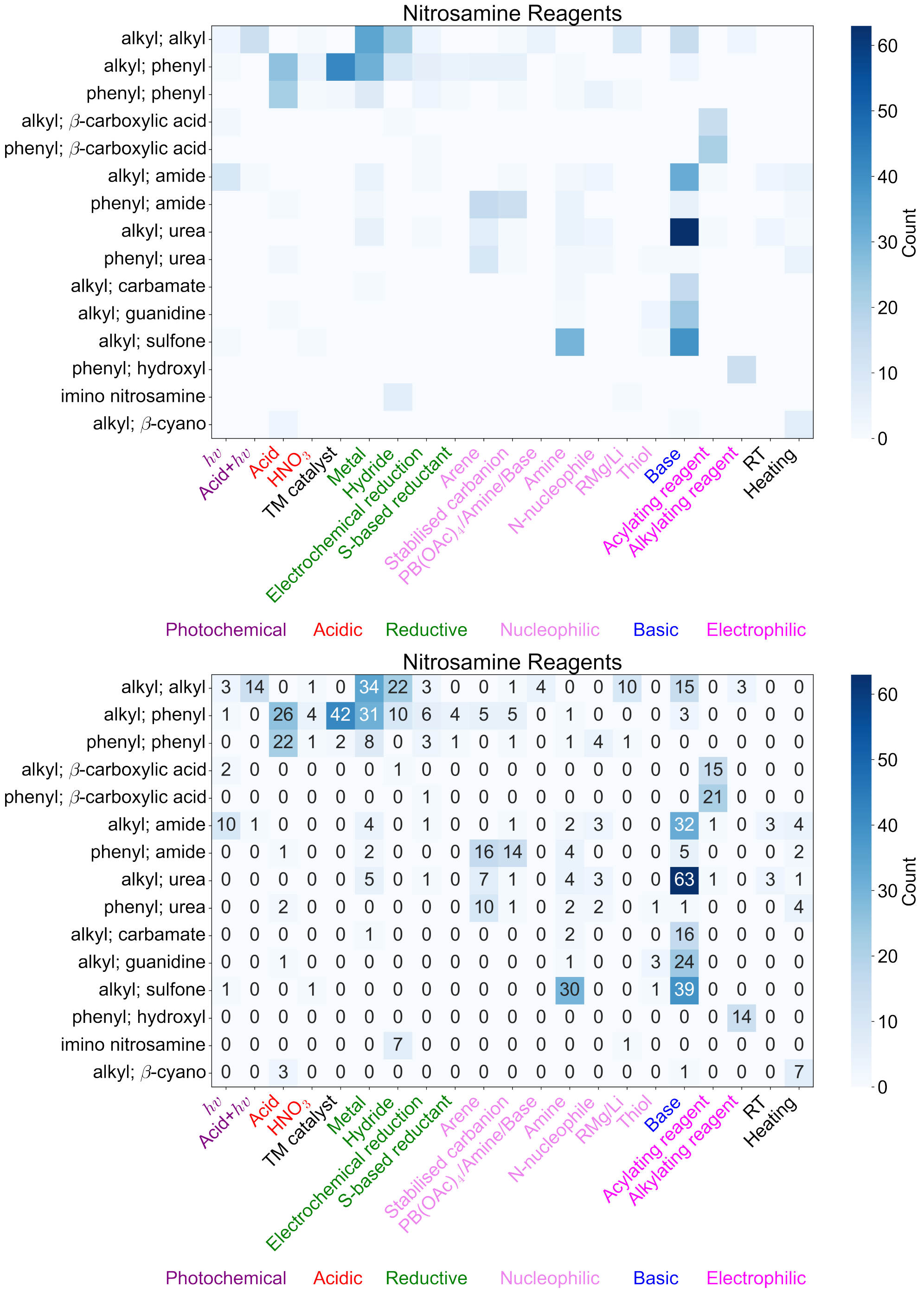

Figure S9: Initial nitrosamine classes and the applicable reagents.

There were clear similarities between the structures in Scheme S1, which allowed for further grouping of the classes by nitrosamines with shared reactive sites. This reduced the number of nitrosamine classes down to 8, which are displayed in Scheme S2.

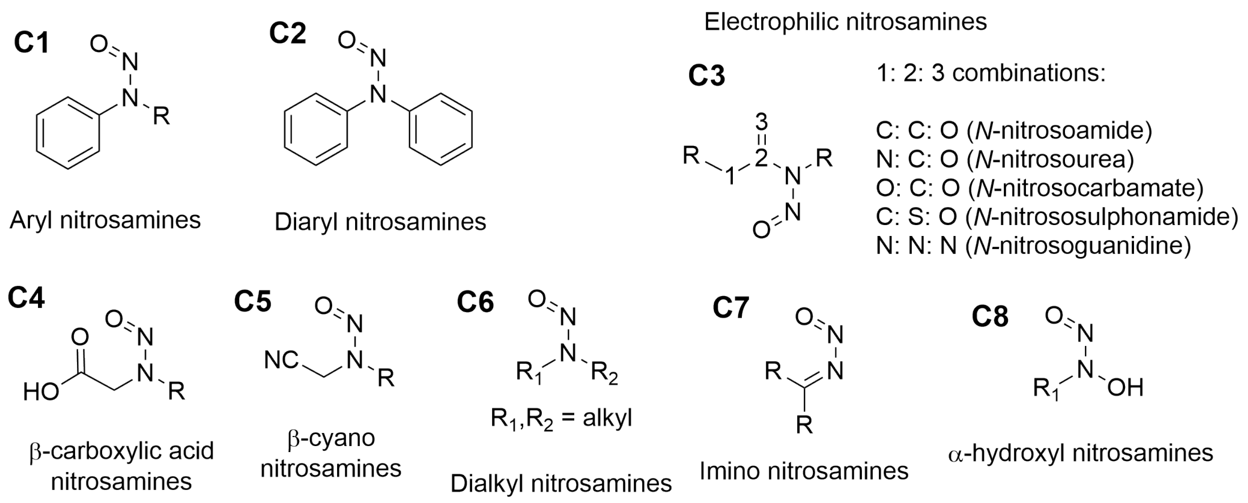

Scheme S2: The 8 nitrosamine classes grouped by reactive sites.

Classification of the reagents and transformation in each of the 731 reactions yielded 13 transformation classes and 32 reagent classes. There were many grouped nitrosamine: reagent: tansformation combinations with poor representation in the dataset (less than 3 examples). The examples with less than 3 count were removed, leaving 639 reactions with 10 transformation classes and 21 reagent classes. The counts of each grouped nitrosamine class in the reamining 639 reactions are displayed in Figure S10. Reactions on diaryl nitrosamines with lower than 3 count were included here to show the similarity in reactivities between classes C1 and C2.

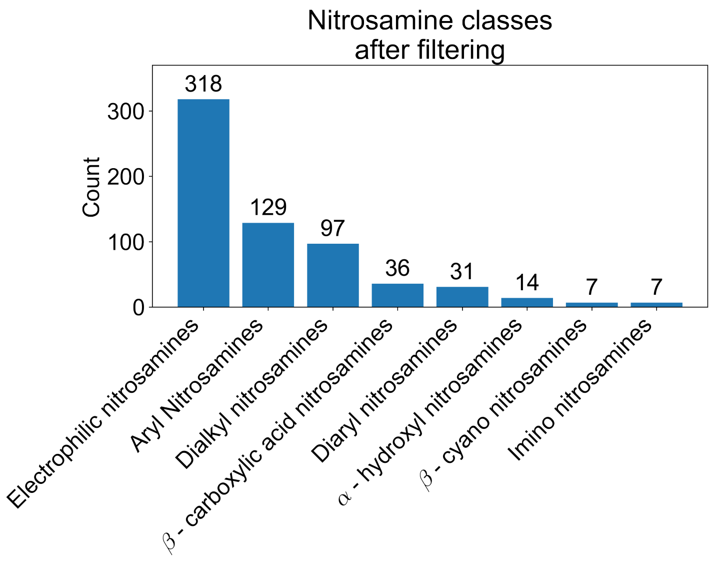

Figure S10: Counts of the nitrosamines in Scheme S2 after filtering.

The classification workflow summary is shown in Figure S11.

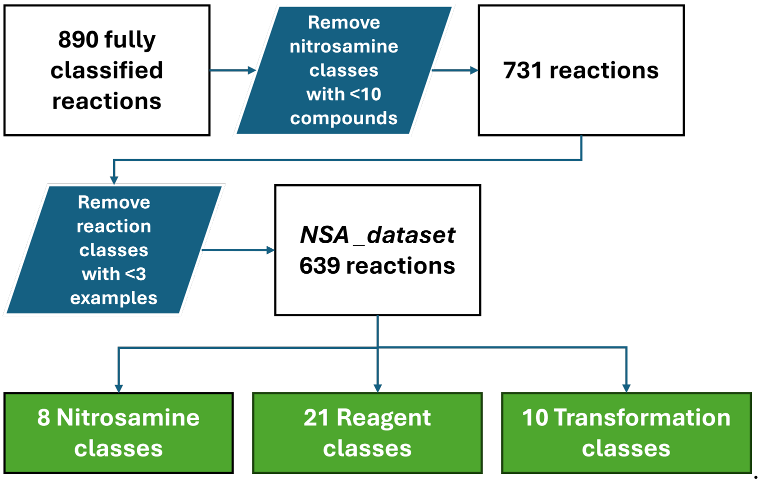

Figure S11: Workflow for classification and streamlining of the dataset.

Figure S12 summarises the reactivity of the grouped classes of N-nitrosamines. reactions highlighted in red are present in the two previous reviews on N-nitrosamine reactivity.[3,14]

Transformation	Reagent	Nitrosamine class							
		C1	C2	C3	C4	C5	C6	C7	C8
De/transnitrosation	Acid	*3*	*3*						
	Acid + *hv*						*13*		
	Amine			34					
	N-nucleophile		*4*						
	Stabilised carbanion	4							
	TM catalyst	9							
	Thiol			5					
	hv			11					
Deprotonation/cyclization with CN	Base						*4*		
Diazonium formation	Amine			5					
	Base			179			10		
	Heating			5					
	N-nucleophile			4					
	Stabilised carbanion			16					
	RT			6					
Fischer-Hepp reaction	Acid	*23*	*19*						
N-alkylation	Heating			5					
	RMg/Li						*10*		
	TM catalyst	20							
N_3 formation	Amine			5					
	$Pb(OAc)_4$/Amine/base						4		
Oxidation NO to NO_2	HNO_3	4							
O-Si/C/S bond formation	Acylating reagent				36				
	Alkylating reagent								14
	Heating					7			
Reduction	Electrochemical reduction	*6*	*3*						
	Hydride	*10*					*22*	7	
	Metal	*31*	*8*	10			*34*		
	S-based reductant	*4*	*1*						
	TM catalyst	*13*	*2*						
Diazo formation	Arene	5		33					

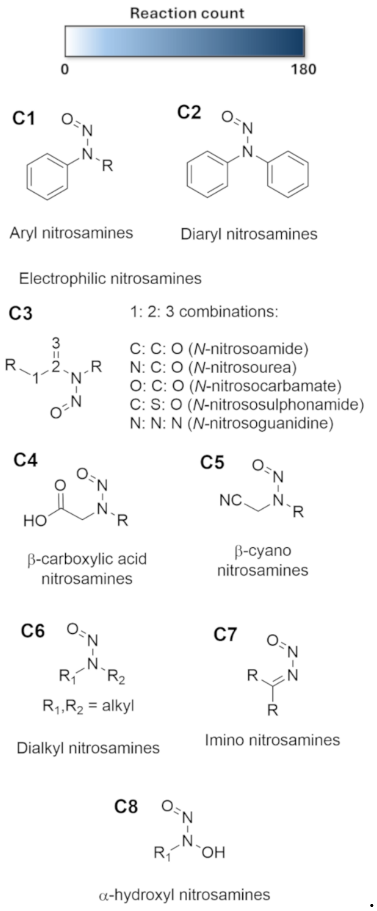

Figure S12: Summary of the classification in this work.

2.3 Heat maps for transformations and reagents of each class of *N*-nitrosamines

Each of the 15 initial nitrosamine classes was subjected to an iterative process which plotted the transformations and reagents applicable to that class. The plots include all 32 reagent classes and all 13 transformation classes. The plots are shown in Figures S13 to S27.

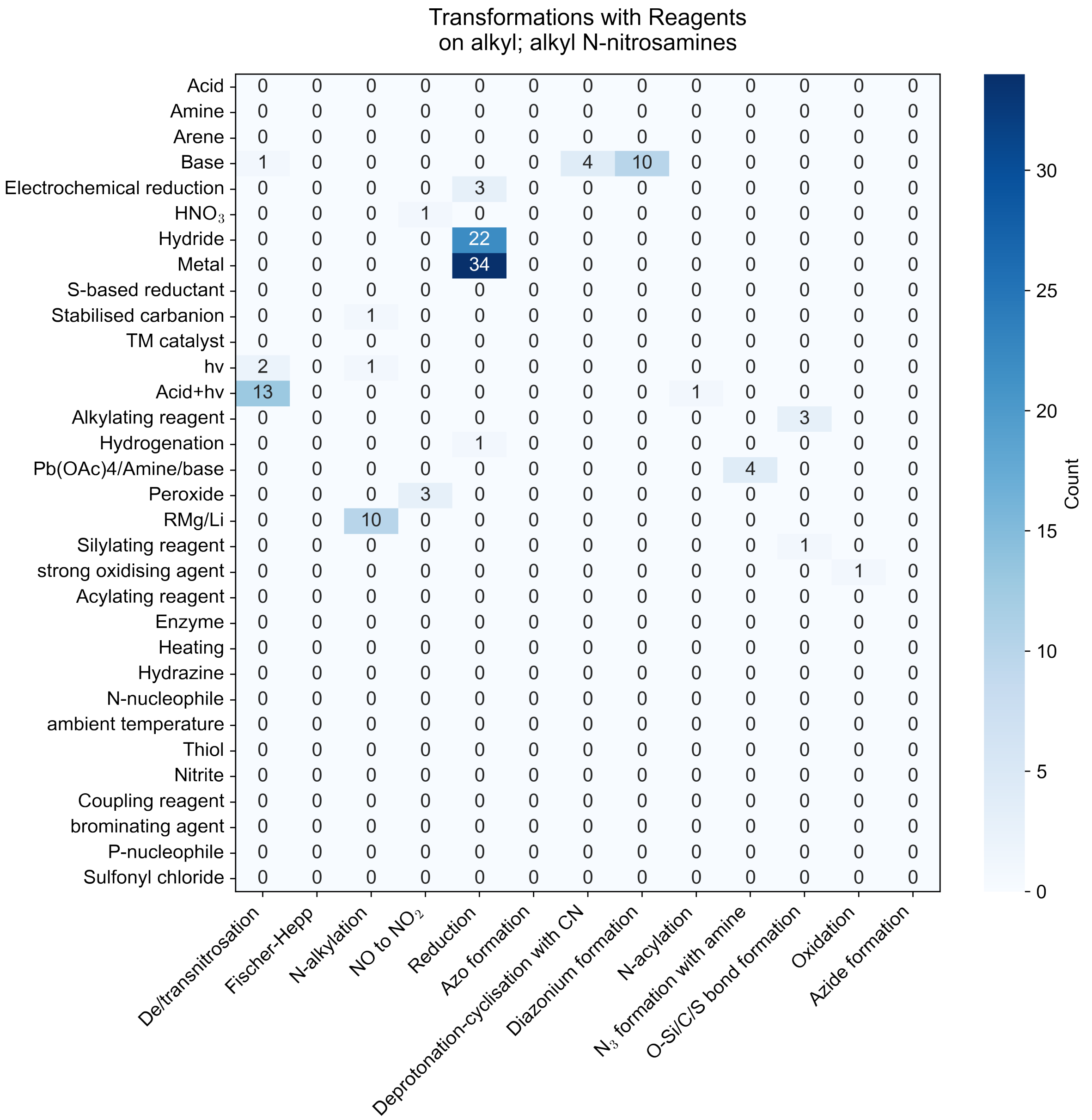

Figure S13: Literature transformations for alkyl;alkyl N-nitrosamines

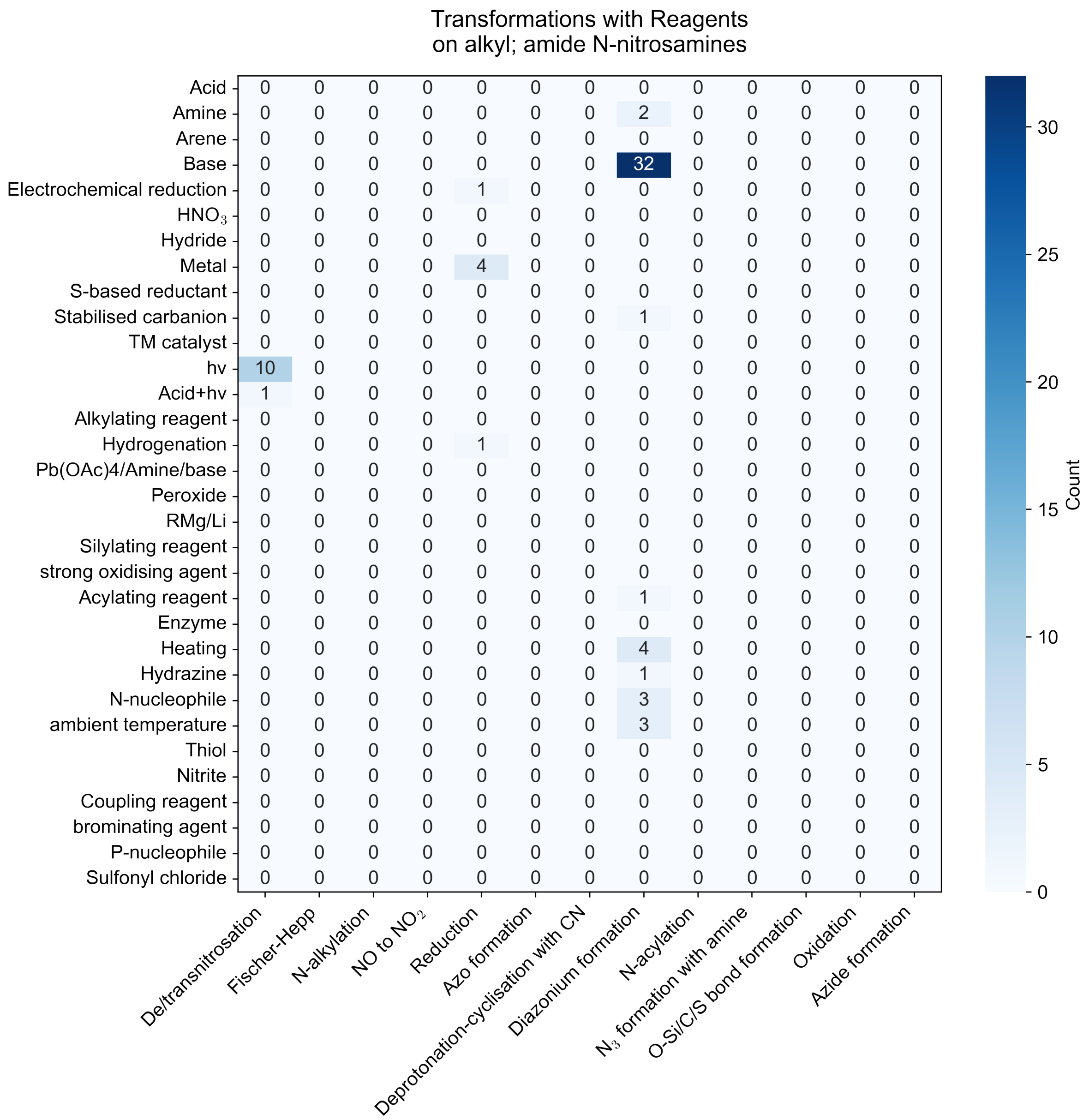

Figure S14: Literature transformations for alkyl;amide N-nitrosamines

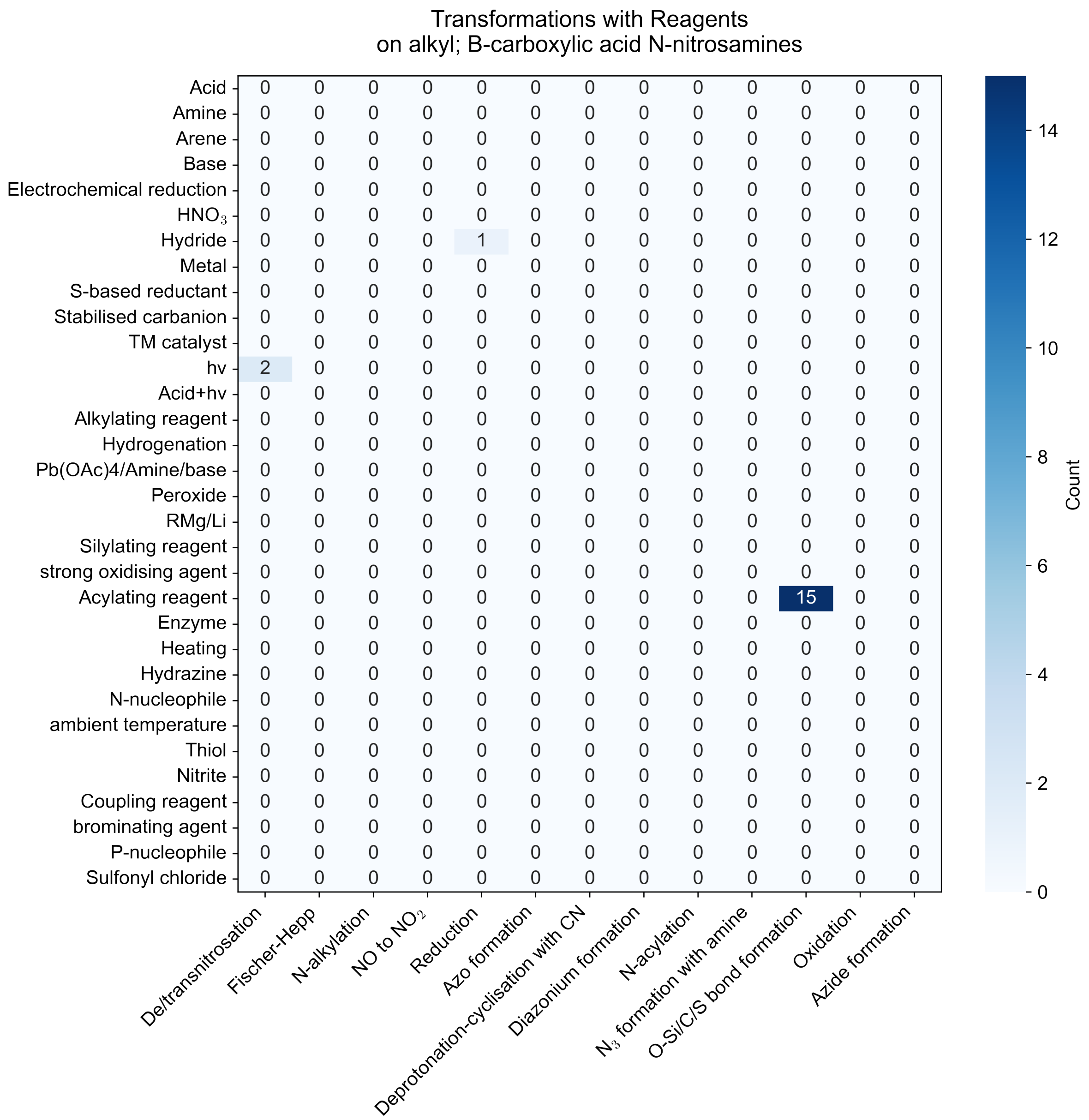

Figure S15: Literature transformations for alkyl;β-carboxylic acid N-nitrosamines

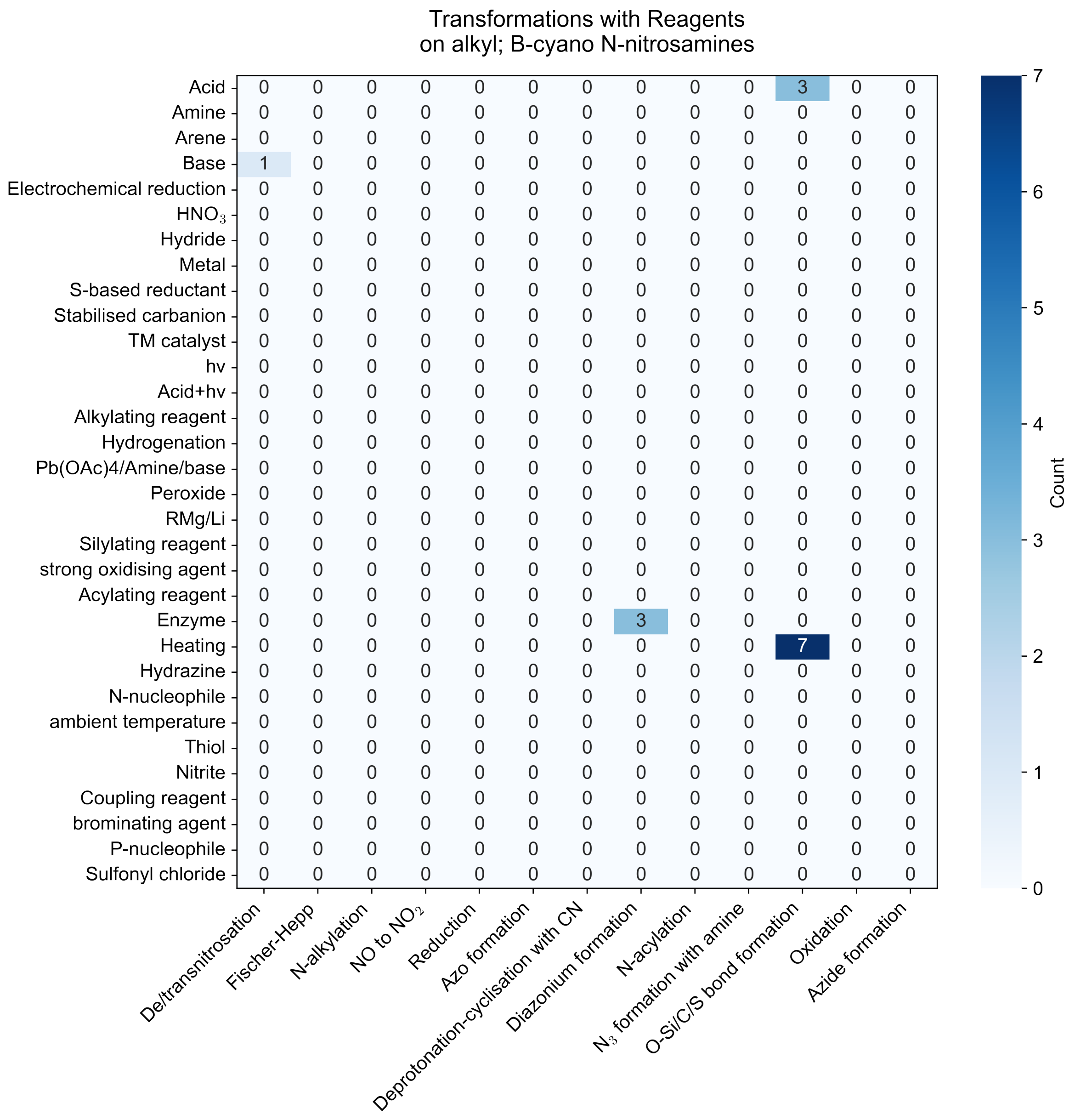

Figure S16: Literature transformations for alkyl;β-cyano N-nitrosamines

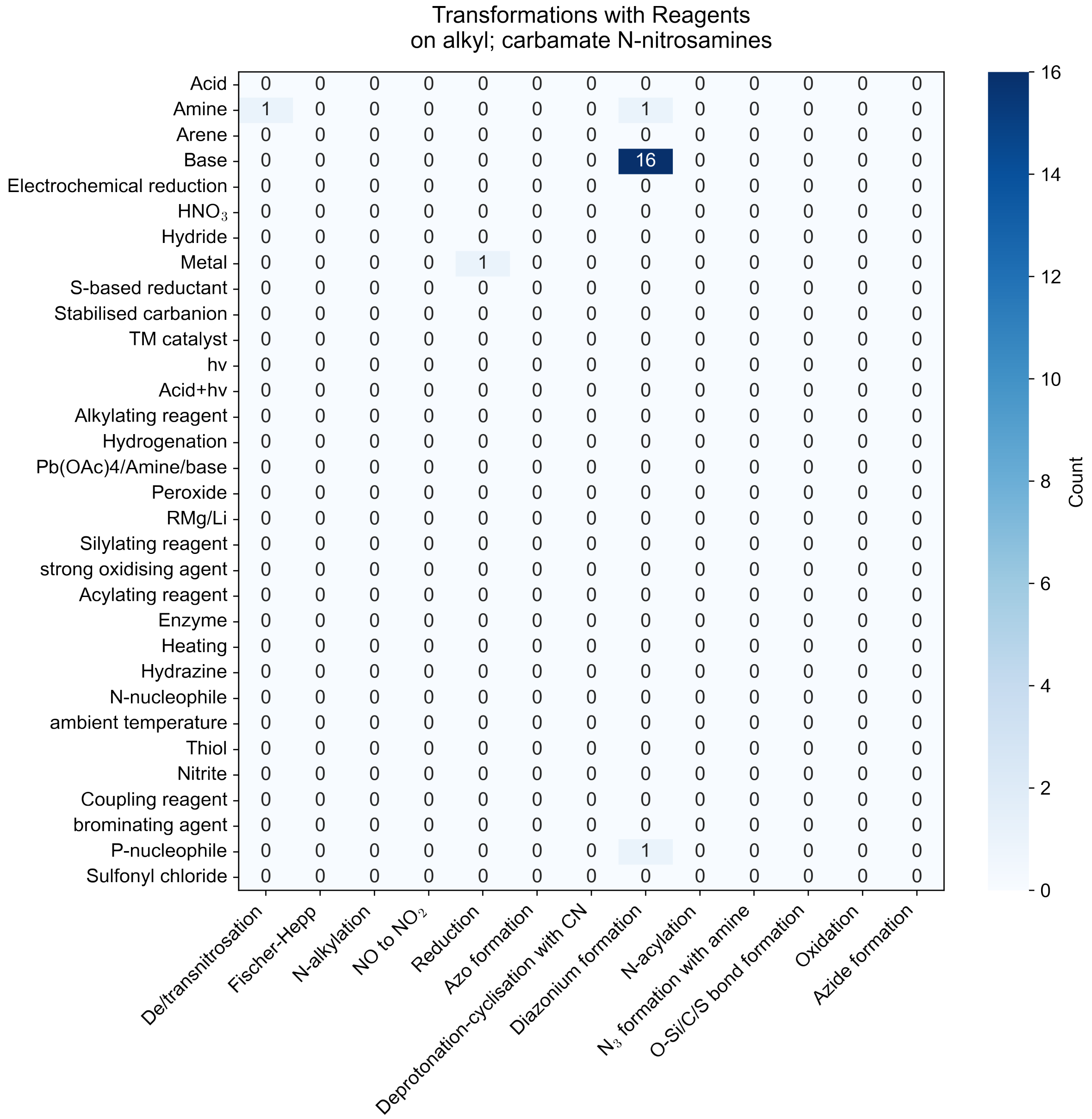

Figure S17: Literature transformations for alkyl;carbamate N-nitrosamines

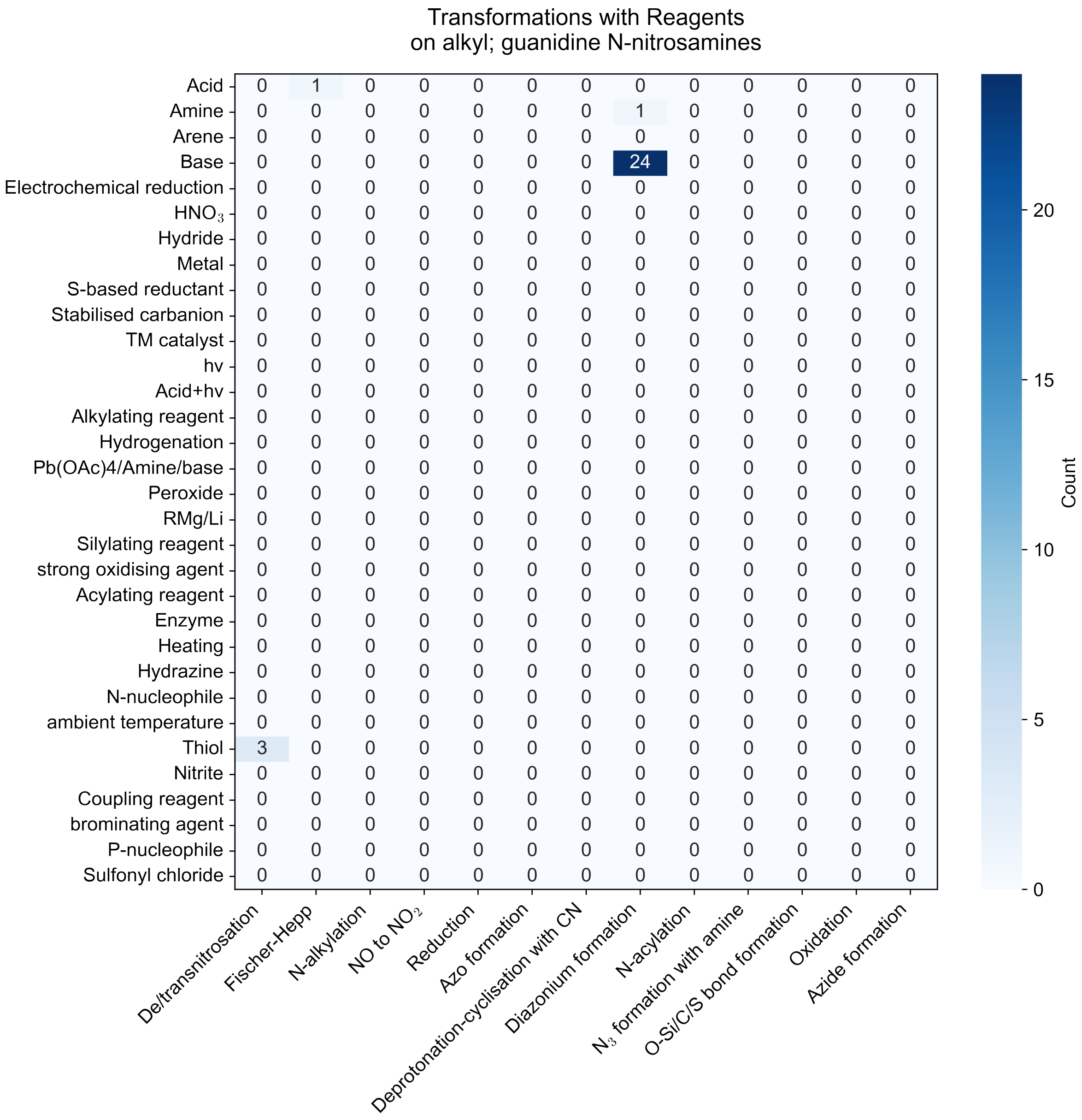

Figure S18: Literature transformations for alkyl;guanidine N-nitrosamines

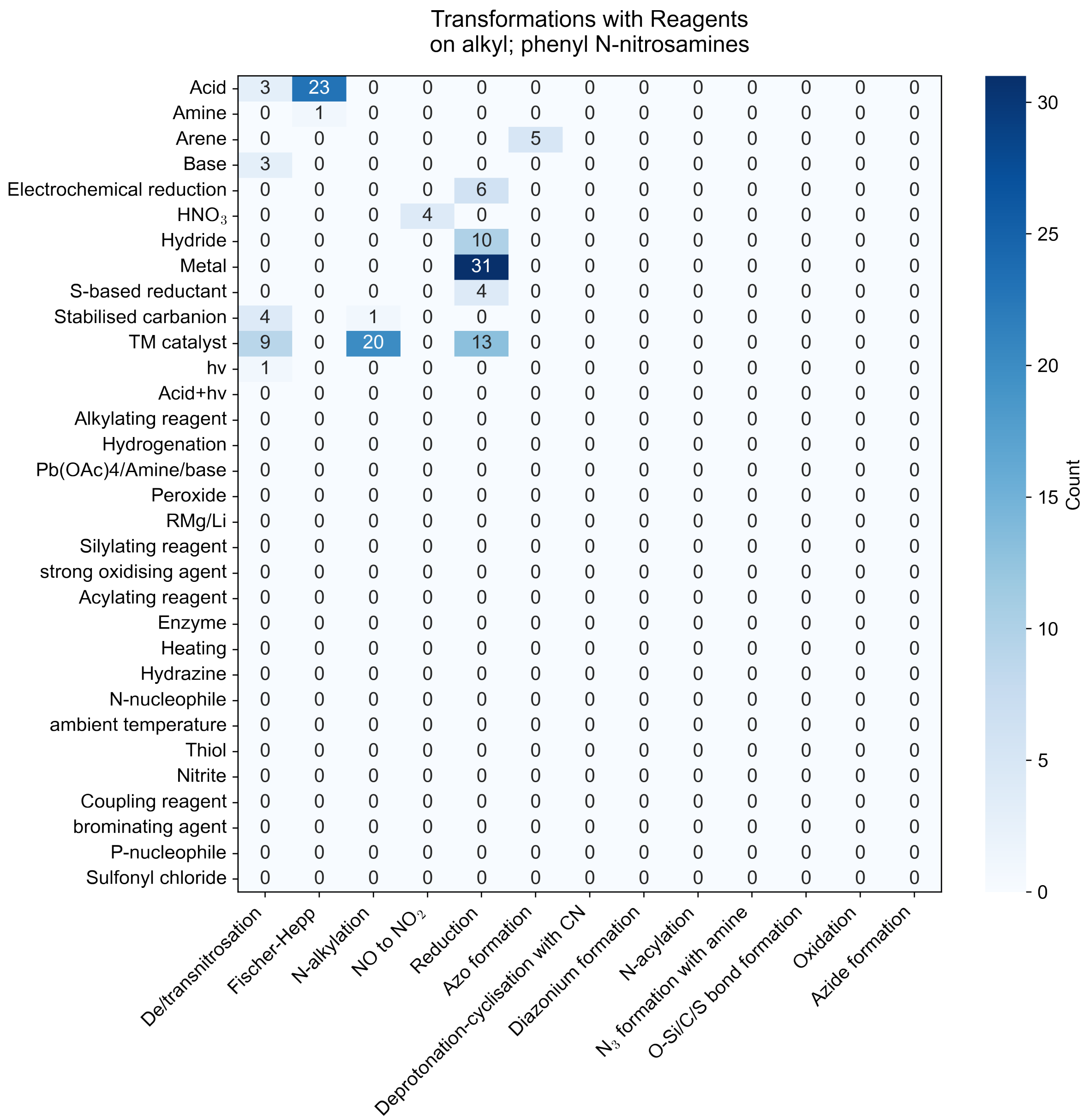

Figure S19: Literature transformations for alkyl;phenyl N-nitrosamines

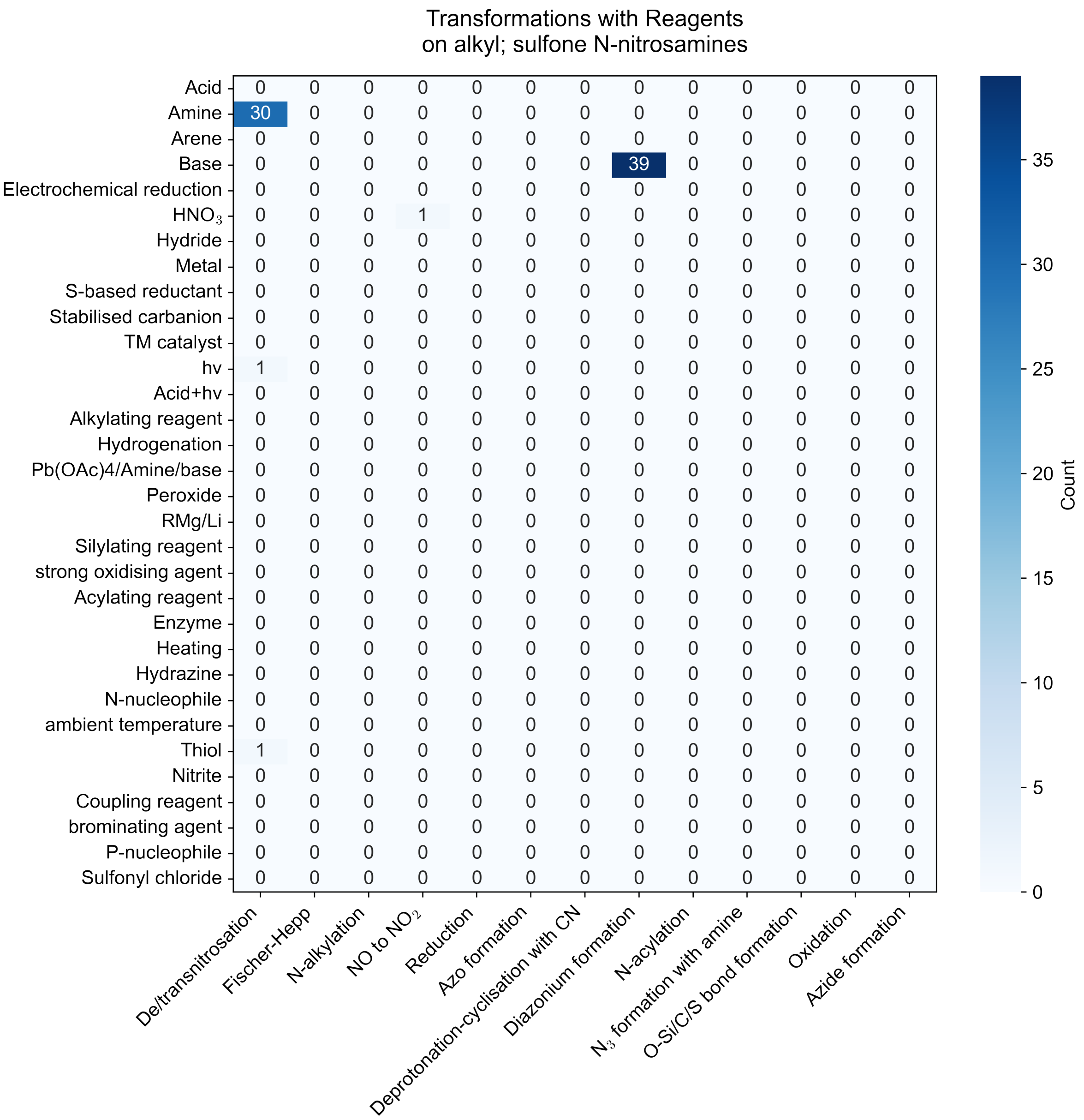

Figure S20: Literature transformations for alkyl;sulfone N-nitrosamines

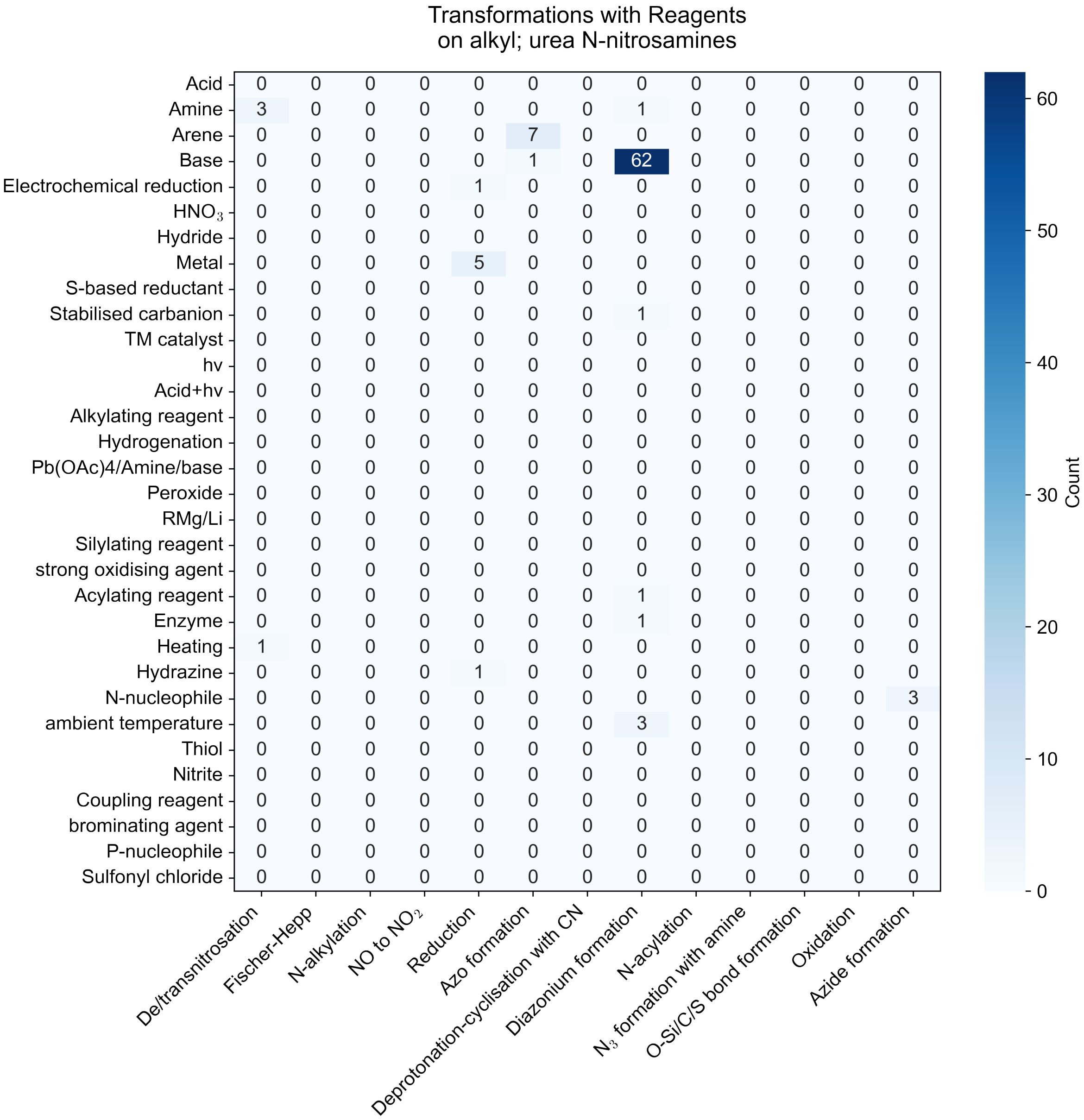

Figure S21: Literature transformations for alkyl;urea N-nitrosamines

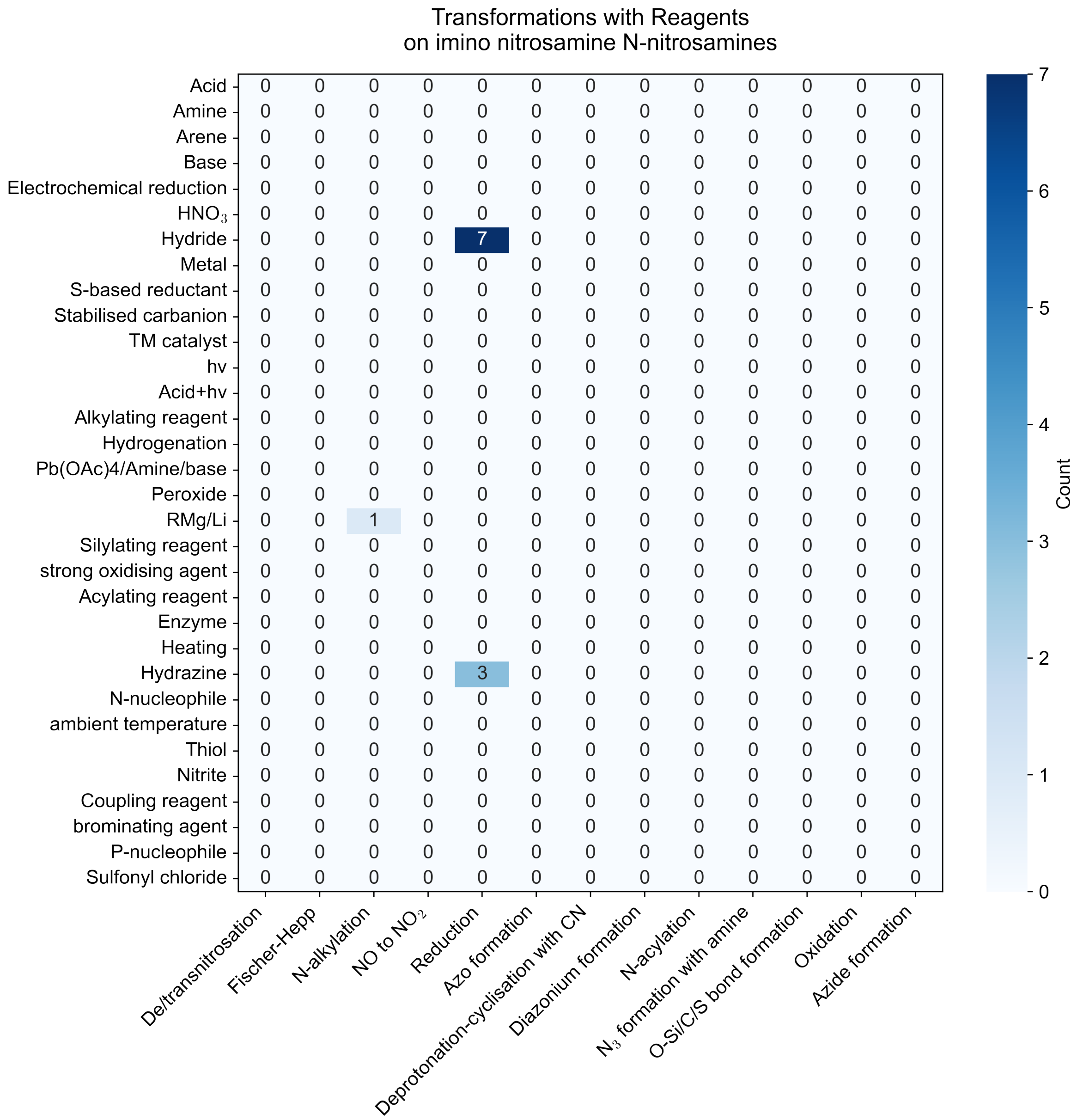

Figure S22: Literature transformations for imino N-nitrosamines

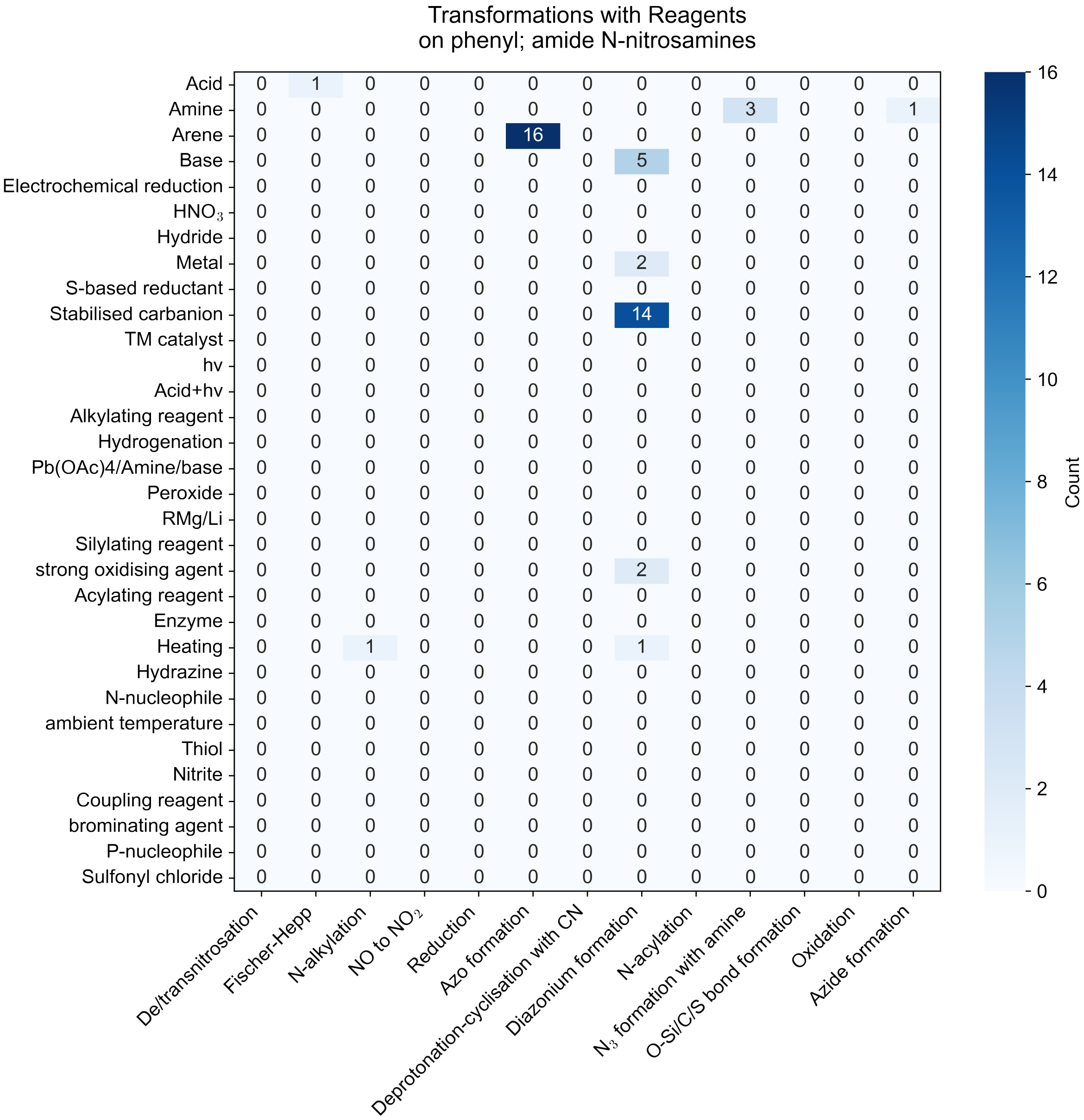

Figure S23: Literature transformations for phenyl;amide N-nitrosamines

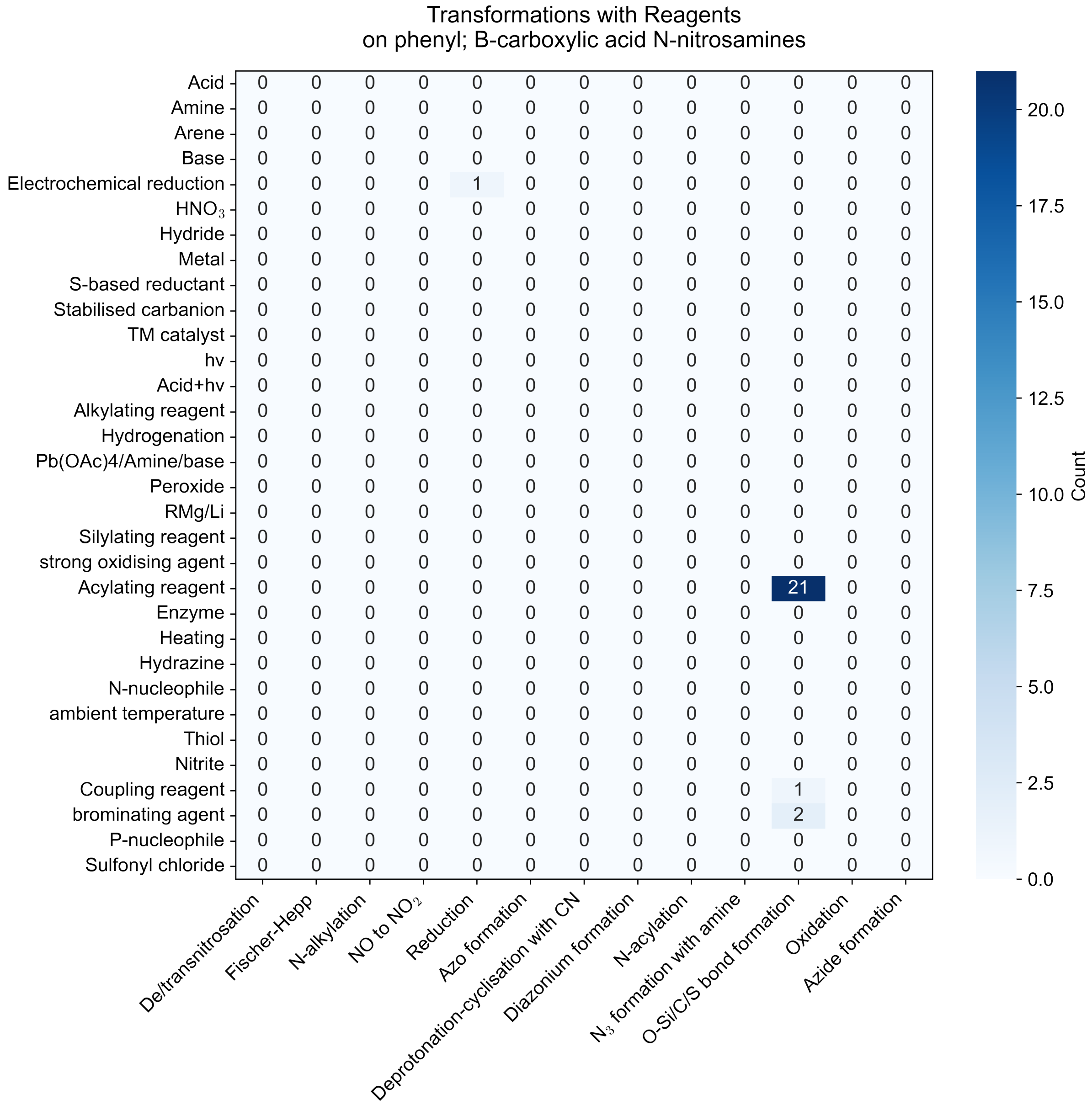

Figure S24: Literature transformations for phenyl;β-carboxylic acid N-nitrosamines

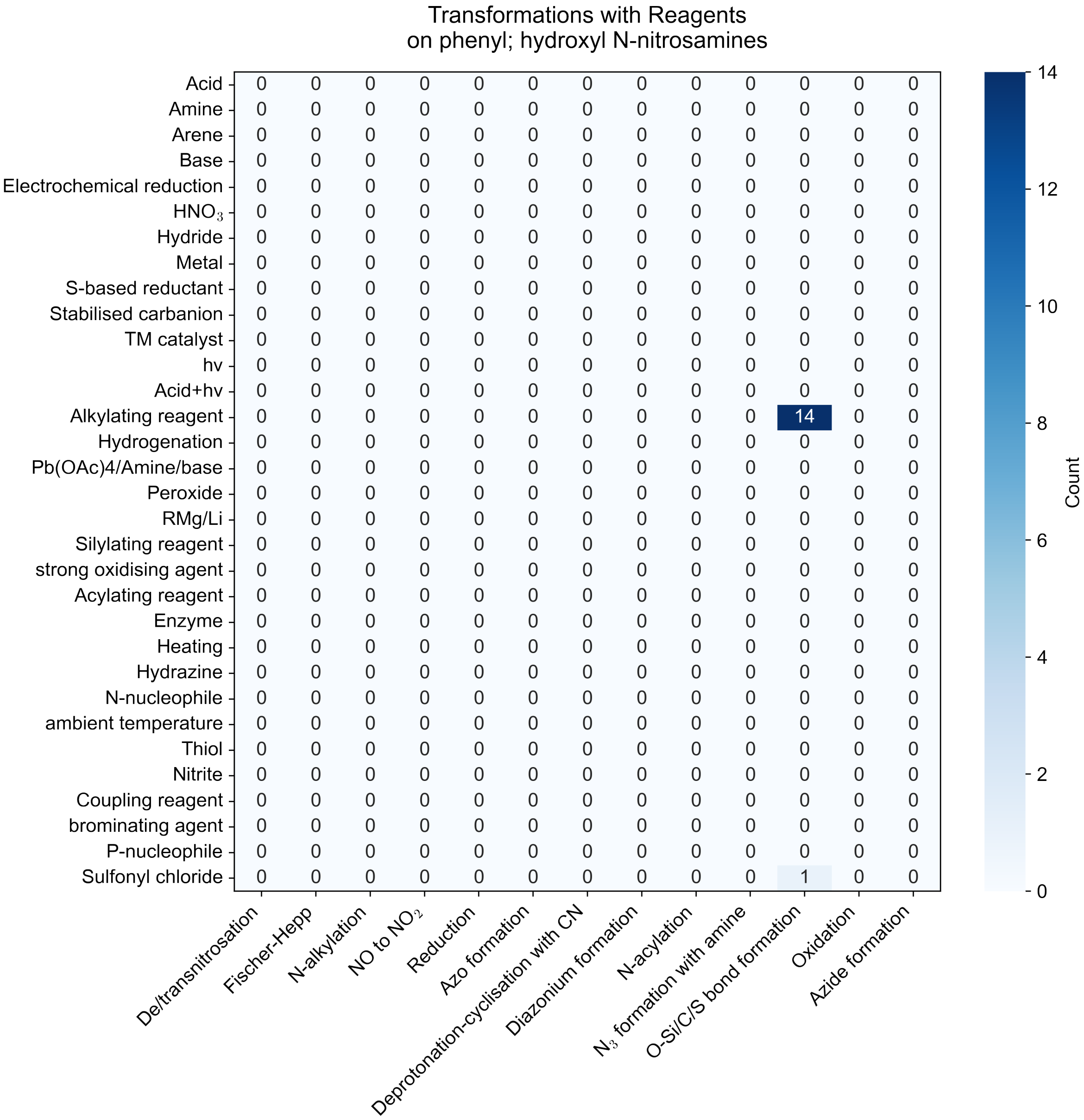

Figure S25: Literature transformations for phenyl;hydroxyl N-nitrosamines

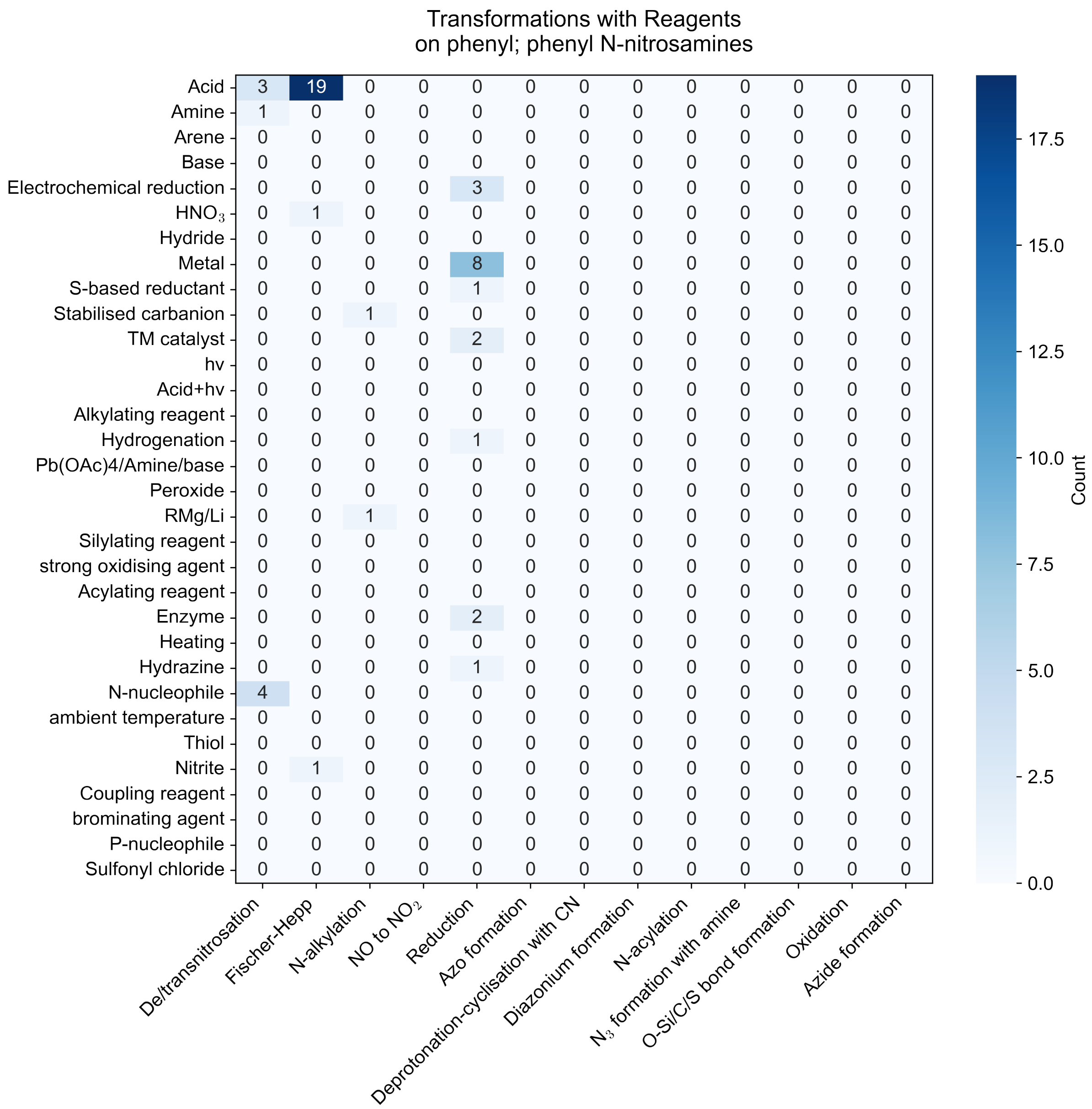

Figure S26: Literature transformations for phenyl;phenyl N-nitrosamines

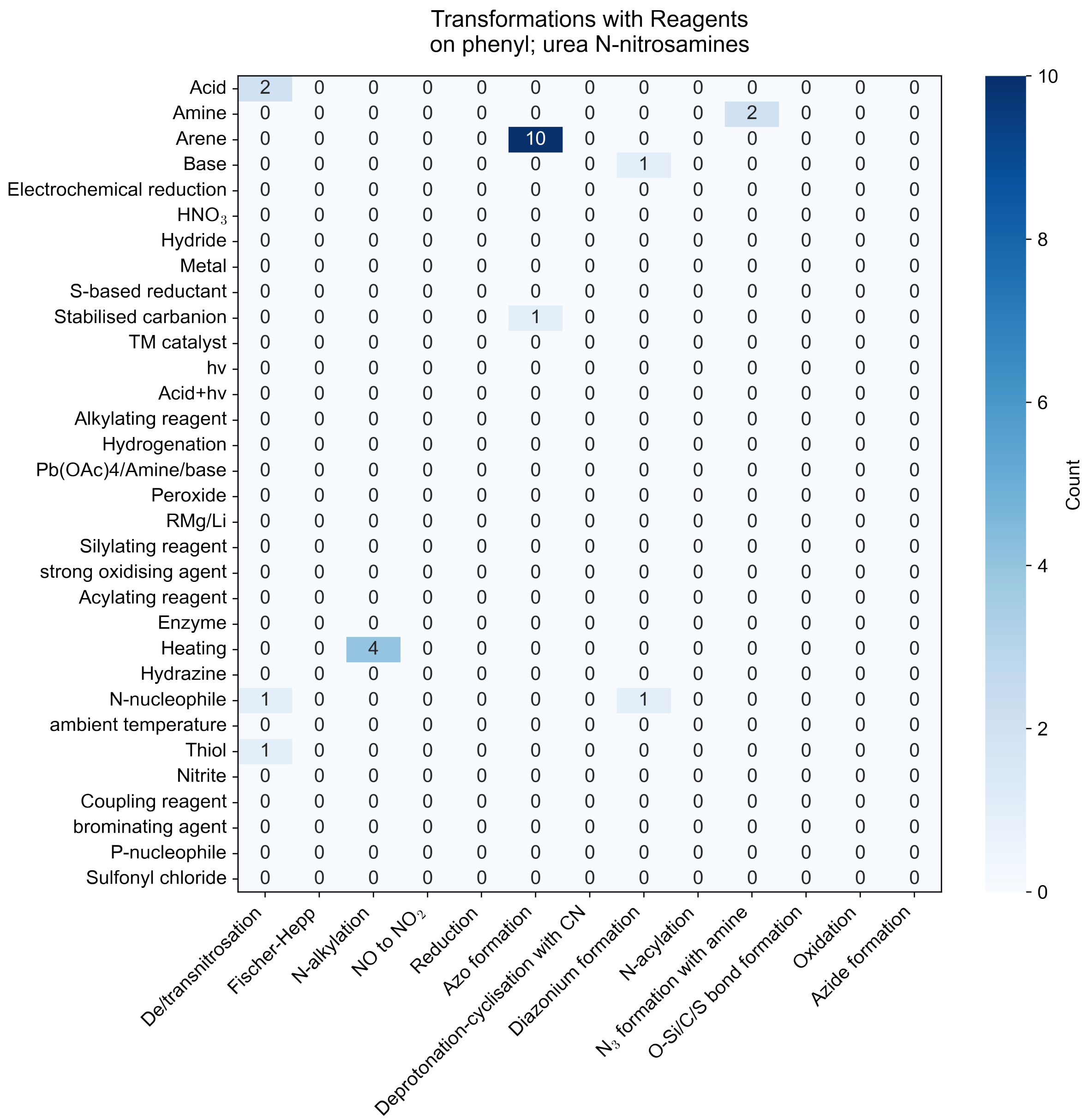

Figure S27: Literature transformations for phenyl;urea N-nitrosamines

2.4 Examples of organic transformations of *N*-nitrosamines

This work yielded a large number of unique nitrosamine: reagent: transformation class combinations, many of which were not present in the two previous reviews. To ensure trust in the validity of the reported examples, we report here the nitrosamine: reagent: transformation examples which have a count above 3. The examples have the reagents and

conditions as they were in the Reaxys database.

Reaction Example:
alkyl; B-carboxylic acid: O-Si/C/S bond formation: Acylating reagent
Reaxys ID: 184057
Reagents: acetic anhydride
Solvent: None
Time: None
Temperature: None
Condition: None

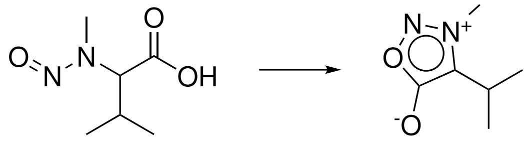

Reaction Example:
alkyl; B-cyano: O-Si/C/S bond formation: Heating
Reaxys ID: 2715288
Reagents: phenyl isocyanate, triethylamine
Solvent: benzene
Time: None
Temperature: 50
Condition: None

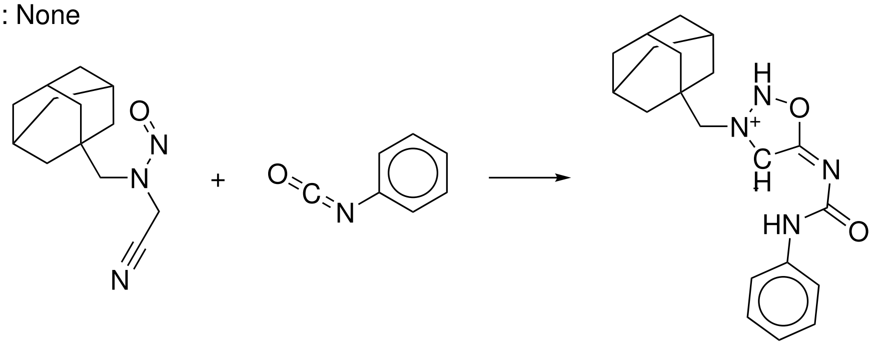

Reaction Example:
alkyl; alkyl: De/transnitrosation: Acid+hv
Reaxys ID: 917069
Reagents: 1,2,3,4,5,6,7,8-octahydro-naphthalene, hydrogenchloride
Solvent: methanol
Time: None
Temperature: None
Condition: Irradiation

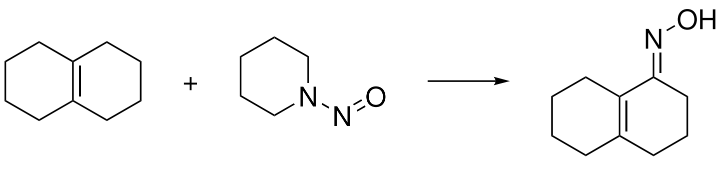

Scheme S3

Reaction Example:
alkyl; alkyl: Deprotonation-cyclisation with CN: Base
Reaxys ID: 886622
Reagents: 2-furancarbonitrile
Solvent: None
Time: None
Temperature: None
Condition: (i) LDA, THF, (ii) /BRN= 107033/

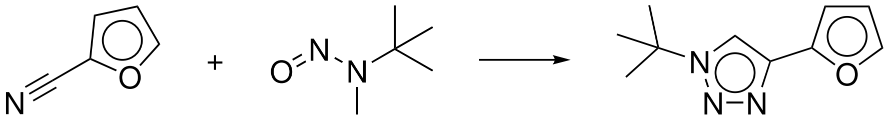

Reaction Example:
alkyl; alkyl: Diazonium formation: Base
Reaxys ID: 240906
Reagents:diethyl ether, sodium isopropylate, isopropyl alcohol
Solvent: None
Time: None
Temperature: None
Condition: None

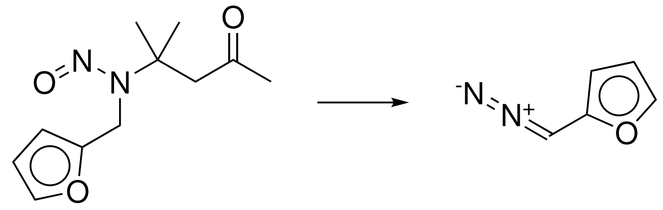

Reaction Example:
alkyl; alkyl: N-alkylation: RMg/Li
Reaxys ID: 10492808
Reagents: n-butyllithium
Solvent: hexane
Time: None
Temperature: None
Condition: None

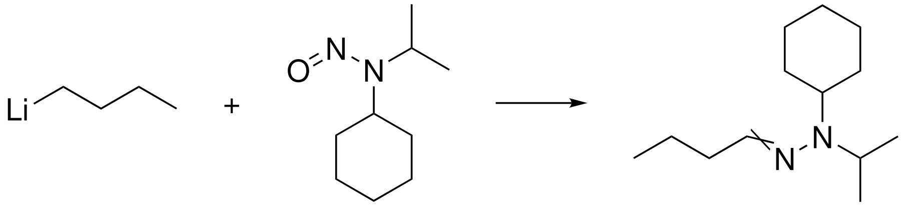

Scheme S4

Reaction Example:
alkyl; alkyl: N3 formation with amine: Pb(OAc)4/Amine/base
Reaxys ID: 1605809
Reagents: O-Methylhydroxylamin, lead(IV) acetate, potassium carbonate
Solvent: None
Time: None
Temperature: None
Condition: 1) CH2Cl2, 2) CH2Cl2, -45 deg C

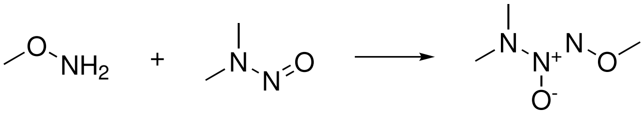

Reaction Example:
alkyl; alkyl: Reduction: Hydride
Reaxys ID: 68792
Reagents: tetrahydrofuran, lithium aluminium tetrahydride
Solvent: None
Time: None
Temperature: None
Condition: None

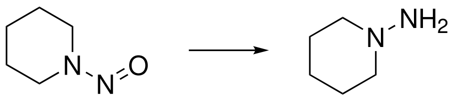

Reaction Example:
alkyl; alkyl: Reduction: Metal
Reaxys ID: 222790
Reagents: sodium hydroxide, aluminium
Solvent: None
Time: None
Temperature: None
Condition: None

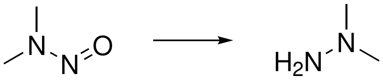

Reaction Example:
alkyl; amide: De/transnitrosation: hv
Reaxys ID: 903837
Reagents: methanol
Solvent: trichlorofluoromethane
Time: None
Temperature: -10
Condition: Irradiation

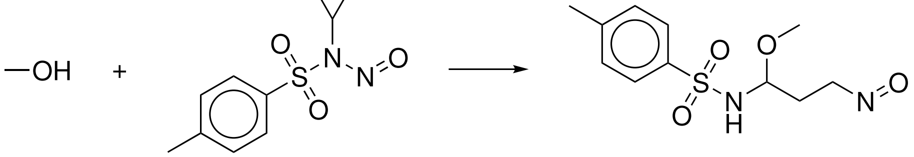

Scheme S5

Reaction Example:
alkyl; amide: Diazonium formation: Base
Reaxys ID: 5403425
Reagents: methanol. KOH-solution, diethyl ether, ammonia, hydrazine
Solvent: None
Time: None
Temperature: None
Condition: Reagens 4:Anilin

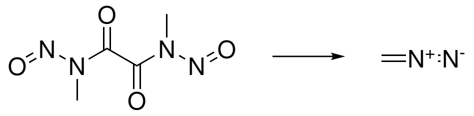

Reaction Example:
alkyl; amide: Diazonium formation: Heating
Reaxys ID: 124378
Reagents: None
Solvent: None
Time: None
Temperature: 55
Condition: Zersetzung,bei 55gradC explosionsartig

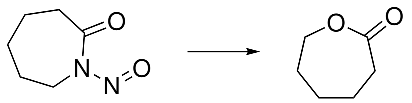

Reaction Example:
alkyl; amide: Reduction: Metal
Reaxys ID: 1751397
Reagents: hydrogenchloride, tin(II) chloride
Solvent: diethyl ether
Time: None
Temperature: None
Condition: Ambient temperature

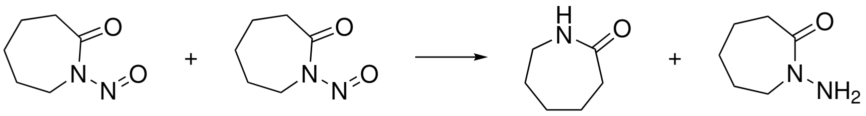

Scheme S6

Reaction Example:
alkyl; carbamate: Diazonium formation: Base
Reaxys ID: 1232060
Reagents: acetic acid
Solvent: None
Time: None
Temperature: None
Condition: (i) NaOMe, AcOEt, EtOH, (ii) /BRN= 506007/

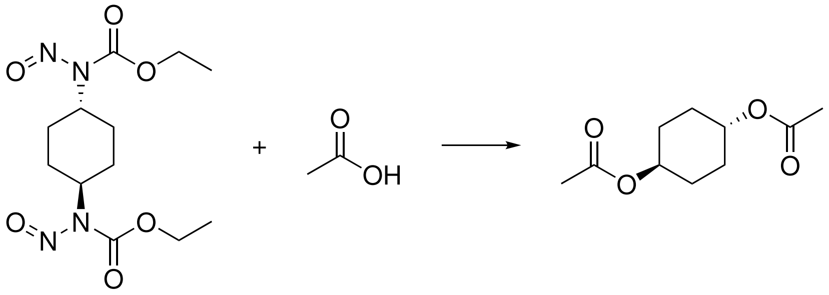

Reaction Example:
alkyl; guanidine: Diazonium formation: Base
Reaxys ID: 6798900
Reagents: aqueous KOH <50 percent >
Solvent: None
Time: None
Temperature: -4
Condition: None

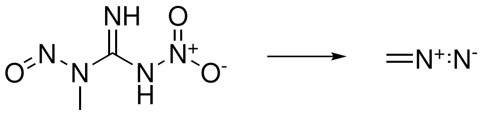

Reaction Example:
alkyl; phenyl: De/transnitrosation: Stabilised carbanion
Reaxys ID: 51797004
Reagents: cyclopentanone, Fmoc-Pro-OH
Solvent: chloroform
Time: 24
Temperature: 80
Condition: None

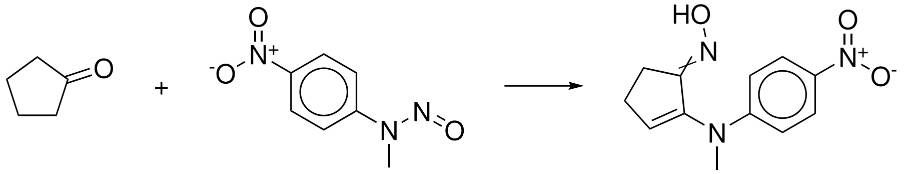

Scheme S7

Reaction Example:
alkyl; phenyl: De/transnitrosation: TM catalyst
Reaxys ID: 42003063
Reagents: diazodimedone, silver hexafluoroantimonate, dichloro(pentamethylcyclopentadienyl)rhodium (III) dimer, acetic acid
Solvent: 1,2-dichloro-ethane
Time: 5
Temperature: 80
Condition: None

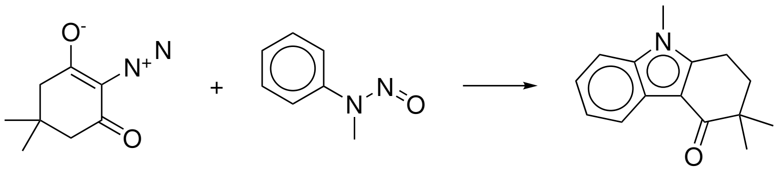

Reaction Example:
alkyl; phenyl: Fischer-Hepp: Acid
Reaxys ID: 261830
Reagents: hydrogenchloride, diethyl ether, ethanol
Solvent: None
Time: None
Temperature: None
Condition: None

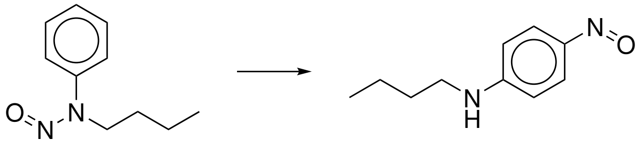

Reaction Example:
alkyl; phenyl: N-alkylation: TM catalyst
Reaxys ID: 54232115
Reagents: 2-[dimethyl(oxo)-Î»6-sulfaneylidene]-1-phenylethan-1-one, Cp*Rh(OAc)2Â·H2O, copper diacetate, silver(I) oxide
Solvent: 2,2,2-trifluoroethanol
Time: 10
Temperature: 100
Condition: Schlenk technique

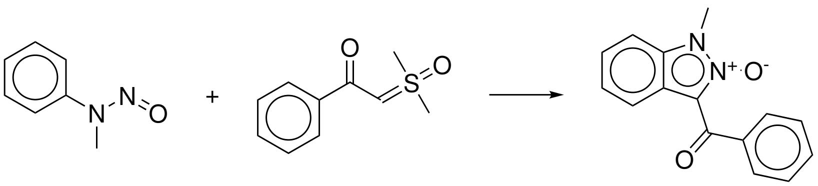

Scheme S8

Reaction Example:
alkyl; phenyl: NO to NO2: HNO3
Reaxys ID: 7979962
Reagents: nitric acid
Solvent: None
Time: None
Temperature: None
Condition: None

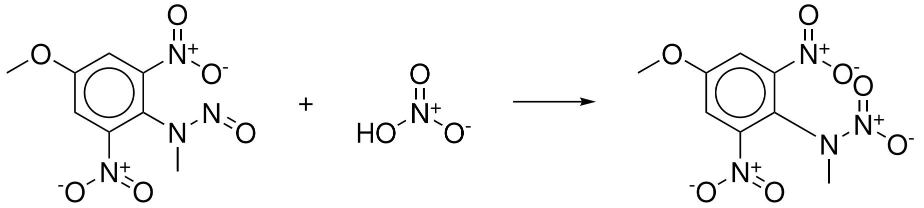

Reaction Example:
alkyl; phenyl: Reduction: Electrochemical reduction
Reaxys ID: 264060
Reagents: sodium acetate, acetic acid
Solvent: None
Time: None
Temperature: None
Condition: bei der elektrolytischen Reduktion an einer verzinnten Kupfer-Kathode

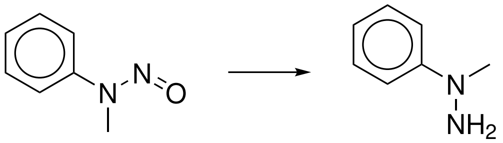

Reaction Example:
alkyl; phenyl: Reduction: Hydride
Reaxys ID: 148152
Reagents: LiAlH4
Solvent: tetrahydrofuran
Time: None
Temperature: None
Condition: None

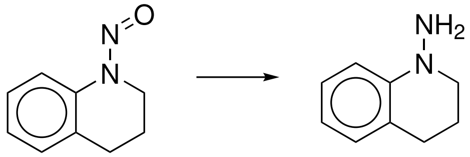

Scheme S9

Reaction Example:
alkyl; phenyl: Reduction: Metal
Reaxys ID: 244667
Reagents: diethyl ether, aluminium amalgam, water
Solvent: None
Time: None
Temperature: None
Condition: None

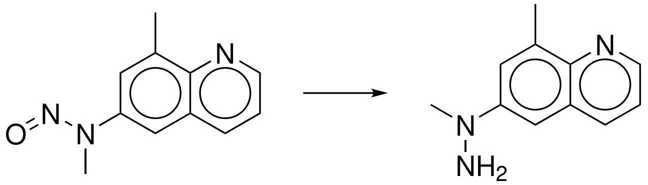

Reaction Example:
alkyl; phenyl: Reduction: S-based reductant
Reaxys ID: 264342
Reagents: sodium hydroxide, sodium dithionite
Solvent: ethanol
Time: 2
Temperature: 90
Condition: None

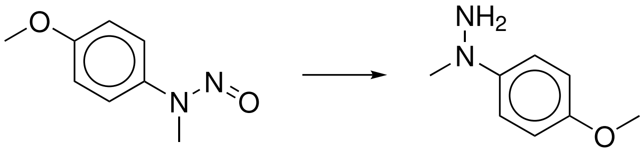

Reaction Example:
alkyl; phenyl: Reduction:TM catalyst
Reaxys ID: 39017822
Reagents: toluene, tert.-butylhydroperoxide, palladium diacetate
Solvent: None
Time: 24
Temperature: 80
Condition: Schlenk technique

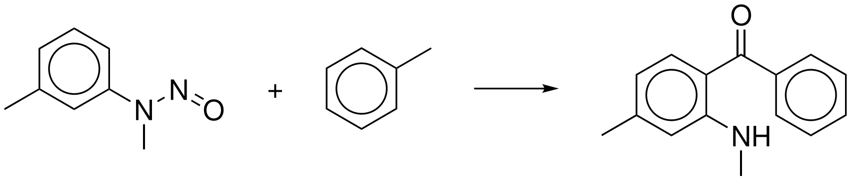

Scheme S10

Reaction Example:
alkyl; phenyl: azo formation: Arene
Reaxys ID: 264134
Reagents: aniline hydrochloride, aniline
Solvent: None
Time: None
Temperature: None
Condition: None

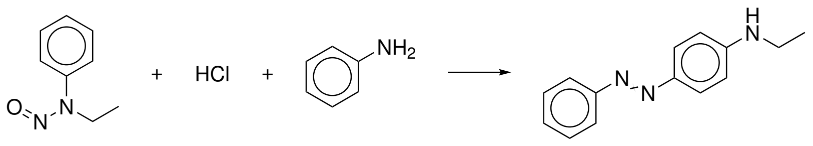

Reaction Example:
alkyl; sulfone: De/transnitrosation: Amine
Reaxys ID: 5112129
Reagents: N-methylaniline
Solvent: 1,4-dioxane, water
Time: None
Temperature: 25
Condition: None

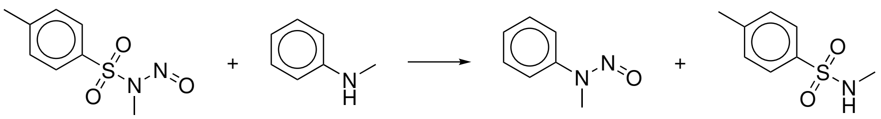

Reaction Example:
alkyl; sulfone: Diazonium formation: Base
Reaxys ID: 37260591
Reagents: (C8H7O2)(NHCHCOO)(CH2C6H5)(CO2C2H5), potassium hydroxide
Solvent: methanol, water
Time: 1.16667
Temperature: 20
Condition: None

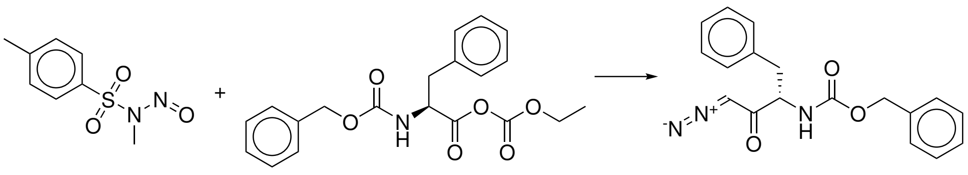

Scheme S11

Reaction Example:
alkyl; urea: Diazonium formation: Base
Reaxys ID: 48638107
Reagents: methacrylic acid methyl ester
Solvent: None
Time: None
Temperature: None
Condition: Alkaline conditions

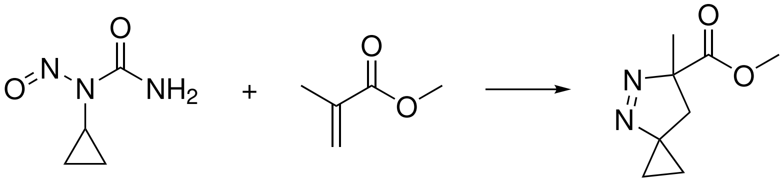

Reaction Example:
alkyl; urea: Reduction: Metal
Reaxys ID: 237336
Reagents: water, acetic acid, zinc
Solvent: None
Time: None
Temperature: None
Condition: ueber mehrere Stufen

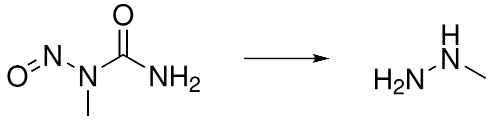

Reaction Example:
imino nitrosamine: Reduction: Hydride
Reaxys ID: 4676243
Reagents: lithium aluminium tetrahydride
Solvent: diethyl ether
Time: None
Temperature: -20
Condition: None

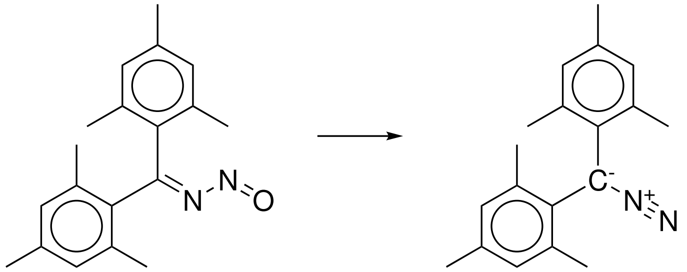

Scheme S12

Reaction Example:
phenyl; B-carboxylic acid: O-Si/C/S bond formation: Acylating reagent
Reaxys ID: 1032821
Reagents: acetic anhydride
Solvent: None
Time: None
Temperature: None
Condition: Heating

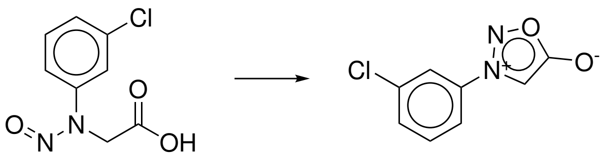

Reaction Example:
phenyl; amide: Diazonium formation: Base
Reaxys ID: 264548
Reagents: aqueous alkali
Solvent: None
Time: None
Temperature: None
Condition: None

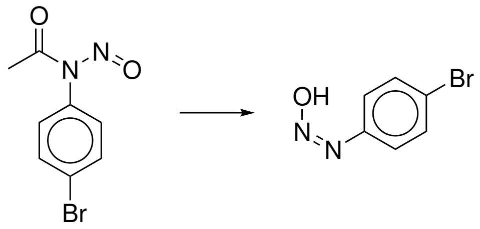

Reaction Example:
phenyl; amide: Diazonium formation: Stabilised carbanion
Reaxys ID: 71779
Reagents: methyl Pyrrole-2-carboxylate, diethyl ether
Solvent: None
Time: None
Temperature: None
Condition: None

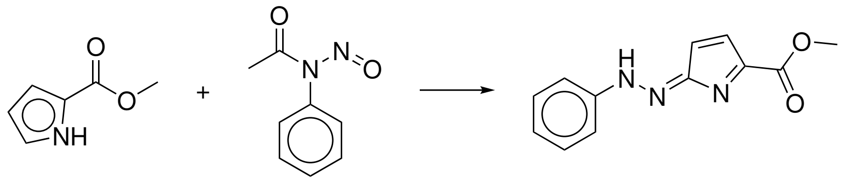

Scheme S13

Reaction Example:
phenyl; amide: azo formation: Arene
Reaxys ID: 580005
Reagents: 1-amino-naphthalene, chloroform
Solvent: None
Time: None
Temperature: None
Condition: None

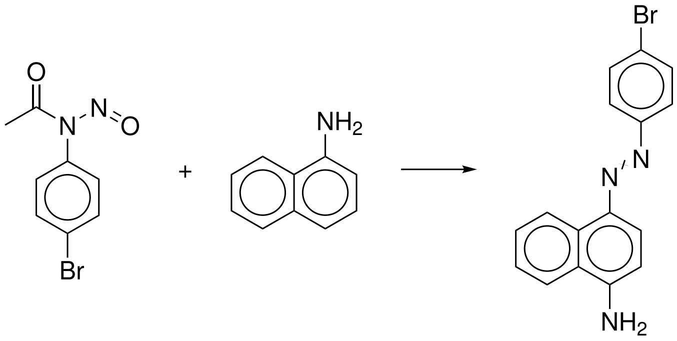

Reaction Example:
phenyl; hydroxyl: O-Si/C/S bond formation: Alkylating reagent
Reaxys ID: 8929
Reagents: diazomethane, diethyl ether
Solvent: None
Time: None
Temperature: 0
Condition: None

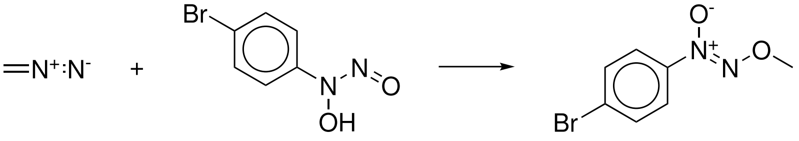

Reaction Example:
phenyl; phenyl: De/transnitrosation: N-nucleophile
Reaxys ID: 26438316
Reagents: potassium amide
Solvent: None
Time: None
Temperature: None
Condition: None

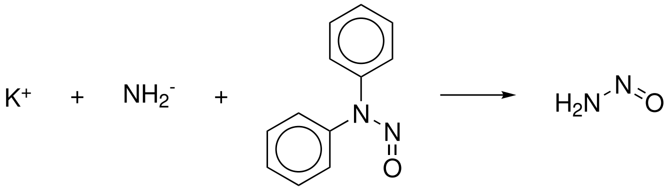

Scheme S14

Reaction Example:
phenyl; phenyl: Fischer-Hepp: Acid
Reaxys ID: 264917
Reagents: hydrogenchloride
Solvent: None
Time: None
Temperature: 20
Condition: None

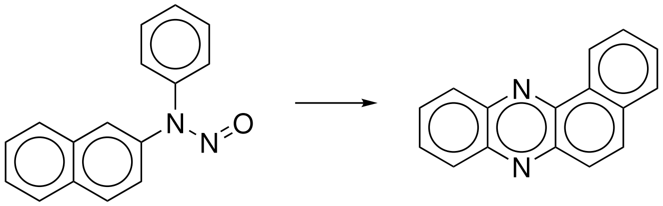

Reaction Example:
phenyl; phenyl: Reduction: Metal
Reaxys ID: 265179
Reagents: ethanol, acetic acid, zinc
Solvent: None
Time: None
Temperature: None
Condition: weiteres Reagens: Aether

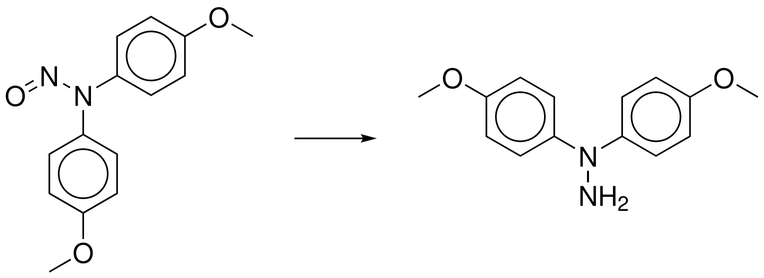

Reaction Example:
phenyl; urea: N-alkylation: Heating
Reaxys ID: 2510510
Reagents: None
Solvent: chloroform
Time: None
Temperature: None
Condition: Heating

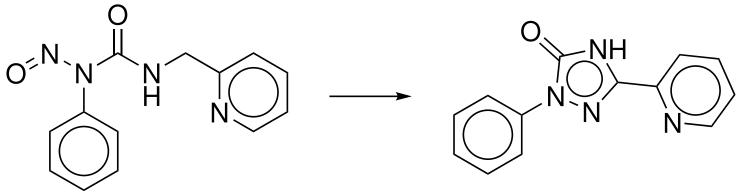

Scheme S15

3 Reactivity screen of *N*-nitrosamines N1-8

The following section details the experimental campaign performed on the 8 *N*-nitrosamines listed in Figure S28.

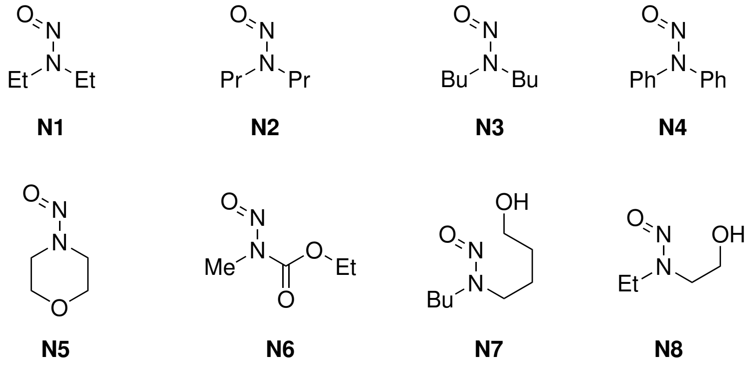

Figure S28: The 8 *N*-nitrosamines used in this study.

3.1 Safety

N-nitrosamines are potential potent carcinogens and the *N*-nitroso group is regarded as alerting towards highly energetic decomposition. To ensure absolute safety with regards to handling them, the following safety protocols were put in place:

- Spill-trays which the screening reactions were performed inside to contain any spills.
- Blunt needles were also used when performing any transfers by needle and during reaction setup using inert atmospheres.
- Butyl-rubber gloves were worn during handling of the nitrosamine reagent bottles and when washing glassware.
- All vials and glass ware in contact with *N*-nitrosamines were disposed of with the rest of the reaction waste to avoid exposing others.
- Reactions were also run at very small scale (0.1 mmol) to avoid producing large amounts of waste, and to minimise exposure.

- All glassware and equipment were decontaminated by treatment with concentrated nitric acid.

For disposal, the quenched reaction mixture were washed (with ethanol and water) into a larger flask containing a basic aqueous solution (20 % NaOH) of $Na_2S_2O_4$ which was then stirred and heated at 40°C overnight, the mixture was then cooled and poured into a hazardous waste container to be disposed of as hazardous waste.

3.2 General procedures

3.2.1 Flow reaction protocol

Flow reactions were carried out at the ROAR facility at Imperial College London. A Vapourtec R-series was used without the autosampler, 0.5 M nitrosamine stock solutions were loaded into the injection loop manually, *via* a Luer-lock syringe, and the switching valve was triggered manually to introduce the nitrosamine into the system and begin the reaction. The reactions were monitored by an in-line IR spectrometer (ReactIR) which was set up as per manufacturer instructions to measure between 0 and 3300 cm^{-1}, the gain was modulated to within recommended limits (ca. 20000), and the instrument was cooled with liquid nitrogen before starting data collection. The scan rate was set to 15 seconds. The in-house software (ICIR) for data processing was unavailable, and a python script was written to extract the results from the raw data.

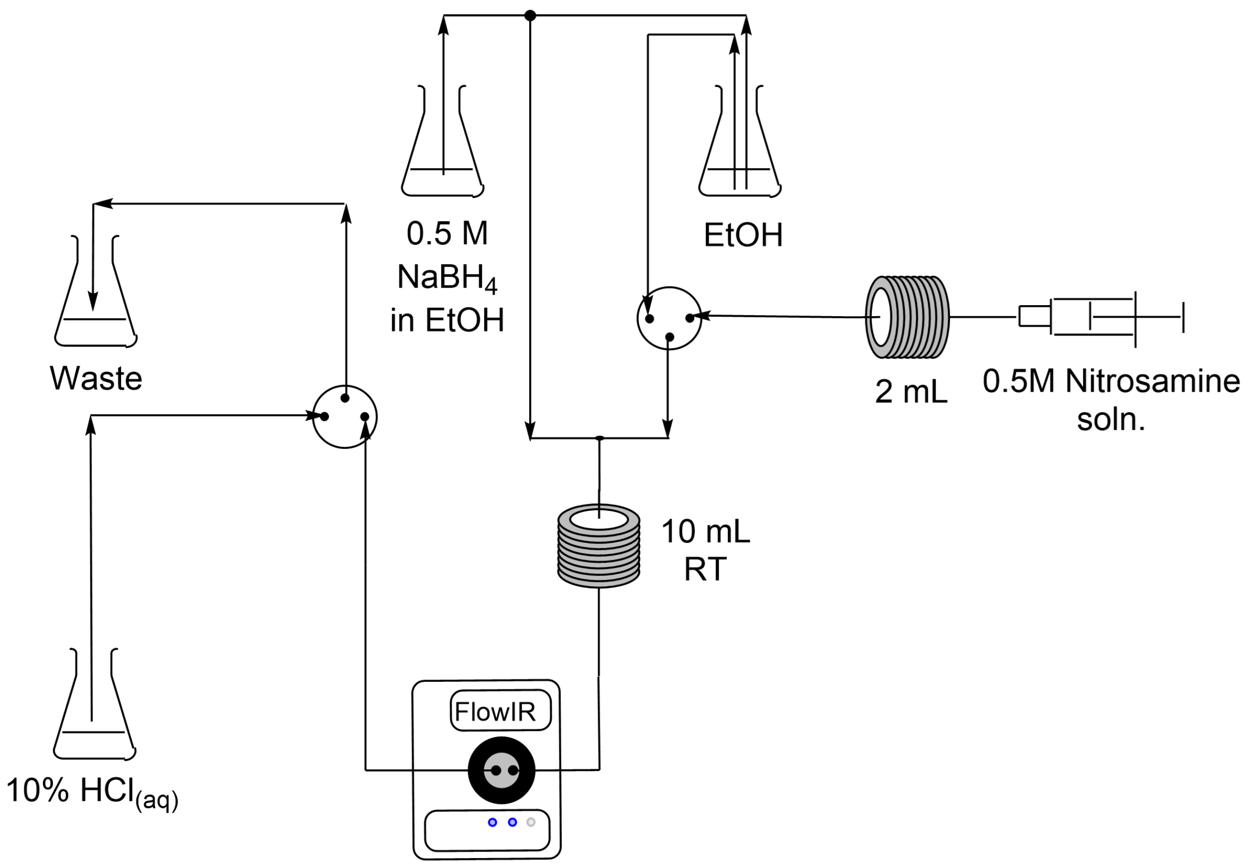

Figure S29: Flow reaction setup.

With reference to Figure S29, the procedure for the flow reactions is as follows: A reference IR spectra for the nitrosamine in the reaction solvent is taken. This is achieved by pumping both influx lines from the solvent reservoir at 5 mL/min (1 minute residence time). Next, the 2 mL injection loop is filled with a 0.5 M solution of nitrosamine in the reaction solvent. The switching valve is triggered, and the nitrosamine solution is injected into the system. At the point of injection, a note is made on the ICIR software, this reference point allows for the capture of the nitrosamine at the time it passes through the IR spectrometer during later processing. The time taken to reach the IR spectrometer was 61 seconds, this calculation accounted for the 136 cm of 1mm internal diameter tubing connecting the injection port to the 10 mL reactor coil and then from the reactor coil to the in-line IR probe. After the reference was taken, one of the influx lines was switched to pump from the 0.5 M $NaBH_4$ solution (the tubing from this line was shorter than that of the line used to inject

the nitrosamine solution, to ensure that the reagent reached the reactor coil before the nitrosamine) and the injection loop was charged with a further 2 mL of the 0.5 M nitrosamine solution. The process described above was repeated and the spectra were collected for analysis. This method allows for qualitative analysis of the effect of the reagent on the nitroso group through comparison of the area under the curve of the nitroso stretch region (1499 - 1402 cm^{-1}) for the reference and the reaction.

3.2.2 Flow reaction analysis workflow

The ICIR software which is provided with the ReactIR spectrometer was unavailable after data collection at the ROAR facility, and because of this, analysis of the data was performed in python. The notes that were made about the time of injections for the reference and reaction spectra were exported as excel files, along with the raw IR data for the ReactIR run. The analysis workflow is as follows: The full IR traces for each time point were zoomed into the wavenumber region of interest (N=O $_{1499-1402 cm^{-1}}$, and C=O $_{1752-1700 cm^{-1}}$). The note for the injection time is entered and the average of three spectra surrounding this timepoint are taken as a reference spectra to which baseline correction is performed using asymmetric least squares (ALS) smoothing. This baseline is then subtracted from 7 consecutive spectra, which start from 45 seconds after the injection note, to allow time for the reaction mixture to pass through the system. The area under the curve is calculated using Simpson's integrator from numpy. The area curve is then subjected to baseline correction by the ALS algorithm so that the change in areas can be compared, this is necessary due to the continuous shifting in baseline height during the data collection. The above procedure is performed for both the reference and reaction injections, and the area under the curve (AUC) traces are plotted against each other using matplotlib.

3.2.3 Batch protocols

Stock solutions of the 8 nitrosamines (0.1 M) were prepared in ethanol or THF. The batch reactor setup consisted of an aluminium heating block which sat on top of a hotplate and allowed for consistent stirring (400 rpm) and heating of four 15 mL reaction vials with 20 mm magnetic stir bars. (Figure S30)

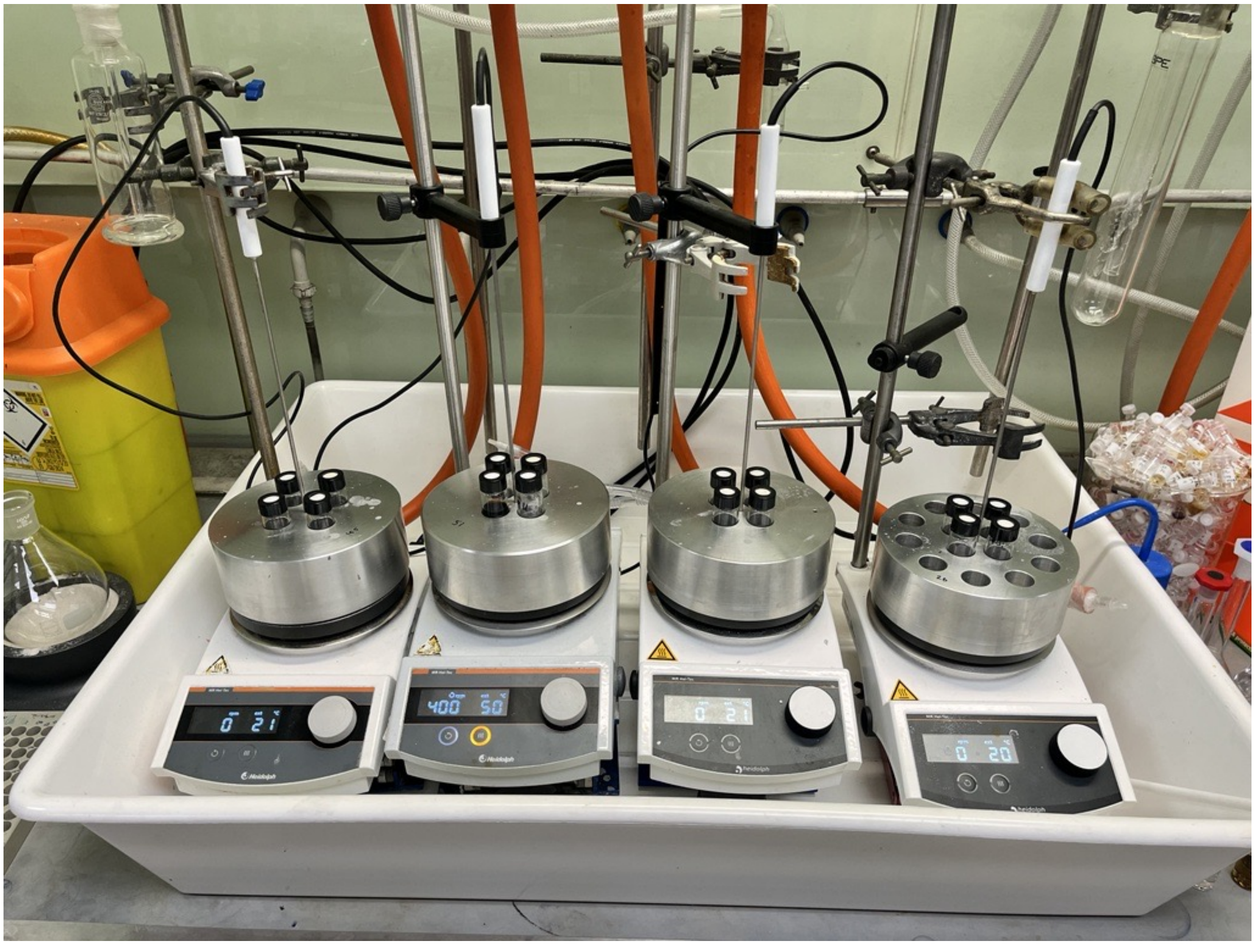

Figure S30: Batch reaction setup.

In performing the batch reactions, stock solutions of the reagents were prepared and the required volume of reagent was first added via 1000 μL Gilson pipette (or 1 mL syringe in the case of DiBAL-H and HBr/AcOH) into a 15 mL vial. The required amount of solvent was next added to the reaction vial to ensure solubility. Heating was then applied *via* a stirrer hotplate to the reaction vial if required, and once at temperature, *N*-nitrosamine

stock solution was added with stirring and the timer was started. Reactions were run at 24 hours if at room temperature, and 16 hours if run at 50°C. After the allotted reaction time, reactions were quenched with a quenching solution, and subjected to sampling for conversion and reaction product analysis. Conversions of the reactions were monitored by GC (conditions 9 and 10) and HPLC (all other conditions). N4 was monitored by HPLC for all experiments.

An internal standard (IS) stock solution of 1,3,5-trimethoxybenzene (TMB) (1 mL, 0.3 M, 0.3 mmol) was also added to the reactions *via* Gilson pipette. The chosen internal standard is not stable to acid, and therefore in the reactions with acid, the internal standard solution was either omitted from the reaction, ignored during conversion calculation or added after the acid was quenched. Consequently, HPLC and GC calibrations with an without internal standard are presented in Figures S52 to S60 and Figures S61 to S67 respectively.

The procedure for product identification involved a mini-workup of the quenched reaction samples, suspending the quenched reaction mixture over saturated sodium chloride solution (3 mL) and ethyl acetate or diethyl ether (3 mL) inside the reaction vial to minimise exposure. The vial was sealed and inverted numerous times to mix the two phases, and after settling, a sample was taken from the upper ethyl acetate layer and directly analysed by GC-MSMS.

In the case of N4 (*N*-nitrosodiphenylamine), the GC-MSMS protocol was unable to separate authentic samples of the nitrosamine and amine, and thus, for this nitrosamine, product identification was achieved via LC-MSMS. LC-MS/MS samples were prepared directly from the reaction mixture post-quench at 10-50 ug/mL in methanol. The same LC-MSMS protocol was also used in cases where there was ambiguous results by GC-MSMS for the other 7 nitrosamines used in the study.

3.3 Experimental conditions

Table S2: Summary of the reaction conditions for the batch HPLC reactivity screen.

#	Reagent	t (h)	T (°C)	[Reagent] (M)	Vol Reagent (μL)	Vol N1-N8 Stock (μL)	Vol H_2O (μL)	Vol EtOH / THF(*) (μL)	Vol TMB Stock (μL)	Quench
1	$H_2O_{2(aq)}$	24	r.t.	2	1000	1000	0	0	1000 [4]	Catalase (1 mL, 1.3 mg/mL)
2	CH_3CO_3H	24	r.t.	5.33	375	1000	0	1000	1000 [6]	1. $NaHCO_3$ sat. (aq) 4 mL<br>2. Catalase (1 mL, 1.3 mg/mL)
3	HBr 33% wt. (in AcOH)	24	r.t.	5.5	300 +/- 10	1000	0	4000	1000 [6]	NaOH (aq) (1 mL, 2 M)
4	HCl (aq)	24	r.t.	2	1000	1000	0	0	1000 [5]	NaOH (aq) (1 mL, 2 M)
5	HCl (aq)	16	50	2	1000	1000	0	0	1000 [5]	NaOH (aq) (1 mL, 2 M)
6	HCl (EtOH)	24	r.t.	1.25	1600	1000	0	0	1000 [5]	NaOH (aq) (1 mL, 2 M)
7	HCl (EtOH)	16	50	1.25	1600	1000	0	0	1000 [5]	NaOH (aq) (1 mL, 2 M)
8	$NaBH_4$	24	r.t.	0.40	4000	1000	0	0	1000 [4]	MeOH (1 mL)
9	DiBAL-H	0.5	r.t.	1.00	1000	2000	0	1000*	1000 [4]	1. EtOAc (1mL)<br>2. H_2O (0.2 mL)<br>3. NaOH (0.4mL 2 M)
10	DiBAL-H	1	r.t.	1.00	1000	2000	0	1000*	1000 [4]	1. EtOAc (1mL)<br>2. H_2O (0.2 mL)<br>3. NaOH (0.4mL 2 M)
11	$Na_2S_2O_4$	24	r.t.	1.00	1000	500	2000	2000	1000 [4]	H_2O_2 (aq), (1 mL, 1 M)
12	$Na_2S_2O_4$ [1]	24	r.t.	1.00	1000	500	2000	2000	1000 [4]	H_2O_2 (aq), (1 mL, 1 M)
13	$Na_2S_2O_4$ [1]	16	50	1.00	1000	500	3000	2000	1000 [4]	H_2O_2 (aq), (1 mL, 1 M)
14	$Na_2S_2O_4$ [2]	16	50	0.25	4000	500	0	2000	1000 [4]	H_2O_2 (aq), (1 mL, 1 M)
15	$Na_2S_2O_4$ [2]	3	50	0.25	4000	500	0	2000	1000 [4]	H_2O_2 (aq), (1 mL, 1 M)
16	$Na_2S_2O_4$ [2]	1	50	0.25	4000	500	0	2000	1000 [4]	H_2O_2 (aq), (1 mL, 1 M)
17	$Na_2S_2O_4$ [3]	16	50	0.25	4000	500	0	2000	0	H_2O_2 (aq), (1 mL, 1 M)
18	Na_2SO_3	24	r.t.	1.00	1000	500	1000	1000	1000 [4]	H_2O_2 (aq), (1 mL, 1 M)
19	Na_2SO_3 [2]	16	50	0.25	4000	500	0	2000	1000 [4]	H_2O_2 (aq), (0.5 mL, 2 M)
20	NaOH	24	r.t.	2.00	1000	1000	0	0	1000 [4]	AcOH aq (1 mL, 2 M)
21	NaOEt 21% w/v	24	r.t.	2.55	1000	1000	0	1000	1000 [4]	H_2O (5 mL)

[1] Reaction charged with additional NaOH (aq) (1 M, 1mL)
[2] Stock solution prepared in NaOH (aq) (1 M)
[3] Stock solution prepared in NaOH (aq) (20% w/v)
[4] Internal standard included at start of reaction.
[5] Internal standard included at end of reaction.
[6] Internal standard not considered in conversion analysis.

Reaction condition 1: $\boldsymbol{H_2O_2}$ 16 oven-dried 15 mL screw-top vials each equipped with a 20 mm magnetic flea were inserted into 4 aluminium heating blocks. To each of the vials was added H_2O_2 $_{(aq)}$ (1 mL, 2 M, 2 mmol), internal standard stock solution (1 mL, 0.3 M, 0.3 mmol TMB in EtOH) and nitrosamine stock solution (1 mL, *ca.* 100 mM, 0.1 mmol in ethanol). Duplicate reactions were peformed for each nitrosamine. The reaction vials were secured with lids with silicon septa and stirring was started. After the alloted reaction time (24 h), the reaction was quenched by addition of Catalase $_{(aq)}$ (1 mL, 1.3 mg/mL) with stirring for 10 minutes. The quenched reaction mixture was filtered through 0.2 μm hydrophillic syringe filters and sampled into HPLC sample vials and subjected to analysis by HPLC. An LC-MS/MS sample for N4 was prepared from the filtered HPLC sample, diluted with methanol. The quenched reaction mixture was treated with brine (2.5 mL) and ethyl acetate (2.5 mL), the mixture was inverted and left until phase separation occurred. A sample from the organic layer was then analysed by GC-MS/MS. GC-MS/MS analysis can be found in Figures S109 and S110. LC-MS/MS analysis of N4 can be found in Figure S111.

N4 was further analysed by 1H and ^{13}C NMR. The screening reaction was repeated, with H_2O_2 $_{(aq)}$ (1 mL, 2 M, 2 mmol) and nitrosamine stock solution (1 mL, *ca.* 100 mM, 0.1 mmol in ethanol). The TMB stock solution was substituted with 1 mL of ethanol. After 24 h, the reaction was suspended over equal portions of brine and ethyl acetate (ca. 15 mL). The aqueous layer was washed with a further 2 15 mL portions of ethyl acetate, and the organic extracts were combined, dried over $MgSO_4$, filtered and the solvent was removed by rotary evaporator. The crude reaction product was dissolved in deuterated methanol and analysed by ^{1}H and ^{13}C NMR (Section 10).

Reaction condition 2: $\boldsymbol{CH_3CO_3H}$ 16 oven-dried 15 mL screw-top vials each equipped with a 20 mm magnetic flea were inserted into 4 aluminium heating blocks. To each of the vials was added CH_3CO_3H (38-40 % w/v, Fisher) (375 μL, 2 mmol), internal standard stock solution (1 mL, 0.3 M, 0.3 mmol TMB in EtOH) and nitrosamine stock solution (1 mL, *ca.*

100 mM, 0.1 mmol in ethanol). Duplicate reactions were performed for each nitrosamine. The reaction vials were secured with lids with silicon septa and stirring was started. After the allotted reaction time (24 h), the reaction was quenched by addition of $NaHCO_3$ $_{(sat.\ (aq))}$ (4 mL) followed by Catalase $_{(aq)}$ (1 mL, 1.3 mg/mL) with stirring for 10 minutes. The quenched reaction mixture was filtered through 0.2 μm hydrophilic syringe filters and sampled into HPLC sample vials and subjected to analysis by HPLC. An LC-MS/MS sample for N4 was prepared from the filtered HPLC sample, diluted with methanol. The quenched reaction mixture was treated with brine (2.5 mL) and ethyl acetate (2.5 mL), the mixture was inverted and left until phase separation occurred. A sample from the organic layer was then analysed by GC-MS/MS. GC-MS/MS analysis can be found in Figures S112 and S113. LC-MS/MS analysis of N4 can be found in Figure S114.

Reaction Condition 3 : HBr 33% wt. in AcOH 8 oven-dried 15 mL screw-top vials each equipped with a 20 mm magnetic flea were inserted into 2 aluminium heating blocks. To each of the vials was added nitrosamine stock solution (1 mL, *ca.* 100 mM, 0.1 mmol in ethanol) and internal standard stock solution (1 mL, 0.3 M, 0.3 mmol TMB in EtOH) and ethanol (4 mL). The reaction vials were secured with lids with silicon septa and stirring was started. A nitrogen atmosphere was introduced via the septa and the reaction vials were purged in this state for 10 minutes at medium nitrogen flow. After purging the N_2 flow was reduced and HBr 33% wt. in acetic acid (0.3 mL +/- 0.01 mL, 1.56-1.71 mmol, Sigma) was added *via* a 1 mL syringe at 2 minute intervals between reactions, removing the purge needle after the addition of acid. After the allotted reaction time (24 h), the reaction was quenched by addition of NaOH (1 mL, 2 M) with stirring for 10 minutes. The quenched reaction mixture was filtered through 0.2 μm hydrophilic syringe filters and sampled into HPLC sample vials and analysed by HPLC. An LC-MS/MS sample for N4 was prepared from the filtered HPLC sample, diluted with methanol. The quenched reaction mixture was treated with brine (2.5 mL) and ethyl acetate (2.5 mL), the mixture was inverted and left until phase separation occurred. A sample from the organic layer was then analysed by

GC-MS/MS. GC-MS/MS analysis can be found in Figure S116. LC-MS/MS analysis of N4 and N8 can be found in Figures S117 and S118.

Reaction Conditions 4 & 5: $HCl_{(aq)}$ 16 oven-dried 15 mL screw-top vials each equipped with a 20 mm magnetic flea were inserted into 4 aluminium heating blocks either at room temperature (Condition 4) or heated to 50 °C (Condition 5). To each of the vials was added $HCl_{(aq)}$ (1 mL, 2 M, 2 mmol) followed by nitrosamine stock solution (1 mL, *ca.* 100 mM, 0.1 mmol in ethanol). Duplicate reactions were performed for each nitrosamine. The reaction vials were secured with lids with silicon septa and stirring was started. After the allotted reaction time (Condition 4: 24 h, Condition 5: 16 h), the reaction was quenched by addition of NaOH $_{(aq)}$ (1 mL, 2 M) with stirring for 10 minutes. Internal standard stock solution (1 mL, 0.3 M, 0.3 mmol TMB in EtOH) was added after quenching. The reaction mixture was filtered through 0.2 μm hydrophilic syringe filters and sampled into HPLC sample vials and analysed by HPLC. An LC-MS/MS sample for N4 was prepared from the filtered HPLC sample, diluted with methanol. For condition 5, the quenched reaction mixture was treated with brine (2.5 mL) and ethyl acetate (2.5 mL), the mixture was inverted to mix and left until phase separation occurred. A sample from the organic layer was then analysed by GC-MS/MS. GC-MS/MS analysis for condition 5 can be found in figures S120 and S121. LC-MS/MS analysis of N4 for both conditions 4 and 5 can be found in figures S119 and S122.

Reaction Conditions 6 & 7: $HCl_{(EtOH)}$ 16 oven-dried 15 mL screw-top vials each equipped with a 20 mm magnetic flea were inserted into 4 aluminium heating blocks either at room temperature (Condition 6) or heated to 50 °C (Condition 7). To each of the vials was added HCl $_{EtOH}$ (1.6 mL, 1.25 M, 2 mmol) followed by nitrosamine stock solution (1 mL, *ca.* 100 mM, 0.1 mmol in ethanol) (1 nitrosamine per reaction vial) such that the reaction was performed in duplicate for each nitrosamine. The reaction vials were secured with lids with silicon septa and stirring was started. After the allotted reaction time (Condition 6: 24 h, Condition 7: 16 h), the reaction was quenched by addition of NaOH $_{(aq)}$ (1 mL, 2

M) with stirring. Internal standard stock solution (1 mL, 0.3 M, 0.3 mmol TMB in EtOH) was added after quenching. The reaction mixture was filtered through 0.2 μm hydrophilic syringe filters and sampled into HPLC sample vials and subjected to analysis by HPLC. An LC-MS/MS sample for N4 was prepared from the filtered HPLC sample, diluted with methanol. For condition 7, the quenched reaction mixture was treated with brine (2.5 mL) and diethyl ether (2.5 mL), the mixture was inverted to mix and left until phase separation occurred. A sample from the organic layer was then subjected to analysis by GC-MS/MS. GC-MS/MS analysis for condition 7 can be found in Figure S124. LC-MS/MS analysis of N4 for both conditions 6 and 7 can be found in Figures S123 and S125. LC-MS/MS analysis of N7 and N8 for condition 7 can be found in Figures S126 and S127.

In the case of N5 in condition 6, the analysis by GC-MS/MS showed no reaction product, while the conversion of the starting material by HPLC was 56%. The reaction was repeated so that NMR analysis could be performed. HCl_{EtOH} (1.6 mL, 1.25 M, 2 mmol) followed by nitrosamine stock solution (1 mL, *ca.* 100 mM, 0.1 mmol in ethanol) and 1 mL ethanol were added to a 15 mL reaction vial, the reaction was stirred at 400 rpm for 24 h. The pH of the reaction mixture was adjusted to 9 (measured by pH paper) using 1M NaOH and then the reaction mixture was suspended over equal portions of brine and dichloromethane (DCM) (ca. 15 mL), the organic layer was removed and the aqueous layer was washed a further two times with 15 mL DCM. The organic portions were combined and dried over $MgSO_4$ and the solvent was removed by rotary evaporator. The crude mixture was dissolved in deuterated chloroform and analysed by ^{1}H and ^{13C} NMR.

Reaction Condition 8: $NaBH_4$ 8 oven-dried 15 mL screw-top vials each equipped with a 20 mm magnetic flea were inserted into 2 aluminium heating blocks. To each of the vials was added nitrosamine stock solution (1 mL, *ca.* 100 mM, 0.1 mmol in THF) and internal standard stock solution (1 mL, 0.3 M, 0.3 mmol TMB in THF). The reaction vials were secured with lids with silicon septa and stirring was started. A nitrogen atmosphere was introduced via the septa and the reaction vials were purged in this state for 10 minutes

at medium nitrogen flow. After purging the N_2 flow was reduced and $NaBH_4$ (4 mL, 0.4 M, 1.6 mmol, Fisher) in ethanol was added *via* a 5 mL syringe at 2 minute interval between reactions, removing the purge needle after addition. After the allotted reaction time (24 h), the reaction was quenched by addition of MeOH (1 mL) with stirring for 10 minutes. The quenched reaction mixture was filtered through 0.2 μm hydrophilic syringe filters and sampled into HPLC sample vials and subjected to analysis by HPLC. An LC-MS/MS sample for N4 was prepared from the filtered HPLC sample, diluted with methanol. The quenched reaction mixture was treated with brine (2.5 mL) and ethyl acetate (2.5 mL), the mixture was inverted to mix and left until phase separation occurred. A sample from the organic layer was then subjected to analysis by GC-MS/MS. GC-MS/MS analysis for condition 8 can be found in Figure S129. LC-MS/MS analysis of N4, N7 and N8 can be found in Figures S130 to S132.

Reaction Conditions 9 & 10: DiBAL-H 8 oven-dried 15 mL screw-top vials each equipped with a 20 mm magnetic flea were inserted into two aluminium heating blocks as described in the general experimental procedure section of this document. To each of the vials was added THF (1 mL) and internal standard stock solution (1 mL, 0.3 M, 0.3 mmol TMB in THF). nitrosamine stock solution (2 mL, *ca.* 100 mM in THF) was next added (1 nitrosamine per reaction vial). The reaction vials were secured with lids with silicon septa and stirring was started. A nitrogen atmosphere was introduced via the septa and the reaction vials were purged in this state for 10 minutes at medium nitrogen flow. After purging the N_2 flow was reduced and DIBAL-H (1 mL, 1 M, 1 mmol in hexane, Sigma) was added *via* a 1 mL syringe at 2 minute intervals between reactions and the purge needle was removed after addition. Reactions were sampled at 0.5 and 1 hours by removing 0.5 mL of the reaction mixture with a 1 mL syringe and adding this to a sample vial containing ethyl acetate (1.6 mL) and NaOH (1M, 0.2 mL) the mixture was stirred at 300 rpm for *ca.* 10 minutes. The now quenched reaction sample was filtered through a 0.2 μm hydrophobic syringe filter into GC sample vials and analysed directly by HPLC (*N*-nitrosodiphenylamine)

and GC-MS/MS (all other nitrosamines). An LC-MS/MS sample for N4 was prepared from the filtered HPLC sample, diluted with methanol. GC-MS/MS analysis can be found in Figures S133 and S135. LC-MS/MS analysis of N4 and N8 can be found in Figures S134, S136, S140 and S141.

Reaction Condition 11: $Na_2S_2O_4$ 16 oven-dried 15 mL screw-top vials each equipped with a 20 mm magnetic flea were inserted into 4 aluminium heating blocks either at room temperature as described in the general experimental procedure section of this document. To each of the vials was added $Na_2S_2O_4$ $_{(aq)}$ (1 mL, 1 M, 1 mmol) followed by deionised water (3 mL), absolute ethanol (2 mL), internal standard stock solution (1 mL, 0.3 M, 0.3 mmol TMB in ethanol) and nitrosamine stock solution (0.5 mL, *ca.* 100 mM, 0.05 mmol in ethanol) (1 nitrosamine per reaction vial) such that the reaction was performed in duplicate for each nitrosamine. The reaction vials were secured with lids with silicon septa and stirring was started. After the allotted reaction time (24 h), the reaction was quenched by addition of H_2O_2 $_{(aq)}$ (1 mL, 1 M) with stirring for 10 minutes. The reaction mixture was filtered through 0.2 μm hydrophilic syringe filters and sampled into HPLC sample vials and subjected to analysis by HPLC. An LC-MS/MS sample for N4 was prepared from the filtered HPLC sample, diluted with methanol. The quenched reaction mixture was treated with brine (2.5 mL) and ethyl acetate (2.5 mL), the mixture was inverted to mix and left until phase separation occurred. A sample from the organic layer was then subjected to analysis by GC-MS/MS. GC-MS/MS analysis can be found in Figures S144 and S145. LC-MS/MS analysis of N4 can be found in Figure S146.

Reaction Conditions 12 & 13: $Na_2S_2O_{4(aq)}$ in NaOH $_{(aq)}$ (0.1 M) 16 oven-dried 15 mL screw-top vials each equipped with a 20 mm magnetic flea were inserted into 4 aluminium heating blocks either at room temperature (Condition 12) or heated to 50 °C (Condition 13). To each of the vials was added $Na_2S_2O_4$ $_{(aq)}$ (1 mL, 1 M, 1 mmol) followed by NaOH $_{(aq)}$ (1 mL, 1 M), deionised water (2 mL), absolute ethanol (2 mL), internal standard stock solution (1 mL, 0.3 M, 0.3 mmol TMB in ethanol) and nitrosamine stock

solution (0.5 mL, *ca.* 100 mM, 0.05 mmol in ethanol) (1 nitrosamine per reaction vial) such that the reaction was performed in duplicate for each nitrosamine. The reaction vials were secured with lids with silicon septa and stirring was started. After the allotted reaction time (Condition 12: 24 h, Condition 13: 16 h), the reaction was quenched by addition of H_2O_2 $_{(aq)}$ (1 mL, 1 M) with stirring for 10 minutes. The reaction mixture was filtered through 0.2 μm hydrophilic syringe filters and sampled into HPLC sample vials and subjected to analysis by HPLC. An LC-MS/MS sample for N4 was prepared from the filtered HPLC sample in methanol. The quenched reaction mixture was treated with brine (2.5 mL) and ethyl acetate (2.5 mL), the mixture was inverted to mix and left to settle. A sample from the organic layer was then subjected to analysis by GC-MS/MS. GC-MS/MS analysis can be found in figures S147, S149 and S150. LC-MS/MS analysis of N4 in conditions 12 and 13 can be found in figures S148 and S151.

Reaction Conditions 14, 15 & 16: $Na_2S_2O_4$ $_{(aq)}$ in NaOH $_{(aq)}$ (1 M) 16 oven-dried 15 mL screw-top vials each equipped with a 20 mm magnetic flea were inserted into 4 aluminium heating blocks heated to 50 °C as described in the general experimental procedure section of this document. To each of the vials was added $Na_2S_2O_4$ $_{(aq)}$ in NaOH $_{(aq)}$ (1 M) (4 mL, 0.25 M, 1 mmol) followed by absolute ethanol (2 mL), internal standard stock solution (1 mL, 0.3 M, 0.3 mmol TMB in ethanol) and nitrosamine stock solution (0.5 mL, *ca.* 100 mM, 0.05 mmol in ethanol) (1 nitrosamine per reaction vial) such that the reaction was performed in duplicate for each nitrosamine. The reaction vials were secured with lids with silicon septa and stirring was started. After the allotted reaction time (Condition 14: 16 h, Condition 15: 3 h, Condition 16: 1 h), the reaction was quenched by addition of H_2O_2 $_{(aq)}$ (1 mL, 1 M) with stirring. The reaction mixture was filtered through 0.2 μm hydrophilic syringe filters and sampled into HPLC sample vials and subjected to analysis by HPLC. An LC-MS/MS sample for N4 was prepared from the filtered HPLC sample in methanol. For conditions 14 and 15, the quenched reaction mixture was treated with brine (2.5 mL) and ethyl acetate (2.5 mL), the mixture was inverted to mix and left to settle. A sample from

the organic layer was then subjected to analysis by GC-MS/MS. GC-MS/MS analysis was performed for conditions 14 and 15 and can be found in figures S152, S153, S155 and S156. LC-MS/MS analysis for N4 in conditions 14 and 15 can be found in figures S154 and S157.

Reaction Condition 17: $Na_2S_2O_{4(aq)}$ in NaOH $_{(aq)}$ (20 % w/v) 16 oven-dried 15 mL screw-top vials each equipped with a 20 mm magnetic flea were inserted into 4 aluminium heating blocks heated to 50 °C. To each of the vials was added $Na_2S_2O_4$ $_{(aq)}$ in NaOH $_{(aq)}$ (20 % w/v) (4 mL, 0.25 M, 1 mmol) followed by absolute ethanol (2 mL) and Nitrosamine stock solution (0.5 mL, *ca.* 100 mM, 0.05 mmol in ethanol) (1 nitrosamine per reaction vial) such that the reaction was performed in duplicate for each nitrosamine. The reaction vials were secured with lids with silicon septa and stirring was started. After the allotted reaction time (16 h), the reaction was quenched by addition of H_2O_2 $_{(aq)}$ (1 mL, 1 M) with stirring. The reaction mixture was filtered through 0.2 μm hydrophilic syringe filters and sampled into HPLC sample vials and subjected to analysis by HPLC. An LC-MS sample for N4 was prepared from the filtered HPLC sample, diluted in methanol. The quenched reaction mixture was treated with brine (2.5 mL) and ethyl acetate (2.5 mL), the mixture was inverted to mix and left until phase separation occurred. A sample from the organic layer was then subjected to analysis by GC-MS/MS. GC-MS/MS analysis can be found in figure S158. LC-MS/MS analysis of N4 can be found in figure S159.

Reaction Condition 18: $Na_2SO_{3(aq)}$ 24 oven-dried 15 mL screw-top vials each equipped with a 20 mm magnetic flea were inserted into 6 aluminium heating blocks at room temperature. To each of the vials was added Na_2SO_3 $_{(aq)}$ (1 mL, 1 M, 1 mmol) followed by deionised water (1mL) absolute ethanol (1 mL), internal standard stock solution (1 mL, 0.3 M, 0.3 mmol TMB in ethanol) and Nitrosamine stock solution (0.5 mL, *ca.* 100 mM, 0.05 mmol in ethanol) (1 nitrosamine per reaction vial) such that the reaction was performed in duplicate for each nitrosamine. The reaction vials were secured with lids with silicon septa and stirring was started. After the allotted reaction time (24 h), the reaction was quenched by addition of H_2O_2 $_{(aq)}$ (1 mL, 1 M) with stirring. The reaction mixture was

filtered through 0.2 μm hydrophilic syringe filters and sampled into HPLC sample vials and subjected to analysis by HPLC.

Reaction Condition 19: Na_2SO_3 $_{(aq)}$ in NaOH $_{(aq)}$ (1 M) 16 oven-dried 15 mL screw-top vials each equipped with a 20 mm magnetic flea were inserted into 4 aluminium heating blocks heated to 50 °C. To each of the vials was added Na_2SO_3 $_{(aq)}$ in NaOH $_{(aq)}$ (1 M) (4 mL, 0.25 M, 1 mmol) followed by absolute ethanol (2 mL), internal standard stock solution (1 mL, 0.3 M, 0.3 mmol TMB in ethanol) and Nitrosamine stock solution (0.5 mL, *ca.* 100 mM, 0.05 mmol in ethanol) (1 nitrosamine per reaction vial) such that the reaction was performed in duplicate for each nitrosamine. The reaction vials were secured with lids with silicon septa and stirring was started. After the allotted reaction time (24 h), the reaction was quenched by addition of H_2O_2 $_{(aq)}$ (0.5 mL, 2 M) with stirring. The reaction mixture was filtered through 0.2 μm hydrophilic syringe filters and sampled into HPLC sample vials and subjected to analysis by HPLC. An LC-MS/MS sample for N4 was prepared from the filtered HPLC sample in methanol. The quenched reaction mixture was treated with brine (2.5 mL) and ethyl acetate (2.5 mL), the mixture was inverted to mix and until phase separation occurred. A sample from the organic layer was then subjected to analysis by GC-MS/MS. GC-MS/MS analysis can be found in Figures S160 and S161. LC-MS/MS analysis of N4 can be found in Figure S162.

Reaction Condition 20: NaOH $_{(aq)}$ 16 oven-dried 15 mL screw-top vials each equipped with a 20 mm magnetic flea were inserted into 4 aluminium heating blocks as described in the general experimental procedure section of this document. To each of the vials was added NaOH $_{(aq)}$ (1 mL, 2 M, 2 mmol) followed by internal standard stock solution (1 mL, 0.3 M, 0.3 mmol TMB in ethanol) and Nitrosamine stock solution (1 mL, *ca.* 100 mM, 0.1 mmol in ethanol) (1 nitrosamine per reaction vial) such that the reaction was performed in duplicate for each nitrosamine. The reaction vials were secured with lids with silicon septa and stirring was started. After the allotted reaction time (24 h), the reaction was quenched by addition of acetic acid $_{(aq)}$ (1 mL, 2 M) with stirring. The reaction mixture

was filtered through 0.2 μm hydrophilic syringe filters and sampled into HPLC sample vials and subjected to analysis by HPLC.

Reaction Condition 21: NaOEt 16 oven-dried 15 mL screw-top vials each equipped with a 20 mm magnetic flea were inserted into 4 aluminium heating blocks at room temperature as described in the general experimental procedure section of this document. To each of the vials was added NaOEt (1 mL, 2.55 M, 2.55 mmol) followed by internal standard stock solution (1 mL, 0.3 M, 0.3 mmol TMB in ethanol) and Nitrosamine stock solution (1 mL, *ca.* 100 mM, 0.1 mmol in ethanol) (1 nitrosamine per reaction vial) such that the reaction was performed in duplicate for each nitrosamine. The reaction vials were secured with lids with silicon septa and stirring was started. After the allotted reaction time (24 h), the reaction was quenched by addition of deionised water (5 mL) with stirring. The reaction mixture was filtered through 0.2 μm hydrophilic syringe filters and sampled into HPLC sample vials and subjected to analysis by HPLC. An LC-MS/MS sample for N4 was prepared from the filtered HPLC sample in methanol. The quenched reaction mixture was treated with brine (2.5 mL) and ethyl acetate (2.5 mL), the mixture was inverted to mix and left to settle. A sample from the organic layer was then subjected to analysis by GC-MS/MS. GC-MS/MS analysis can be found in figures S164 and S165. LC-MS/MS analysis of N4 can be found in figure S166.

4 Reaction screening results

4.1 Conversion calculation method and information

For repeatability, a discussion of the method for conversion calculation is included here. The conversion tables prepared in excel are provided in section 4.2, and the column headings are explained below:

- **Nitrosamine**: The nitrosamine (N1-8) under study followed by the experiment number (reactions mostly performed in duplicate, some in triplicate).
- **Nitrosamine Area Response**: The HPLC area response for the nitrosamine in the reaction sample.
- **TMB Area Response**: The HPLC area response for the internal standard (1,3,5-trimethoxybenzene) in the reaction sample.
- **Response Factor**: The Nitrosamine Area Response divided by the TMB area response.
- **[Nitrosamine if no rxn] (mM)**: The concentration of the nitrosamine expected if no conversion took place. Calculated from the total sample volume, volume of nitrosamine stock solution added, and nitrosamine stock solution concentration.
- **[Nitrosamine] (mM)**: The concentration of the nitrosamine in the reaction sample calculated from the response factor (or the area response) and the corresponding calibration curve equation (see sections 5.1 and 5.2).
- **Conversion**: The calculated nitrosamine concentration divided by the concentration if no reaction multiplied by 100.
- **Average conversion (%)**: The average conversion for each nitrosamine over all repeats.

- **Std deviation (%)**: The standard deviation of the conversion results over all repeats.

Conversion with respect to the nitrosamine was calculated in Excel using the calibration equations in Tables S3 to S5. Due to the wide range of reaction conditions in the screen, calibrations with internal standard were plotted such that the internal standard concentration could be varied within the reaction. Firstly, for those reactions using an internal standard the response factor was calculated from the ratio of the analyte peak area to the internal standard peak area (Equation (S1)). If the internal standard was ignored or otherwise due to the presence of acid in the reaction mixture, the absolute integral was used in place of the response factor.

$$\frac{\text{Area }_{\text{Analyte}}}{\text{Area }_{\text{IS}}} = RF \tag{S1}$$

For those calibrations plots with linear fit line equations, the equations take the forms in Equation (S2) and Equation (S3) for the calibrations including and excluding internal standard respectively.

$$RF = a + b \cdot \frac{\text{[Analyte] (mM)}}{\text{[IS] (mM)}} \tag{S2}$$

$$\text{Area }_{\text{Analyte}} = a + b \cdot \text{ [Analyte] (mM)} \tag{S3}$$

To find the concentration of the analyte, Equations (S2) and (S3) can be rearanged to give Equations (S4) and (S5) respectively:

$$\text{[Analyte] (mM)} = \frac{RF - a}{b} \cdot \text{[IS] (mM)} \tag{S4}$$

$$\text{[Analyte] (mM)} = \frac{\text{Area}_{\text{Analyte}} - a}{b} \tag{S5}$$

Similarly, calibration graphs with an exponential fit line equation with forms Equa-

tions (S6) and (S7) can be rearranged for the analyte concentration to give Equations (S8) and (S9).

$$RF = y_0 + A \cdot e^{R_0 \cdot \frac{[\text{Analyte}]\ (\text{mM})}{[\text{IS}]\ (\text{mM})}} \tag{S6}$$

$$\text{Area}_{\ \text{Analyte}} = y_0 + A \cdot \exp(R_0 \cdot [\text{Analyte}]\,(\text{mM})) \tag{S7}$$

$$[\text{Analyte}]\ (\text{mM}) = \frac{[\text{IS}]\ (\text{mM}) \cdot \ln\left(\frac{RF - y_0}{A}\right)}{R_0} \tag{S8}$$

$$[\text{Analyte}]\,(\text{mM}) = \frac{\ln\left(\frac{\text{Area}_{\text{Analyte}} - y_0}{A}\right)}{R_0} \tag{S9}$$

Equation S10 calculates the expected concentration of the analyte if no reaction were to occur ($[\text{Analyte}]_0$) from the starting nitrosamine stock solution concentration and the total reaction volume at the time the HPLC sample is taken (Equation (S10)). The conversion is then calculated as the average result over two or three repeat reactions (equation S11).

$$[\text{Analyte}]_0\ (\text{mM}) = \frac{[\text{Stock}]_{(\text{Analyte})} \cdot \text{V}_{\ \text{Stock}}}{\text{V}_{\text{Reaction}}} \tag{S10}$$

$$\text{Conversion}\ (\%) = \frac{[\text{Analyte}]\ (\text{mM})}{[\text{Analyte}]_0\ (\text{mM})} \cdot 100 \tag{S11}$$

4.2 Conversion tables

Condition 1	Total sample volume (mL): 4	Vol nitrosamine (mL)	1	Vol TMB	1	TMB STOCK	TMB 06/11/2023	T (°C)	22
Nitrosamine	Nitrosamine Area Response	TMB Area Response	Response Factor	[TMB] (mM)	[Nitrosamine if no rxn] (mM)	[Nitrosamine] (mM)	Conversion (%)	Average Conversion (%)	Std deviation (%)
N1 1	63462.69	150444.35	0.42	7.56	29.40	28.53	2.96		
N1 2	64514.07	151260.41	0.43	7.56	29.40	28.84	1.89	2.42	0.53
N2 1	103729.80	53058.02	1.96	7.56	28.73	29.57	-2.94		
N2 2	102891.41	52277.93	1.97	7.56	28.73	29.77	-3.64	-3.29	0.35
N3 1	93402.93	52373.28	1.78	7.56	26.19	25.12	4.10		
N3 2	93963.63	52217.56	1.80	7.56	26.19	25.34	3.24	3.67	0.43
N4 1	185696.86	51188.94	3.63	7.56	25.20	20.69	17.89		
N4 2	186052.99	50671.61	3.67	7.56	25.20	20.99	16.70	17.29	0.59
N5 1	86234.90	51979.69	1.66	7.56	28.96	28.49	1.61		
N5 2	87627.73	51616.99	1.70	7.56	28.96	29.15	-0.67	0.47	1.14
N6 1	88189.02	48569.22	1.82	7.56	33.74	32.44	3.86		
N6 2	88862.24	47561.90	1.87	7.56	33.74	33.38	1.05	2.45	1.40
N7 1	87461.45	53512.30	1.63	7.56	27.84	27.13	2.52		
N7 2	87509.15	53058.11	1.65	7.56	27.84	27.39	1.61	2.06	0.45
N8 1	114936.13	52799.72	2.18	7.56	28.69	27.55	4.01		
N8 2	114718.18	53828.92	2.13	7.56	28.69	26.97	6.01	5.01	1.00

Figure S31: Condition 1 HPLC conversion table.

Condition 2	Total sample volume (mL): 8.375	Vol nitrosamine (mL)	1	T (°C)	22	
**Nitrosamine**	**Nitrosamine Area Response**	**[Nitrosamine if no rxn] (mM)**	**[Nitrosamine] (mM)**	**Conversion (%)**	**Average Conversion (%)**	**Std deviation (%)**
N1 1	53762.68	14.04	12.84	8.56		
N1 2	53846.29	14.04	12.86	8.42	8.49	0.07
N2 1	45184.35	13.72	12.54	8.63		
N2 2	45436.41	13.72	12.61	8.11	8.37	0.26
N3 1	40305.34	12.51	11.33	9.42		
N3 2	42117.34	12.51	11.85	5.26	7.34	2.08
N4 1	6211.00	12.03	0.72	94.03		
N4 2	3705.07	12.03	0.49	95.91	94.97	0.94
N5 1	37926.37	13.83	12.00	13.21		
N5 2	37418.15	13.83	11.85	14.34	13.78	0.57
N6 1		16.11	-0.14	100.86		
N6 2		16.11	-0.14	100.86	100.86	0.00
N7 1	37075.86	13.29	11.18	15.89		
N7 2	38772.46	13.29	11.71	11.89	13.89	2.00
N8 1	51030.43	13.70	12.11	11.65		
N8 2	50572.24	13.70	12.00	12.45	12.05	0.40

Figure S32: Condition 2 HPLC conversion table.

Condition 3	Total sample volume (mL): 7.3	Vol nitrosamine (mL)	1	T (°C)	22	
**Nitrosamine**	**Nitrosamine Area Response**	**[Nitrosamine if no rxn] (mM)**	**[Nitrosamine] (mM)**	**Conversion (%)**	**Average Conversion (%)**	**Std deviation (%)**
N1 1	54191.52	13.64	12.94	5.11		
N1 2	52919.37	13.64	12.63	7.40	6.25	1.15
N2 1	49090.93	14.65	13.65	6.84		
N2 2	49129.01	14.65	13.66	6.76	6.80	0.04
N3 1	45529.85	14.66	12.83	12.48		
N3 2	45172.14	14.66	12.73	13.18	12.83	0.35
N4 1	81316.97	14.03	8.09	42.30		
N4 2	81043.83	14.03	8.07	42.51	42.40	0.10
N5 1	36101.07	15.35	11.44	25.46		
N5 2	36139.67	15.35	11.45	25.39	25.43	0.04
N6 1	6520.61	14.02	2.14	84.77		
N6 2	6415.26	14.02	2.10	85.03	84.90	0.13
N7 1	46371.47	14.55	14.09	3.16		
N7 2	47710.28	14.55	14.51	0.28	1.72	1.44
N8 1	45079.36	14.48	10.69	26.19		
N8 2	45229.77	14.48	10.72	25.94	26.07	0.12

Figure S33: Condition 3 HPLC conversion table.

Condition 4	Total sample volume (mL): 4	Vol nitrosamine (mL)	1	Vol TMB	1	TMB STOCK	TMB 05/09/2023	T (°C)	22
Nitrosamine	Nitrosamine Area Response	TMB Area Response	Response Factor	[TMB] (mM)	[Nitrosamine if no rxn] (mM)	[Nitrosamine] (mM)	Conversion (%)	Average Conversion (%)	Std deviation (%)
N11_220	57495.65	154220.61	0.37	7.55	25.34	25.21	0.51		
N12_220	56316.34	152794.69	0.37	7.55	25.34	24.93	1.63	1.58	0.85
N13_220	56299.30	154279.79	0.36	7.55	25.34	24.69	2.59		
N21	89657.24	52182.62	1.72	7.55	25.15	25.91	-3.04		
N22	89374.60	50671.41	1.76	7.55	25.15	26.61	-5.82	-5.40	1.78
N23	91897.76	51375.27	1.79	7.55	25.15	26.99	-7.34		
N31	85547.76	50706.55	1.69	7.55	25.01	23.74	5.08		
N32	83704.77	51374.06	1.63	7.55	25.01	22.93	8.33	6.16	1.54
N33	86342.97	51161.17	1.69	7.55	25.01	23.75	5.05		
N41	77532.68	25759.39	3.01	3.77	12.49	8.32	33.38		
N42	84406.34	26234.72	3.22	3.77	12.49	8.98	28.08	37.41	9.69
N43	59958.97	26109.35	2.30	3.77	12.49	6.15	50.77		
N51	75932.12	51702.35	1.47	7.55	25.52	25.22	1.20		
N52	77116.33	50609.09	1.52	7.55	25.52	26.16	-2.48	-1.18	1.68
N53	78154.09	51409.85	1.52	7.55	25.52	26.10	-2.25		
N61		50779.68	0.00	7.55	25.08	-0.20	100.82		
N62		51949.21	0.00	7.55	25.08	-0.20	100.82	100.82	0.00
N63		51807.11	0.00	7.55	25.08	-0.20	100.82		
N71	88117.92	50789.07	1.73	7.55	25.15	28.82	-14.57		
N72	87460.30	50926.79	1.72	7.55	25.15	28.52	-13.38	-12.07	2.74
N73	85273.60	51945.92	1.64	7.55	25.15	27.23	-8.26		
N81	87643.91	52852.01	1.66	7.55	21.73	20.99	3.42		
N82	87603.56	56652.67	1.55	7.55	21.73	19.58	9.91	6.22	2.72
N83	84811.04	52187.07	1.63	7.55	21.73	20.57	5.34		

Figure S34: Condition 4 HPLC conversion table.

Condition 5	Total sample volume (mL): 5	Vol nitrosamine (mL)	1	Vol TMB	1	TMB STOCK	TMB 24/10/2023	T (°C)	50
Nitrosamine	Nitrosamine Area Response	TMB Area Response	Response Factor	[TMB] (mM)	[Nitrosamine if no rxn] (mM)	[Nitrosamine] (mM)	Conversion (%)	Average Conversion (%)	Std deviation (%)
N1 1	48123.54	125497.17	0.38	6.04	20.56	20.75	-0.93		
N1 2	47584.46	128949.16	0.37	6.04	20.56	19.98	2.84	0.96	1.88
N2 1	68447.81	36387.50	1.88	6.04	21.86	22.74	-4.00		
N2 2	67801.19	35991.13	1.88	6.04	21.86	22.77	-4.16	-4.08	0.08
N3 1	73968.98	42529.69	1.74	6.04	21.41	19.59	8.51		
N3 2	67581.81	42776.68	1.58	6.04	21.41	17.80	16.87	12.69	4.18
N4 1	20978.26	31719.69	0.66	6.04	20.50	2.70	86.83		
N4 2	21650.28	31098.95	0.70	6.04	20.50	2.84	86.14	86.48	0.35
N5 1	71488.78	42554.44	1.68	6.04	24.66	23.06	6.49		
N5 2	72345.96	41706.60	1.73	6.04	24.66	23.81	3.46	4.97	1.51
N6 1		54143.15	0.00	6.04	23.37	-0.16	100.70		
N6 2		54042.10	0.00	6.04	23.37	-0.16	100.70	100.70	0.00
N7 1	71925.81	35541.17	2.02	6.04	23.65	26.99	-14.13		
N7 2	72857.26	35989.90	2.02	6.04	23.65	27.00	-14.17	-14.15	0.02
N8 1	86081.14	42547.89	2.02	6.04	21.42	20.47	4.40		
N8 2	83344.45	43047.33	1.94	6.04	21.42	19.60	8.49	6.45	2.05

Figure S35: Condition 5 HPLC conversion table.

Condition 6	Total sample volume (mL): 4.6	Vol nitrosamine (mL)	1	Vol TMB	1	TMB STOCK	TMB 05/09/2023	T (°C)	22
**Nitrosamine**	**Nitrosamine Area Response**	**TMB Area Response**	**Response Factor**	**[TMB] (mM)**	**[Nitrosamine if no rxn] (mM)**	**[Nitrosamine] (mM)**	**Conversion (%)**	**Average Conversion (%)**	**Std deviation (%)**
N1 1_220	97760.14	46330.32	2.11	6.56	22.04	23.45	-6.40		
N1 2_220	95854.38	47739.86	2.01	6.56	22.04	22.29	-1.16	-2.04	3.25
N1 3_220	92326.47	47169.54	1.96	6.56	22.04	21.72	1.43		
N2 1	80308.94	46782.32	1.72	6.56	21.87	22.51	-2.94		
N2 2	81502.67	47000.03	1.73	6.56	21.87	22.74	-4.01	-2.04	2.08
N2 3	79157.18	47841.94	1.65	6.56	21.87	21.68	0.84		
N3 1	77690.07	38812.44	2.00	6.56	21.75	24.49	-12.57		
N3 2	73247.90	47402.38	1.55	6.56	21.75	18.91	13.05	3.42	11.39
N3 3	75262.17	46936.88	1.60	6.56	21.75	19.62	9.78		
N4 1		47798.76	0.00	6.56	21.72	0.09	99.57		
N4 2		47871.43	0.00	6.56	21.72	0.09	99.57	99.57	0.00
N4 3			#DIV/0!	6.56	21.72	#DIV/0!	#DIV/0!		
N5 1	30506.75	48134.63	0.63	6.56	22.19	9.55	56.95		
N5 2	33078.97	48270.41	0.69	6.56	22.19	10.32	53.51	55.66	1.53
N5 3	30932.29	48303.93	0.64	6.56	22.19	9.65	56.51		
N6 1		56935.76	0.00	6.56	21.81	-0.18	100.82		
N6 2		46639.04	0.00	6.56	21.81	-0.18	100.82	100.82	0.00
N6 3		47067.71	0.00	6.56	21.81	-0.18	100.82		
N7 1	75333.71	47848.22	1.57	6.56	21.87	22.69	-3.72		
N7 2	75514.09	46511.43	1.62	6.56	21.87	23.41	-7.04	-6.02	1.62
N7 3	76808.73	47204.22	1.63	6.56	21.87	23.46	-7.28		
N8 1	68084.91	48388.70	1.41	6.56	18.89	15.50	17.98		
N8 2	69946.99	47215.30	1.48	6.56	18.89	16.31	13.67	15.20	1.97
N8 3	69646.15	47158.85	1.48	6.56	18.89	16.26	13.94		

Figure S36: Condition 6 HPLC conversion table.

Condition 7	Total sample volume (mL): 5.6	Vol nitrosamine (mL)	1	Vol TMB	1	TMB STOCK	TMB 24/10/2023	T (°C)	50
Nitrosamine	Nitrosamine Area Response	TMB Area Response	Response Factor	[TMB] (mM)	[Nitrosamine if no rxn] (mM)	[Nitrosamine] (mM)	Conversion (%)	Average Conversion (%)	Std deviation (%)
N1 1	12302.15	115024.68	0.11	5.39	18.36	5.29	71.18		
N1 2	12420.01	116793.75	0.11	5.39	18.36	5.26	71.34	71.26	0.08
N2 1	33852.07	38179.06	0.89	5.39	19.52	9.40	51.84		
N2 2	32269.70	39419.10	0.82	5.39	19.52	8.66	55.66	53.75	1.91
N3 1	33188.60	38516.52	0.86	5.39	19.12	8.68	54.57		
N3 2	31686.56	38574.12	0.82	5.39	19.12	8.28	56.68	55.63	1.06
N4 1	1178.86	39072.51	0.03	5.39	18.30	0.18	99.02		
N4 2	1248.21	38328.55	0.03	5.39	18.30	0.19	98.97	98.99	0.02
N5 1	1057.97	39014.62	0.03	5.39	22.02	0.46	97.90		
N5 2	1125.37	39449.83	0.03	5.39	22.02	0.48	97.83	97.87	0.04
N6 1		39600.13	0.00	5.39	20.87	-0.15	100.70		
N6 2		39495.26	0.00	5.39	20.87	-0.15	100.70	100.70	0.00
N7 1	34201.57	39054.29	0.88	5.39	21.11	10.16	51.87		
N7 2	34179.16	39060.73	0.88	5.39	21.11	10.15	51.91	51.89	0.02
N8 1		39977.51	0.00	5.39	19.12	0.07	99.64		
N8 2		39531.85	0.00	5.39	19.12	0.07	99.64	99.64	0.00

Figure S37: Condition 7 HPLC conversion table.

Condition 8	Total sample volume (mL): 7	Vol nitrosamine (mL)	1	Vol TMB	1	TMB STOCK	TMB 28/11/2023	T (°C)	22
**Nitrosamine**	**Nitrosamine Area Response**	**TMB Area Response**	**Response Factor**	**[TMB] (mM)**	**[Nitrosamine if no rxn] (mM)**	**[Nitrosamine] (mM)**	**Conversion (%)**	**Average Conversion (%)**	**Std deviation (%)**
N1 1	31211.53	88756.90	0.35	4.33	14.03	13.65	2.69		
N1 2	30073.49	86246.51	0.35	4.33	14.03	13.54	3.50	3.09	0.41
N2 1	48488.50	26747.07	1.81	4.33	15.93	15.70	1.48		
N2 2	38840.35	20636.57	1.88	4.33	15.93	16.31	-2.34	-0.43	1.91
N3 1	44794.61	25951.62	1.73	4.33	14.62	13.93	4.72		
N3 2	46002.49	25652.34	1.79	4.33	14.62	14.48	1.01	2.87	1.85
N4 1	85539.16	25868.32	3.31	4.33	14.49	10.64	26.60		
N4 2	89235.56	25037.51	3.56	4.33	14.49	11.61	19.88	23.24	3.36
N5 1	39751.60	26724.36	1.49	4.33	14.33	14.65	-2.22		
N5 2	38570.80	26302.49	1.47	4.33	14.33	14.45	-0.79	-1.50	0.72
N6 1		27690.37	0.00	4.33	14.72	-0.12	100.80		
N6 2		24215.51	0.00	4.33	14.72	-0.12	100.80	100.80	0.00
N7 1	37018.83	23217.94	1.59	4.33	16.40	15.16	7.54		
N7 2	39260.80	23287.88	1.69	4.33	16.40	16.05	2.10	4.82	2.72
N8 1	51757.99	25817.52	2.00	4.33	14.90	14.54	2.38		
N8 2	52482.70	25081.88	2.09	4.33	14.90	15.18	-1.87	0.25	2.13

Figure S38: Condition 8 HPLC conversion table.

Conditions 9 & 10	Total sample volume (mL): 6	Vol Nitrosamine (mL):	2	Vol TMB (mL):	1	TMB STOCK:	TMB 28/11/2023	Temperature (°C):	22
**Nitrosamine**	**Nitrosamine peak retention time**	**Nitrosamine Peak m/z**	**Nitrosamine Area Response**	**TMB Area Response**	**Response Factor**	**Sample [TMB] (mM)**	**[Sample if no rxn] (mM)**	**[Nitrosamine] (mM)**	**Conversion (%)**
N1 0.5	3.15707	102	568285.12	5634218.04	0.101	1.15	7.12	2.03	72
N1 1	3.16497	102	423828.20	7852579.58	0.054	1.15	7.12	1.07	85
N2 0.5	6.50522	130	1550387.38	4057916.47	0.382	1.15	8.08	2.71	66
N2 1	6.50567	130	1343944.20	7539958.75	0.178	1.15	8.08	1.13	86
N3 0.5	10.77955	158	2447003.54	3736892.48	0.655	1.15	7.42	2.90	61
N3 1	10.78402	158	2937902.50	6777977.27	0.433	1.15	7.42	1.82	75
N4 0.5 (HPLC)	4.02292	N/A	18545.49	8492.52	2.184	1.15	7.35	1.77	76
N4 1 (HPLC)	4.02458	N/A	12641.82	9059.33	1.395	1.15	7.35	1.10	85
N5 0.5		No peak		7266117.37	0.000	1.15	7.27	-0.09	101
N5 1		No peak		5304771.43	0.000	1.15	7.27	-0.09	101
N6 0.5 (103)	2.57078	103	13562.36	9139923.54	0.001	1.15	7.46	0.78	90
N6 1 (103)	2.57092	103	6970.90	4425197.42	0.002	1.15	7.46	0.80	89
N6 0.5 (132)		No peak		9139923.54	0.000	1.15	7.46	0.00	100
N6 1 (132)		No peak		4425197.42	0.000	1.15	7.46	0.00	100
N7 0.5	12.9327	157	8883826.80	10050000.00	0.884	1.15	8.32	4.10	51
N7 1	12.91693	157	1080940.20	6126640.14	0.176	1.15	8.32	0.82	90
N8 0.5	8.01777	87	656719.84	9084177.73	0.072	1.15	7.56	2.32	69
N8 1		No peak		5430796.13	0.000	1.15	7.56	-0.06	101

Figure S39: Conditions 9 and 10 GC conversion table.

Condition 11	Total sample volume (mL): 8.5	Vol nitrosamine (mL)	0.5	Vol TMB	1	TMB STOCK	TMB 05/10/2023	T (°C)	22
Nitrosamine	Nitrosamine Area Response	TMB Area Response	Response Factor	[TMB] (mM)	[Nitrosamine if no rxn] (mM)	[Nitrosamine] (mM)	Conversion (%)	Average Conversion (%)	Std deviation (%)
N1 1	15046.02	79355.67	0.19	3.52	6.62	6.04	8.80		
N1 2	14817.61	79128.67	0.19	3.52	6.62	5.97	9.91	9.35	0.55
N2 1	22009.14	24520.87	0.90	3.52	6.23	6.22	0.11		
N2 2	21245.15	24864.52	0.85	3.52	6.23	5.91	5.07	2.59	2.48
N3 1	21429.75	24666.86	0.87	3.52	6.24	5.72	8.36		
N3 2	21211.30	24915.99	0.85	3.52	6.24	5.60	10.19	9.28	0.92
N4 1	7722.41	24545.18	0.31	3.52	5.89	0.77	87.00		
N4 2	7512.57	24715.29	0.30	3.52	5.89	0.74	87.42	87.21	0.21
N5 1	13758.16	24837.31	0.55	3.52	5.92	4.49	24.06		
N5 2	13445.33	24982.65	0.54	3.52	5.92	4.37	26.18	25.12	1.06
N6 1	7596.25	25004.30	0.30	3.52	6.56	2.45	62.62		
N6 2	7585.90	25137.93	0.30	3.52	6.56	2.43	62.88	62.75	0.13
N7 1	22284.38	24803.91	0.90	3.52	6.37	6.82	-7.01		
N7 2	21541.85	24712.28	0.87	3.52	6.37	6.61	-3.69	-5.35	1.66
N8 1	25302.27	24846.73	1.02	3.52	6.32	6.03	4.52		
N8 2	25021.98	24917.91	1.00	3.52	6.32	5.95	5.84	5.18	0.66

Figure S40: Condition 11 HPLC conversion table.

Condition 12	Total sample volume (mL): 8.5	Vol nitrosamine (mL)	0.5	Vol TMB	1	TMB STOCK	TMB 26/09/2023	T (°C)	22
**Nitrosamine**	**Nitrosamine Area Response**	**TMB Area Response**	**Response Factor**	**[TMB] (mM)**	**[Nitrosamine if no rxn] (mM)**	**[Nitrosamine] (mM)**	**Conversion (%)**	**Average Conversion (%)**	**Std deviation (%)**
N11	15046.02	79355.67	0.19	3.59	5.86	6.15	-4.93		
N12	14817.61	79128.67	0.19	3.59	5.86	6.07	-3.66	-4.29	0.64
N21	22009.14	24520.87	0.90	3.59	6.29	6.33	-0.61		
N22	21245.15	24864.52	0.85	3.59	6.29	6.01	4.39	1.89	2.50
N31	21429.75	24666.86	0.87	3.59	6.30	5.82	7.59		
N32	21211.30	24915.99	0.85	3.59	6.30	5.70	9.43	8.51	0.92
N41	7722.41	24545.18	0.31	3.59	6.02	0.78	87.08		
N42	7512.57	24715.29	0.30	3.59	6.02	0.75	87.49	87.28	0.21
N51	13758.16	24837.31	0.55	3.59	6.59	4.57	30.63		
N52	13445.33	24982.65	0.54	3.59	6.59	4.44	32.57	31.60	0.97
N71	22284.38	24803.91	0.90	3.59	6.25	6.94	-11.00		
N72	21541.85	24712.28	0.87	3.59	6.25	6.72	-7.55	-9.28	1.72
N81	25302.27	24846.73	1.02	3.59	6.22	6.14	1.28		
N82	25021.98	24917.91	1.00	3.59	6.22	6.05	2.65	1.96	0.68

Figure S41: Condition 12 HPLC conversion table.

Condition 13	Total sample volume (mL): 8.5	Vol nitrosamine (mL)	0.5	Vol TMB	1	TMB STOCK	TMB 05/10/2023	T (°C)	50
**Nitrosamine**	**Nitrosamine Area Response**	**TMB Area Response**	**Response Factor**	**[TMB] (mM)**	**[Nitrosamine if no rxn] (mM)**	**[Nitrosamine] (mM)**	**Conversion (%)**	**Average Conversion (%)**	**Std deviation (%)**
N1 1	13749.12	82467.55	0.17	3.52	6.62	5.32	19.60		
N1 2	14314.76	83137.06	0.17	3.52	6.62	5.50	17.03	18.31	1.29
N2 1	20978.84	25201.29	0.83	3.52	6.23	5.75	7.60		
N2 2	20915.15	25003.00	0.84	3.52	6.23	5.78	7.14	7.37	0.23
N3 1	20931.30	24797.43	0.84	3.52	6.24	5.56	10.95		
N3 2	20664.00	24739.71	0.84	3.52	6.24	5.50	11.88	11.42	0.46
N4 1		25079.71	0.00	3.52	5.89	0.05	99.16		
N4 2		24762.86	0.00	3.52	5.89	0.05	99.16	99.16	0.00
N5 1	5003.85	25035.07	0.20	3.52	5.92	1.68	71.67		
N5 2	6786.04	25394.16	0.27	3.52	5.92	2.21	62.62	67.14	4.53
N6 1		25580.74	0.00	3.52	6.56	-0.10	101.46		
N6 2		25340.78	0.00	3.52	6.56	-0.10	101.46	101.46	0.00
N7 1	19620.52	24890.59	0.79	3.52	6.37	5.94	6.70		
N7 2	20239.29	25361.23	0.80	3.52	6.37	6.02	5.48	6.09	0.61
N8 1	16327.27	25137.76	0.65	3.52	6.32	3.86	38.85		
N8 2	15765.09	25255.59	0.62	3.52	6.32	3.72	41.20	40.02	1.18

Figure S42: Condition 13 HPLC conversion table.

Condition 14	Total sample volume (mL): 8.5	Vol nitrosamine (mL)	0.5	Vol TMB	1	TMB STOCK	TMB 24/10/2023	T (°C)	50
**Nitrosamine**	**Nitrosamine Area Response**	**TMB Area Response**	**Response Factor**	**[TMB] (mM)**	**[Nitrosamine if no rxn] (mM)**	**[Nitrosamine] (mM)**	**Conversion (%)**	**Average Conversion (%)**	**Std deviation (%)**
N1 1	3311.13	82367.36	0.04	3.55	6.05	1.38	77.19		
N1 2	3266.40	85261.34	0.04	3.55	6.05	1.32	78.17	77.68	0.49
N2 1	10386.23	25543.43	0.41	3.55	6.43	2.73	57.58		
N2 2	10396.32	25322.12	0.41	3.55	6.43	2.76	57.14	57.36	0.22
N3 1	10563.92	25136.27	0.42	3.55	6.30	2.80	55.48		
N3 2	10291.79	25091.15	0.41	3.55	6.30	2.74	56.54	56.01	0.53
N4 1		25268.22	0.00	3.55	6.03	0.05	99.17		
N4 2		25401.78	0.00	3.55	6.03	0.05	99.17	99.17	0.00
N5 1	33.35	25167.93	0.00	3.55	7.25	0.10	98.66		
N5 2	27.44	25134.63	0.00	3.55	7.25	0.10	98.69	98.67	0.01
N6 1		25213.60	0.00	3.55	6.87	-0.10	101.40		
N6 2		25059.93	0.00	3.55	6.87	-0.10	101.40	101.40	0.00
N7 1	4125.79	25217.19	0.16	3.55	6.95	1.00	85.64		
N7 2	3565.98	25378.43	0.14	3.55	6.95	0.81	88.29	86.96	1.33
N8 1	27.93	25653.75	0.00	3.55	6.30	0.05	99.18		
N8 2	35.45	25834.11	0.00	3.55	6.30	0.05	99.16	99.17	0.01

Figure S43: Condition 14 HPLC conversion table.

Condition 15	Total sample volume (mL): 8.5	Vol nitrosamine (mL)	0.5	Vol TMB	1	TMB STOCK	TMB 06/11/2023	T (°C)	50
**Nitrosamine**	**Nitrosamine Area Response**	**TMB Area Response**	**Response Factor**	**[TMB] (mM)**	**[Nitrosamine if no rxn] (mM)**	**[Nitrosamine] (mM)**	**Conversion (%)**	**Average Conversion (%)**	**Std deviation (%)**
N1 1	12900.90	82637.95	0.16	3.56	6.08	5.04	17.07		
N1 2	12859.79	84235.26	0.15	3.56	6.08	4.93	18.87	17.97	0.90
N2 1	22432.62	25410.92	0.88	3.56	6.37	6.17	3.10		
N2 2	22757.23	24887.11	0.91	3.56	6.37	6.40	-0.49	1.30	1.79
N3 1	21217.75	25447.80	0.83	3.56	6.30	5.54	12.02		
N3 2	21139.10	25027.98	0.84	3.56	6.30	5.61	10.88	11.45	0.57
N4 1		25093.23	0.00	3.56	6.05	0.05	99.17		
N4 2		25286.60	0.00	3.56	6.05	0.05	99.17	99.17	0.00
N5 1	5495.49	25385.94	0.22	3.56	7.22	1.82	74.72		
N5 2	5536.19	25174.71	0.22	3.56	7.22	1.85	74.34	74.53	0.19
N6 1		25202.90	0.00	3.56	6.77	-0.10	101.43		
N6 2		25266.23	0.00	3.56	6.77	-0.10	101.43	101.43	0.00
N7 1	21806.48	25714.67	0.85	3.56	7.43	6.48	12.84		
N7 2	21343.73	25729.04	0.83	3.56	7.43	6.33	14.83	13.84	0.99
N8 1	17496.57	25452.26	0.69	3.56	6.12	4.12	32.60		
N8 2	17851.55	25612.18	0.70	3.56	6.12	4.18	31.68	32.14	0.46

Figure S44: Condition 15 HPLC conversion table.

Condition 16	Total sample volume (mL): 8.5	Vol nitrosamine (mL)	0.5	Vol TMB	1	TMB STOCK	TMB 24/10/2023	T (°C)	50
**Nitrosamine**	**Nitrosamine Area Response**	**TMB Area Response**	**Response Factor**	**[TMB] (mM)**	**[Nitrosamine if no rxn] (mM)**	**[Nitrosamine] (mM)**	**Conversion (%)**	**Average Conversion (%)**	**Std deviation (%)**
N1 1	12588.83	82812.03	0.15	3.55	6.05	4.91	18.87		
N1 2	13006.84	82968.40	0.16	3.55	6.05	5.06	16.39	17.63	1.24
N2 1	22260.67	25355.42	0.88	3.55	6.43	6.13	4.65		
N2 2	23510.50	25254.33	0.93	3.55	6.43	6.51	-1.30	1.67	2.98
N3 1	20958.67	25150.87	0.83	3.55	6.30	5.53	12.12		
N3 2	20886.71	24947.43	0.84	3.55	6.30	5.56	11.71	11.91	0.21
N4 1	489.50	24861.96	0.02	3.55	6.03	0.09	98.43		
N4 2	1952.39	24653.19	0.08	3.55	6.03	0.23	96.19	97.31	1.12
N5 1	11963.99	25401.95	0.47	3.55	7.25	3.87	46.71		
N5 2	12664.38	24708.51	0.51	3.55	7.25	4.20	42.11	44.41	2.30
N6 1		25226.47	0.00	3.55	6.87	-0.10	101.40		
N6 2		25026.18	0.00	3.55	6.87	-0.10	101.40	101.40	0.00
N7 1	23718.46	25597.71	0.93	3.55	6.95	7.10	-2.10		
N7 2	24532.43	25649.30	0.96	3.55	6.95	7.34	-5.54	-3.82	1.72
N8 1	22371.51	25981.97	0.86	3.55	6.30	5.15	18.21		
N8 2	23046.61	26130.04	0.88	3.55	6.30	5.28	16.24	17.23	0.99

Figure S45: Condition 16 HPLC conversion table.

Condition 17	Total sample volume (mL): 8.5	Vol nitrosamine (mL)	0.5	T (°C)	22	
**Nitrosamine**	**Nitrosamine Area Response**	**[Nitrosamine if no rxn] (mM)**	**[Nitrosamine] (mM)**	**Conversion (%)**	**Average Conversion (%)**	**Std deviation (%)**
N1 1		6.92	-0.39	105.61		
N1 2		6.92	-0.39	105.61	105.61	0.00
N2 1		6.76	-0.29	104.28		
N2 2		6.76	-0.29	104.28	104.28	0.00
N3 1		6.16	-0.26	104.24		
N3 2		6.16	-0.26	104.24	104.24	0.00
N4 1	1234.81	5.93	0.27	95.43		
N4 2	1299.46	5.93	0.28	95.34	95.39	0.05
N5 1		6.81	0.29	95.80		
N5 2		6.81	0.29	95.80	95.80	0.00
N6 1		7.94	-0.14	101.74		
N6 2		7.94	-0.14	101.74	101.74	0.00
N7 1		6.55	-0.43	106.51		
N7 2		6.55	-0.43	106.51	106.51	0.00
N8 1		6.75	-0.07	101.08		
N8 2		6.75	-0.07	101.08	101.08	0.00

Figure S46: Condition 17 HPLC conversion table.

Condition 18	Total sample volume (mL): 5.5	Vol nitrosamine (mL)	0.5	Vol TMB	1	TMB STOCK	TMB 09/08/2023	T (°C)	22
**Nitrosamine**	**Nitrosamine Area Response**	**TMB Area Response**	**Response Factor**	**[TMB] (mM)**	**[Nitrosamine if no rxn] (mM)**	**[Nitrosamine] (mM)**	**Conversion (%)**	**Average Conversion (%)**	**Std deviation (%)**
N1 1	21391.52	116745.97	0.18	5.45	9.09	9.03	0.66		
N1 2	20978.86	118261.92	0.18	5.45	9.09	8.75	3.77	4.44	3.39
N1 3	20281.74	120883.30	0.17	5.45	9.09	8.29	8.88		
N2 1	34968.59	38872.56	0.90	5.45	9.15	9.64	-5.41		
N2 2	33054.70	38648.92	0.86	5.45	9.15	9.15	-0.05	-1.83	2.54
N2 3	32641.80	38177.84	0.85	5.45	9.15	9.15	-0.02		
N3 1	31274.86	38482.57	0.81	5.45	9.16	8.28	9.63		
N3 2	32745.30	38355.86	0.85	5.45	9.16	8.69	5.09	6.48	2.23
N3 3	32740.89	38204.05	0.86	5.45	9.16	8.73	4.72		
N4 1	71582.76	38015.53	1.88	5.45	9.17	7.16	21.92		
N4 2	73008.28	37619.86	1.94	5.45	9.17	7.39	19.35	22.18	2.42
N4 3	68774.65	38044.11	1.81	5.45	9.17	6.85	25.26		
N5 1	29184.31	38402.37	0.76	5.45	9.14	9.48	-3.73		
N5 2	28735.59	37942.74	0.76	5.45	9.14	9.45	-3.37	-2.24	1.86
N5 3	26990.58	37005.16	0.73	5.45	9.14	9.11	0.39		
N6 1		37758.74	0.00	5.45	9.31	-0.15	101.59		
N6 2		37911.24	0.00	5.45	9.31	-0.15	101.59	101.59	0.00
N6 3		38952.38	0.00	5.45	9.31	-0.15	101.59		
N7 1	32460.24	38109.23	0.85	5.45	9.13	9.97	-9.19		
N7 2	30365.50	38173.93	0.80	5.45	9.13	9.28	-1.63	-6.04	3.21
N7 3	31861.62	38033.28	0.84	5.45	9.13	9.80	-7.31		
N8 1	37180.70	37799.96	0.98	5.45	9.53	9.01	5.43		
N8 2	38891.93	37983.10	1.02	5.45	9.53	9.38	1.58	2.74	1.90
N8 3	39172.20	38114.40	1.03	5.45	9.53	9.42	1.22		

Figure S47: Condition 18 HPLC conversion table.

Condition 19	Total sample volume (mL): 8	Vol nitrosamine (mL)	0.5	Vol TMB	1	TMB STOCK	TMB 07/12/2023	T (°C)	50
Nitrosamine	Nitrosamine Area Response	TMB Area Response	Response Factor	[TMB] (mM)	[Nitrosamine if no rxn] (mM)	[Nitrosamine] (mM)	Conversion (%)	Average Conversion (%)	Std deviation (%)
N1 1	32750.73	28419.24	1.15	3.74	7.35	7.21	1.94		
N1 2	32753.00	28291.32	1.16	3.74	7.35	7.24	1.47	1.70	0.23
N2 1	27475.89	28545.74	0.96	3.74	7.18	7.10	1.13		
N2 2	27127.70	28225.36	0.96	3.74	7.18	7.09	1.28	1.21	0.07
N3 1	25198.00	28858.50	0.87	3.74	6.55	6.11	6.75		
N3 2	25425.44	28985.59	0.88	3.74	6.55	6.13	6.33	6.54	0.21
N4 1	13799.87	22078.56	0.63	3.74	6.30	1.58	74.89		
N4 2	13686.71	21835.54	0.63	3.74	6.30	1.59	74.82	74.86	0.04
N5 1	23158.56	27556.34	0.84	3.74	7.24	7.19	0.62		
N5 2	23168.75	27576.00	0.84	3.74	7.24	7.19	0.65	0.63	0.01
N6 1		27900.08	0.00	3.74	8.43	-0.10	101.20		
N6 2		28236.37	0.00	3.74	8.43	-0.10	101.20	101.20	0.00
N7 1	24544.41	27647.38	0.89	3.74	6.96	7.15	-2.78		
N7 2	24473.56	27627.38	0.89	3.74	6.96	7.14	-2.55	-2.66	0.12
N8 1	31751.67	27629.48	1.15	3.74	7.17	7.23	-0.73		
N8 2	31467.96	27249.12	1.15	3.74	7.17	7.26	-1.22	-0.97	0.25

Figure S48: Condition 19 HPLC conversion table.

Condition 20	Total sample volume (mL): 4	Vol nitrosamine (mL)	1	Vol TMB	1	TMB STOCK	TMB 07/12/2023	T (°C)	22
**Nitrosamine**	**Nitrosamine Area Response**	**TMB Area Response**	**Response Factor**	**[TMB] (mM)**	**[Nitrosamine if no rxn] (mM)**	**[Nitrosamine] (mM)**	**Conversion (%)**	**Average Conversion (%)**	**Std deviation (%)**
N1 1	63617.49	147191.96	0.43	7.49	29.40	28.95	1.51		
N1 2	63494.62	143198.63	0.44	7.49	29.40	29.70	-1.02	0.25	1.27
N2 1	102337.20	49876.93	2.05	7.49	28.73	30.77	-7.11		
N2 2	101701.32	51575.75	1.97	7.49	28.73	29.55	-2.88	-5.00	2.12
N3 1	91877.38	50672.85	1.81	7.49	26.19	25.30	3.40		
N3 2	92547.58	50938.68	1.82	7.49	26.19	25.35	3.21	3.31	0.10
N4 1	215019.56	52533.88	4.09	7.49	25.20	23.69	5.97		
N4 2	230999.34	51908.80	4.45	7.49	25.20	26.27	-4.24	0.86	5.10
N5 1	83196.86	51381.82	1.62	7.49	28.96	27.55	4.85		
N5 2	88219.63	49865.50	1.77	7.49	28.96	30.09	-3.91	0.47	4.38
N6 1		51432.09	0.00	7.49	33.74	-0.20	100.60		
N6 2		50804.67	0.00	7.49	33.74	-0.20	100.60	100.60	0.00
N7 1	83021.16	53456.53	1.55	7.49	27.84	25.51	8.34		
N7 2	87626.19	54091.50	1.62	7.49	27.84	26.64	4.29	6.32	2.02
N8 1	117313.13	53030.94	2.21	7.49	28.69	27.73	3.36		
N8 2	112758.38	54189.73	2.08	7.49	28.69	26.09	9.08	6.22	2.86

Figure S49: Condition 20 HPLC conversion table.

Condition 21	Total sample volume (mL): 10	Vol nitrosamine (mL)	1	Vol TMB	1	TMB STOCK	TMB 07/12/2023	T (℃)	22
**Nitrosamine**	**Nitrosamine Area Response**	**TMB Area Response**	**Response Factor**	**[TMB] (mM)**	**[Nitrosamine if no rxn] (mM)**	**[Nitrosamine] (mM)**	**Conversion (%)**	**Average Conversion (%)**	**Std deviation (%)**
N1 1	52903.16	21457.65	2.47	3.74	14.70	15.66	-6.53		
N1 2	53137.06	21500.30	2.47	3.74	14.70	15.70	-6.79	-6.66	0.13
N2 1	35432.90	18703.96	1.89	2.99	11.49	11.35	1.22		
N2 2	34991.05	18431.30	1.90	2.99	11.49	11.38	1.01	1.12	0.11
N3 1	32813.13	18724.64	1.75	2.99	10.48	9.78	6.63		
N3 2	32584.19	18311.48	1.78	2.99	10.48	9.93	5.20	5.91	0.72
N4 1	79069.93	17942.41	4.41	2.99	10.08	10.38	-2.98		
N4 2	79994.83	18080.16	4.42	2.99	10.08	10.43	-3.49	-3.24	0.26
N5 1	30923.65	18673.54	1.66	2.99	11.58	11.27	2.70		
N5 2	30813.85	18595.18	1.66	2.99	11.58	11.28	2.63	2.67	0.03
N6 1		18950.87	0.00	2.99	13.50	-0.08	100.60		
N6 2		18866.48	0.00	2.99	13.50	-0.08	100.60	100.60	0.00
N7 1	30982.31	18604.79	1.67	2.99	11.13	10.96	1.55		
N7 2	30783.73	18631.07	1.65	2.99	11.13	10.87	2.34	1.95	0.39
N8 1	39818.28	18670.83	2.13	2.99	11.48	10.70	6.82		
N8 2	40376.86	18961.90	2.13	2.99	11.48	10.68	6.96	6.89	0.07

Figure S50: Condition 21 HPLC conversion table.

4.3 Conversion summary table

(a) Commercial NSAs in this study

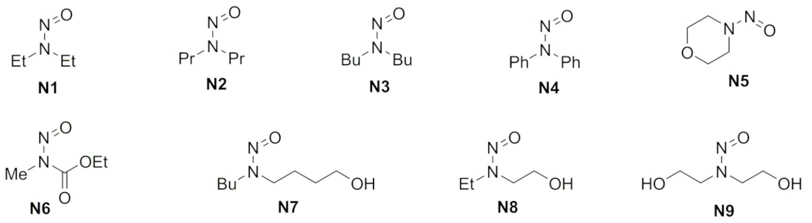

(b) Conversions of N1-8 against common reagents in organic syntheses

Conversion (%) 0 – 100

No.	Class	Reagents	Solvent	Temp. (°C)	Reaction time (h)	N1	N2	N3	N4	N5	N6	N7	N8
1	Oxidant	H_2O_2 2M	H_2O/EtOH	22	24	2	-3	4	17	0	2	2	5
2	Oxidant	CH_3CO_3H	EtOH	22	24	8	8	7	95	14	101	14	12
3	Acid	HBr	AcOH/EtOH	22	24	6	7	13	42	25	85	2	26
4	Acid	HCl 2M	H_2O/EtOH	22	24	2	-5	6	37	-1	101	-12	6
5	Acid	HCl 2M	H_2O/EtOH	50	16	1	-4	13	86	5	101	-14	6
6	Acid	HCl 2M	EtOH	22	24	-2	-2	3	100	56	101	-6	15
7	Acid	HCl 2M	EtOH	50	16	71	54	56	99	98	101	52	100
8	Reductant	$NaBH_4$ 16 eq	EtOH	22	24	3	0	3	23	-2	101	5	0
9	Reductant	DiBAL-H 5 eq.	THF/Hexane	22	0.5	72	66	61	76	101	100	51	69
10	Reductant	DiBAL-H 5 eq.	THF/Hexane	22	1	85	86	75	85	101	100	90	101
11	Reductant	$Na_2S_2O_4$	H_2O/EtOH	22	24	9	3	9	87	25	63	-5	5
12	Reductant	$Na_2S_2O_4$ + NaOH 0.1M	H_2O/EtOH	22	24	-4	2	9	87	32	100	-9	2
13	Reductant	$Na_2S_2O_4$ + NaOH 0.1M	H_2O/EtOH	50	16	18	7	11	99	67	101	6	40
14	Reductant	$Na_2S_2O_4$ + NaOH 1M	H_2O/EtOH	50	16	78	57	56	99	99	101	87	99
15	Reductant	$Na_2S_2O_4$ + NaOH 1M	H_2O/EtOH	50	3	18	1	11	99	75	101	14	32
16	Reductant	$Na_2S_2O_4$ + NaOH 1M	H_2O/EtOH	50	1	18	2	12	97	44	101	-4	17
17	Reductant	$Na_2S_2O_4$ + 20% NaOH	H_2O/EtOH	50	16	106	104	104	95	96	100	107	101
18	Reductant	Na_2SO_3	H_2O/EtOH	22	24	4	-2	6	22	-2	102	-6	3
19	Reductant	Na_2SO_3 + 1M NaOH	H_2O/EtOH	50	16	2	1	7	75	1	101	-3	-1
20	Base	NaOH 2M	H_2O/EtOH	22	24	0	-5	3	1	0	101	6	6
21	Base	NaOEt 21% w/v	EtOH	22	24	-7	1	6	-3	3	101	2	7

Reaction conversions were determined by HPLC

Figure S51: Conversions summary table for the 21 reaction conditions.

5 Calibrations

5.1 HPLC Calibrations

5.1.1 HPLC method

The HPLC analysis of *N*-nitrosamine conversion was performed on an Agilent 1260 HPLC. The HPLC method used in all experiments is as follows: Solvents: A) MeCN 0.1% TFA, B) H_2O 0.1% TFA. Flow rate = 1.5 mL/min. Polarity gradient: 0 mins; Solvent A 5%, Solvent B 95% 5 mins; Solvent A 95%, Solvent B 5%. Injection volume 1 μL. Detector: DAD 210 nm & DAD 220 nm. Column: Ascentis Express C18, 2.7 Micron HPLC Column, 2.7 mum particle size, L x I.D 5 cm x 4.6 mm. Calibrations were performed for the 8 *N*-nitrosamines in the concentration range of 40-0.625 mM. All reactions were analysed within the calibration limits to avoid extrapolation. Both calibrations including and excluding the internal standard were performed. This was because the chosen internal standard (1,3,5-trimethoxybenzene) was unstable to acid, and therefore the absolute peak area for the nitrosamines was used to calculate the conversions for reactions involving acid as mentioned earlier. Table S3 gives the parameters of the exponential fits for *N*-nitrosodiphenylamine and Table S4 gives the parameters of the linear fits for the remaining *N*-nitrosamines. The calibration curves themselves are shown in figures S52 to S60.

5.1.2 HPLC calibration curve equations

Table S3: Exponential HPLC calibration curve equations for *N*-nitrosodiphenylamine.

**Calibration curve data**	**λ (nm)**	**y0**	**A**	**R0**	**r^2**
N-nitrosodiphenylamine (IS)	220	12.65281	-12.67495	-0.12402	0.9992
N-nitrosodiphenylamine (No IS)	220	496368.91381	-498165.74319	-0.02255	0.9993

Table S4: Linear HPLC calibration curve equations for remaining *N*-nitrosamines.

**Calibration curve data**	**λ (nm)**	**gradient**	**intercept**	**r^2**
N-nitrosodiethylamine (IS)	210	0.11267	-0.00354	0.9996
N-nitrosodipropylamine (IS)	220	0.49214	0.02893	0.9984
N-nitrosodibutylamine (IS)	220	0.53761	-0.00385	0.9991
N-ethyl-*N*-(2-hydroxyethyl)nitrosamine (IS)	220	0.59921	-0.00759	0.9996
N-butyl-*N*-(2-hydroxybutyl)nitrosamine (IS)	220	0.44435	0.03870	0.9993
N-methyl-*N*-nitrosourethane (IS)	220	0.42030	0.01140	0.9998
N-nitrosomorpholine (IS)	220	0.44284	-0.01077	0.9989
Diphenylamine 0-20mM (IS)	220	0.75369	0.01476	0.9983
N-nitrosodiethylamine (No IS)	210	2269.40863	-355.84068	0.9997
N-nitrosodipropylamine (No IS)	220	3522.83772	1019.94959	0.9989
N-nitrosodibutylamine (No IS)	220	3476.73713	909.21290	0.9995
N-ethyl-*N*-(2-hydroxyethyl)nitrosamine (No IS)	220	4189.37773	305.43737	0.9999
N-butyl-*N*-(2-hydroxybutyl)nitrosamine (No IS)	220	3193.89269	1361.67662	0.9996
N-methyl-*N*-nitrosourethane (No IS)	220	2868.71231	395.65576	0.9998
N-nitrosomorpholine (No IS)	220	3236.75029	-925.92477	0.9994

5.1.3 HPLC calibration curves

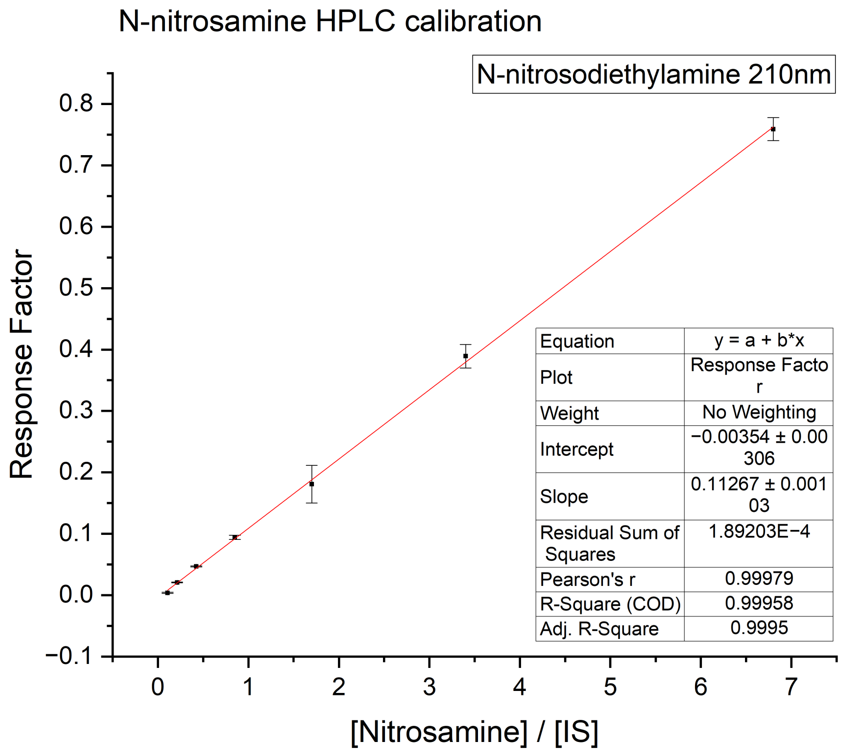

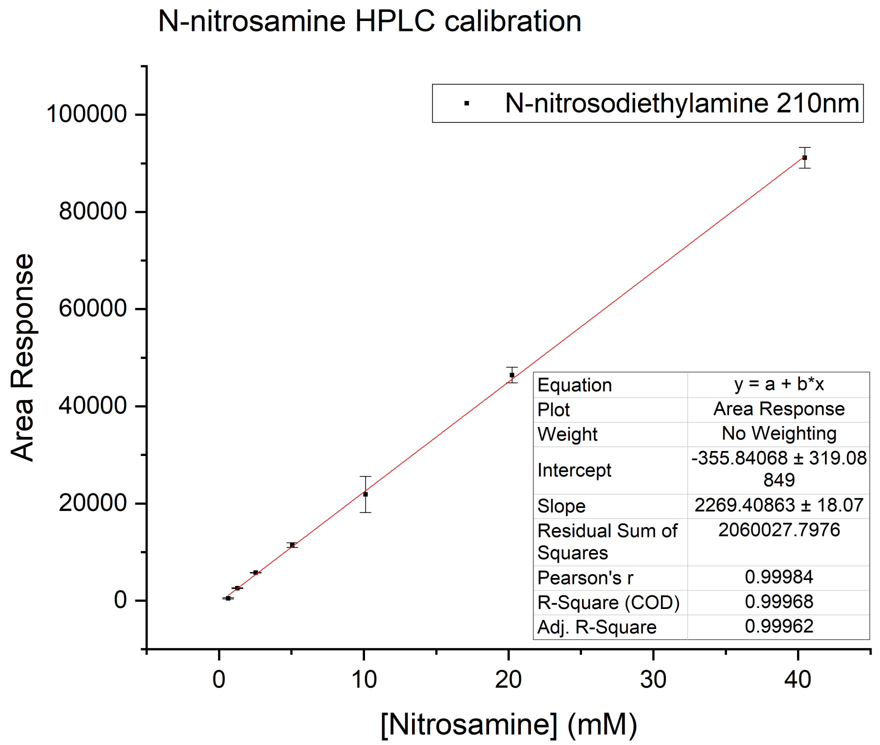

Figure S52: HPLC Calibration curves for N-nitrosodiethylamine (N1) at 210nm.

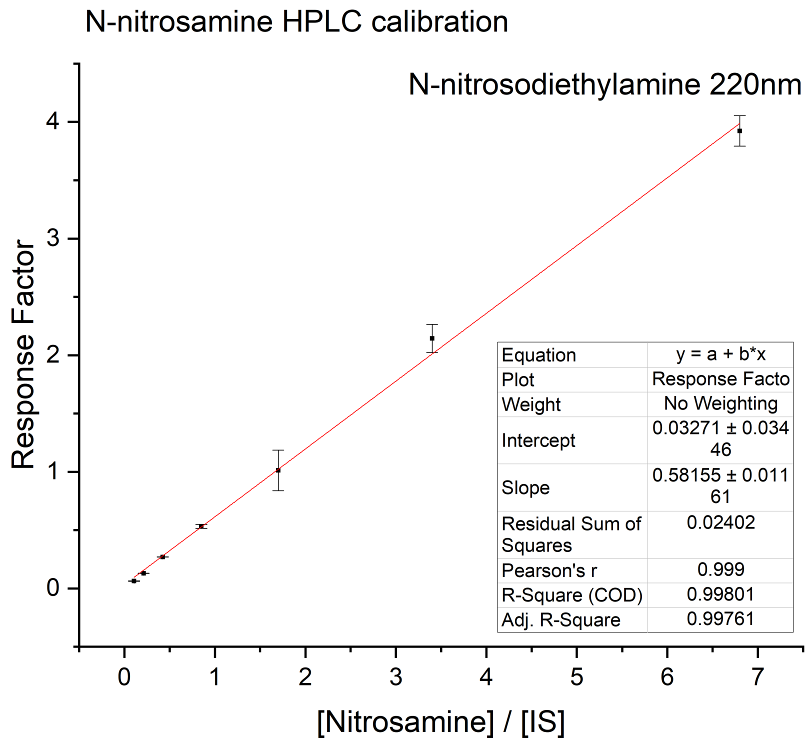

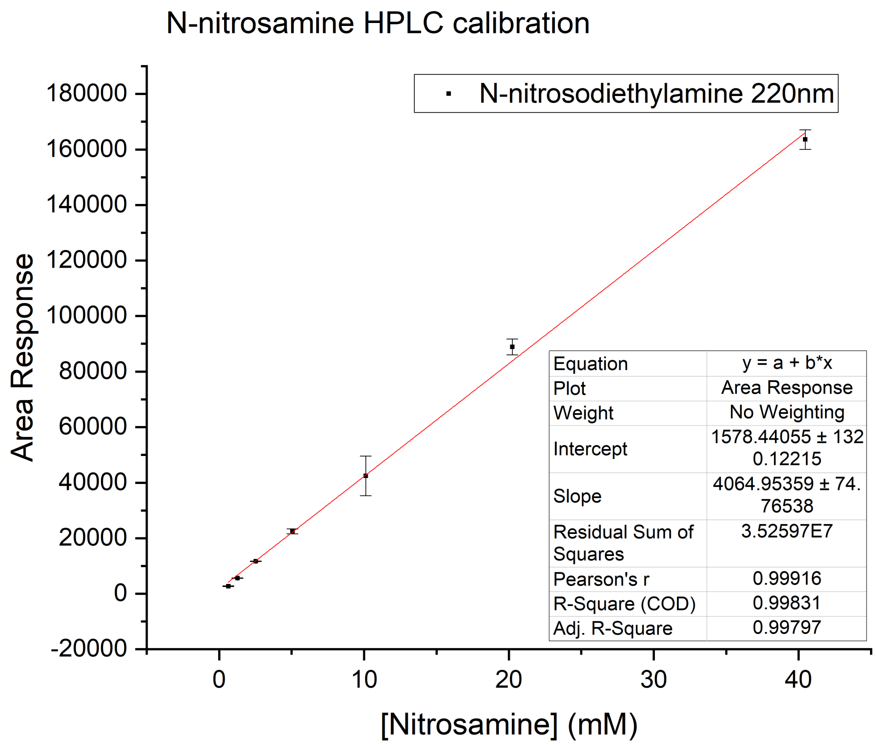

Figure S53: HPLC Calibration curves for *N*-nitrosodiethylamine (N1) at 220nm.

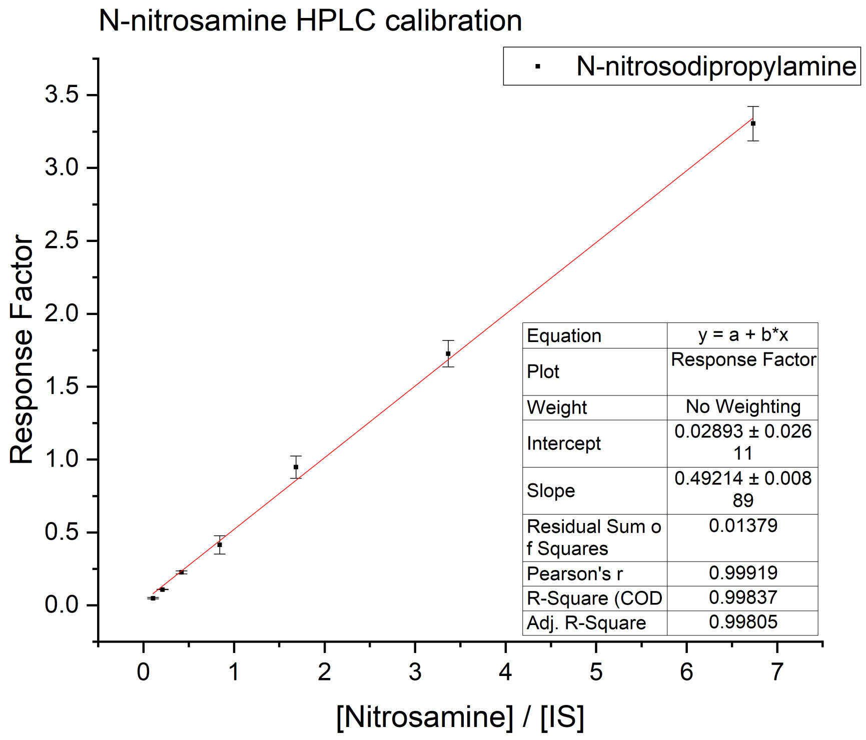

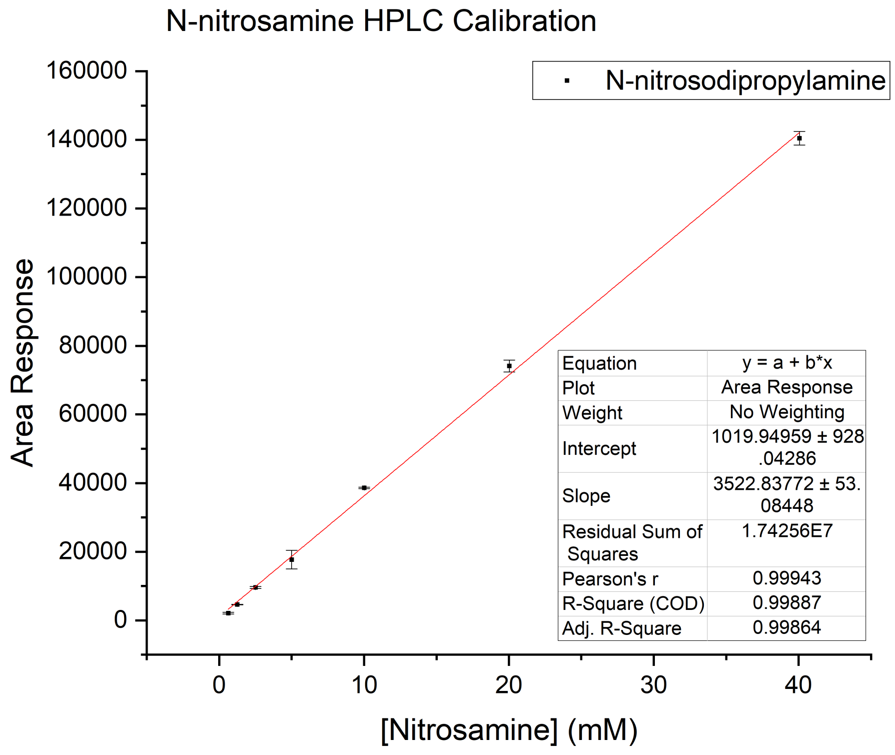

Figure S54: HPLC Calibration curves for *N*-nitrosodipropylamine (N2).

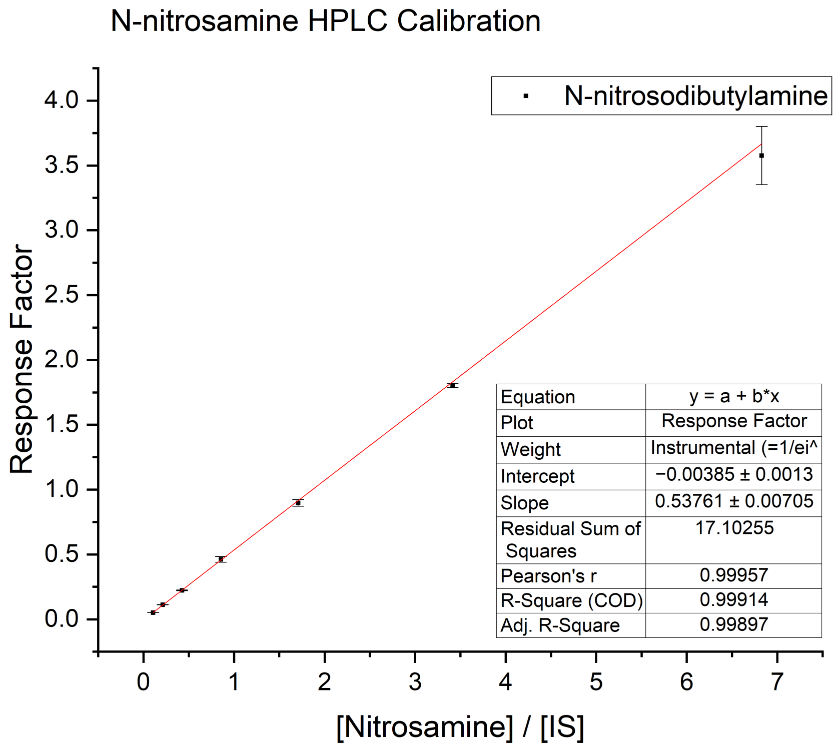

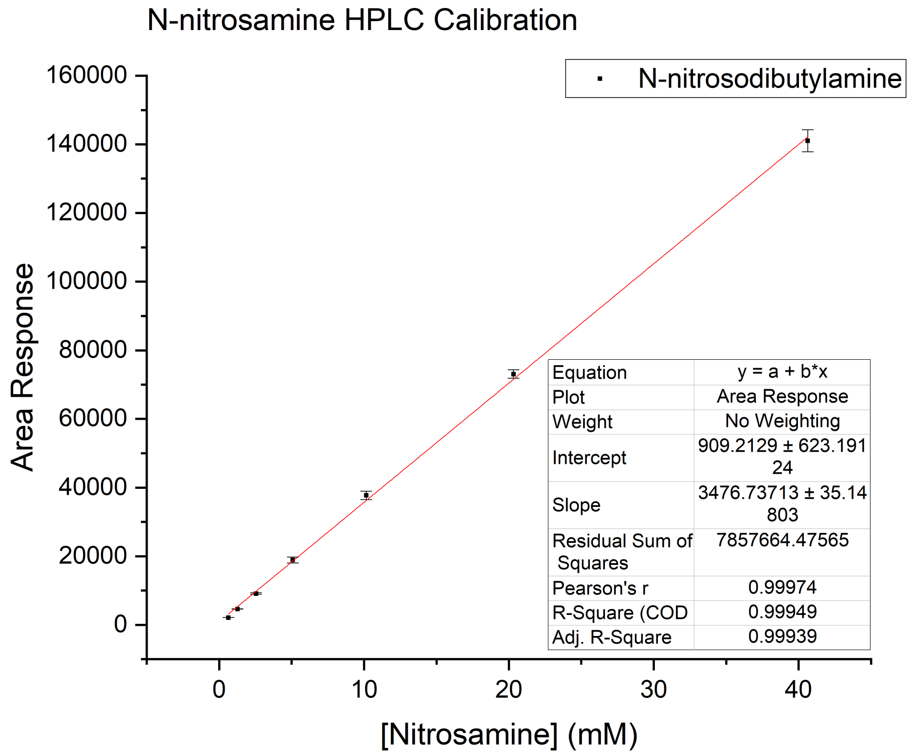

Figure S55: HPLC Calibration curves for *N*-nitrosodibutylamine (N3).

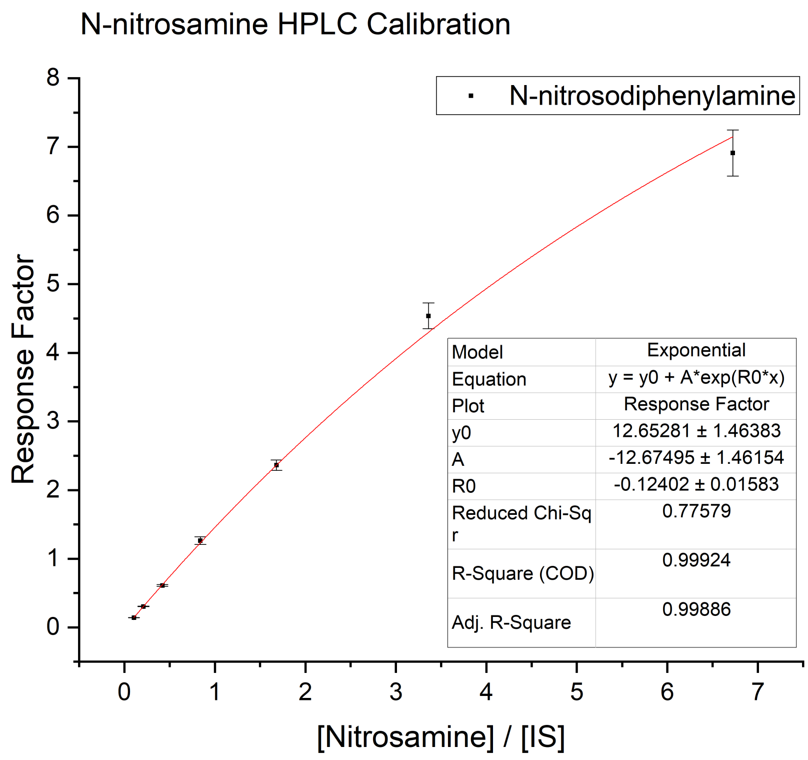

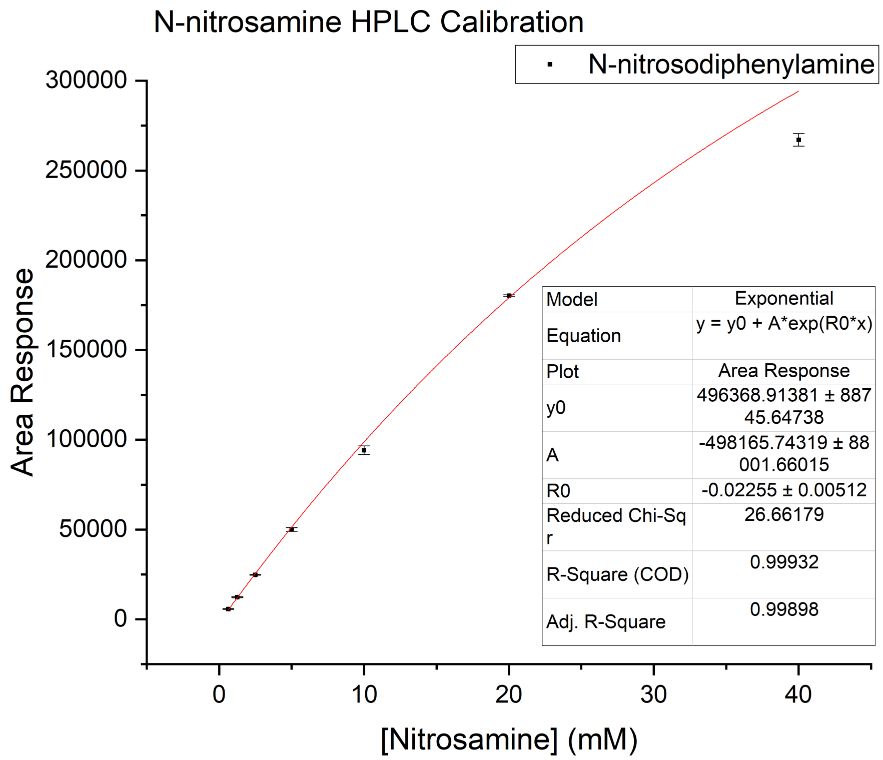

Figure S56: HPLC Calibration curves for *N*-nitrosodiphenylamine (N4).

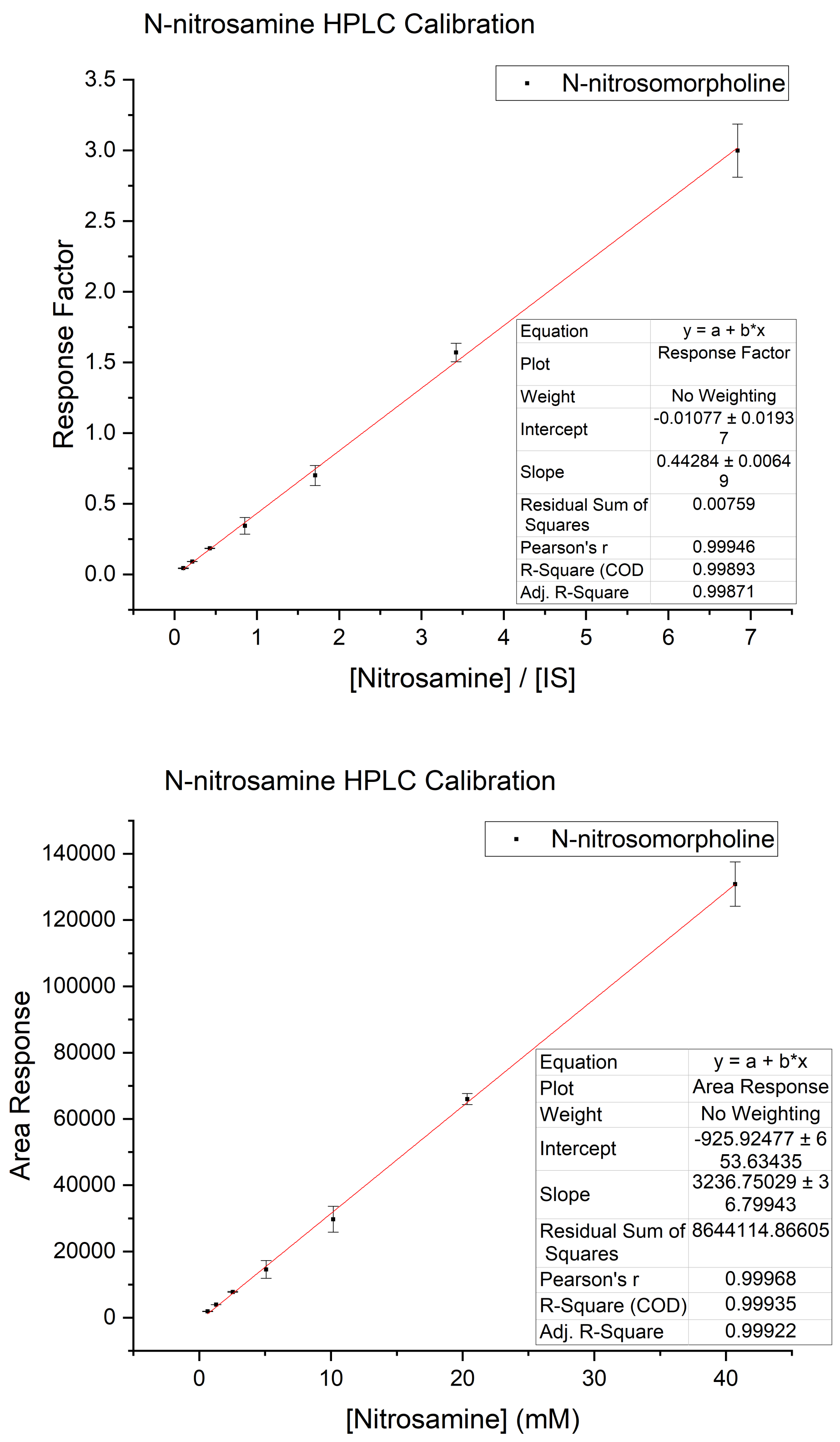

Figure S57: HPLC Calibration curves for *N*-nitrosomorpholine (N5).

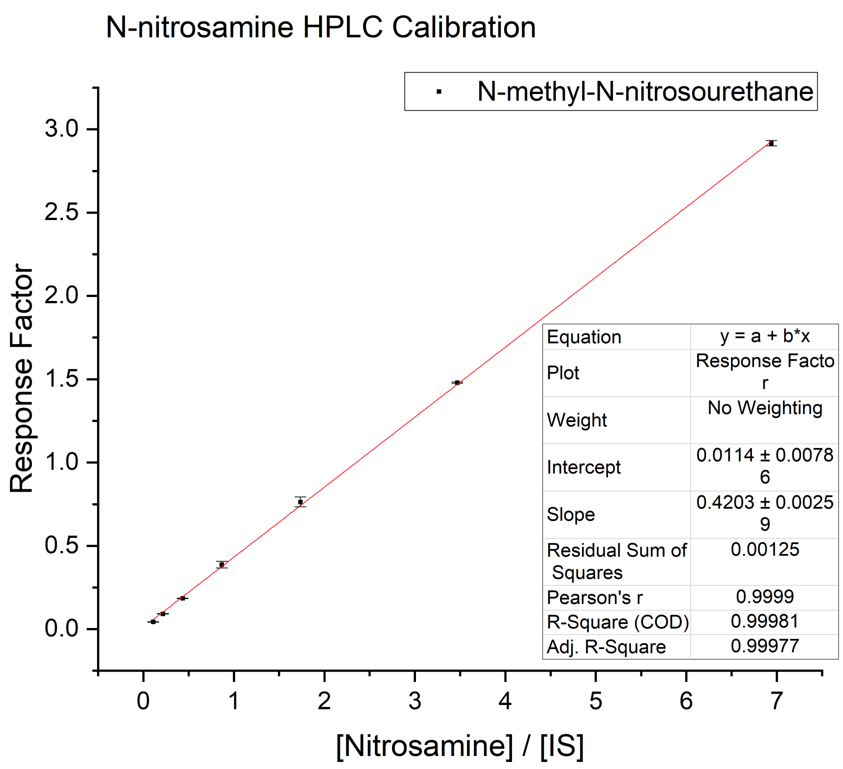

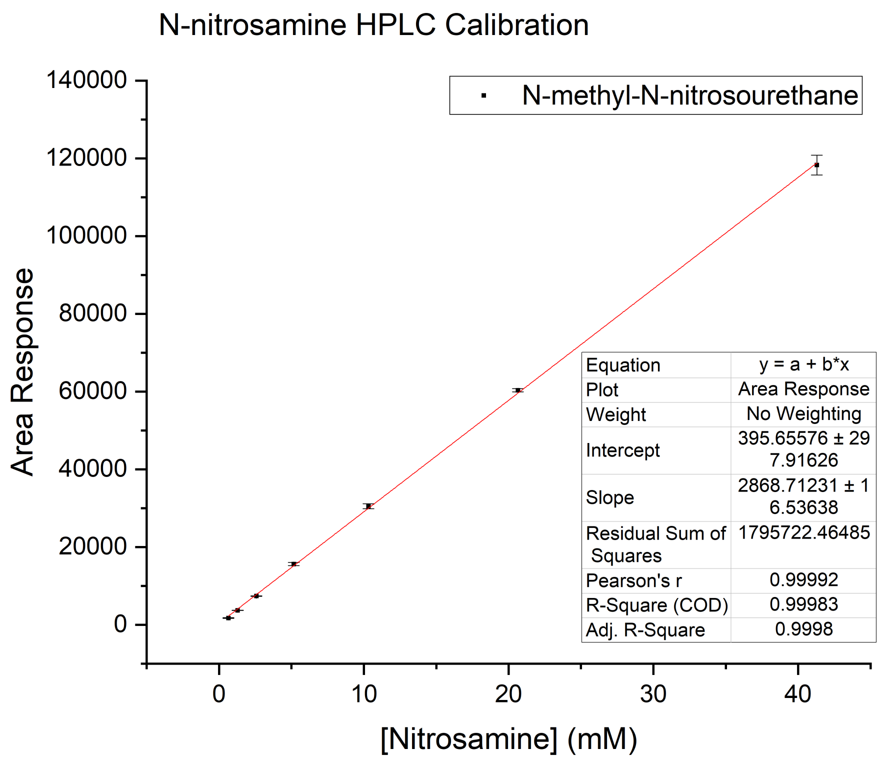

Figure S58: HPLC Calibration curves for N-methyl-N-nitrosourethane (N6).

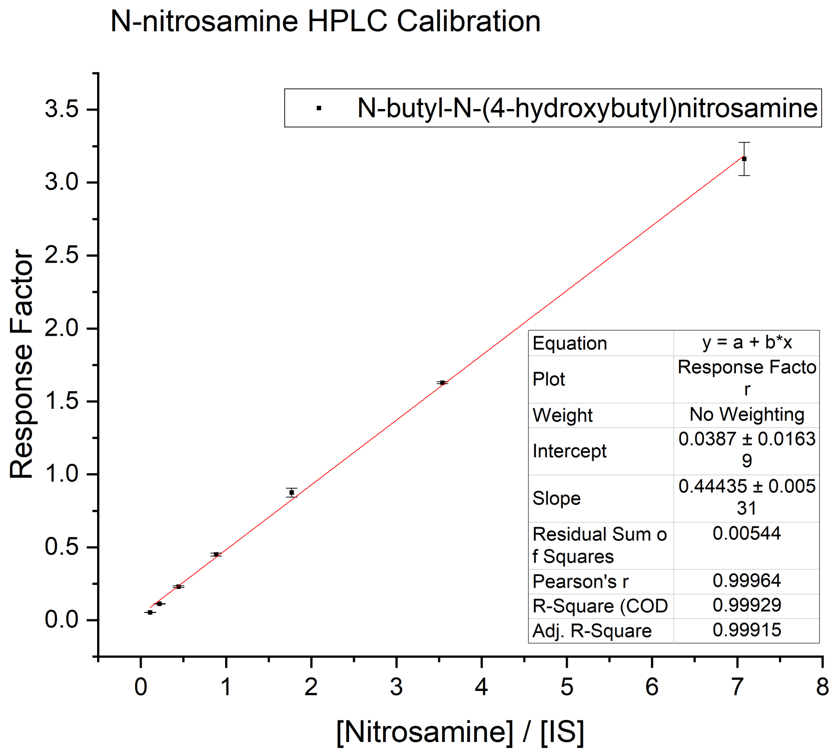

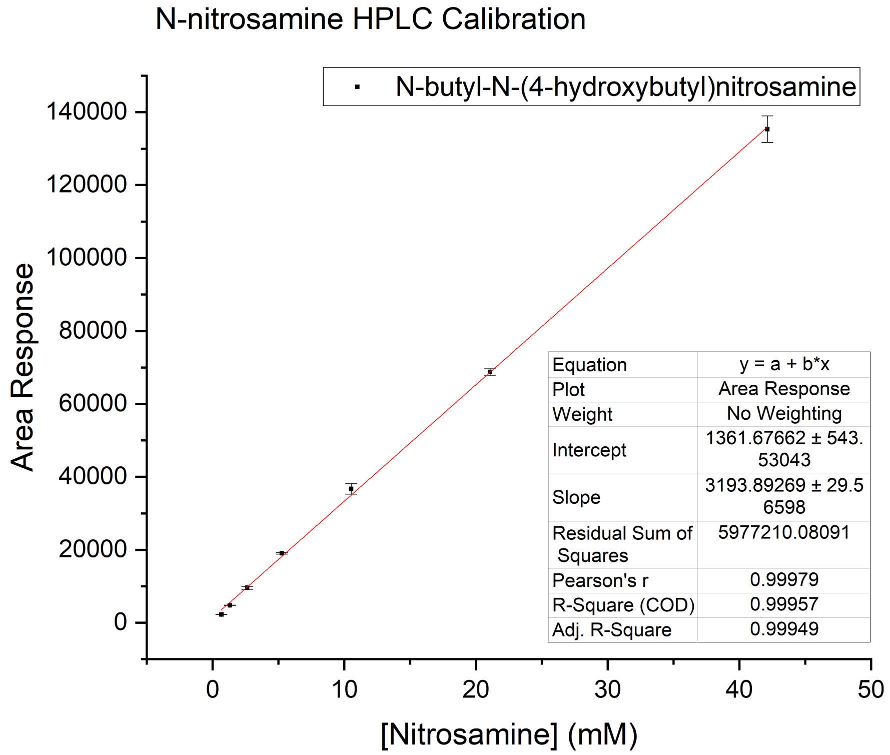

Figure S59: HPLC Calibration curves for *N*-butyl-*N*-(4-hydroxybutyl)nitrosamine (N7).

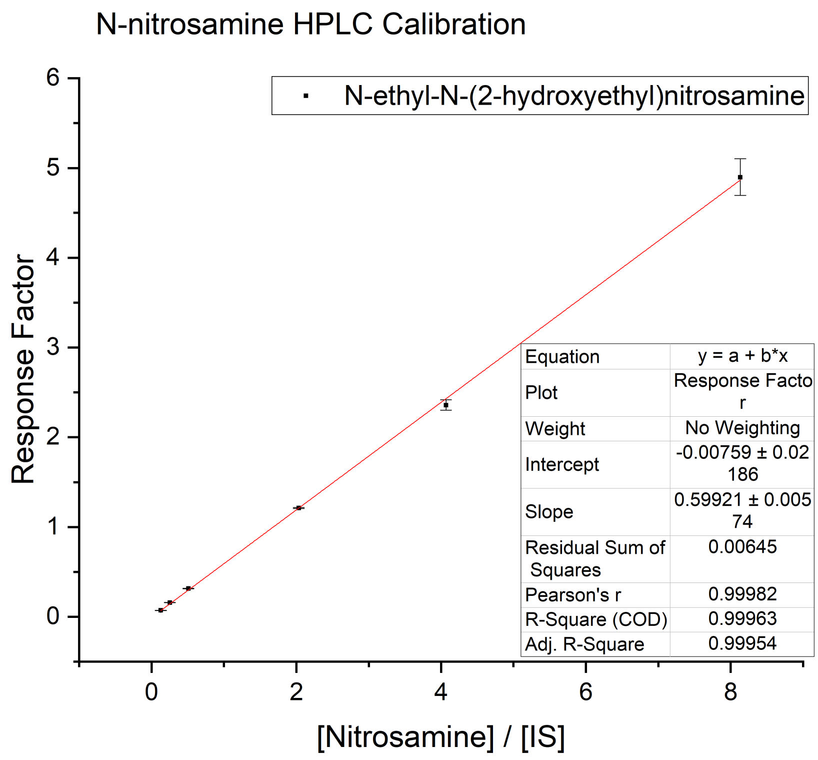

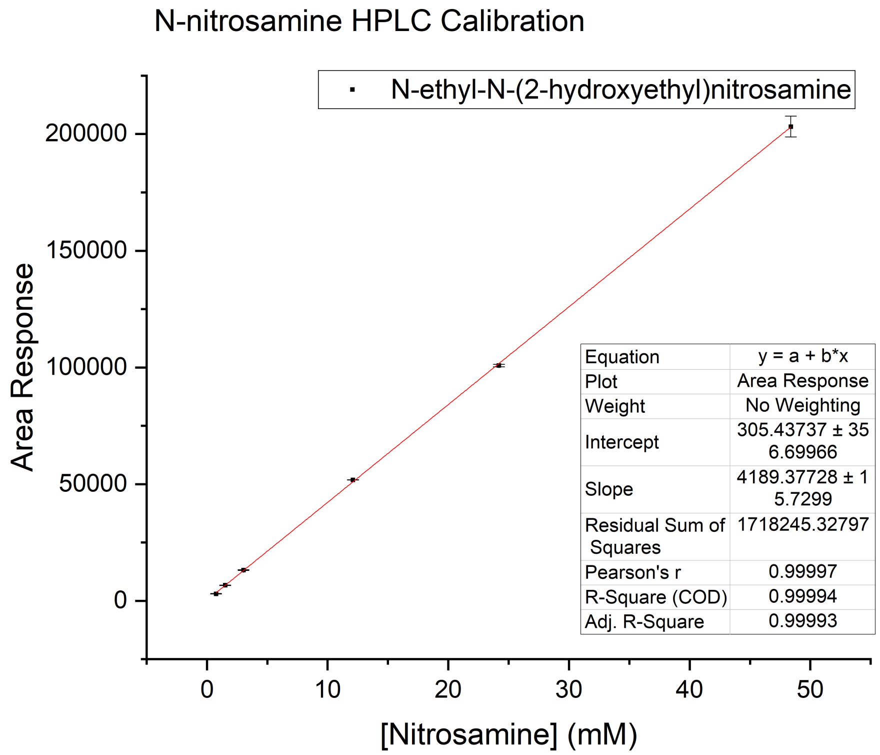

Figure S60: HPLC Calibration curves for N-ethyl-N-(2-hydroxyethyl)nitrosamine (N8).

5.2 GC Calibrations

For conditions 9 and 10, reaction conversions were monitored using a gas chromatography with tandem mass spectrometry instrument. Specifically, an Agilent 8890 GC system with 7000E GC/TQ (triple quad mass spec) with hydroinert EI (Electrospray ionisation) 350 source using H_2 as a carrier gas and a DB-5MS UI, 20m x 0.180 mm, 0.18 um column. The line equations of the calibrations are summarised in Table S5. The GC-MS/MS calibrations were performed in the same manner as for the HPLC calibrations described in section 5.1, with TMB as an internal standard. The calibration plots can be found in Figures S61 to S67.

Table S5: The determined calibration curve equations for the nitrosamines analysed by GC for conditions 9 and 10.

**Nitrosamine (ref)**	**fitting**	**a**	**b**	**y0**	**A**	**R0**	**r^2**
N1 (figure S61)	y = a + b*x	0.00189	0.05604	-	-	-	0.99771
N2 (figure S62)	y = y0 + A*exp(R0*x)	-	-	1.13987	-1.13919	-0.17267	0.99489
N3 (figure S63)	y = y0 + A*exp(R0*x)	-	-	2.33399	-2.33784	-0.16082	0.99653
N5 (figure S64)	y = y0 + A*exp(R0*x)	-	-	0.83249	-0.82662	-0.09051	0.99297
N6 (figure S65)	y = a + b*x	-0.00172	0.01453	-	-	-	0.99691
N7 (figure S66)	y = a + b*x	-0.00118	0.24806	-	-	-	0.99422
N8 (figure S67)	y = y0 + A*exp(R0*x)	-	-	0.19386	-0.19154	-0.22543	0.97999

5.2.1 GC methods

The GC-MS/MS method settings are listed in Table S6:

Table S6: Gas chromatography settings.

**Inlet**		
Mode	Split	
Split Ratio	25:1	
Septum Purge Flow	3 mL/min	
Heater	250 °C	
Pressure	7.9 psi	
Gas Saver	20 mL/min	
**Injector**		
Injection Volume	1 μL	
Solvent Washes	Acetone, MeOH	
**Oven Programme**		
t (mins)	T (°C) (rate °C/min)	Hold Time (mins)
0	50	3
3	80 (20ºC/min)	2
6.5	100 (5°C/min)	2
12.5	280(35°C/min)	2.36
**Post Run**		
t (mins)	T (°C)	Hold Time (mins)
20	325	20

5.2.2 GC calibration curves

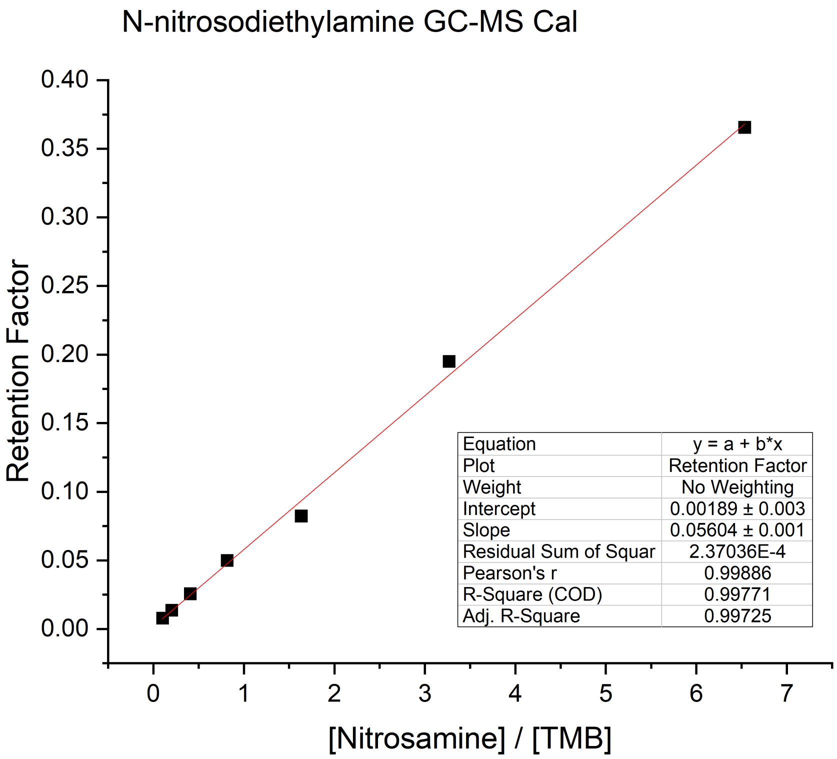

Figure S61: HPLC Calibration curves for N-nitrosodiethylamine (N1).

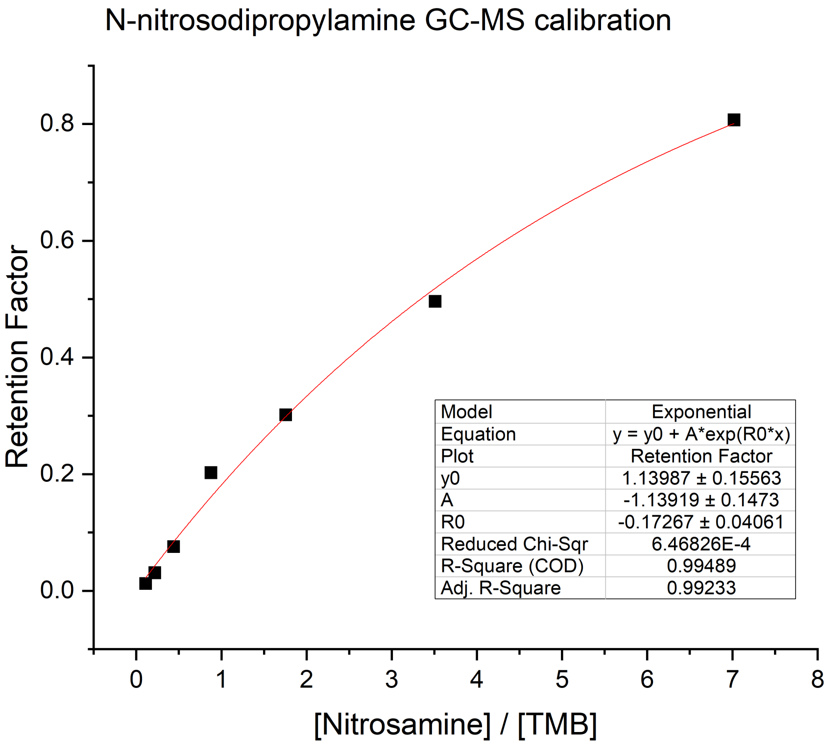

Figure S62: HPLC Calibration curves for N-nitrosodipropylamine (N2).

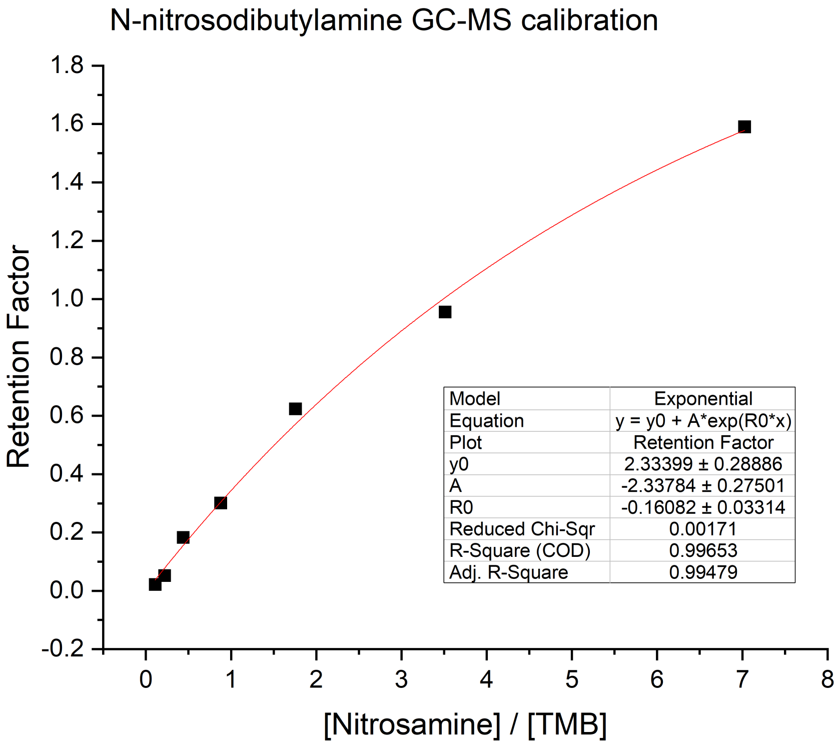

Figure S63: HPLC Calibration curves for N-nitrosodibutylamine (N3).

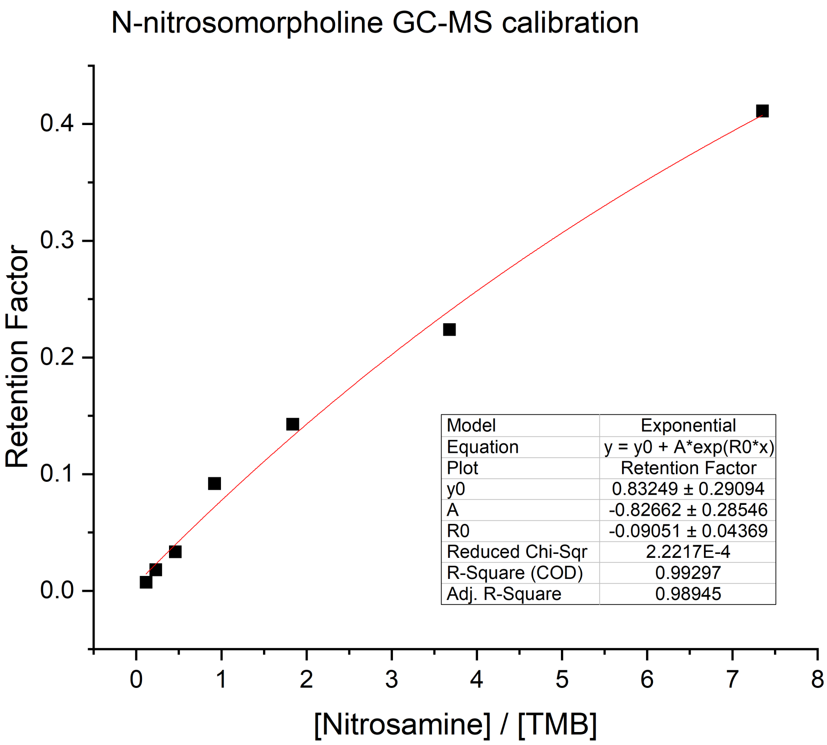

Figure S64: HPLC Calibration curves for N-nitrosomorpholine (N5).

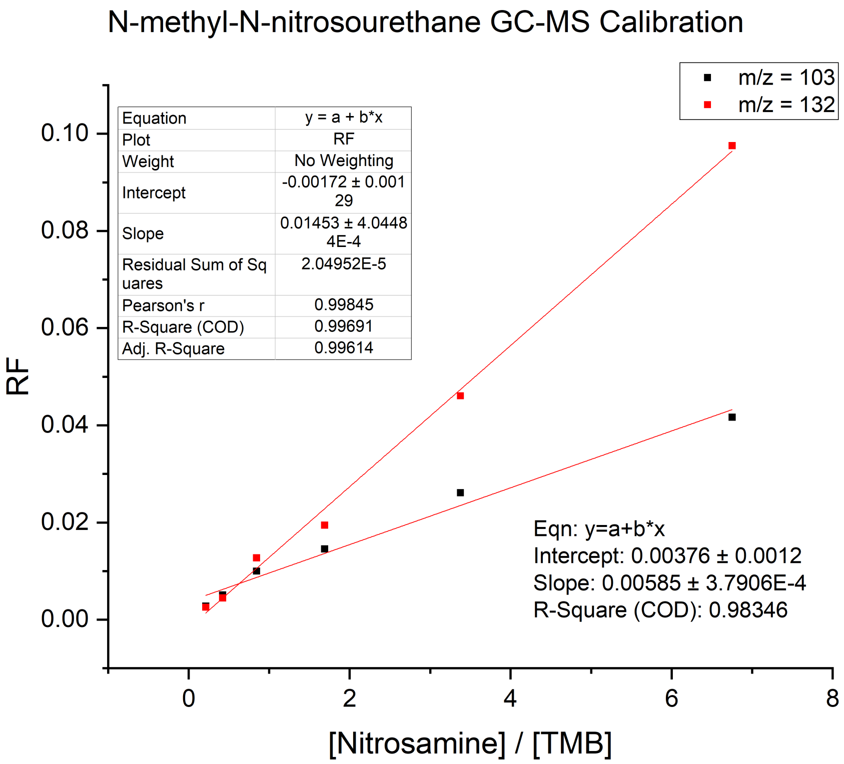

Figure S65: HPLC Calibration curves for N-methyl-N-nitrosourethane (N6).

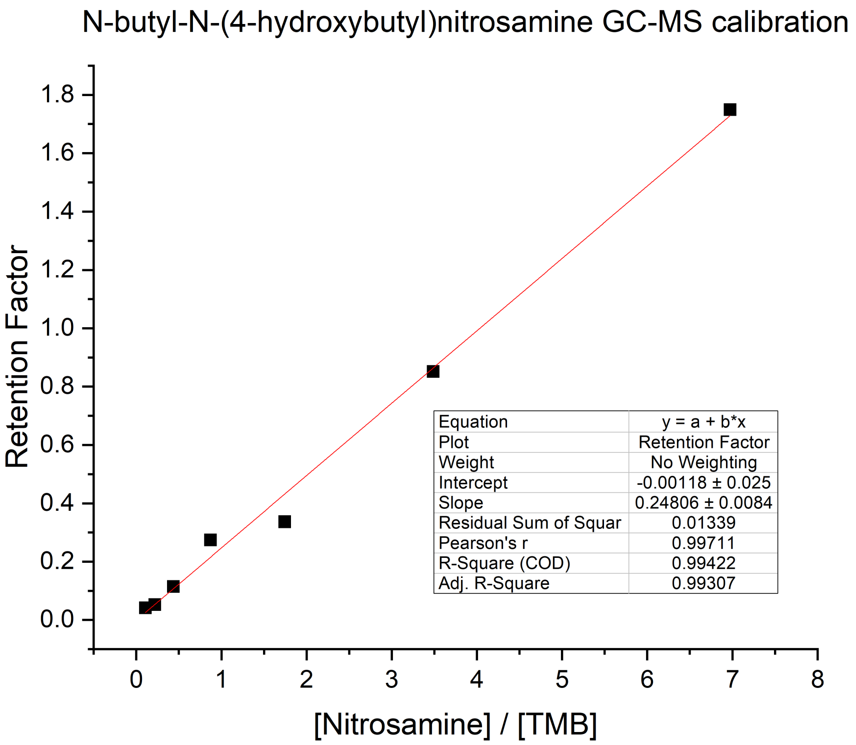

Figure S66: HPLC Calibration curves for *N*-butyl-*N*-(4-hydroxybutyl)nitrosamine (N7).

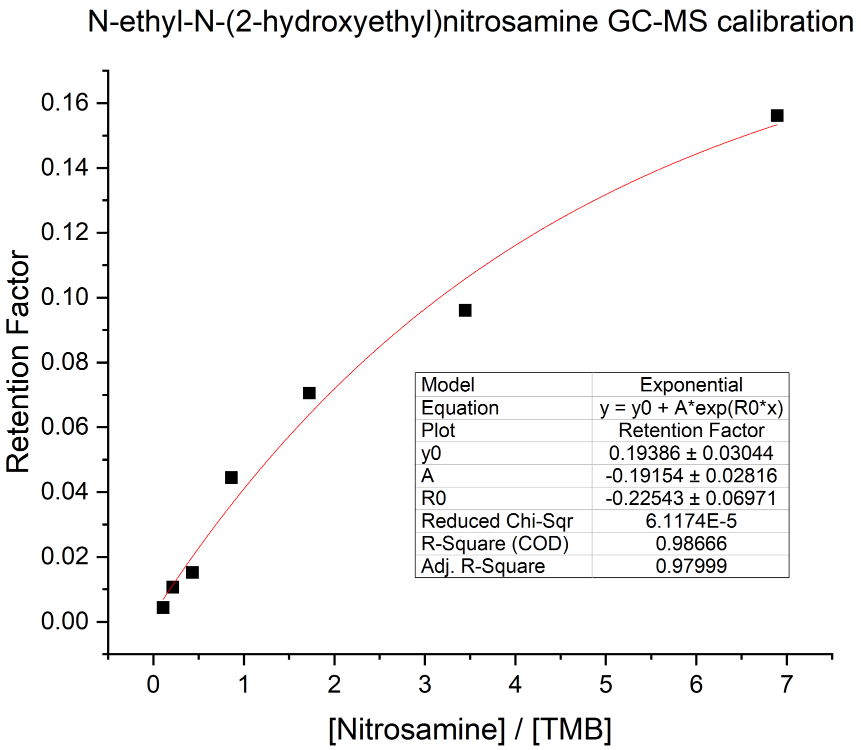

Figure S67: HPLC Calibration curves for *N*-ethyl-*N*-(2-hydroxyethyl)nitrosamine (N8).

6 GC-MS/MS References

The following section details the retention times and mass spectra of the identified products and side products discovered during the GC-MS/MS campaign in this study.

Table S7: GC-MS/MS peak data for known products.

**Analyte**	**MW (g/mol)**	**RT (mins)**	**Mass peaks**	**Adduct**	**Source (ref)**
N1	102.14	3.18	102; 85; 71; 57	M	Authentic (Figure S68)
N-nitrodiethylamine	118.14	5.31	118; 103.1; 91; 79	M	Condition 2 (Figure S81)
N2	130.19	6.53	130; 113; 101; 70	M	Cal (figure S69)
dipropylhydrazine	116.21	2.69	116.1; 87; 72; 59.1	M	Condition 9 (Figure S76)
dipropylamine	101.19	5.05	142.1; 113.1; 85; 71	M+Na+H_2O	Condition 9 (Figure S76)
N-nitrodipropylamine	146.19	8.92	146; 117; 91; 79; 70	M	Condition 2 (Figure S82)
N3	158.25	10.86	158; 141; 116; 99; 84	M	Cal (Figure S70)
dibutylhydrazine	144.26	6.64	144.1; 101.1; 86.1; 59.1	M	Condition 9 (Figure S77)
dibutylamine	129.15	4.48	129; 86	M	Authentic (Figure S75)
dibutylamine	129.15	8.79	170.1; 127.1; 85; 71	M+Na+H_2O	Condition 9 (Figure S77)
N5	116.12	6.52	116; 86; 56	M	Cal (Figure S71)
4-aminomorpholine	102.14	3.44	102.1; 86.1; 72.1; 57.1	M	authentic (Figure S79)
N6	132.12	3.89	132; 103; 58	M	Cal (Figure S72)
N-methyl ethylcarbamate	103.12	2.57	103; 58	M	Cal (Figure S72)
N7	174.24	12.91	157; 100; 84; 71	M	Cal (Figure S73)
N-butyl-*N*-(4-hydroxybutyl) hydrazine	160.26	11.64	160.1; 117.1; 101.1	M	Condition 9 (Figure S80)
4-(butylamino)-1-butanol	145.25	12.32	186.1; 143.1; 127.1	M+Na+H_2O	Condition 9 (Figure S80)
N8	118.14	8.02	87; 75; 57	M	Cal (Figure S74)
2-(1-ethylhydrazino) ethanol	104.15	4.29	104; 73	M	Condition 9 (Figure S137)
2-(ethylamino)ethanol	89.14	3.79	130.1; 115; 99; 73	M+Na+H_2O	Condition 9 (Figure S137)
N8 unexpected product	unknown	8.65	160.1; 129.1; 117.1; 103.1; 88; 73; 58.1	unknown	Condition 9 (Figure S137)
TMB	168.19	12.10	168; 139; 25; 109	M	N1 Cal (Figure S68)
2,6-di-tert-butyl-4-methylphenol (BHT)	220.36	12.73	220.1; 205.1; 177; 145	M	N1 Condition 8 (Figure S87)

Furthermore, side products associated with reaction of the internal standard in conditions

1 and 2 are also suggested. Though these peaks are not verified by authentic samples as they did not form part of the core objective of the project. Details of the retention times and mass peaks for the side products found are summarised in Table S8. Links to the reference plots are available by clicking the link in the "Source" column.

Table S8: GC-MS/MS peak data for side products.

**Analyte**	**MW (g/mol)**	**RT (mins)**	**Mass peaks**	**Adduct**	**Source (ref)**
2-Chloro-1,3,5-trimethoxybenzene	202.63	13.28	202; 173; 159; 138	M	N6 Condition 1 (Figure S83)
2-Chloro-1,3,5-trimethoxybenzene	202.63	13.59	247.9; 245.9; 168; 138	M+EtOH	N1 Condition 2 (Figure S84)
2,4-dichloro-1,3,5-trimethoxybenzene	202.63	13.64	237.9; 235.9; 192.9; 177.9	M	N3 Condition 2 (Figure S85)
2,4-dichloro-1,3,5-trimethoxybenzene	202.63	13.95	283.9; 281.9; 238.9; 202	M+EtOH	N5 Condition 2 (Figure S86)
2-bromo-4-chloro-1,3,5-trimethoxybenzene	281.53	14.26	327.9; 325.9; 324.0	M+EtOH	N7 Condition 3 (Figure S88)

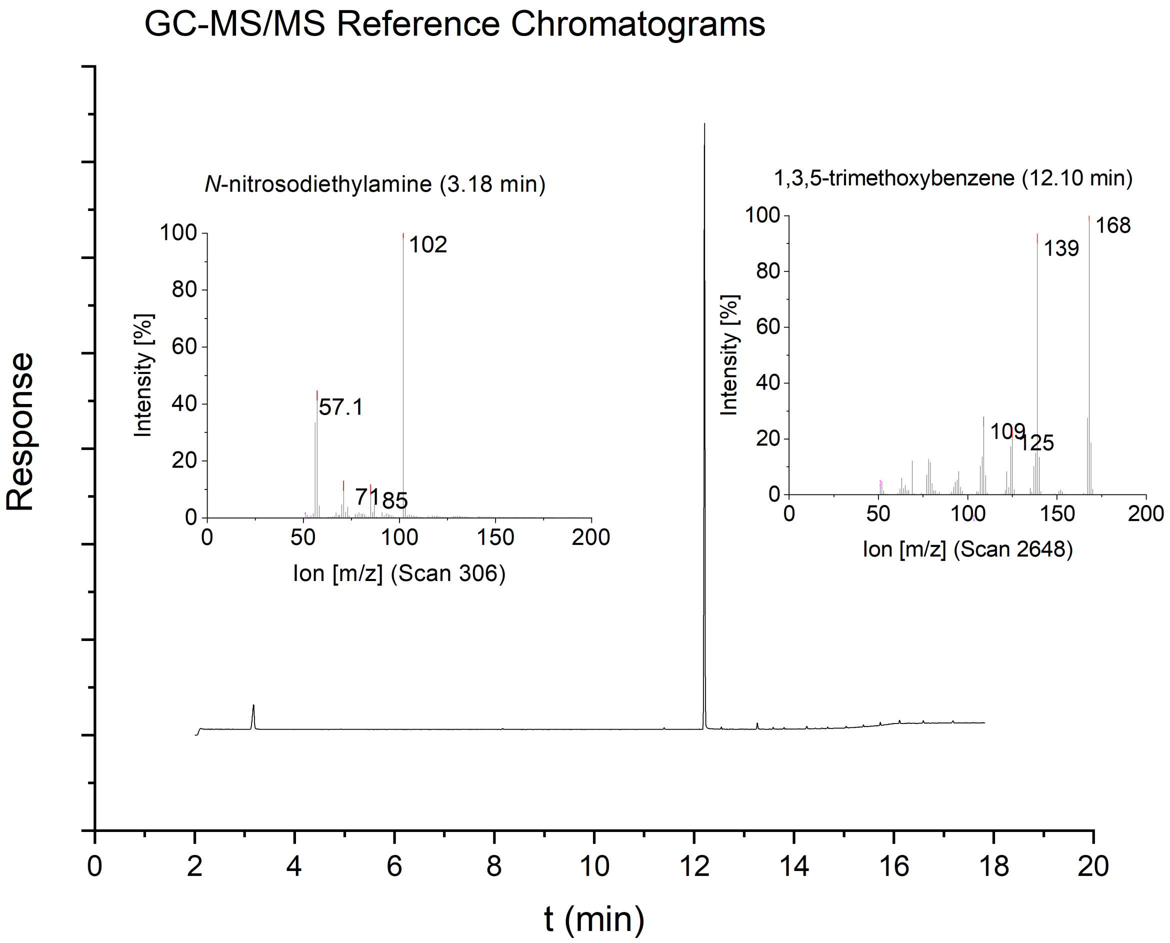

Figure S68: GC-MS/MS reference for N-nitrosodiethylamine (N1). Referenced in Table S7

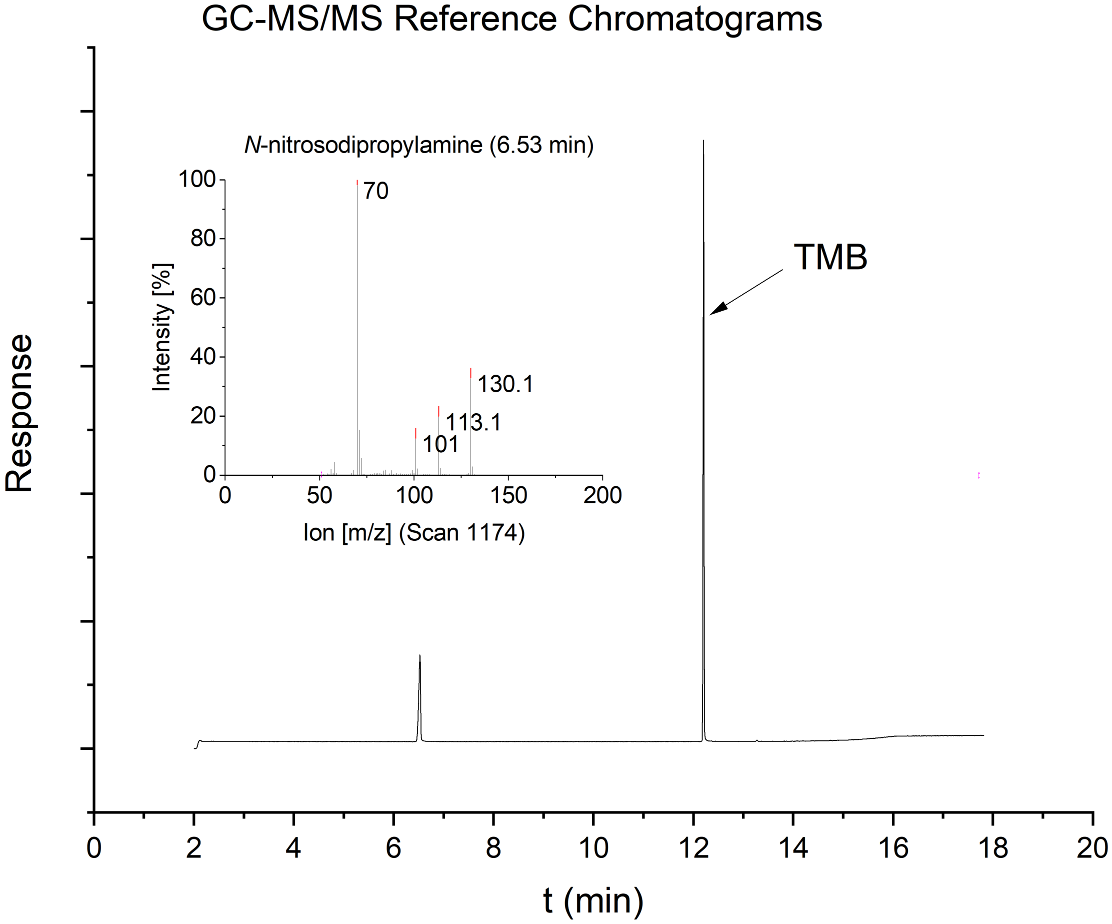

Figure S69: GC-MS/MS reference for N-nitrosodipropylamine (N2). Referenced in Table S7

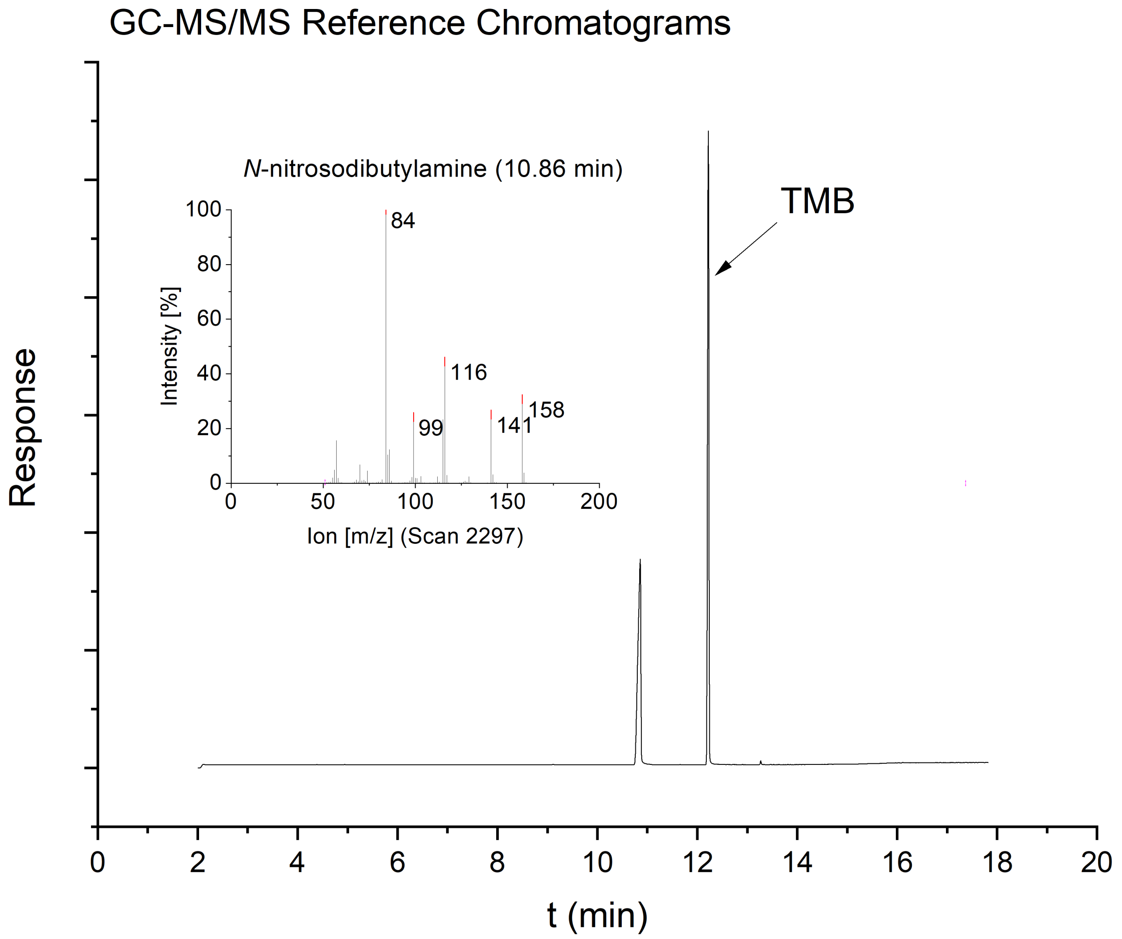

Figure S70: GC-MS/MS reference for N-nitrosodibutylamine (N3). Referenced in Table S7

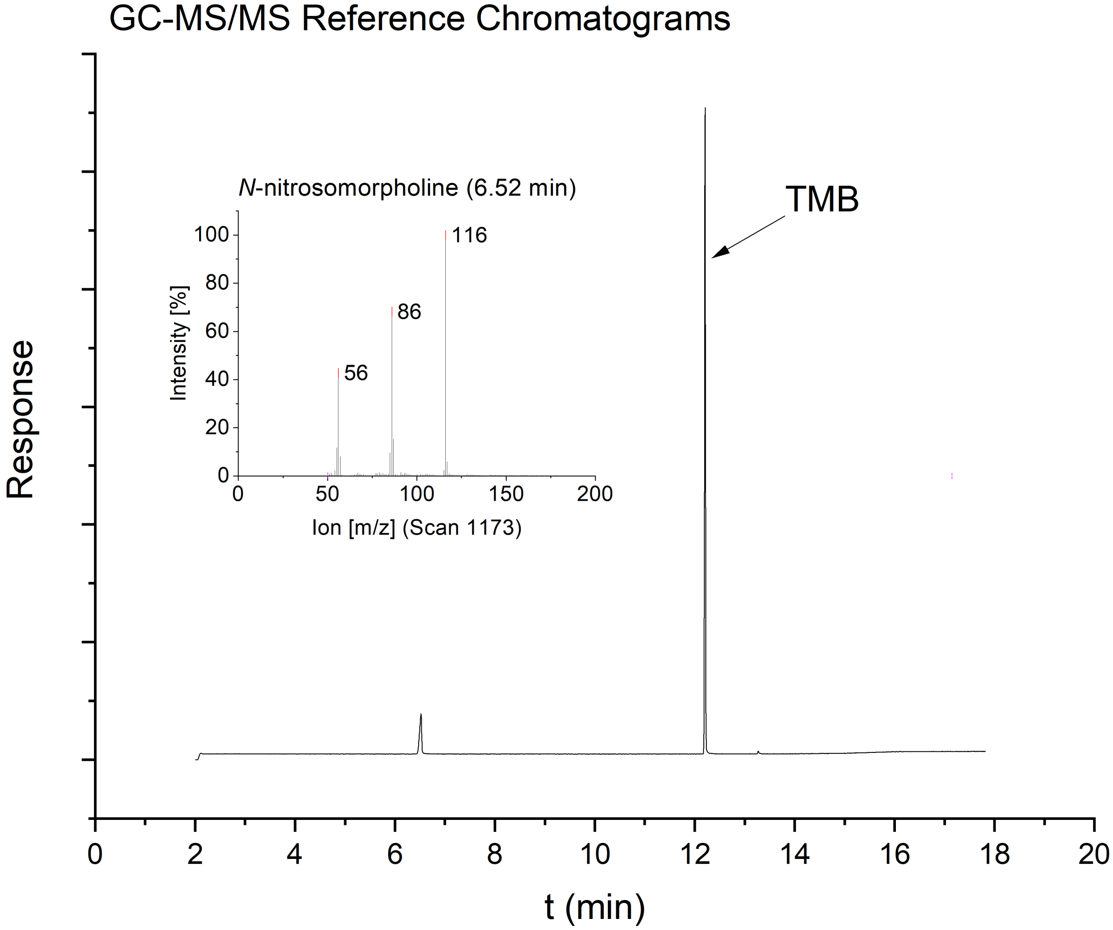

Figure S71: GC-MS/MS reference for N-nitrosmorpholine (N5). Referenced in Table S7

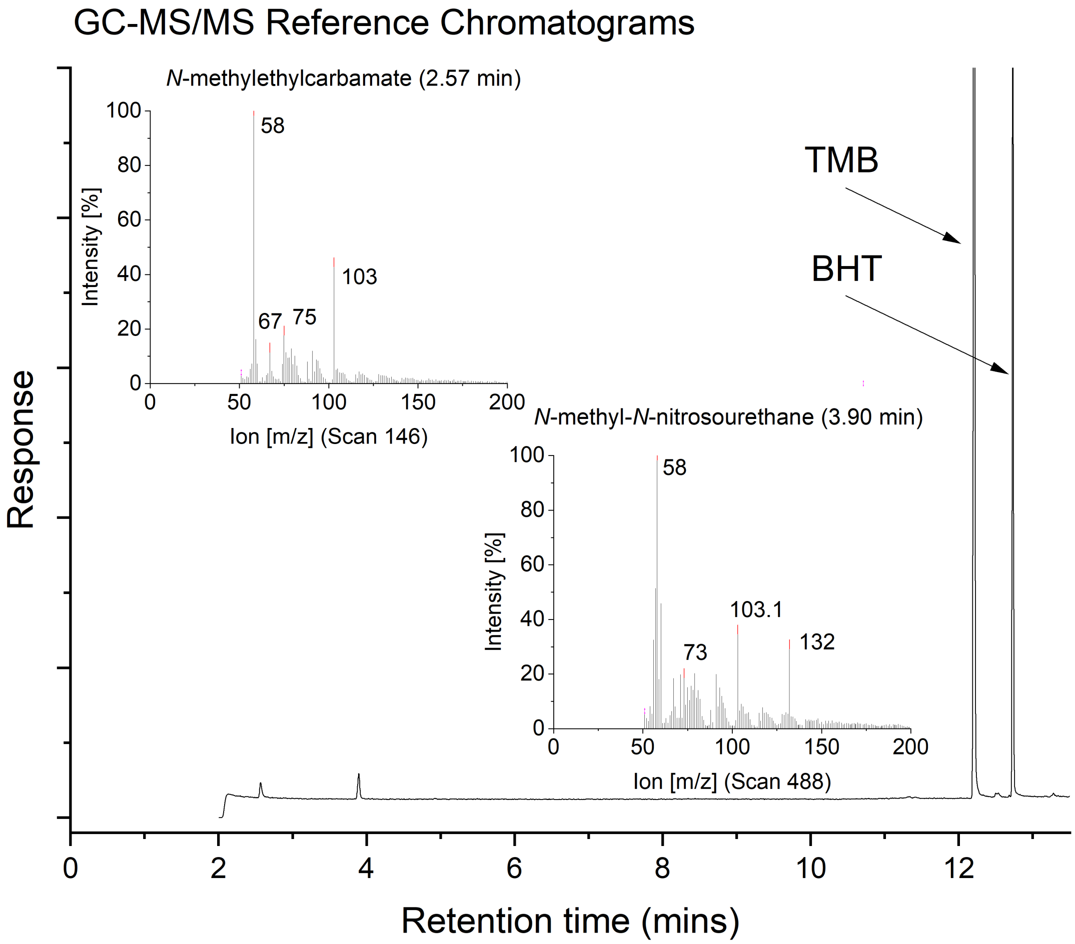

Figure S72: GC-MS/MS reference for N-methyl-N-nitrosourethane (N6) and the corresponding amine N-methylethylcarbamate. Referenced in Table S7

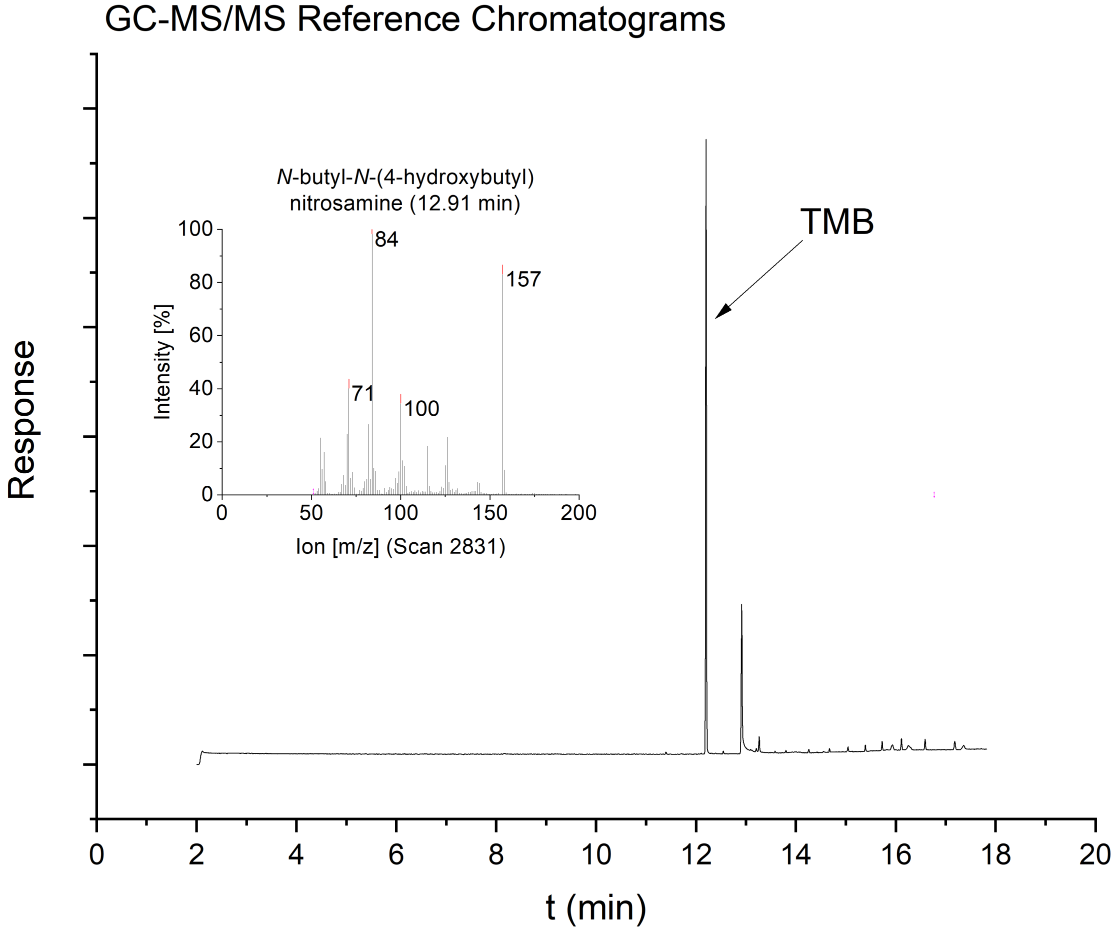

Figure S73: GC-MS/MS reference for *N*-butyl-*N*-(4-hydroxybutyl)-nitrosamine (N7). Referenced in Table S7

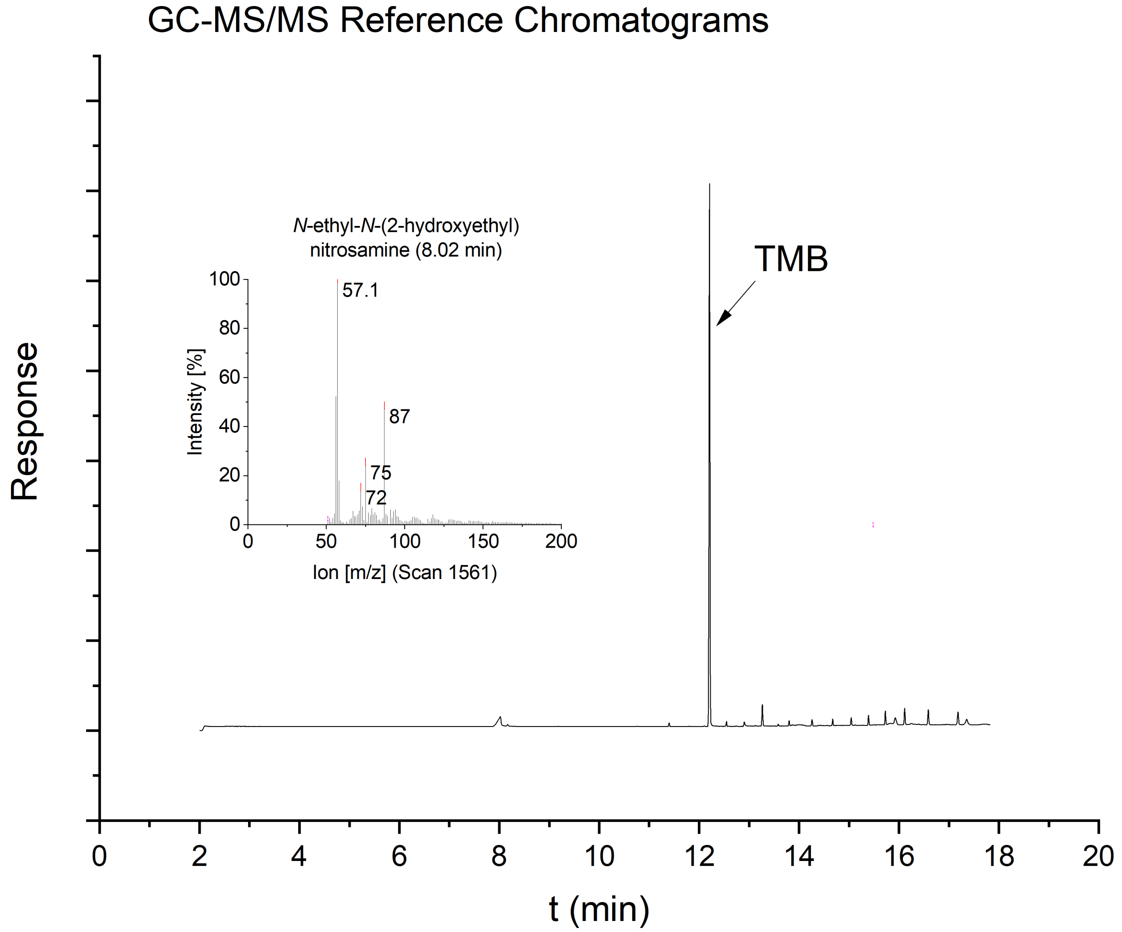

Figure S74: GC-MS/MS reference for *N*-ethyl-*N*-(2-hydroxyethyl)-nitrosamine (N8). Referenced in Table S7

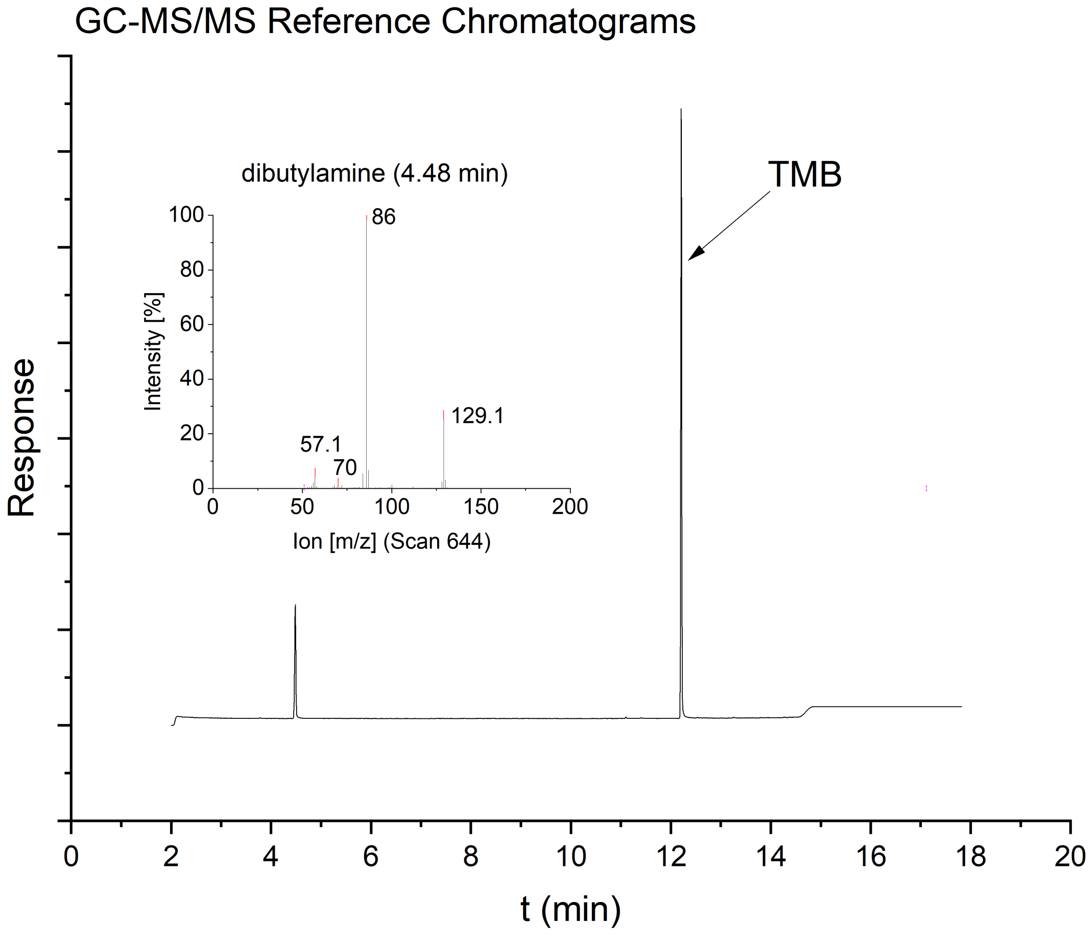

Figure S75: GC-MS/MS reference for dibutylamine. Referenced in Table S7

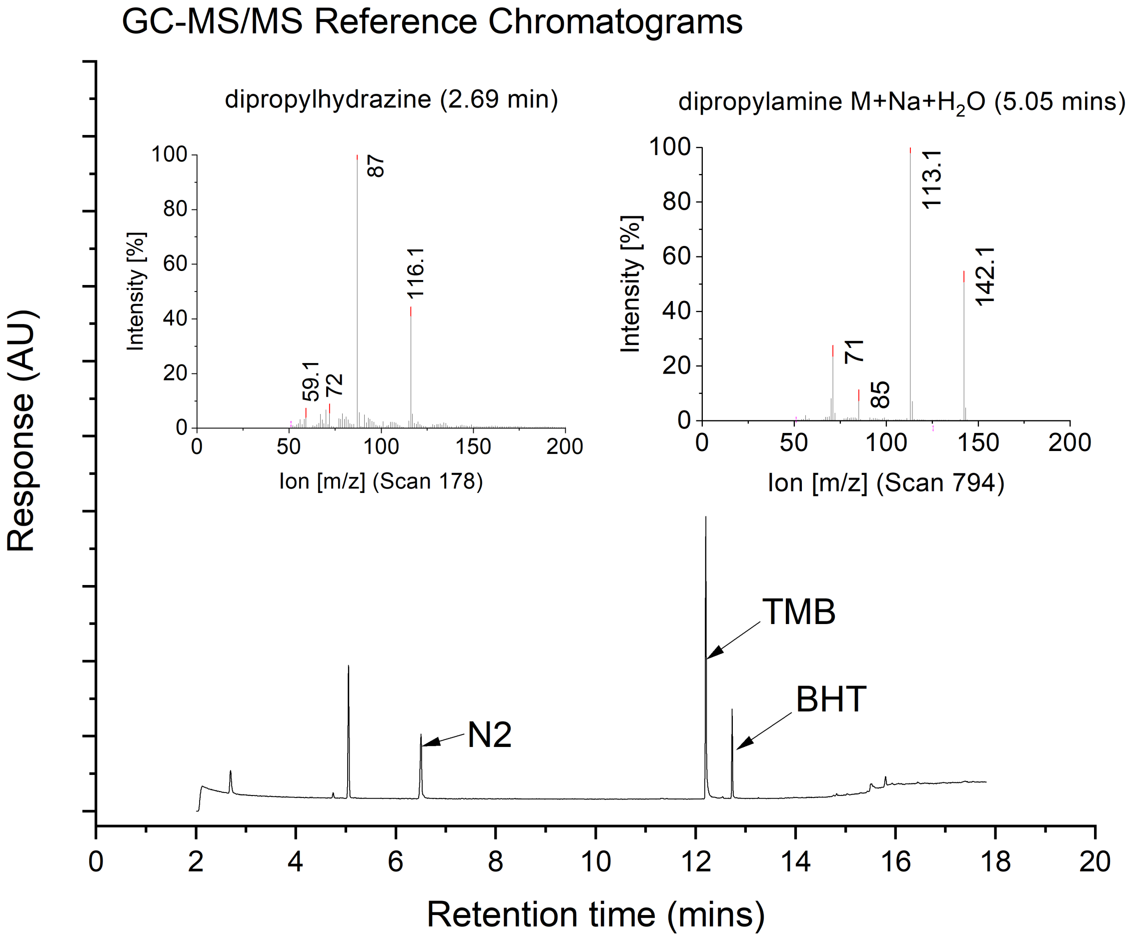

Figure S76: GC-MS/MS reference for dipropylamine and dipropylhydrazine, from reaction of N2 with DiBAL-H. Referenced in Table S7

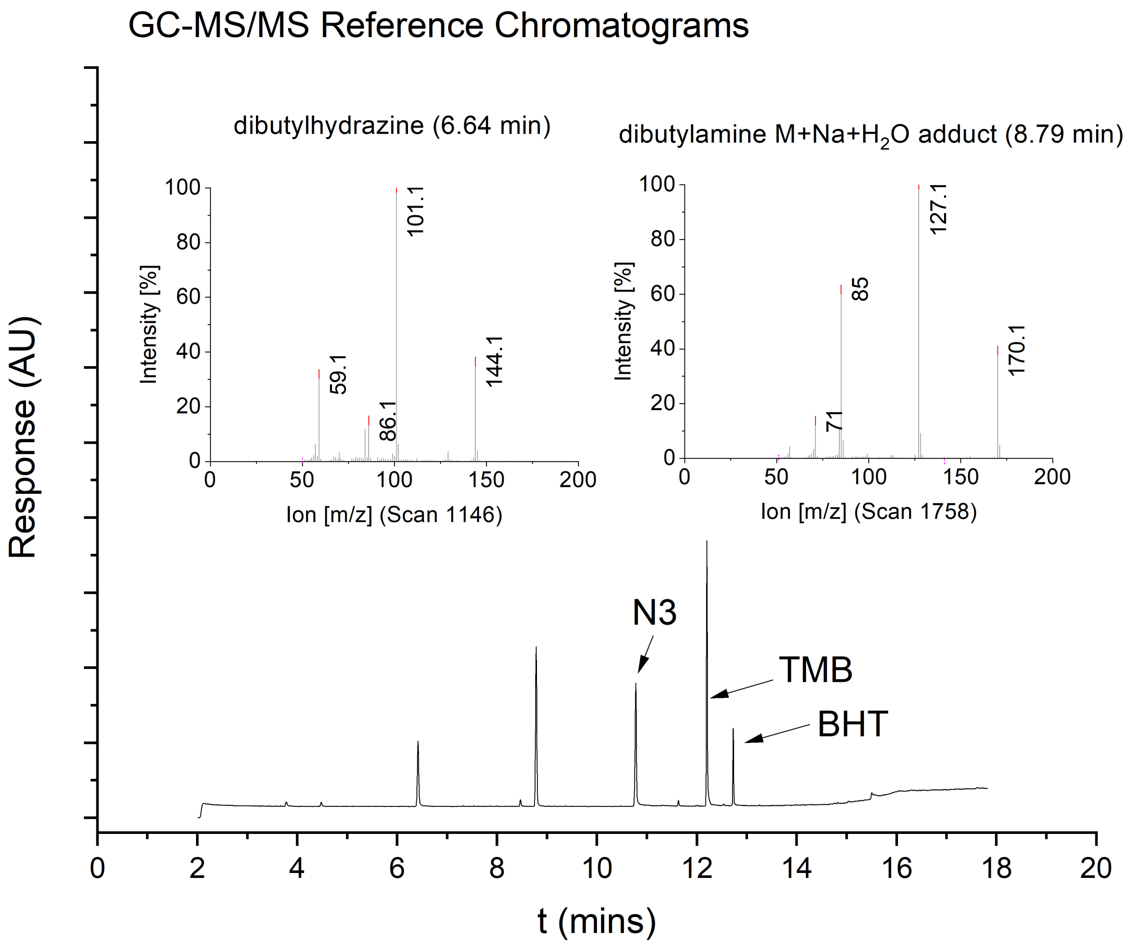

Figure S77: GC-MS/MS reference for dibutylamine and dibutylhydrazine, from reaction of N3 with DiBAL-H. Referenced in Table S7

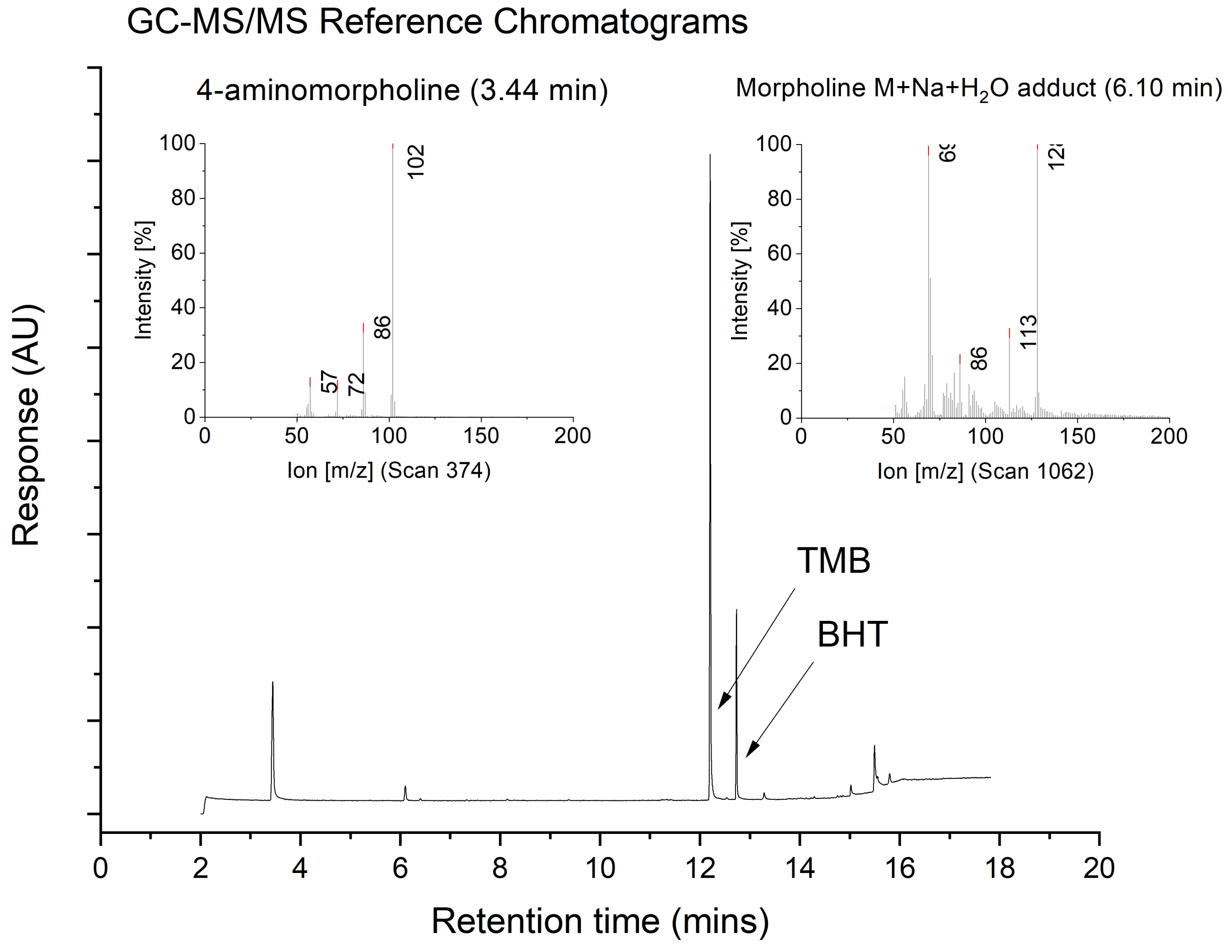

Figure S78: GC-MS/MS reference for morpholine and 4-aminomorpholine, from reaction of N5 with DiBAL-H. Referenced in Table S7

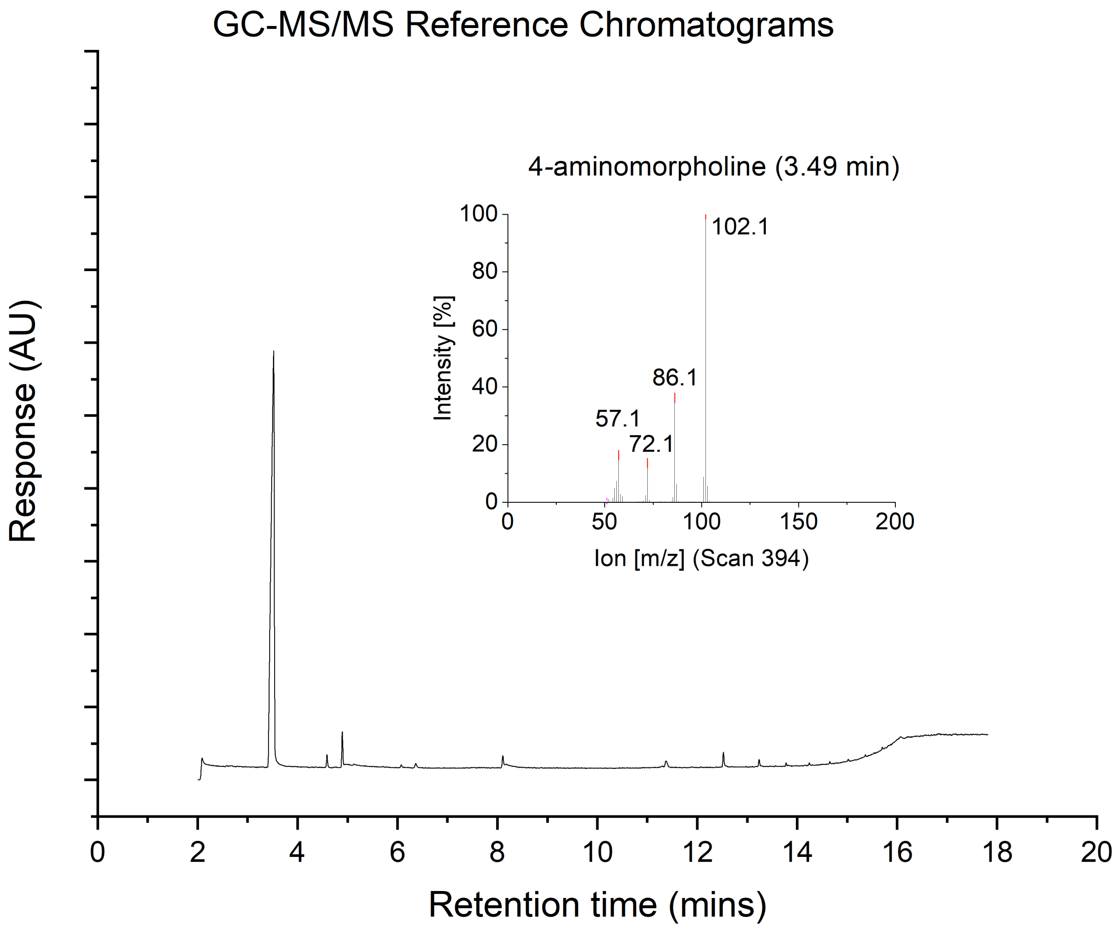

Figure S79: GC-MS/MS reference for 4-aminomorpholine, from authentic reference sample. Referenced in Table S7

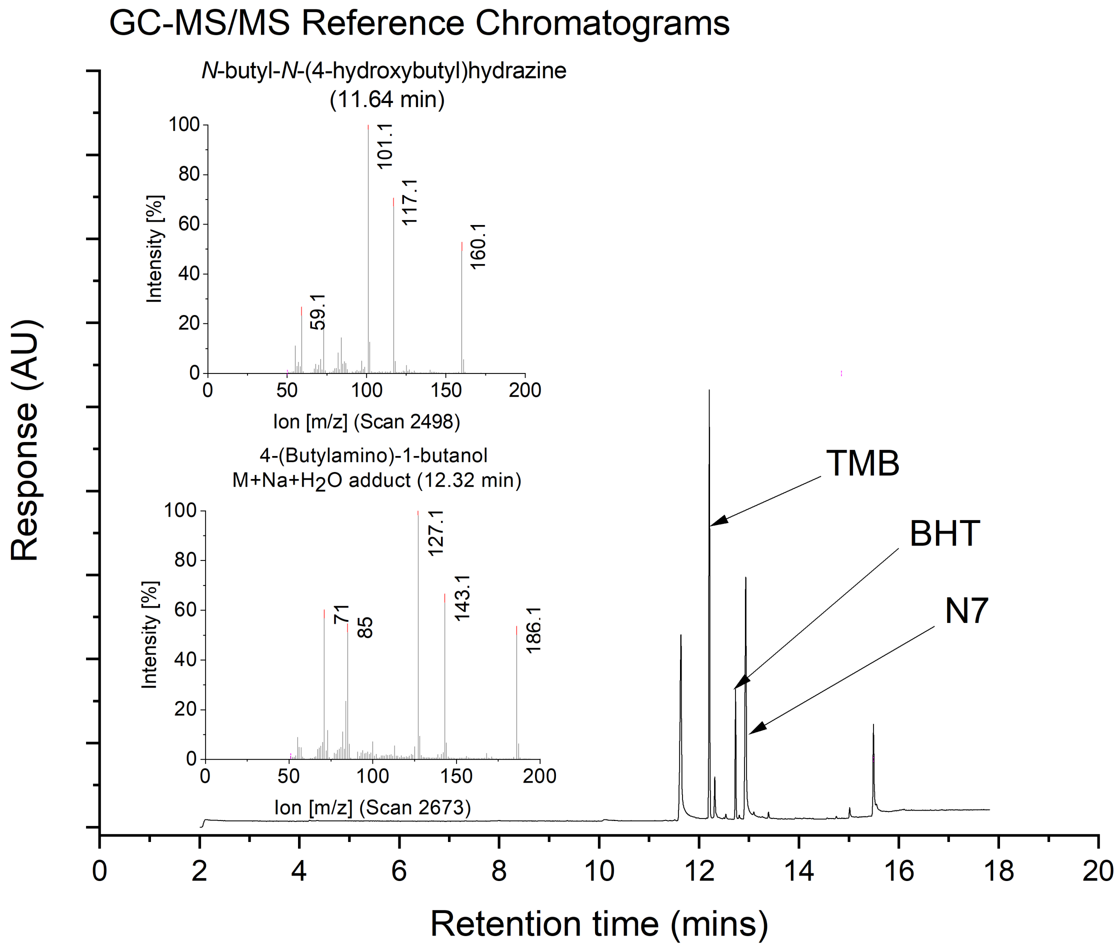

Figure S80: GC-MS/MS reference for 4-(butylamino)-1-butanol and N-butyl-N-(4-hydroxybutyl)hydrazine, from reaction of N7 with DiBAL-H. Referenced in Table S7

GC-MS/MS Reference Chromatograms

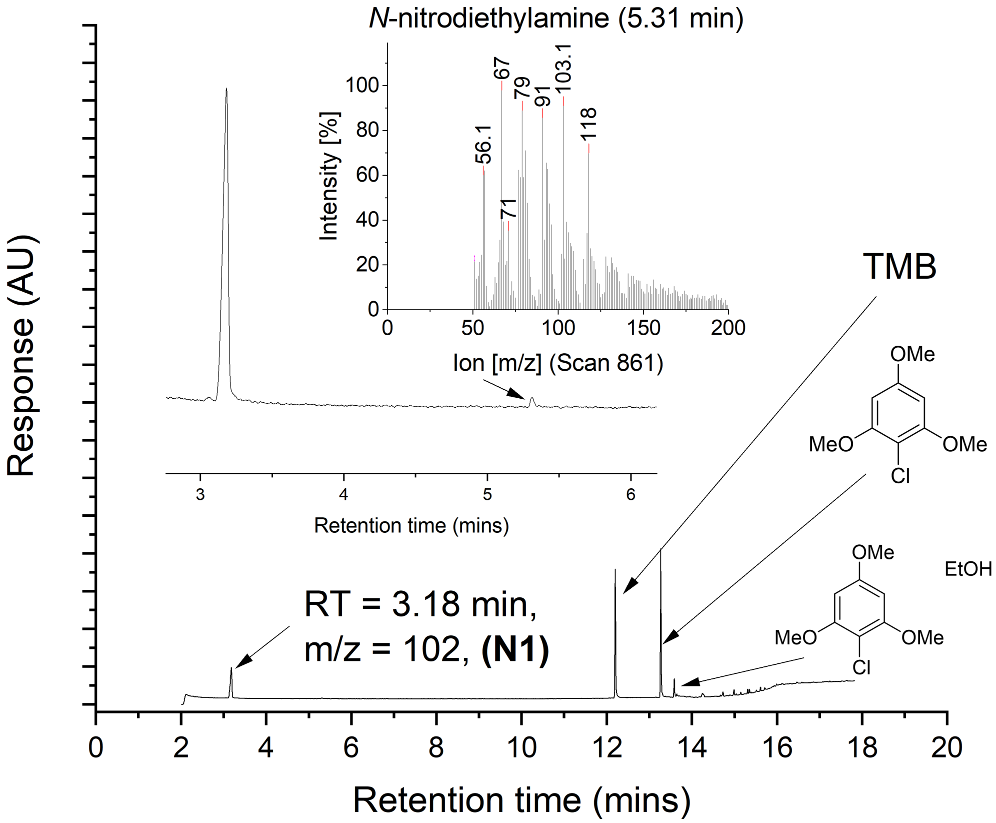

Figure S81: GC-MS/MS reference for N-nitrodiethylamine from reaction of N1 with CH_3CO_3H. Referenced in Table S7

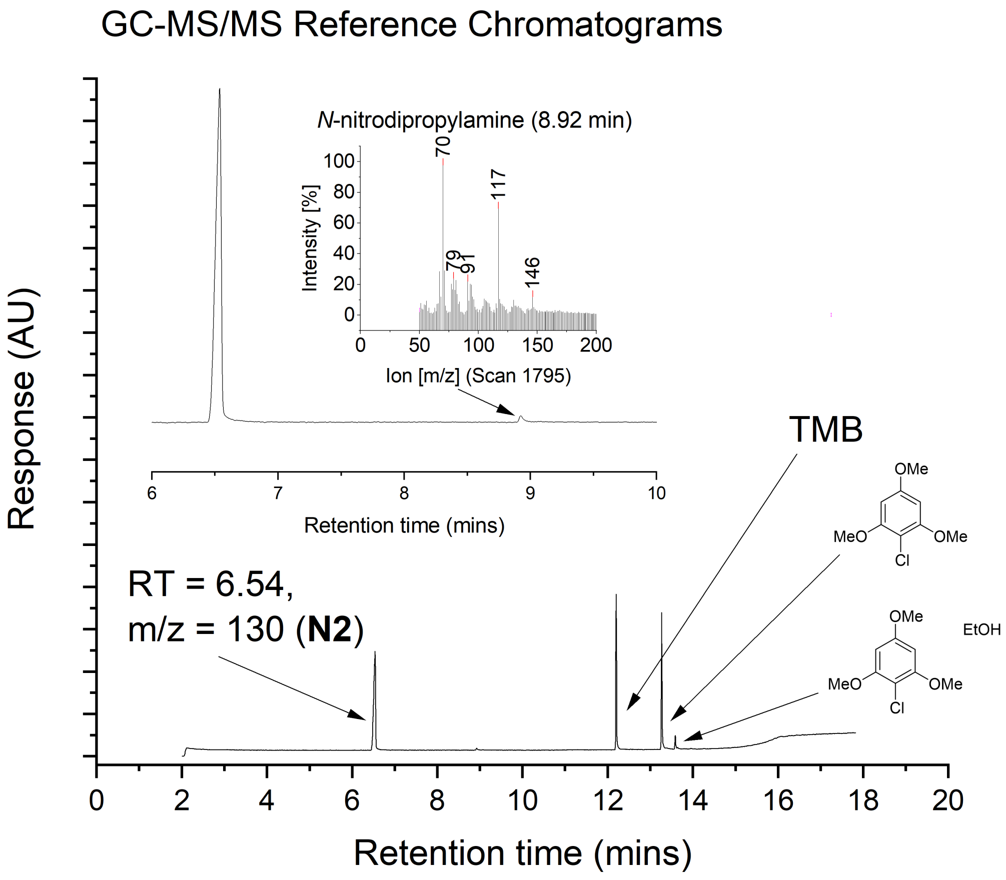

Figure S82: GC-MS/MS reference for N-nitrodipropylamine from reaction of N2 with CH_3CO_3H. Referenced in Table S7

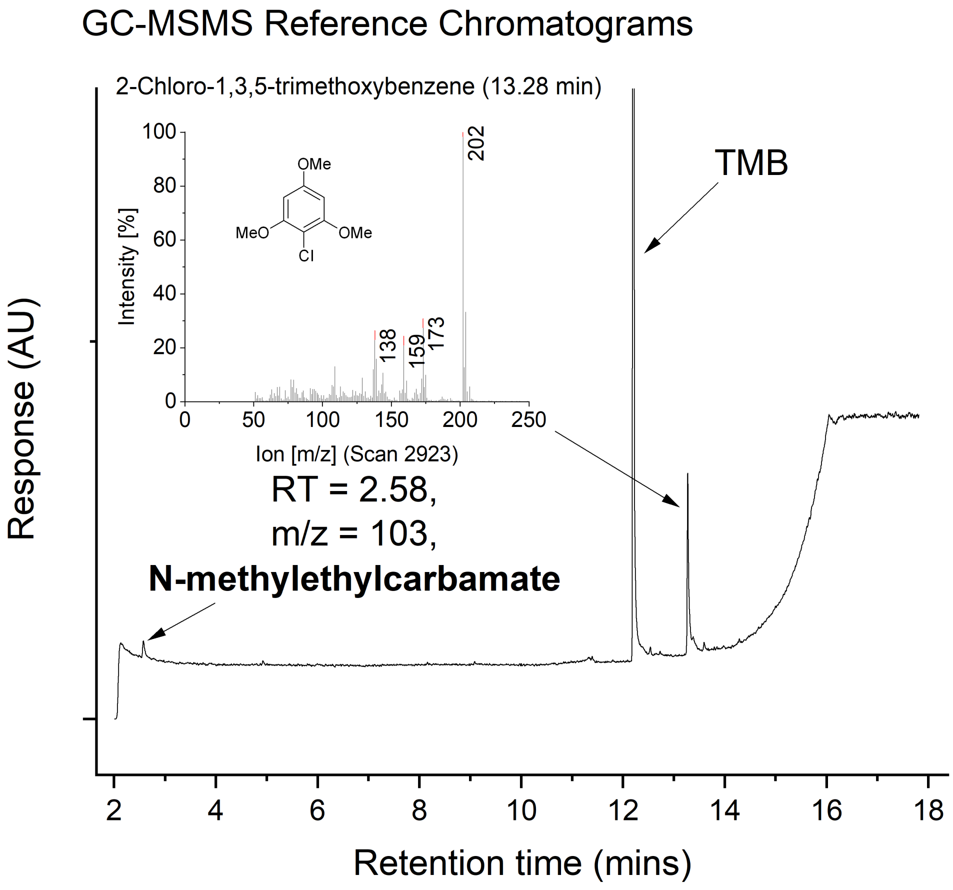

Figure S83: GC-MS/MS reference for 2-Chloro-1,3,5-trimethoxybenzene from reaction of N6 with H_2O_2. Referenced in Table S8

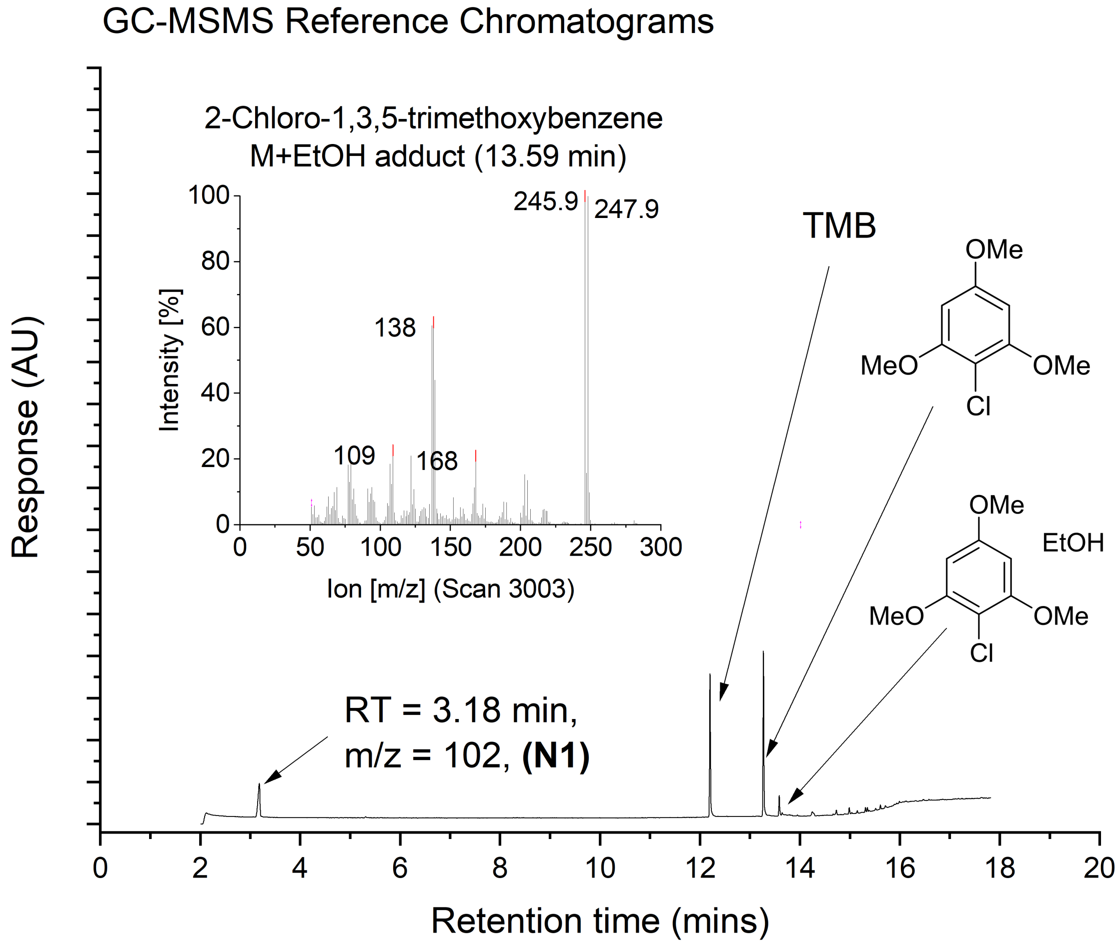

Figure S84: GC-MS/MS reference for 2-Chloro-1,3,5-trimethoxybenzene (M+EtOH adduct) from reaction of N1 with CH_3CO_3H. Referenced in Table S8

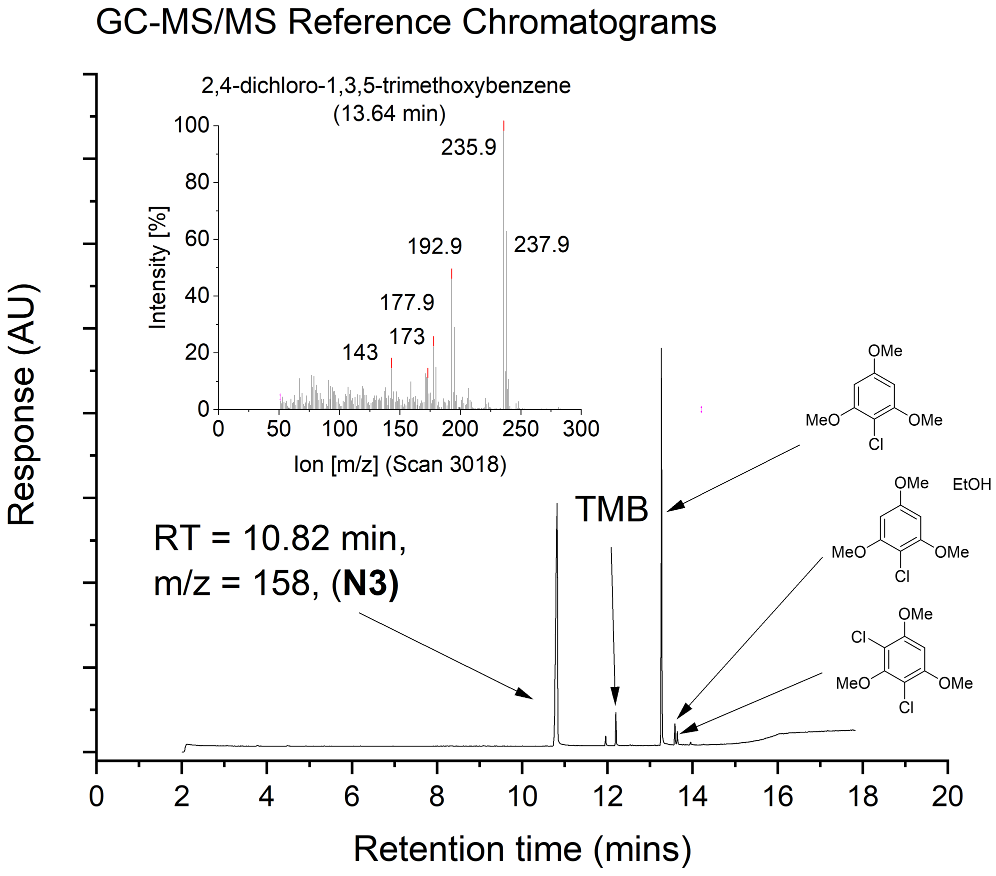

Figure S85: GC-MS/MS reference for 2,4-dichloro-1,3,5-trimethoxybenzene from reaction of N3 with CH_3CO_3H. Referenced in Table S8

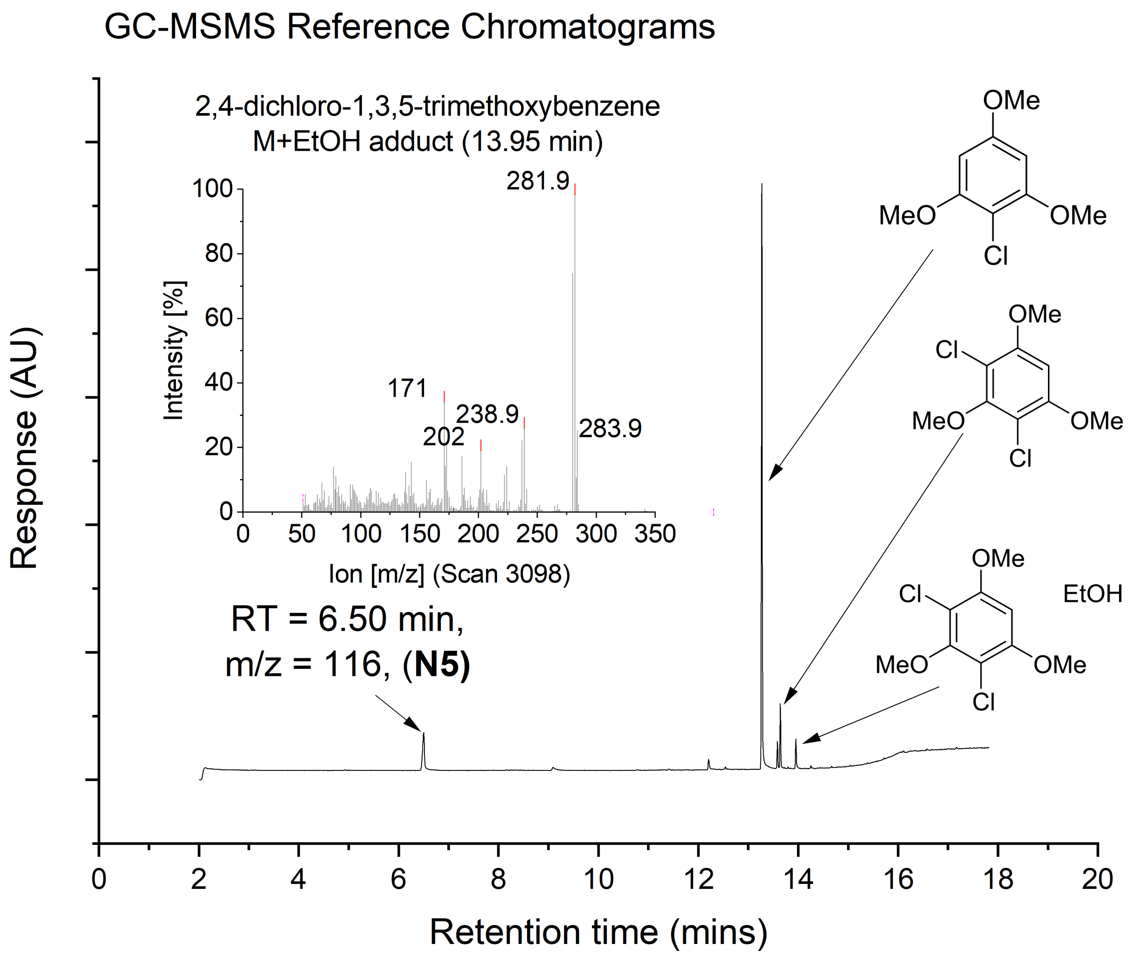

Figure S86: GC-MS/MS reference for 2,4-dichloro-1,3,5-trimethoxybenzene (M+EtOH adduct) from reaction of N5 with CH_3CO_3H. Referenced in Table S8

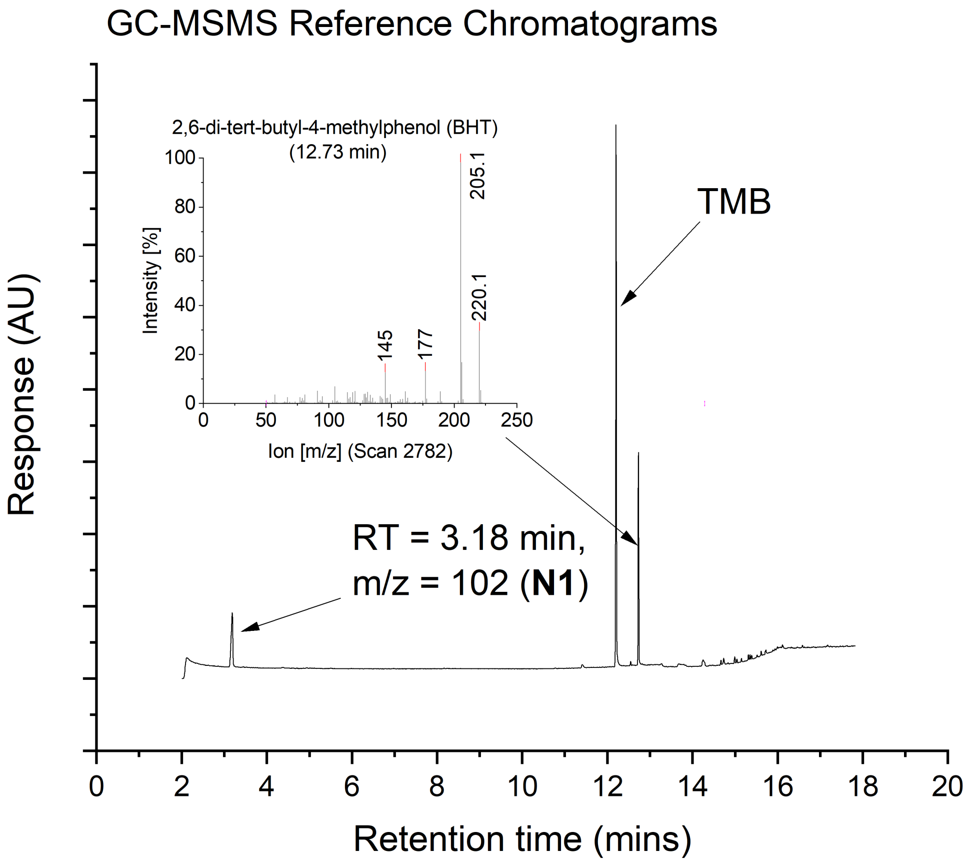

Figure S87: GC-MS/MS reference for 2,6-di-tert-butyl-4-methylphenol (BHT) from reaction of N1 with $NaBH_4$. Referenced in Table S7

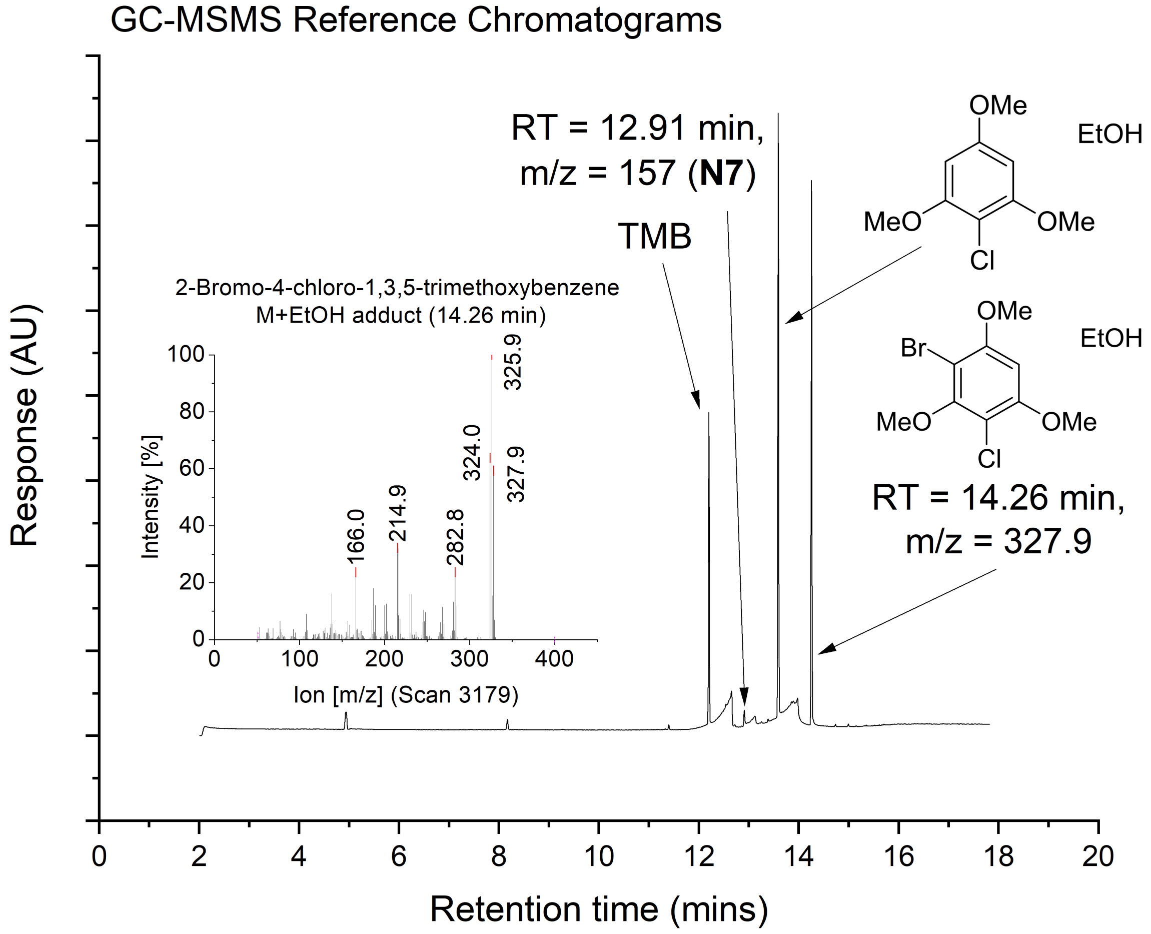

Figure S88: GC-MS/MS reference for 2-Bromo-4-chloro-1,3,5-trimethoxybenzene M+EtOH adduct from reaction of N7 with HBr. Referenced in Table S8

7 LC-MS/MS References

The following section details the retention times annd mass spectra for the products identified by the LC-MS/MS campaign employed in this work.

Table S9: LC-MS/MS reference mass peaks and retention times.

Analyte	MW (g/mol)	RT (min)	Mass peaks	Adduct	Source	ref
N4	198.23	5.66	199.09; 184.07; 169.09	M+H	Authentic	Figures S89 and S90
diphenylamine	169.23	5.75	170.10	M+H	Authentic	Figures S91 and S92
N,N'-diphenylhydrazine	184.24	5.21	185.11; 168.08	M+H	Authentic	Figures S93 and S94
p-nitrosodiphenylamine	198.23	5.42	199.08; 122.09	M+H	Condition 7	Figures S95 and S96
N-nitrodiphenylamine	214.22	5.70	215.09; 170.10	M+H	Condition 2	Figures S97 and S98
N7	174.24	4.62	175.14; 102.09	M+H	Authentic	Figures S99 and S100
N8	118.14	1.42	119.08	M+H	Authentic	Figures S101 and S102
4-(butylamino)-1-butanol	145.25	0.61	146.16	M+H	Authentic	Figures S103 and S104
N8 condition 9 (3+4)	N/A	1.17	161.16	Unknown	Condition 9	Figures S105 and S106

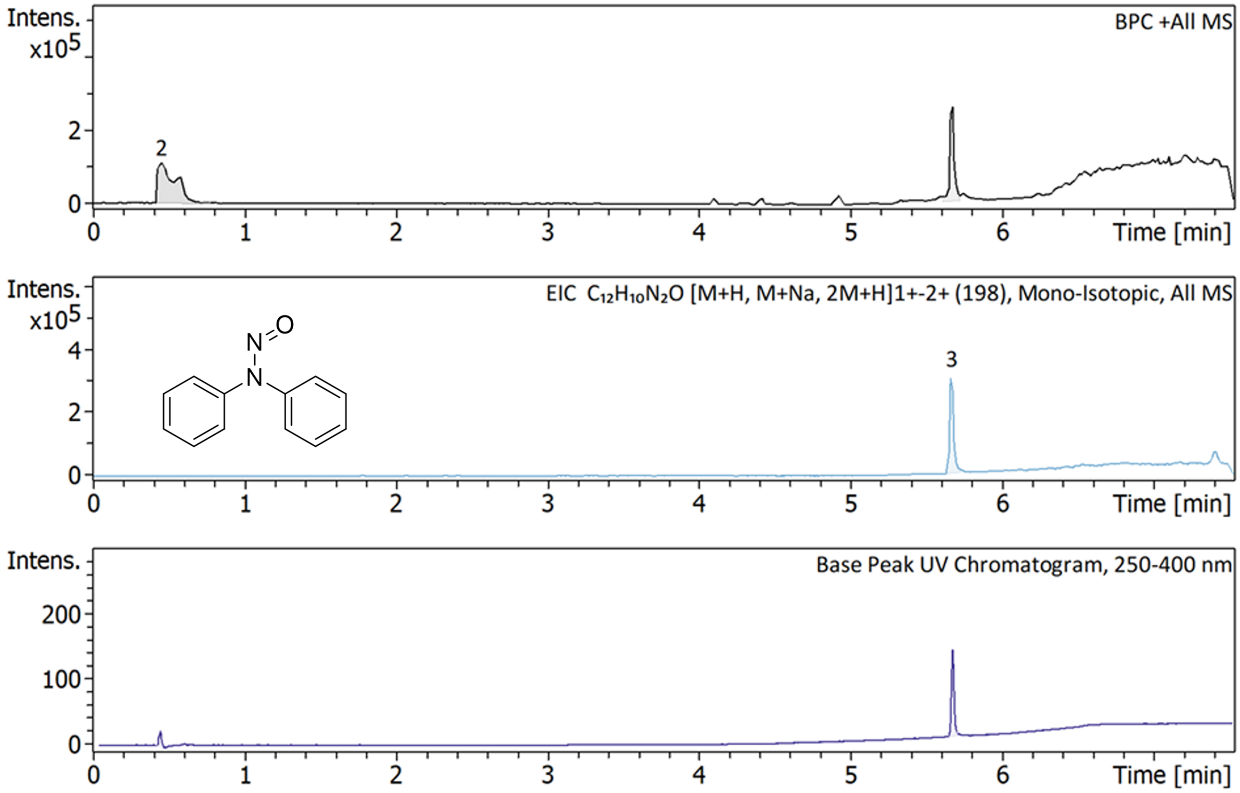

Summary of Results

Name	RT	BPC Area(%)	UV Area(%)	Confirm Formula Results
Cmpd 2, 0.5 min	0.45	62.0	14.1	
Cmpd 3, 5.7 min	5.66	38.0	85.9	C12H10N2O

Figure S89: LC-MS/MS reference chromatogram for *N*-nitrosodiphenylamine (N4). As referenced in Table S9.

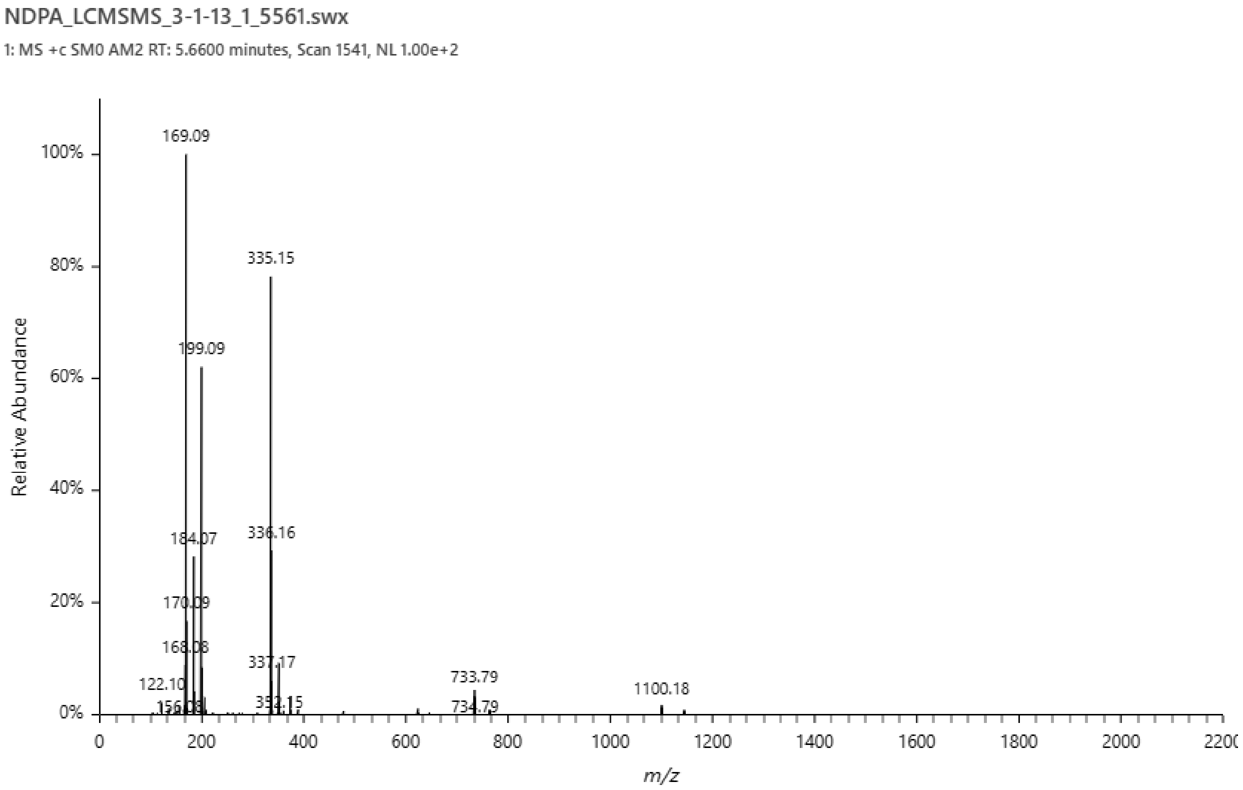

Figure S90: LC-MS/MS reference mass peaks for *N*-nitrosodiphenylamine (N4). As referenced in Table S9.

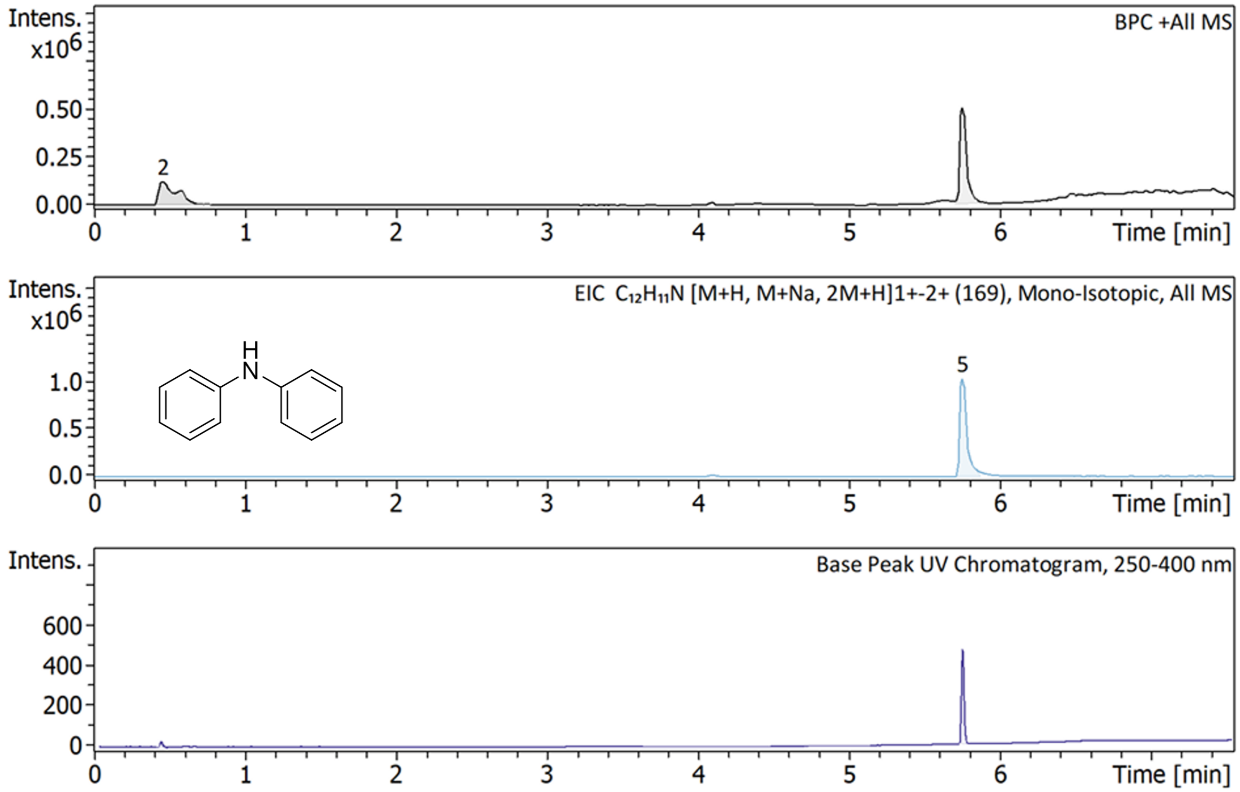

Summary of Results

Name	RT	BPC Area(%)	UV Area(%)	Confirm Formula Results
Cmpd 2, 0.5 min	0.46	38.5	4.5	
Cmpd 5, 5.7 min	5.75	61.5	78.2	C12H11N

Figure S91: LC-MS/MS reference chromatogram for diphenylamine. As referenced in Table S9.

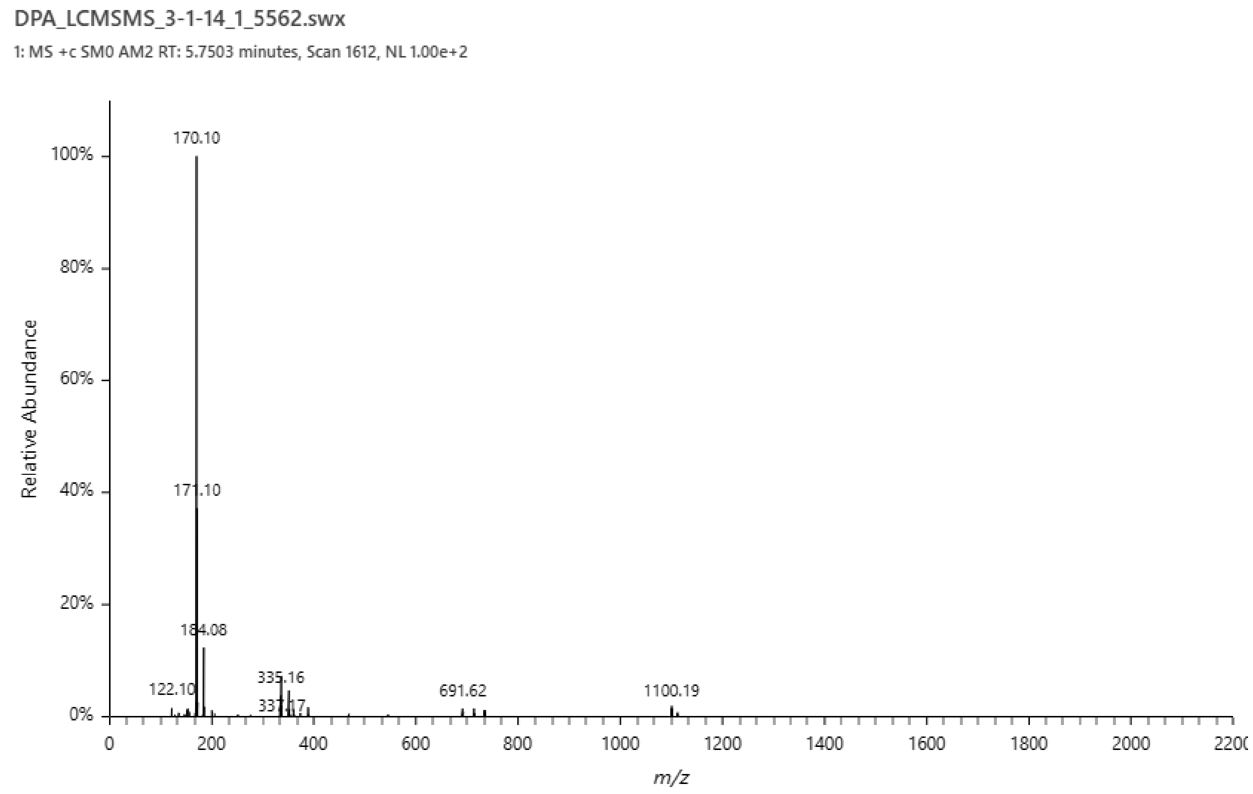

Figure S92: LC-MS/MS reference reference mass peaks for diphenylamine. As referenced in Table S9.

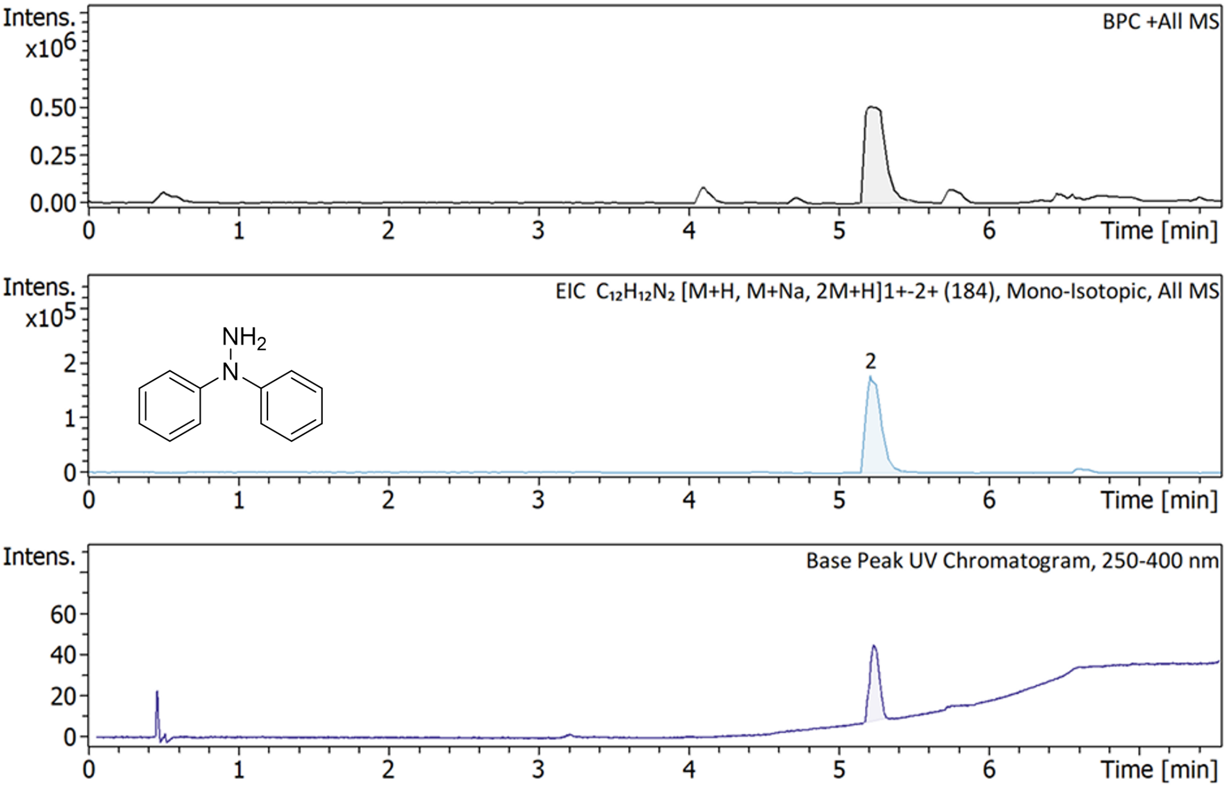

Summary of Results

Name	RT	BPC Area(%)	UV Area(%)	Confirm Formula Results
Cmpd 2, 5.2 min	5.21	100.0	88.4	C12H12N2

Figure S93: LC-MS/MS reference chromatogram for *N*,*N*'-diphenylhydrazine. As referenced in Table S9.

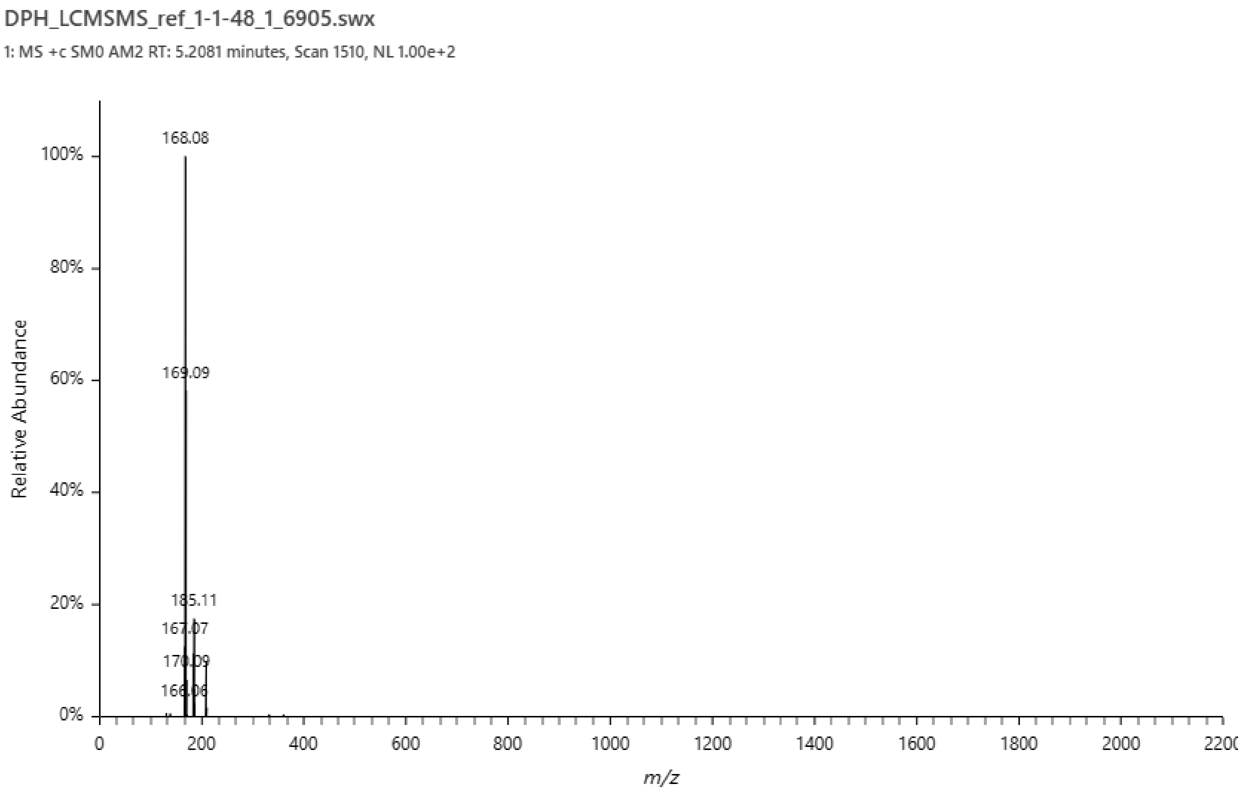

Figure S94: LC-MS/MS reference mass peaks for *N*,*N*'-diphenylhydrazine. As referenced in Table S9.

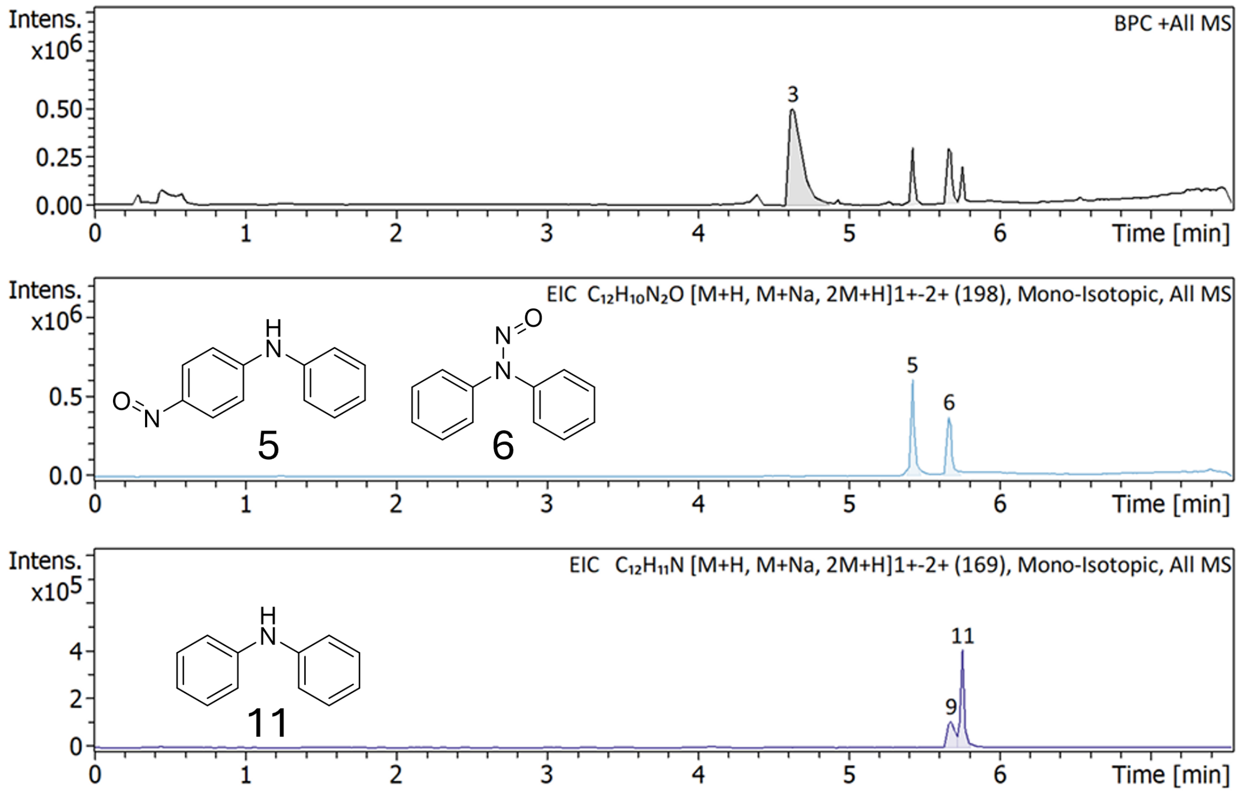

Summary of Results

Name	RT	BPC Area(%)	UV Area(%)	Confirm Formula Results
Cmpd 3, 4.6 min	4.63	72.8	no peak	
Cmpd 5, 5.4 min	5.42	10.9	no peak	C12H10N2O
Cmpd 6, 5.7 min	5.66	16.3	78.2	C12H10N2O
Cmpd 9, 5.7 min	5.67	16.3	78.2	C12H10N2O
Cmpd 11, 5.8 min	5.75	no peak	4.3	C12H11N

Figure S95: LC-MS/MS reference chromatogram for *p*-nitrosodiphenylamine from reaction condition 4. As referenced in Table S9.

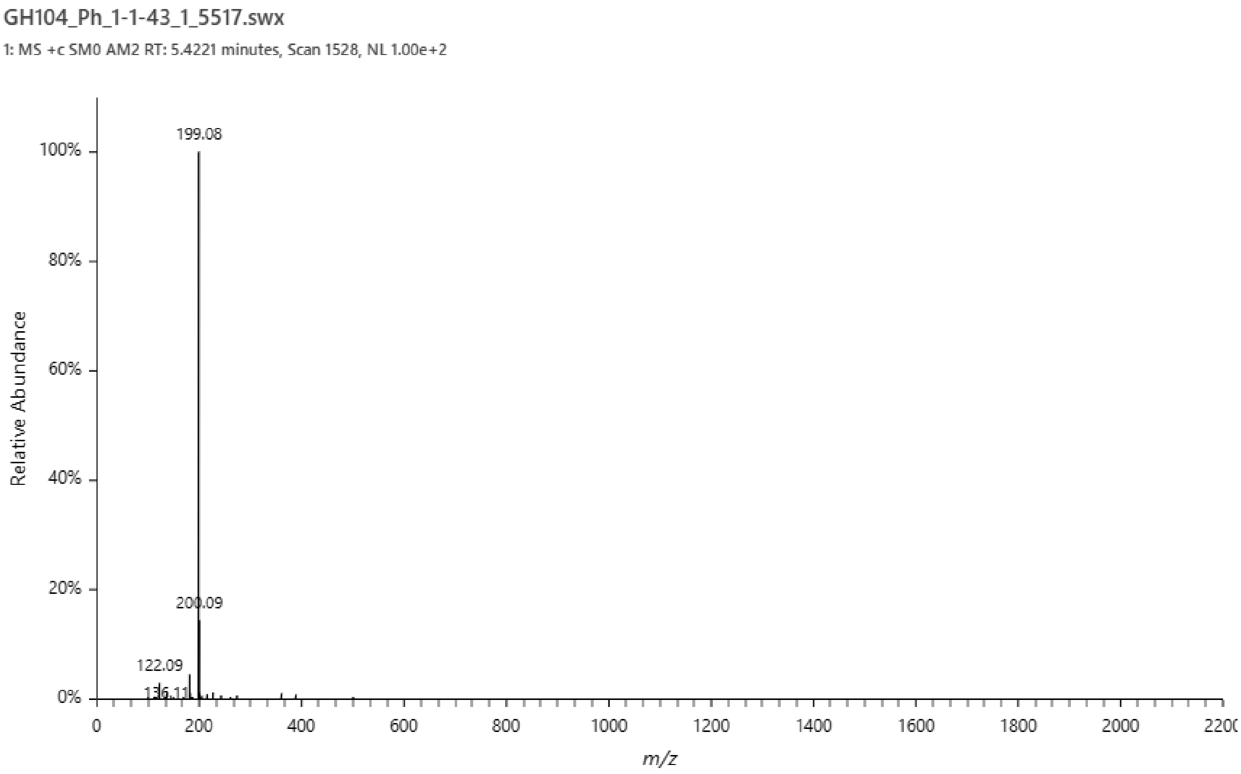

Figure S96: LC-MS/MS reference mass peaks for *p*-nitrosodiphenylamine from reaction condition 4. As referenced in Table S9.

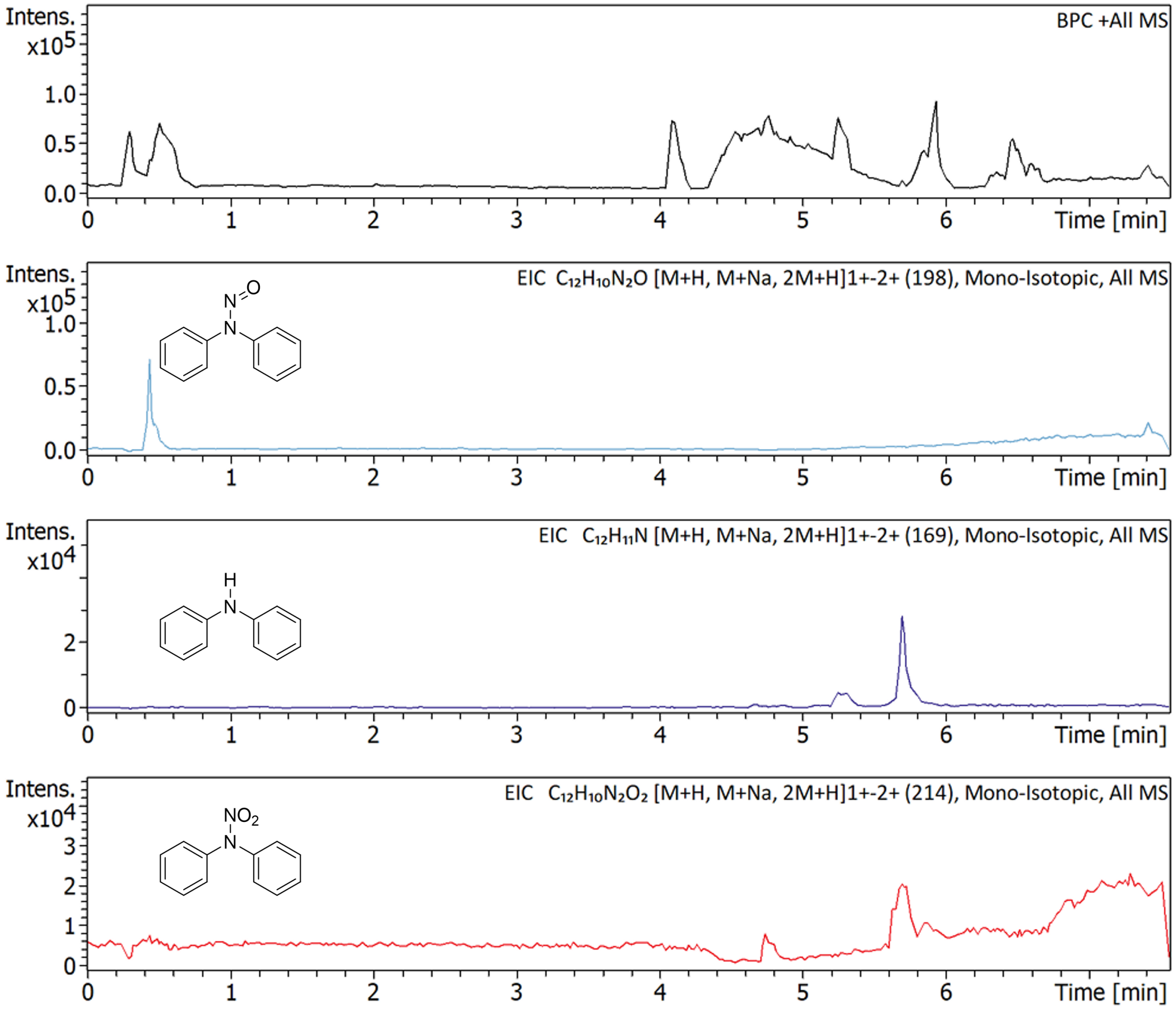

Figure S97: LC-MS/MS reference chromatogram for N-nitrodiphenylamine from reaction condition 2. As referenced in Table S9.

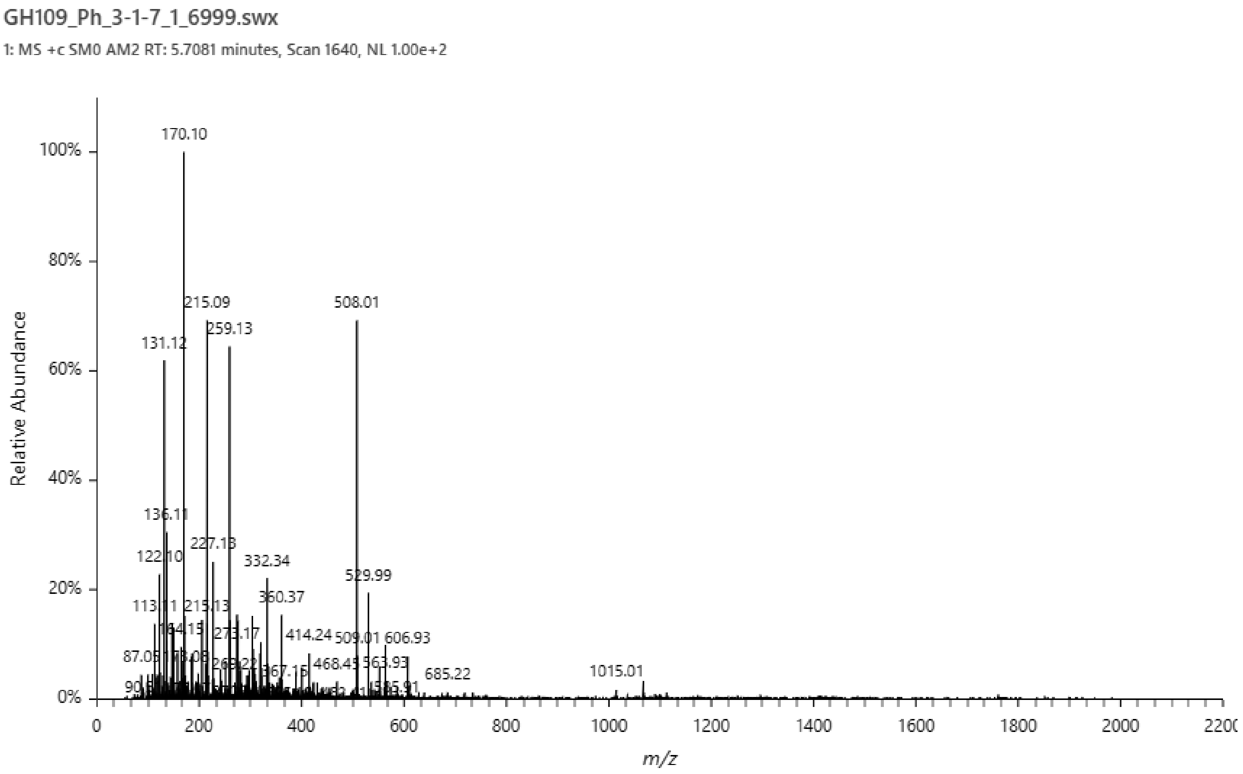

Figure S98: LC-MS/MS reference mass peaks for *N*-nitrodiphenylamine from reaction condition 2. As referenced in Table S9.

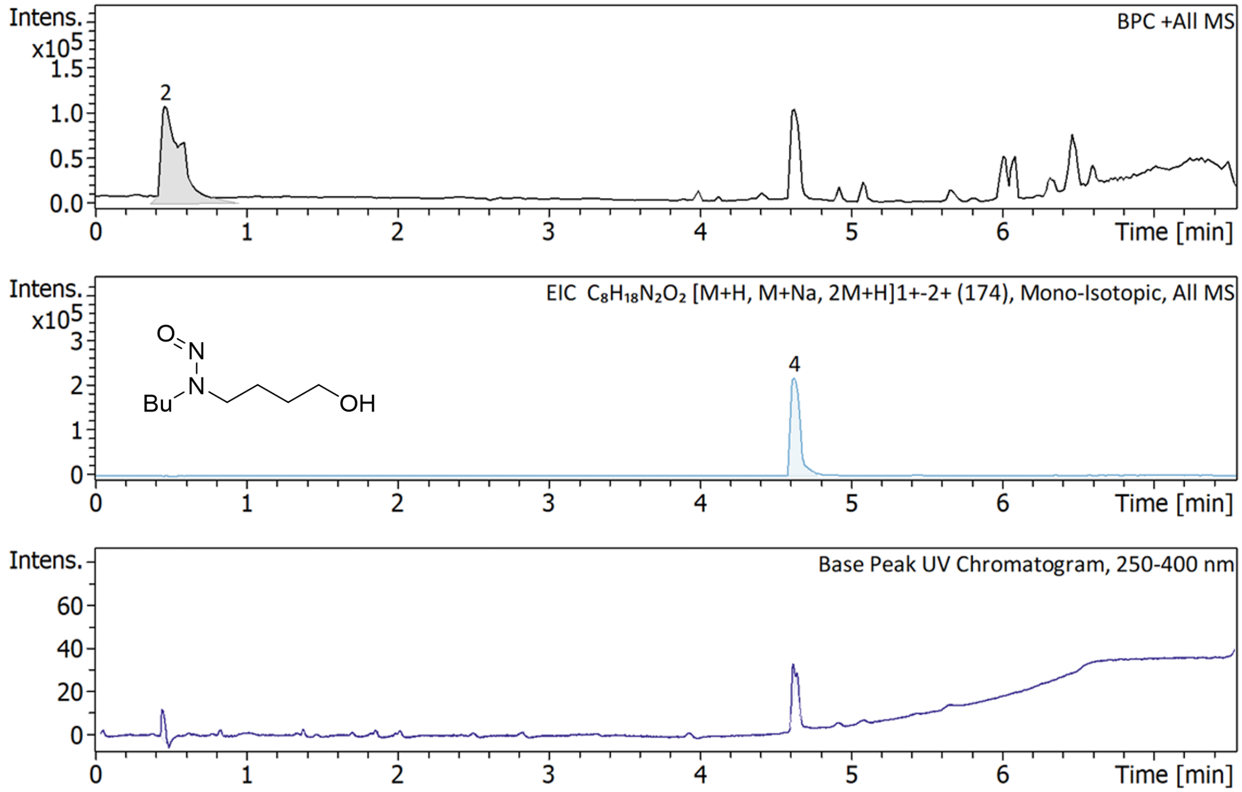

Summary of Results

Name	RT	BPC Area(%)	UV Area(%)	Confirm Formula Results
Cmpd 2, 0.5 min	0.47	100.0	47.0	
Cmpd 4, 4.6 min	4.62	no peak	36.4	C8H18N2O2

Figure S99: LC-MS/MS reference chromatogram for *N*-butyl-*N*-(4-hydroxybutyl)nitrosamine N7. As referenced in Table S9.

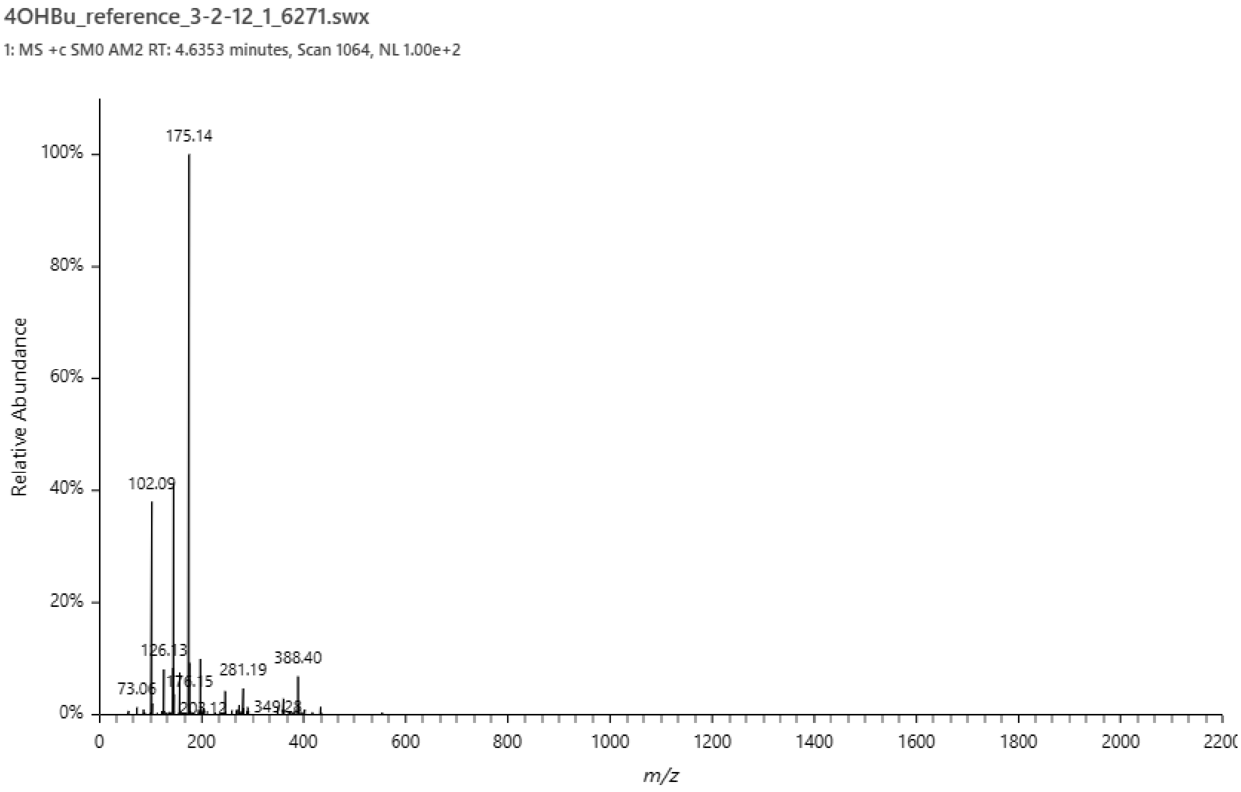

Figure S100: LC-MS/MS reference mass peaks for *N*-butyl-*N*-(4-hydroxybutyl)nitrosamine N7. As referenced in Table S9.

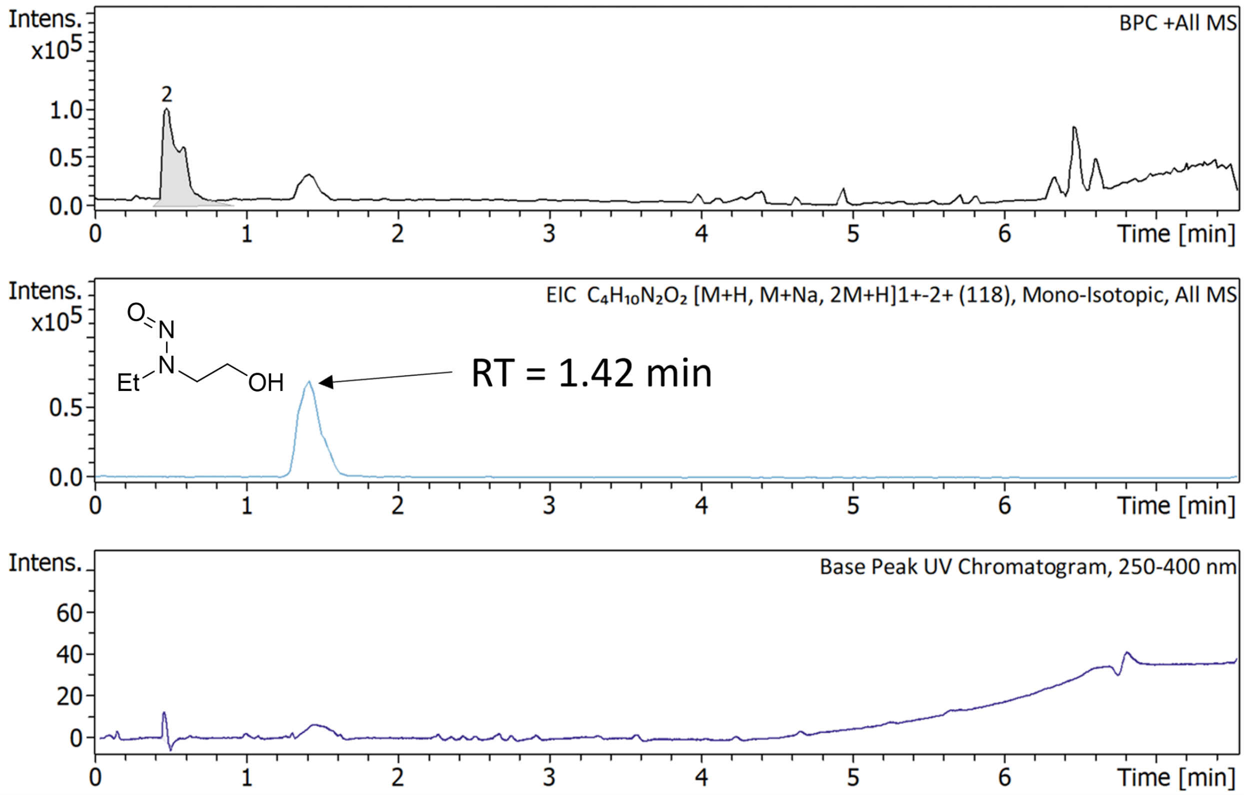

Figure S101: LC-MS/MS reference chromatogram for *N*-ethyl-*N*-(2-hydroxyethyl)nitrosamine N8. As referenced in Table S9.

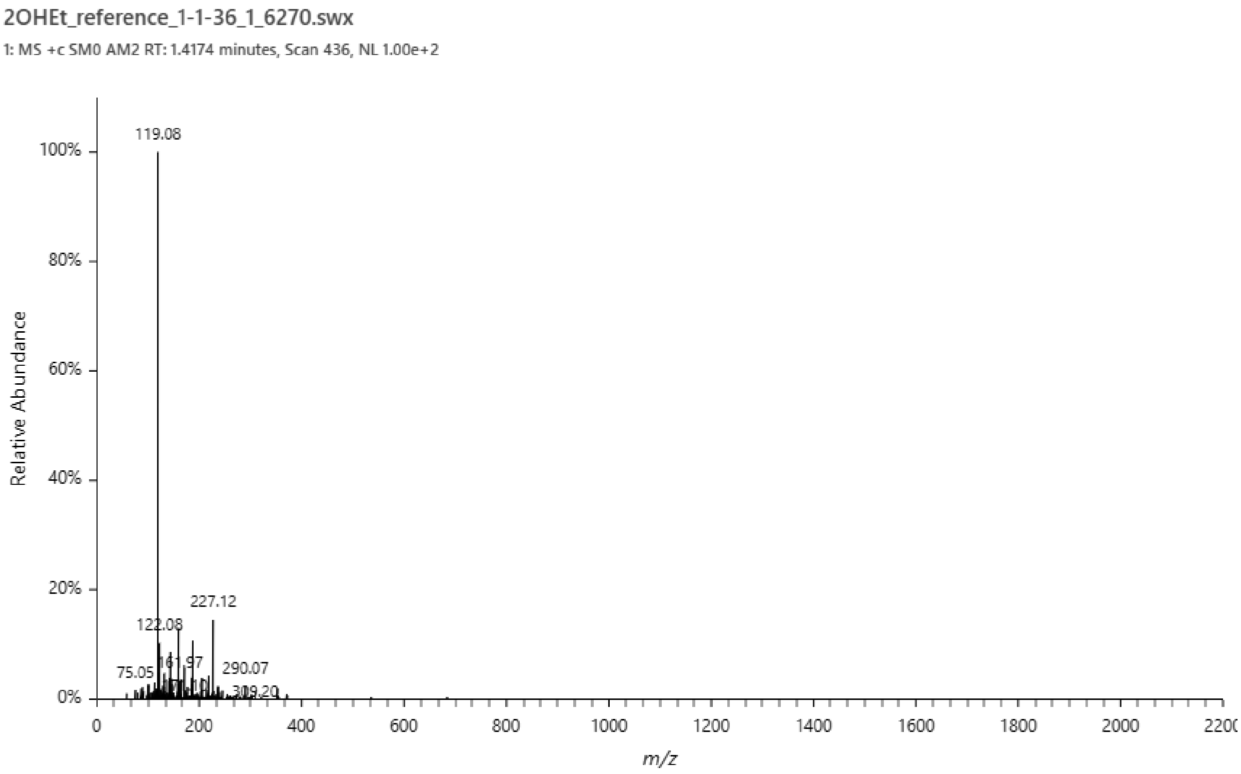

Figure S102: LC-MS/MS reference mass peaks for *N*-ethyl-*N*-(2-hydroxyethyl)nitrosamine N8. As referenced in Table S9.

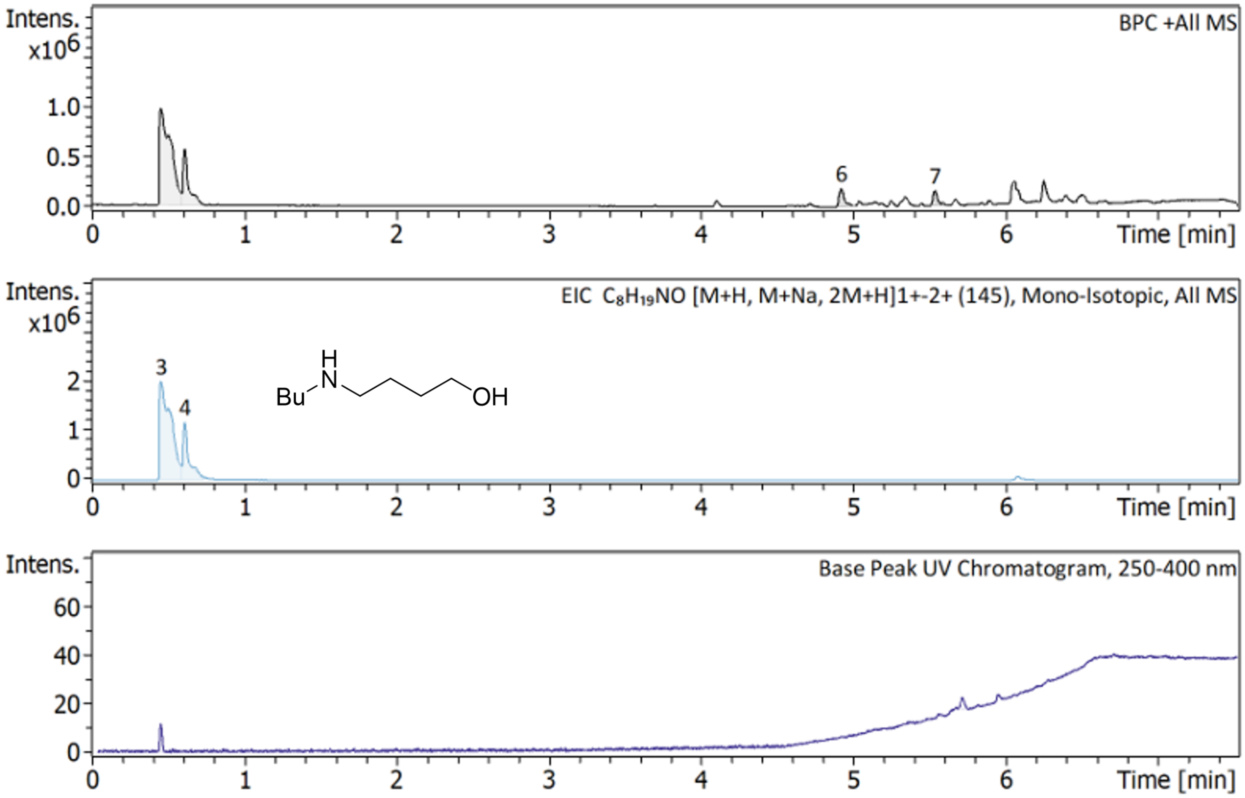

Summary of Results

Name	RT	BPC Area(%)	UV Area(%)	Confirm Formula Results
Cmpd 3, 0.5 min	0.46	70.0	61.4	
Cmpd 4, 0.6 min	0.61	19.2	no peak	
Cmpd 6, 4.9 min	4.92	6.5	no peak	
Cmpd 7, 5.5 min	5.53	4.3	no peak	

Figure S103: LC-MS/MS reference chromatogram for 4-(butylamino)-1-butanol. As referenced in Table S9.

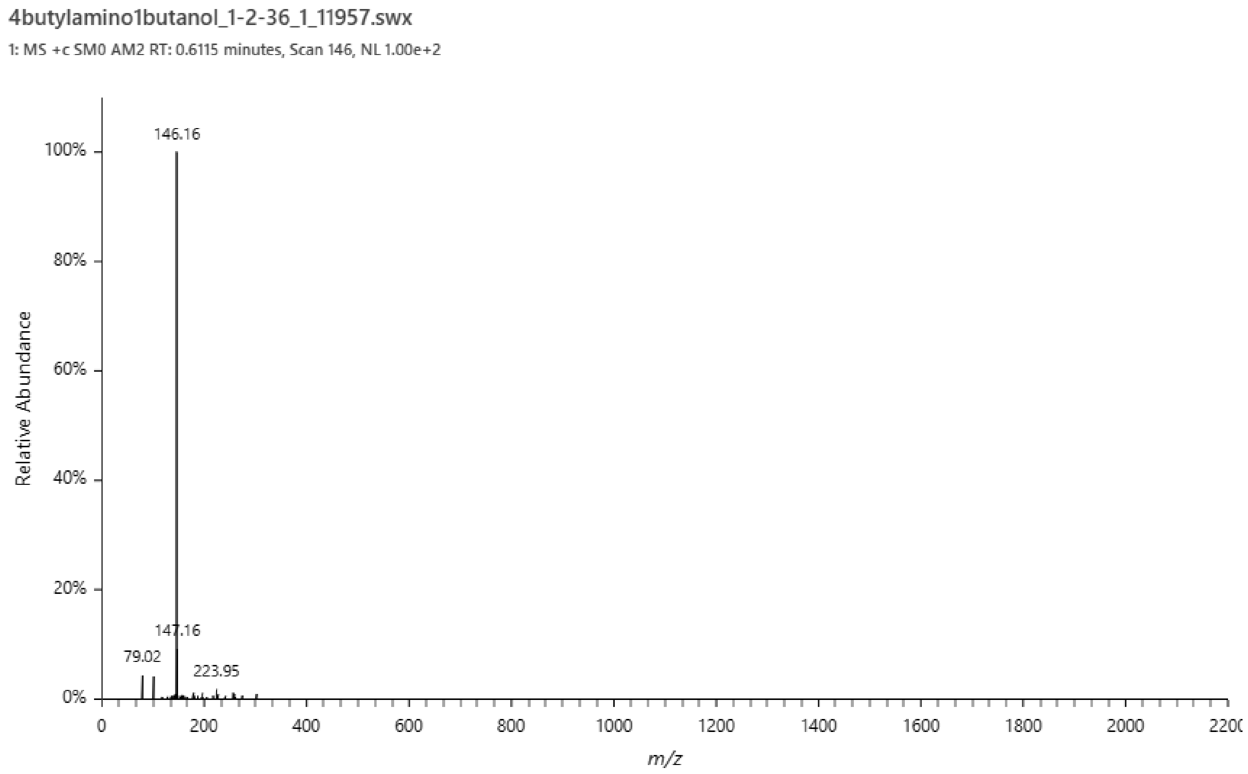

Figure S104: LC-MS/MS reference mass peaks for 4-(butylamino)-1-butanol. As referenced in Table S9.

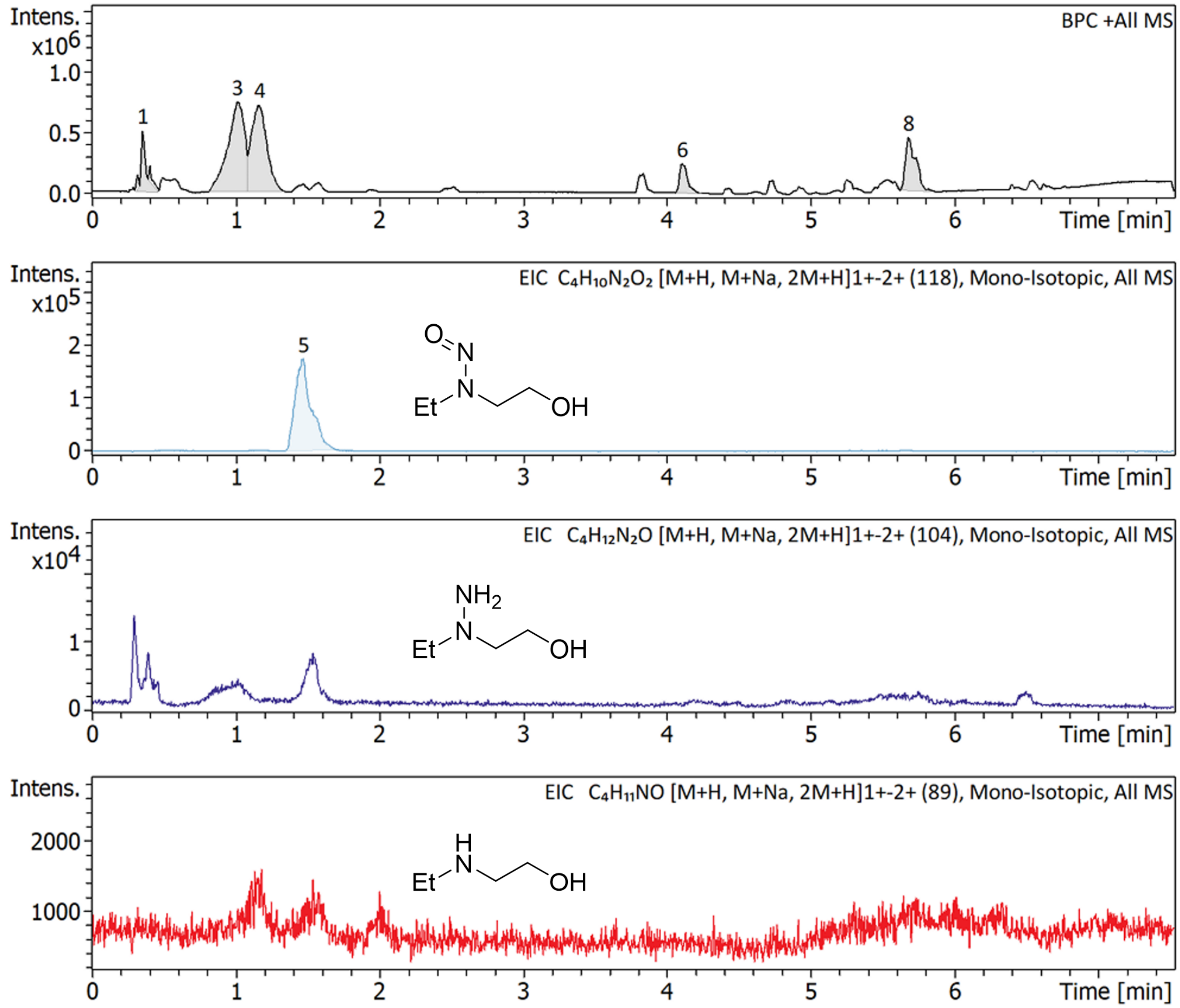

Summary of Results

Name	RT	BPC Area(%)	UV Area(%)	Confirm Formula Results
Cmpd 1, 0.4 min	0.36	7.9	no peak	
Cmpd 3, 1.0 min	1.01	37.9	no peak	
Cmpd 4, 1.2 min	1.17	34.3	no peak	
Cmpd 5, 1.5 min	1.47	no peak	no peak	C4H10N2O2,C4H10.5N2O2
Cmpd 6, 4.1 min	4.11	6.0	no peak	
Cmpd 8, 5.7 min	5.68	13.9	48.8	

Figure S105: LC-MS/MS reference chromatogram for the products of the N8 in condition 9. As referenced in Table S9.

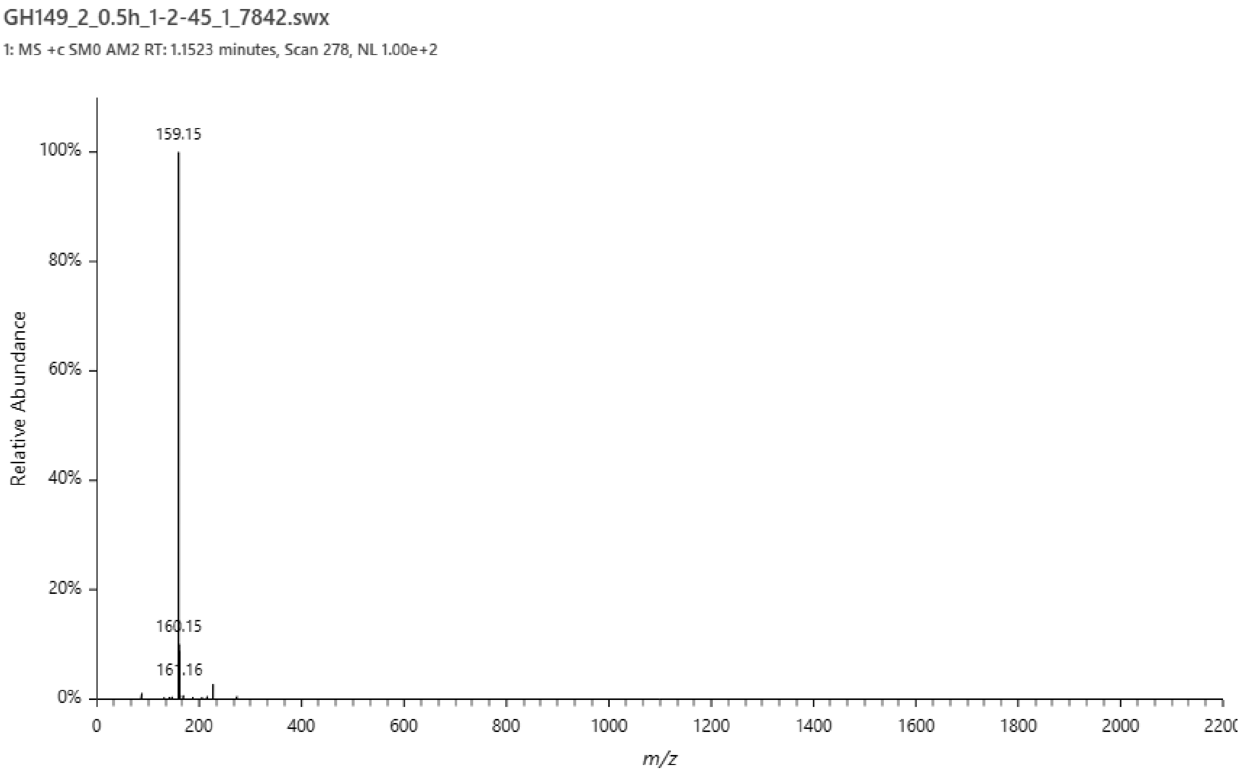

Figure S106: LC-MS/MS reference mass peaks for the products of the N8 in condition 9 (Compounds 3+4). As referenced in Table S9.

8 Flow reaction results

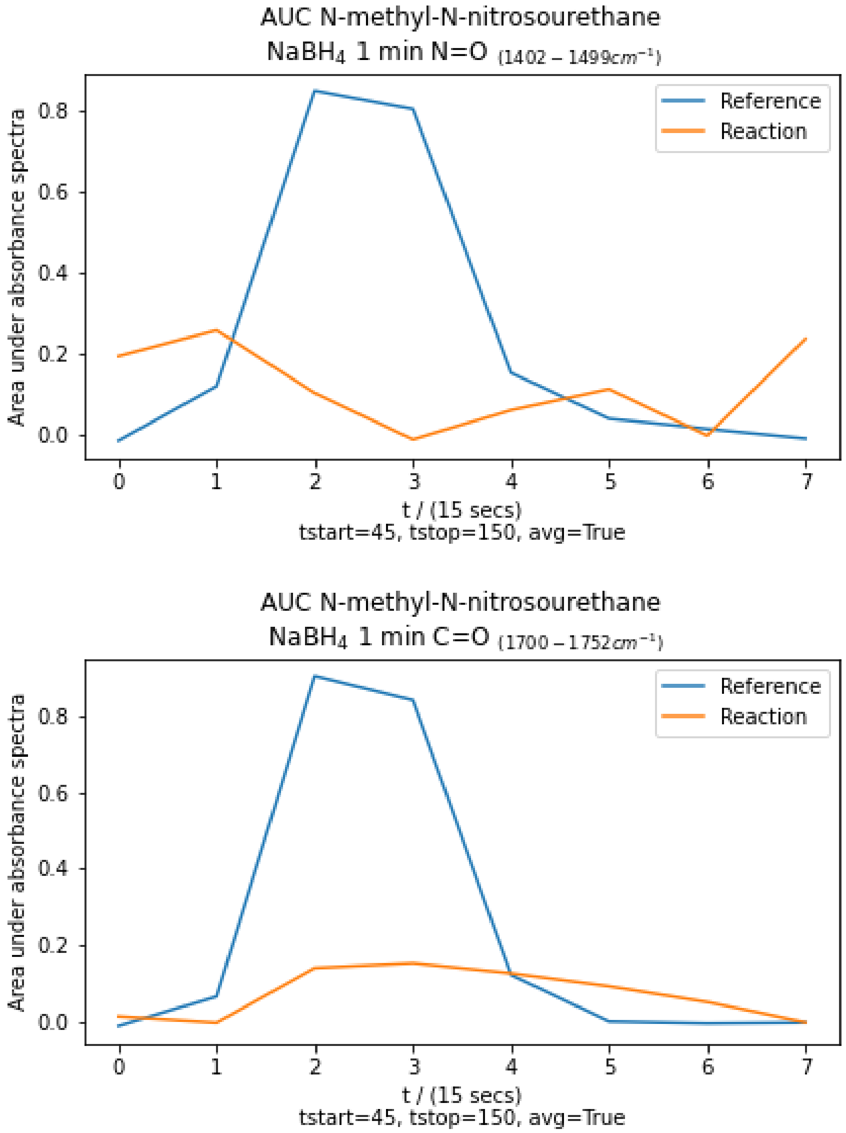

Figure S107: Area under the curve (AUC) plots for the reaction between *N*-methyl-*N*-nitrosourethane (N6) and $NaBH_4$. Showing consumption of both the N=O and C=O bonds.

9 Reaction products by LC-MS/MS and GC-MS/MS

9.1 GC-MS/MS and LC-MS/MS analysis

Reaction product elucidation was achieved via Gas Chromatography with tandem mass spectrometry on the same instrument used to perform the calibrations (detailed in 5.2). The instrument settings were as per Table S6. The method was developed to ensure all *N*-nitrosamines could be captured by the same method.

9.1.1 LC-MS/MS method

In some cases the analysis by GC-MS/MS was insufficient to confirm the reaction products, either if no product was found by GC-MS/MS (e.g. N8 in condition 3) or if the GC-MS/MS method was insufficient to distinguish between reactants and products (N4 in all conditions). In these cases, a high sensitivity liquid chromatography tandem mass-spectrometry (LC-MS/MS) method was employed using a Bruker Impact II QqTOF system with Electrospray Ionisation (ESI). The LC-MS/MS method settings are listed in Table S10.

Table S10: LC-MS/MS method settings.

**Guard Column:**
Waters Acquity Premier CSH C18 1.7um VanGuard FIT 2.1mm x 5mm VanGuard FIT 2.1mm x 5mm
**Column:**
Waters Acquity Premier VanGuard FIT CSH C18 2.1mmx 1000mm
**Method:**
0.7ml/ min A: Water(0.1% formic acid) B: Acetonitrile (0.1% formic acid)
**Gradient (linear interpolation):**

t (mins)	% A
-1.2	99
2.5	95
4.0	70
6.0	5
7.5	99
7.9	99

9.2 Oxidative conditions (1-2):

a)

Key:	0	50	100
conversion (%)			
No data			

No.	Class	Reagents	Solvent	Temp. (°C)	Reaction time (h)	N1	N2	N3	N4	N5	N6	N7	N8
1	Oxidant	H_2O_2 2M	H_2O/EtOH	22	24	2	-3	4	17	0	2	2	5
2	Oxidant	CH_3CO_3H	EtOH	22	24	8	8	7	95	14	101	14	12

b)

Key:	
Found	
Absent	
No Data	

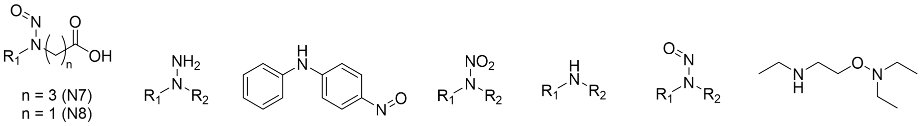

CA H P O A N D

	N1				N2				N3				N4					N5			N6		N7				N8				
No.	N	H	A	O	N	H	A	O	N	H	A	O	N	H	A	P	O	N	H	A	N	A	N	H	A	CA	N	H	A	CA	D
1	Found	Absent	Absent	Absent	Found	Absent	Absent	Absent	Found	Absent	Absent	Absent	Found	Absent	Found	Absent	Absent	Found	Absent	Absent	Absent	Found	Found	Absent	Absent	Absent	Found	Absent	Absent	Absent	Absent
2	Found	Absent	Absent	Found	Found	Absent	Absent	Found	Found	Absent	Absent	Absent	Absent	Absent	Absent	Absent	Found	Found	Absent	Absent	Absent	Found	Found	Absent	Absent	Absent	Found	Absent	Absent	Absent	Absent

Figure S108: Summary tables for the results of the reactivity screen for conditions 1 and 2 showing a) The conversions with respect to the *N*-nitrosamine and b) The products of the reactions determined by GC-MS/MS and LC-MS/MS.

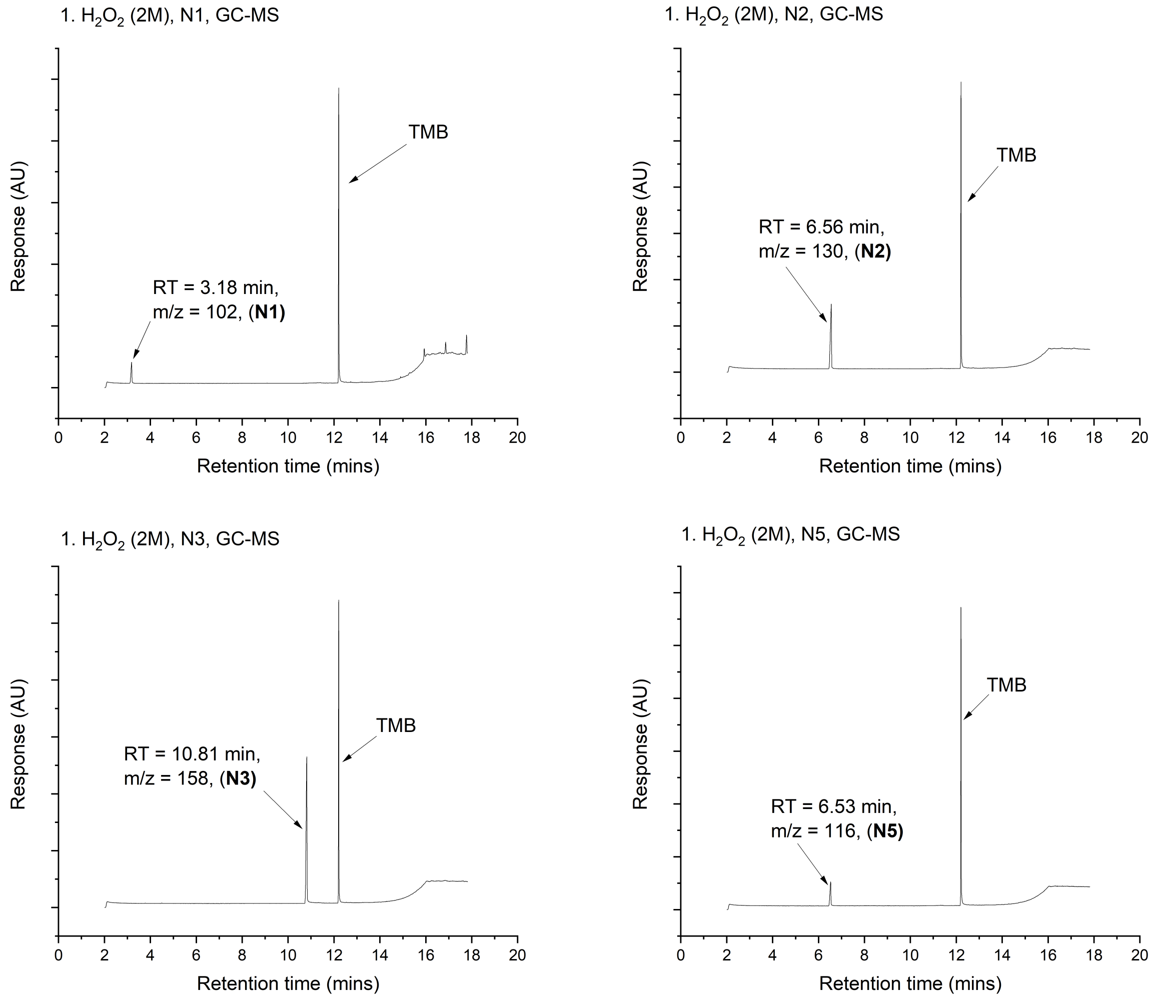

Figure S109: GC-MS/MS plots for N1, N2, N3 and N5 condition 1. As referenced in Section 3.3

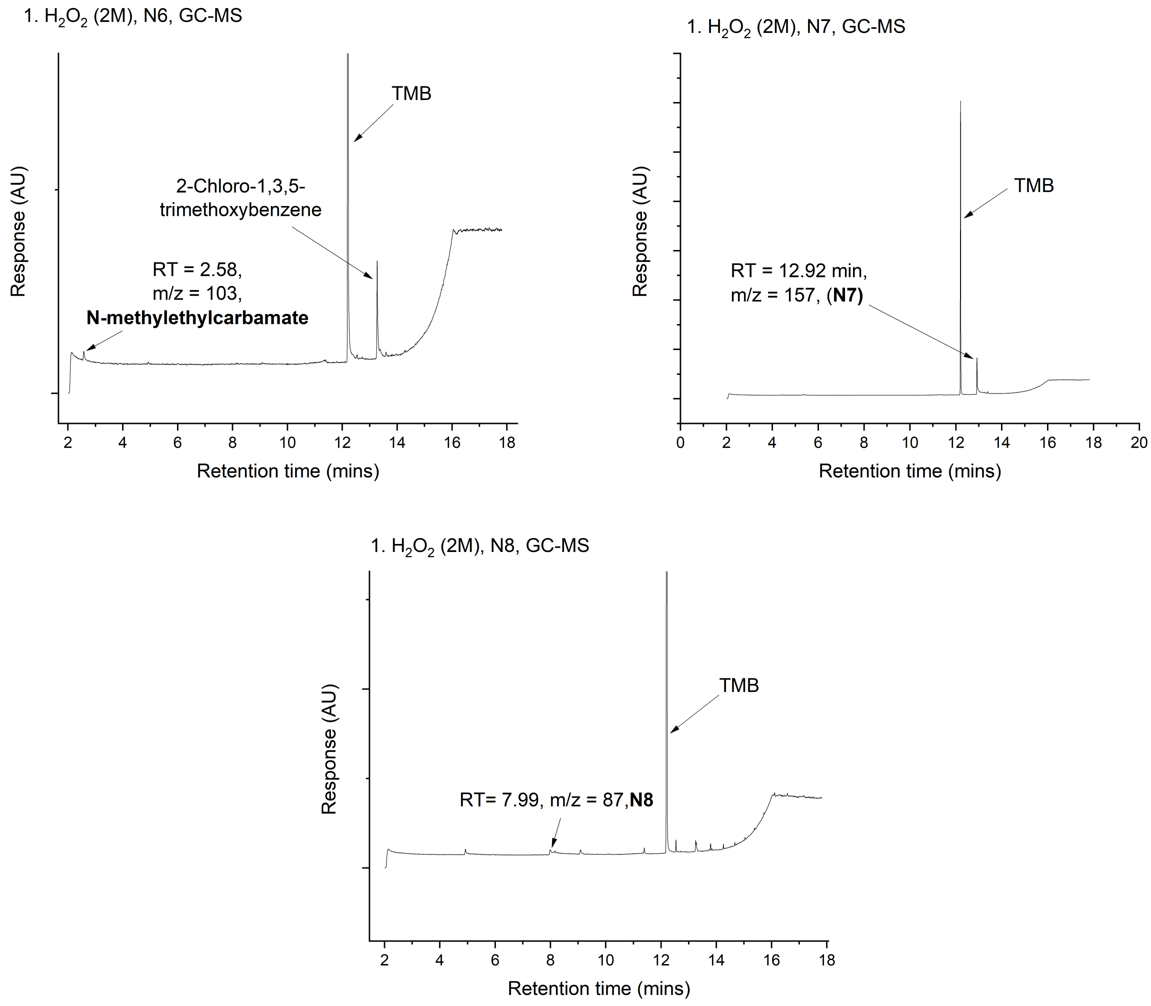

Figure S110: GC-MS/MS plots for N6-8 for condition 1. As referenced in Section 3.3

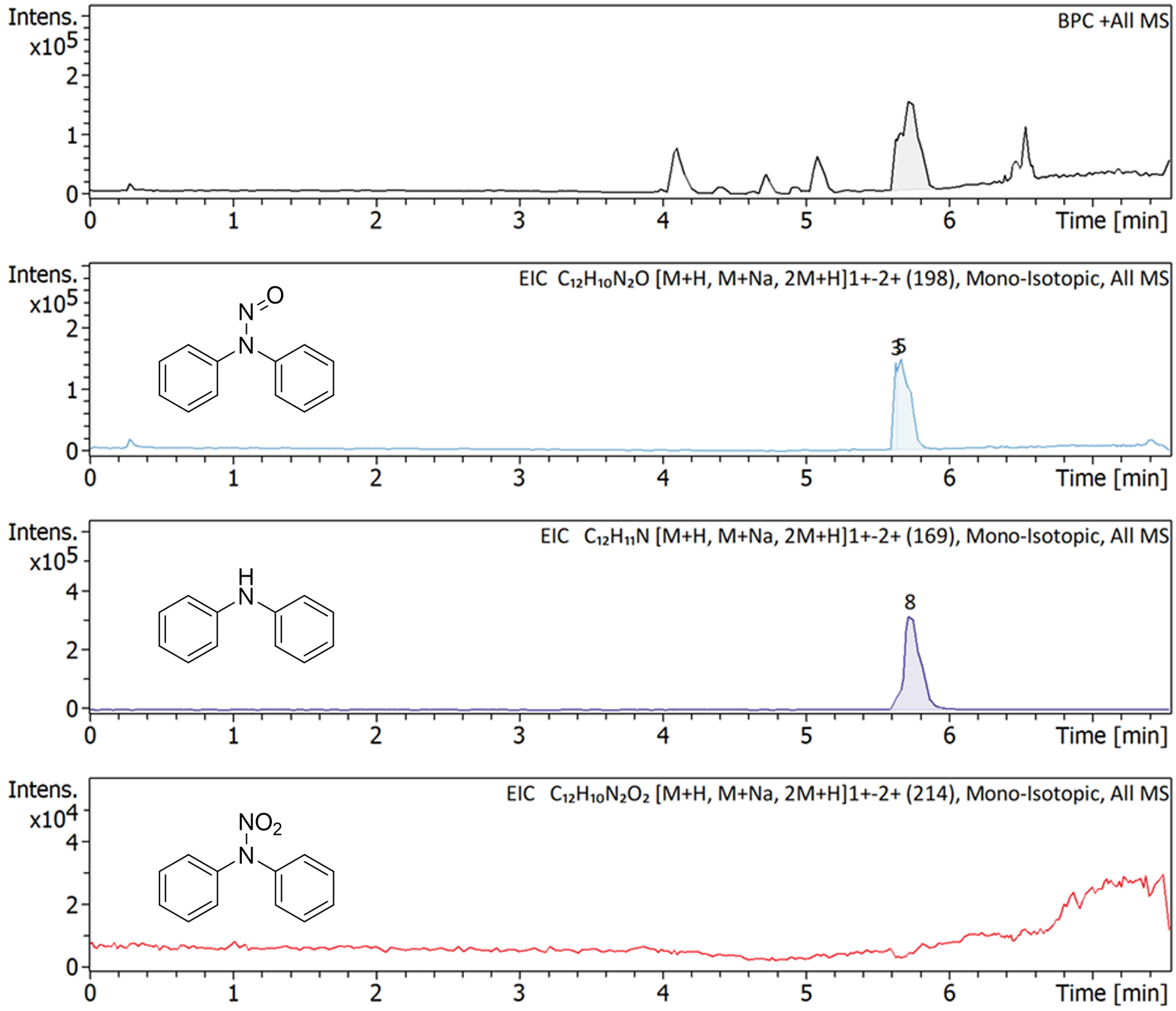

Summary of Results

Name	RT	BPC Area(%)	UV Area(%)	Confirm Formula Results
Cmpd 3, 5.6 min	5.63	no peak	27.9	C12H10N2O
Cmpd 5, 5.7 min	5.66	100.0	27.9	C12H10N2O,C12H11N
Cmpd 8, 5.7 min	5.72	100.0	15.9	C12H10N2O,C12H11N

Figure S111: LC-MS/MS results for N4 in condition 1. The products of this reaction were validated by ^{1}H and ^{13}C NMR in Section 10.

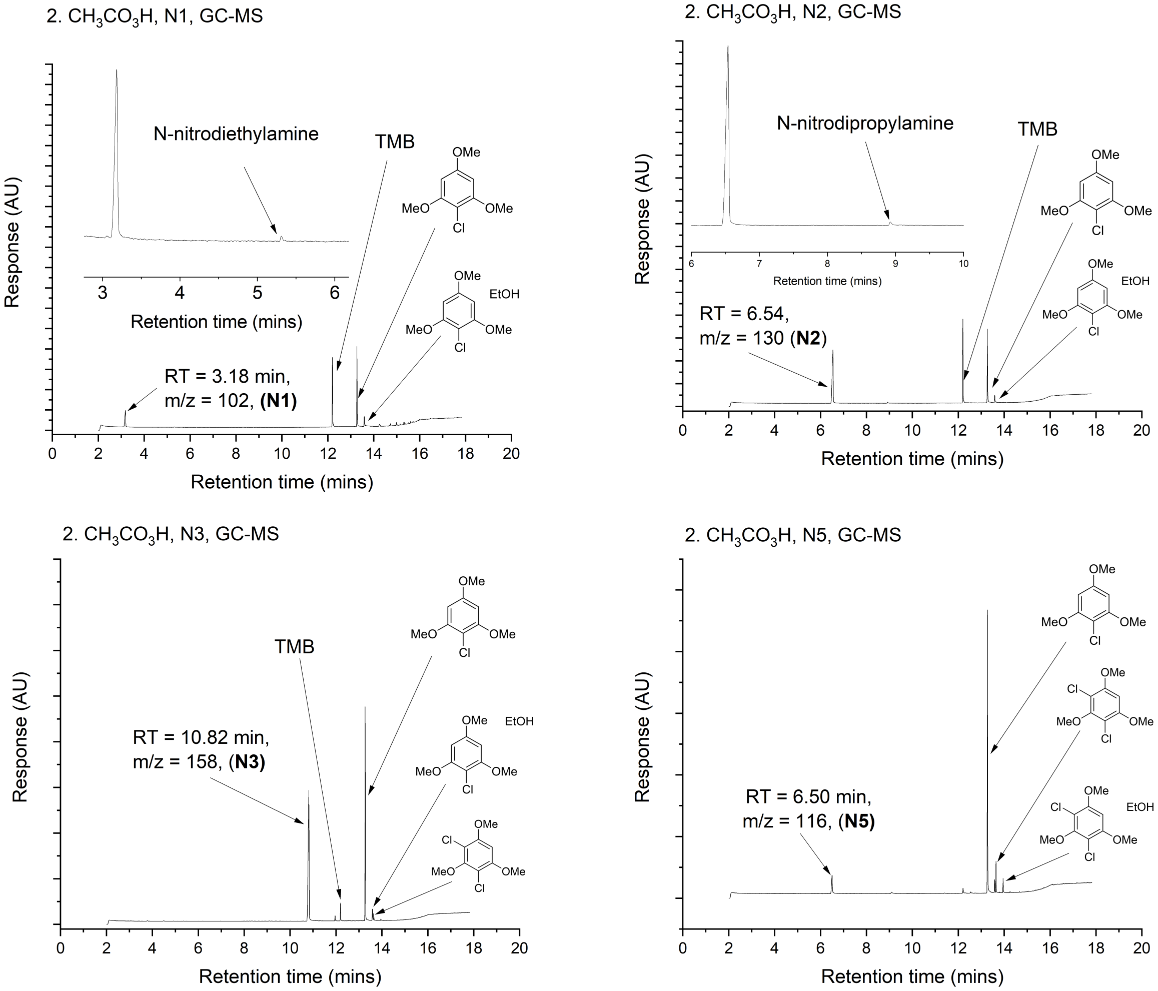

Figure S112: GC-MS/MS plots for N1, N2, N3 and N5 condition 2. As referenced in Section 3.3

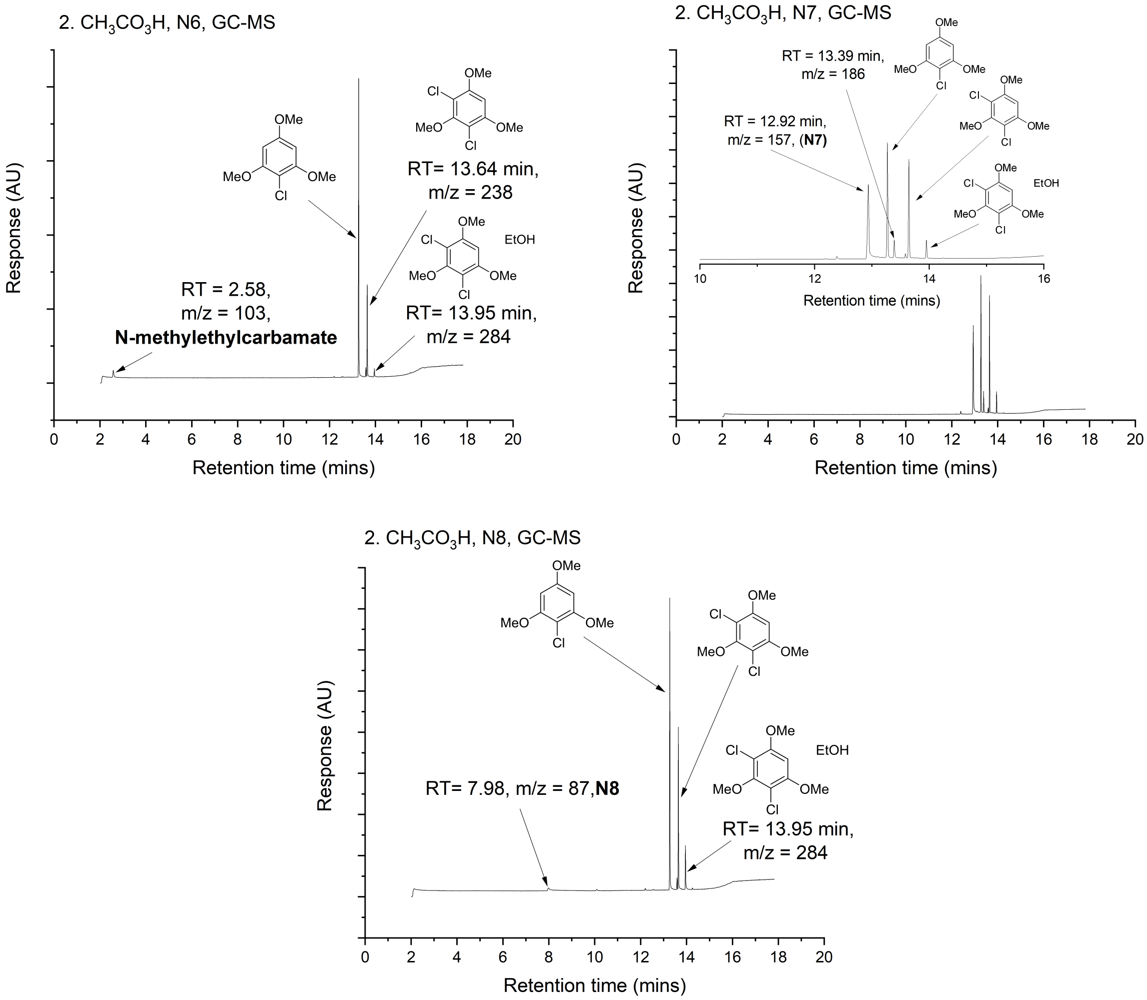

Figure S113: GC-MS/MS plots for N6-8 for condition 2. As referenced in Section 3.3

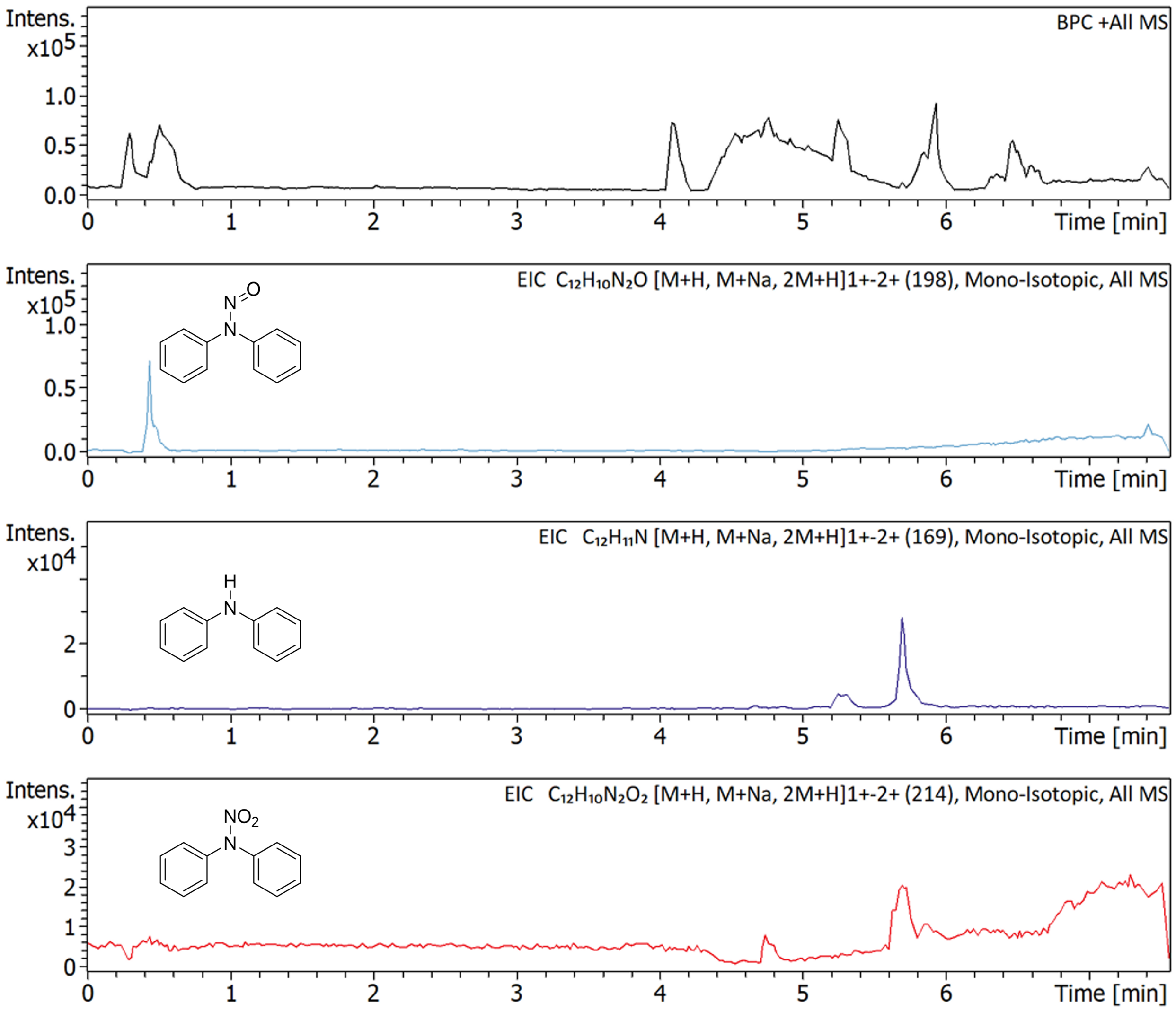

Figure S114: LC-MS/MS results for N4 in condition 2.

9.3 Acidic conditions (3-7):

a)

Key:	0	50	100
conversion (%)			
No data			

No.	Class	Reagents	Solvent	Temp. (°C)	Reaction time	N1	N2	N3	N4	N5	N6	N7	N8
3	Acid	HBr 33 % wt.	AcOH/EtOH	22	24	6	7	13	42	25	85	2	26
4	Acid	HCl 2M	H_2O/EtOH	22	24	2	-5	6	37	-1	101	-12	6
5	Acid	HCl 2M	H_2O/EtOH	50	16	1	-4	13	86	5	101	-14	6
6	Acid	HCl 2M	EtOH	22	24	-2	-2	3	100	56	101	-6	15
7	Acid	HCl 2M	EtOH	50	16	71	54	56	99	98	101	52	100

b)

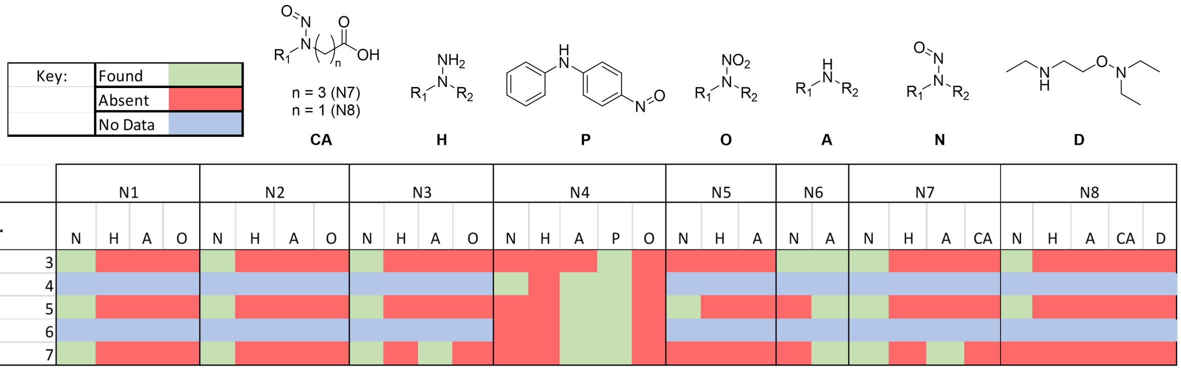

Figure S115: Reactivity of N-nitrosamines under acidic conditions 3-7. a) Conversions with respect to the N-nitrosamine and b) Products of the reactions determined by GC-MS/MS and LC-MS/MS.

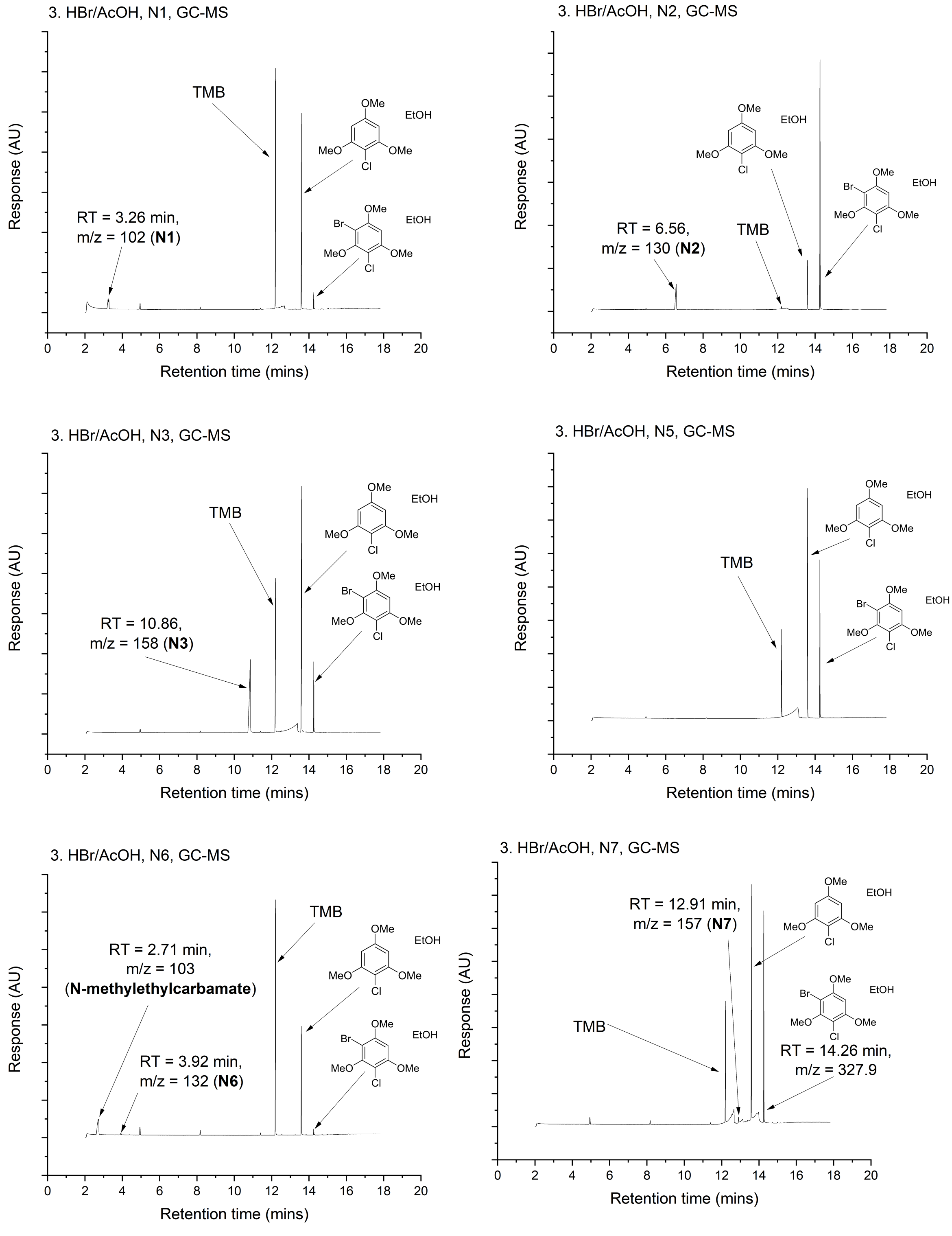

Figure S116: GC-MS/MS plots for N1, N2, N3, and N5-7 for condition 3. As referenced in Section 3.3

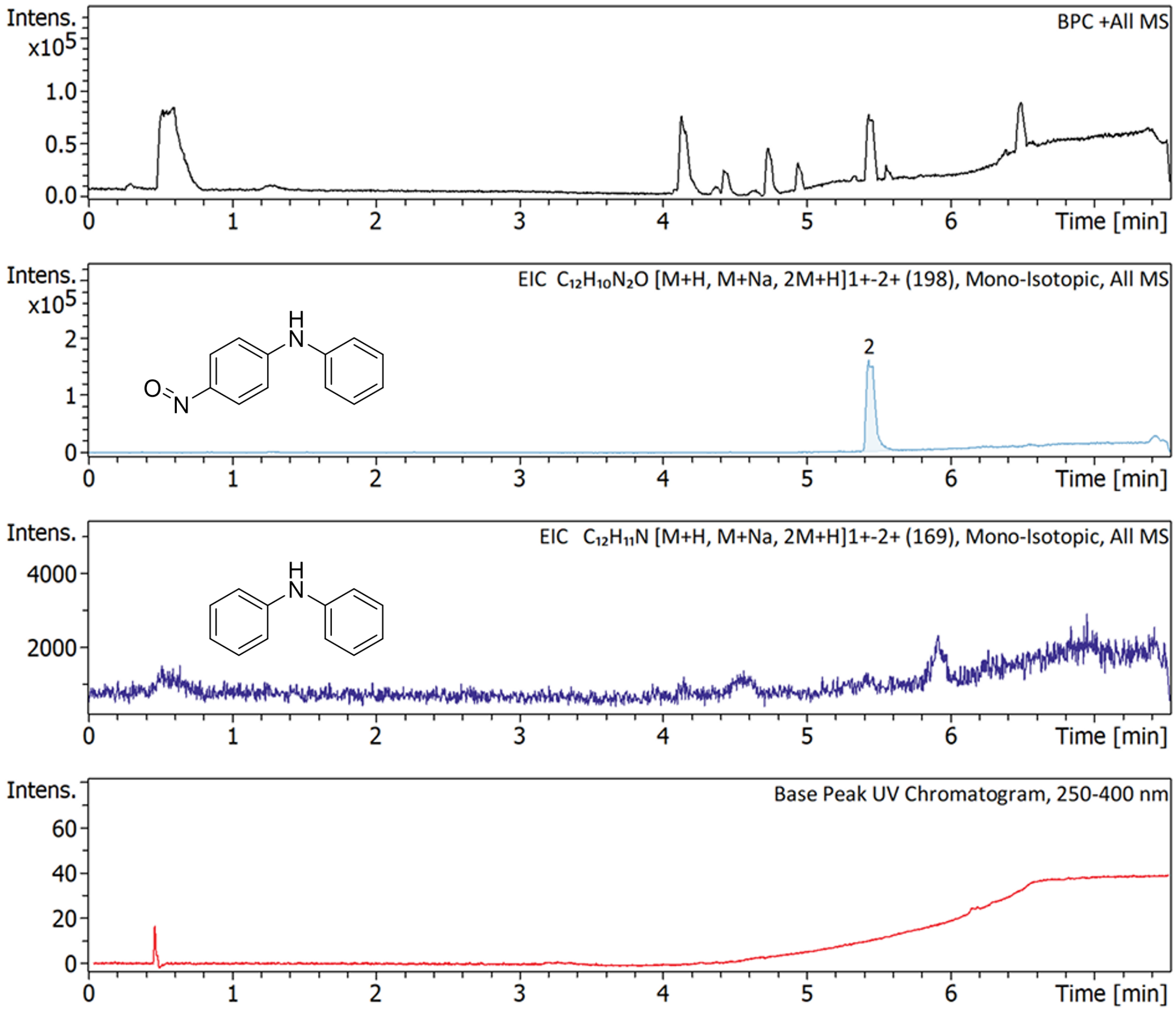

Summary of Results

Name	RT	BPC Area(%)	UV Area(%)	Confirm Formula Results
Cmpd 2, 5.4 min	5.43	no peak	no peak	C12H10N2O

Figure S117: LC-MS/MS experiment chromatogram for *N*-nitrosodiphenylamine (N4) in reaction condition 3. Referenced in Section 3.3.

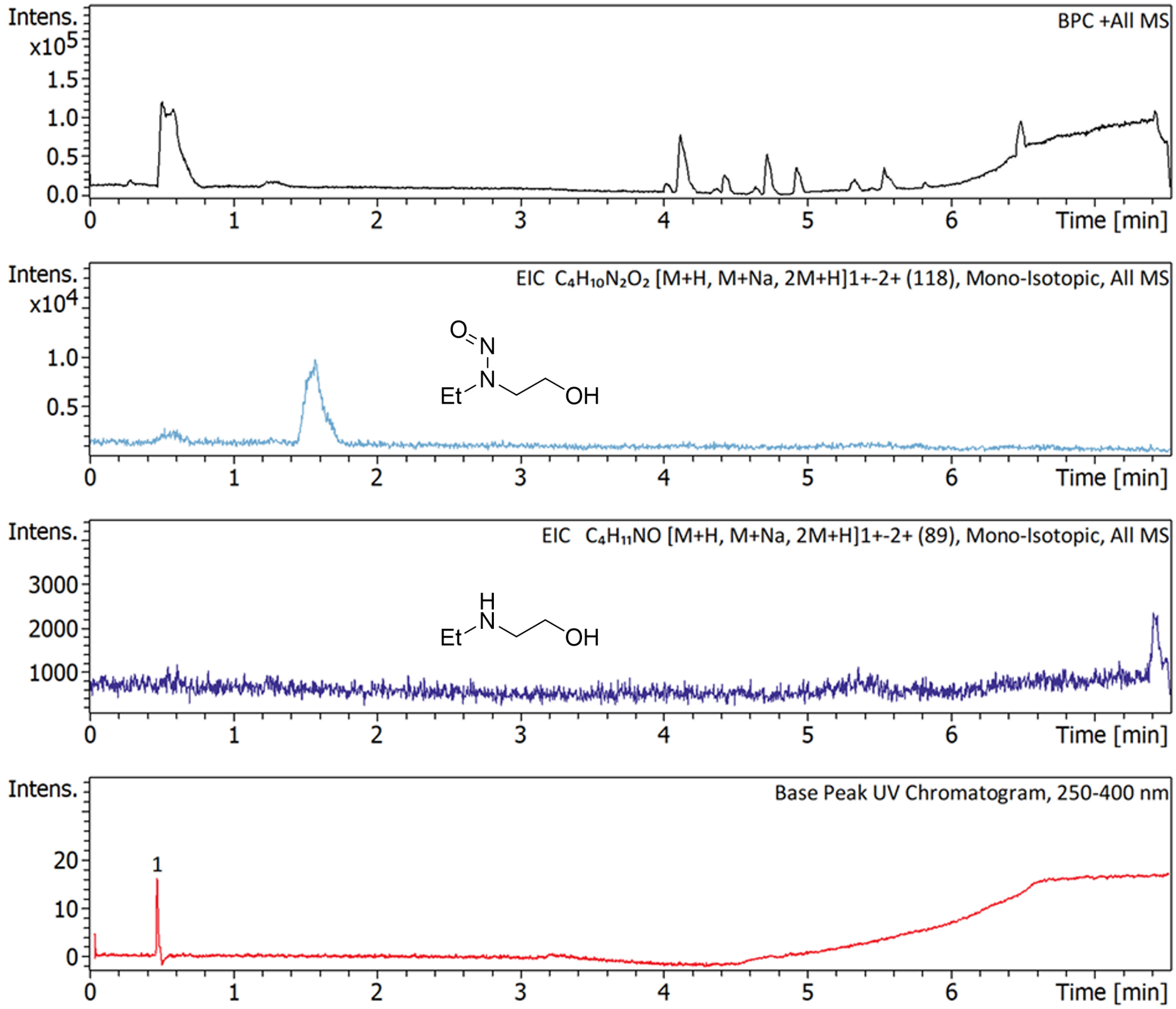

Summary of Results

Name	RT	BPC Area(%)	UV Area(%)	Confirm Formula Results
Cmpd 1, 0.5 min	0.47	no peak	no uv	

Figure S118: LC-MS/MS experiment chromatogram for *N*-ethyl-*N*-(2-hydroxyethyl)nitrosamine (N8) in reaction condition 3. Referenced in Section 3.3.

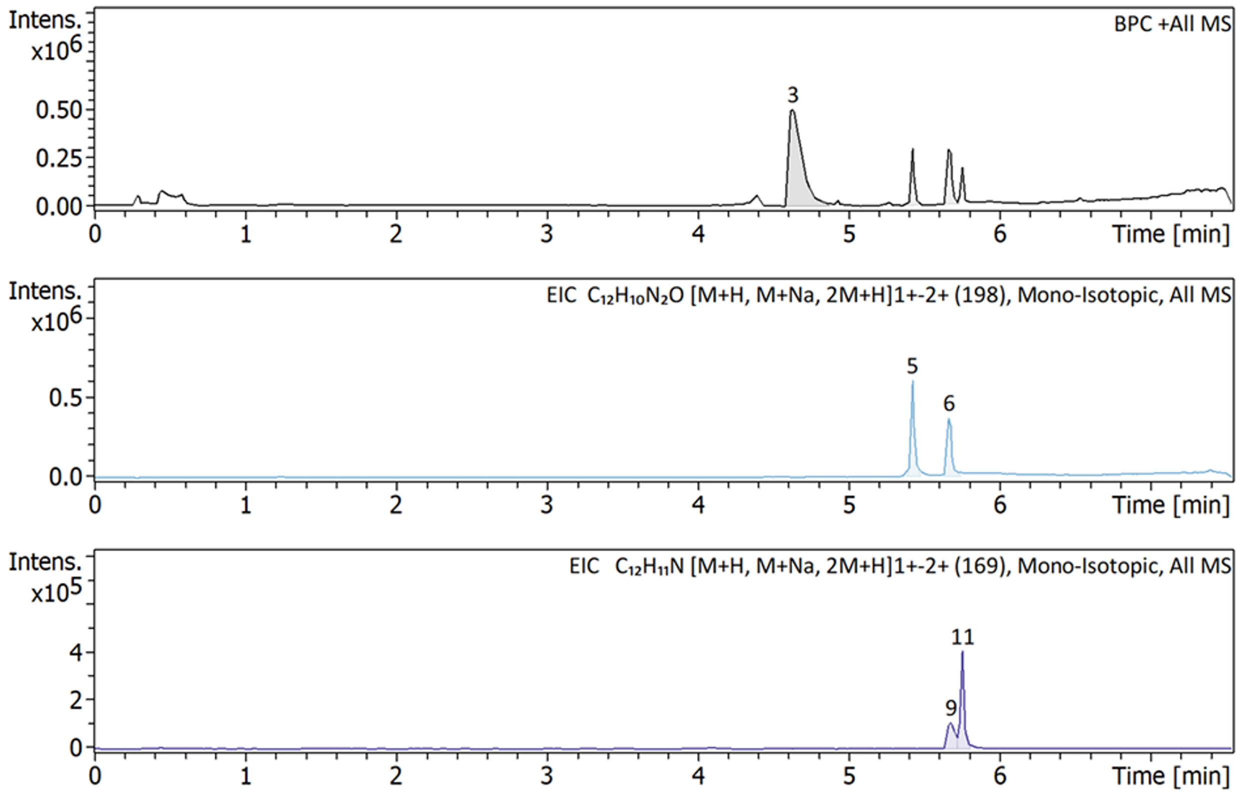

Summary of Results

Name	RT	BPC Area(%)	UV Area(%)	Confirm Formula Results
Cmpd 3, 4.6 min	4.63	72.8	no peak	
Cmpd 5, 5.4 min	5.42	10.9	no peak	C12H10N2O
Cmpd 6, 5.7 min	5.66	16.3	78.2	C12H10N2O
Cmpd 9, 5.7 min	5.67	16.3	78.2	C12H10N2O
Cmpd 11, 5.8 min	5.75	no peak	4.3	C12H11N

Figure S119: LC-MS/MS experiment chromatogram for *N*-nitrosodiphenylamine (N4) in reaction condition 4. Referenced in Section 3.3.

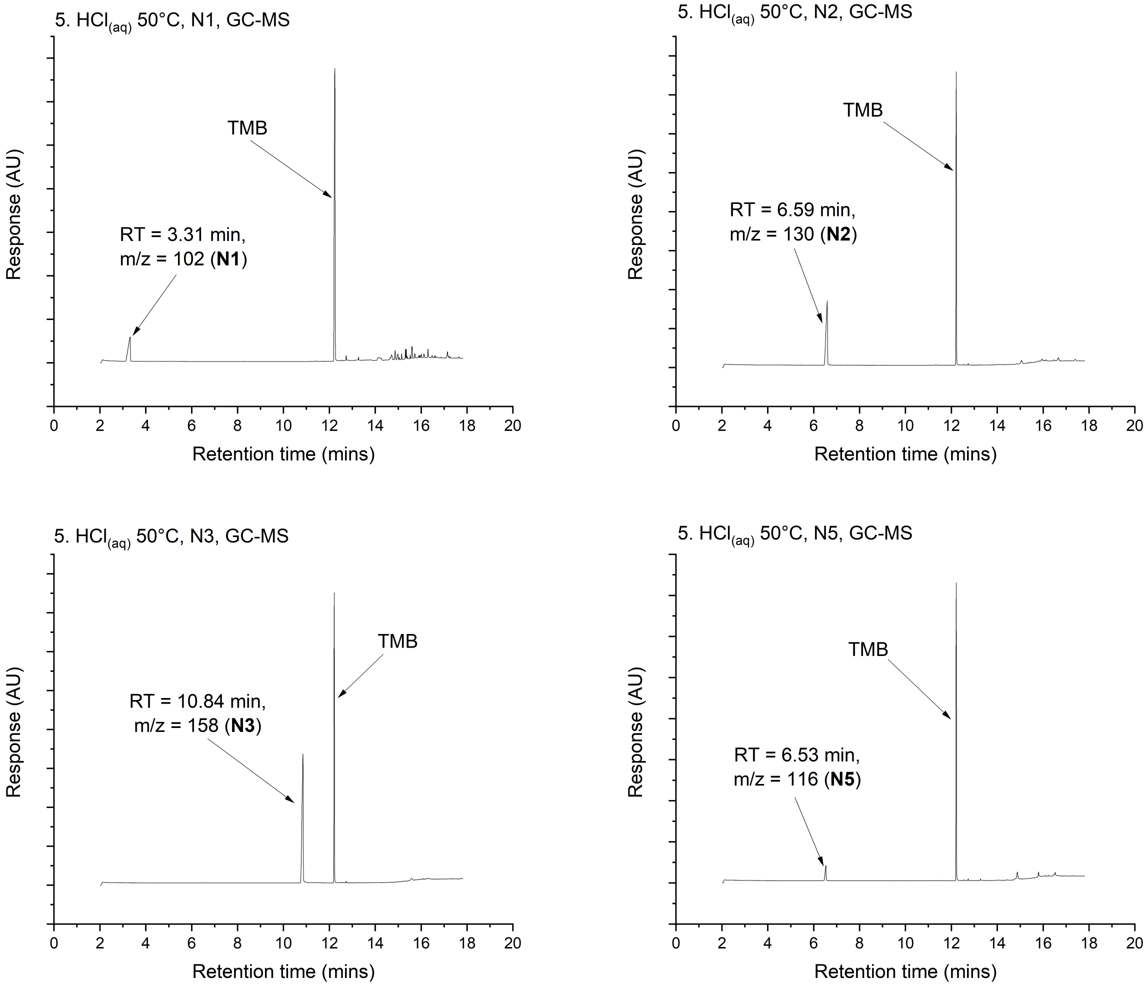

Figure S120: GC-MS/MS plots for N1, N2, N3 and N5 condition 5. As referenced in Section 3.3

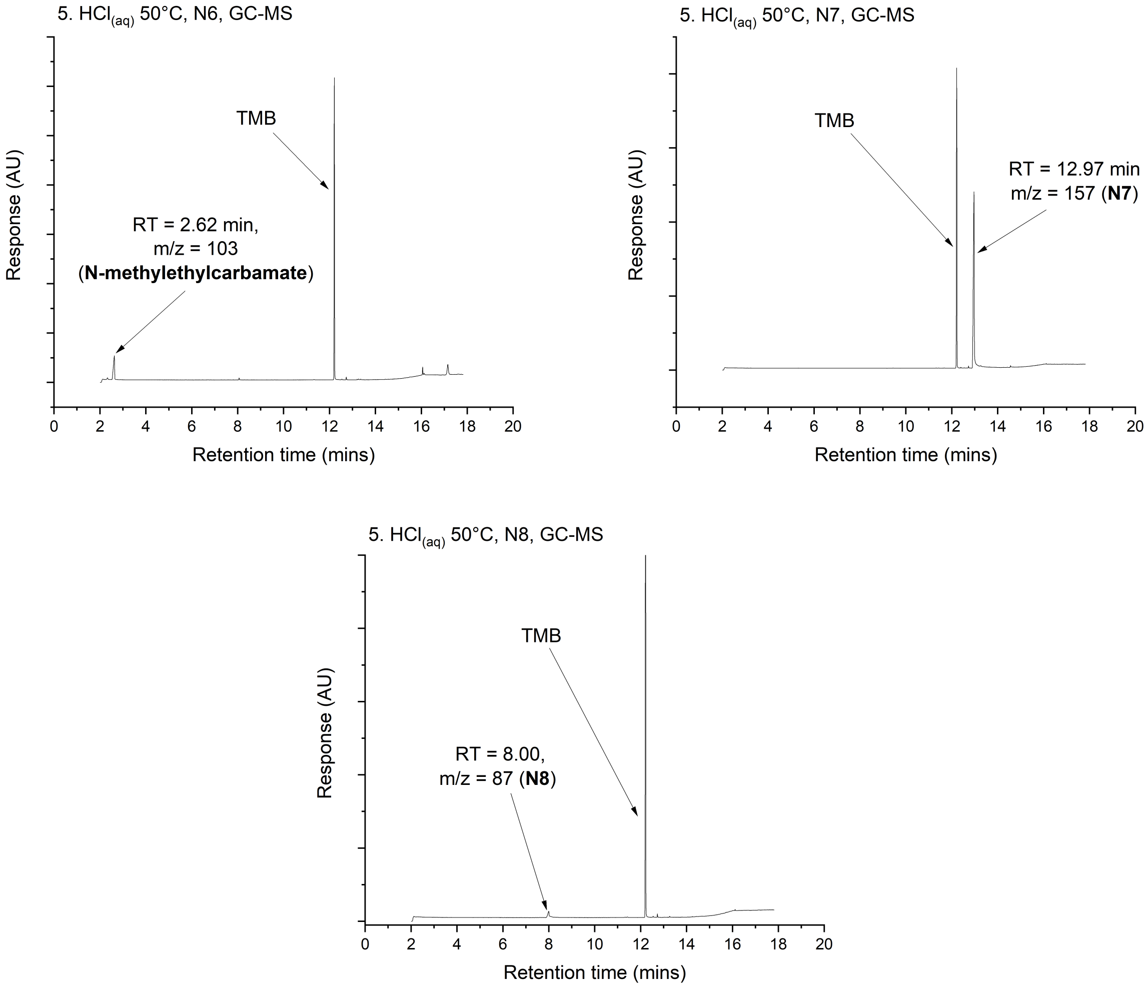

Figure S121: GC-MS/MS plots for N6-8 for condition 5. As referenced in Section 3.3

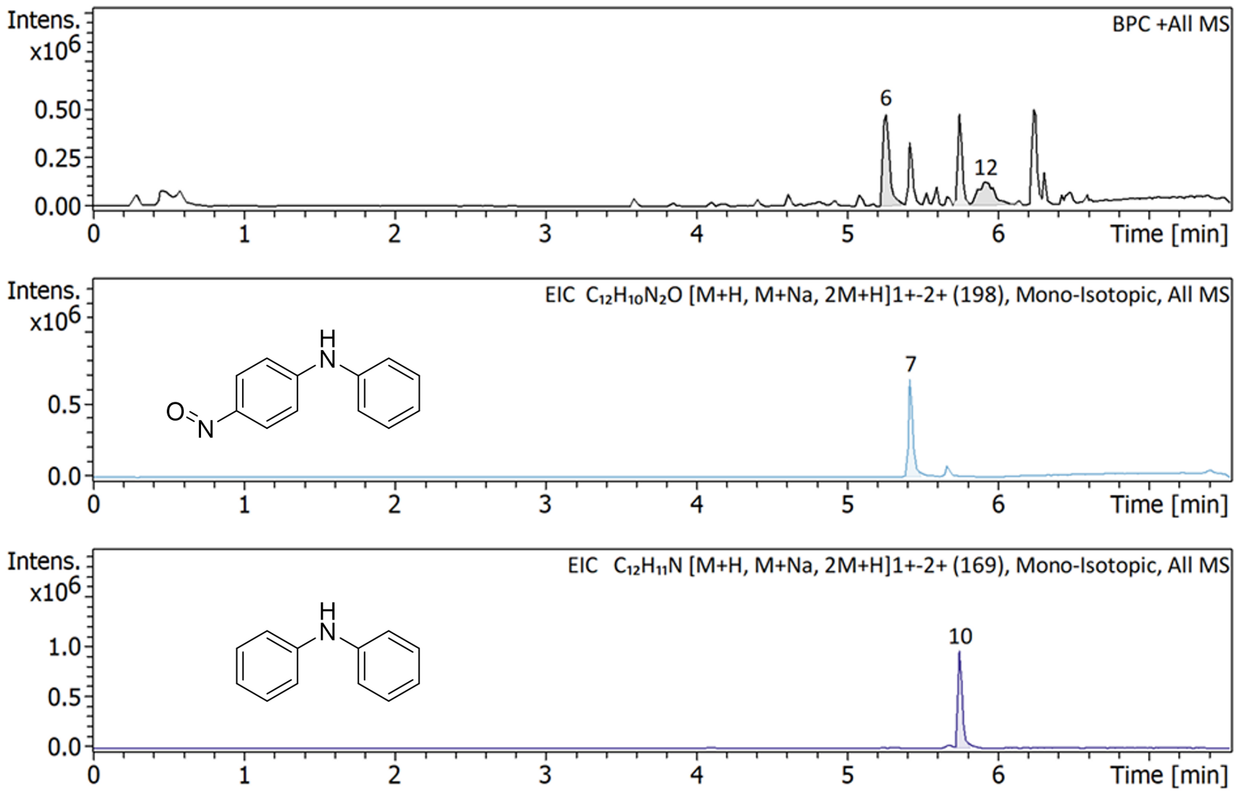

Summary of Results

Name	RT	BPC Area(%)	UV Area(%)	Confirm Formula Results
Cmpd 6, 5.3 min	5.26	44.2	no peak	
Cmpd 7, 5.4 min	5.42	no peak	no peak	C12H10N2O
Cmpd 10, 5.7 min	5.75	28.6	34.5	C12H11N
Cmpd 12, 5.9 min	5.92	27.2	no peak	

Figure S122: LC-MS/MS experiment chromatogram for *N*-nitrosodiphenylamine (N4) in reaction condition 5. Referenced in Section 3.3.

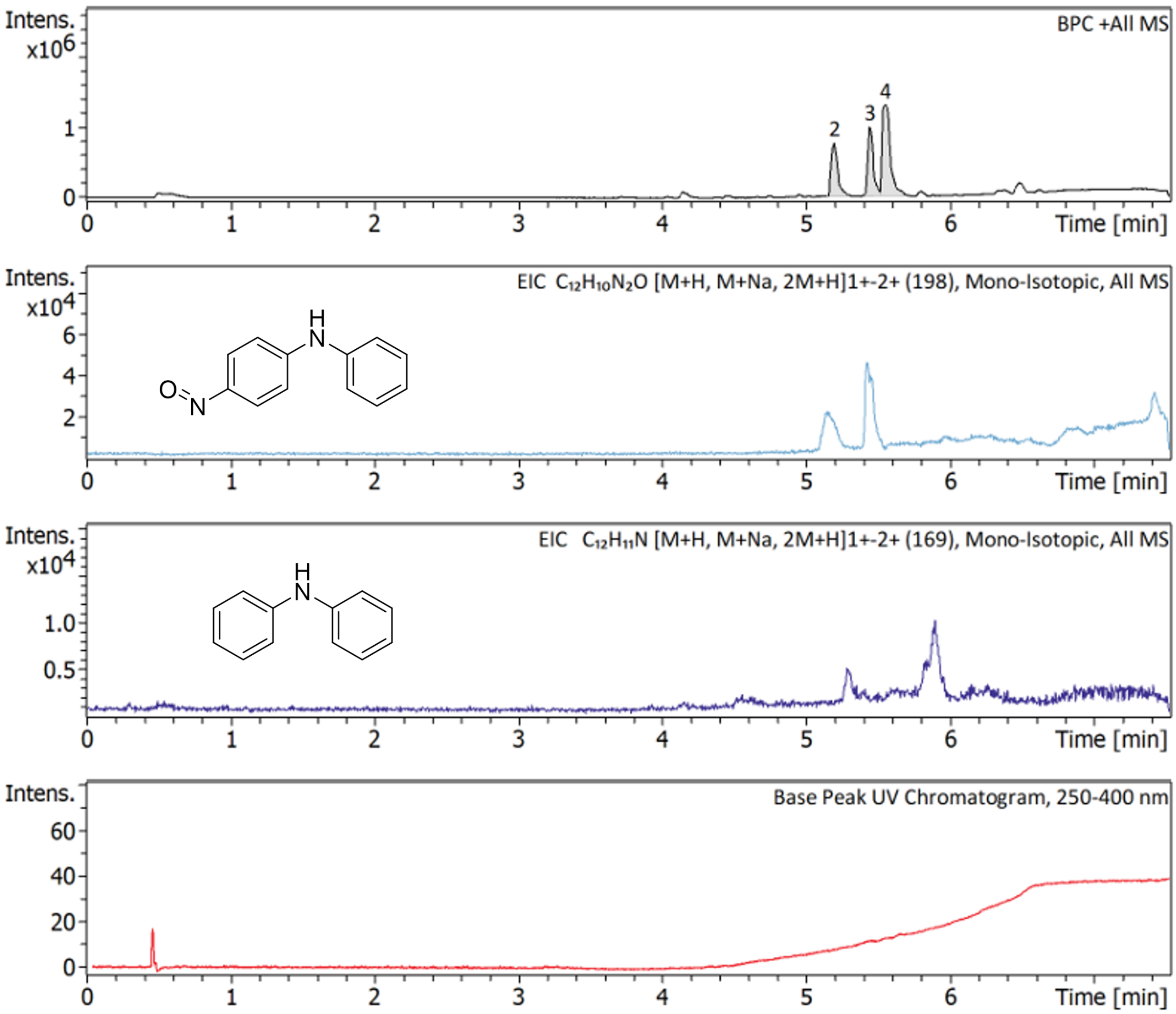

Summary of Results

Name	RT	BPC Area(%)	UV Area(%)	Confirm Formula Results
Cmpd 2, 5.2 min	5.19	23.2	no peak	
Cmpd 3, 5.4 min	5.44	25.9	no peak	C12H10N2O
Cmpd 4, 5.5 min	5.55	50.8	no peak	

Figure S123: LC-MS/MS experiment chromatogram for *N*-nitrosodiphenylamine (N4) in reaction condition 6. Referenced in Section 3.3.

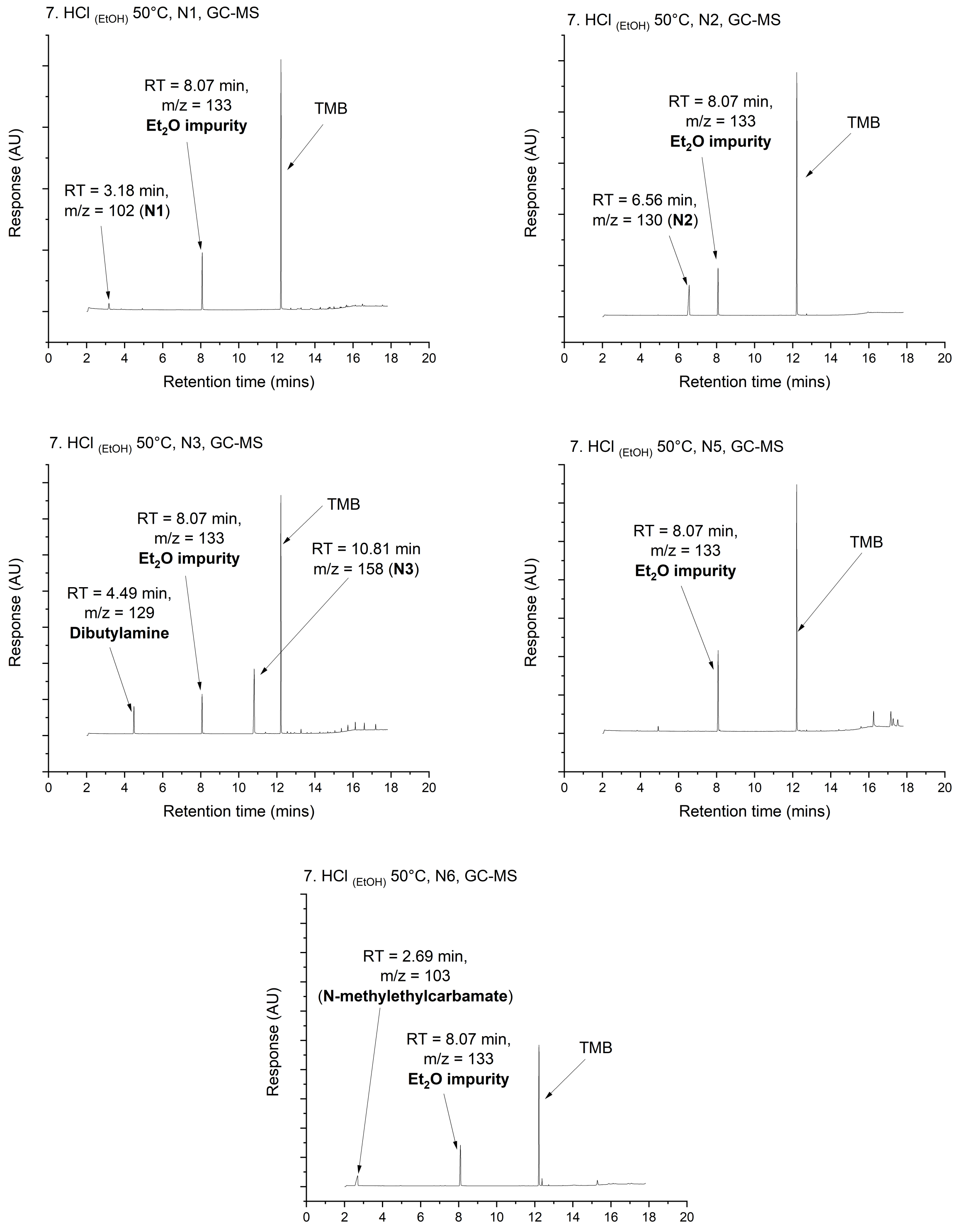

Figure S124: GC-MS/MS plots for N1, N2, N3, N5 and N6 for condition 7. As referenced in Section 3.3

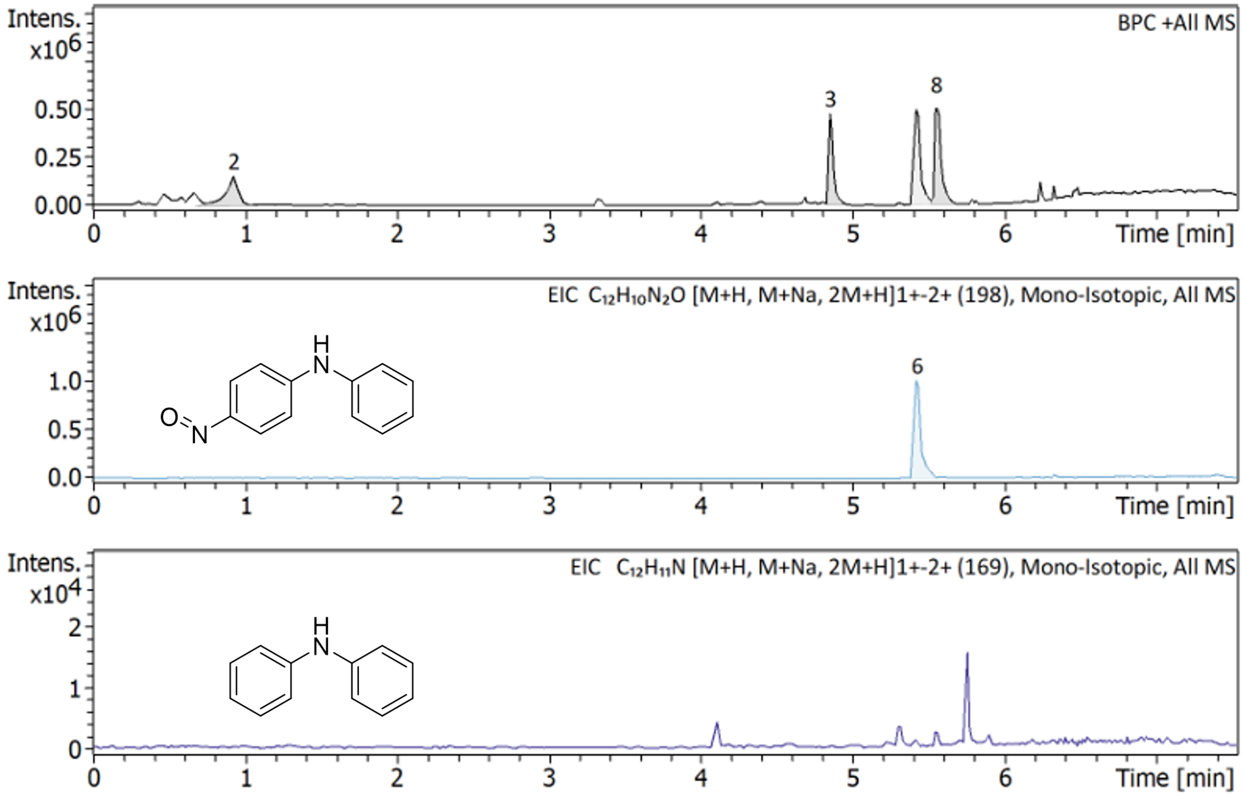

Summary of Results

Name	RT	BPC Area(%)	UV Area(%)	Confirm Formula Results
Cmpd 2, 0.9 min	0.93	18.4	no peak	
Cmpd 3, 4.9 min	4.85	21.4	18.0	
Cmpd 6, 5.4 min	5.42	29.2	14.0	C12H10N2O
Cmpd 8, 5.5 min	5.55	30.9	30.6	

Figure S125: LC-MS/MS experiment chromatogram for *N*-nitrosodiphenylamine (N4) in reaction condition 7. Referenced in Section 3.3.

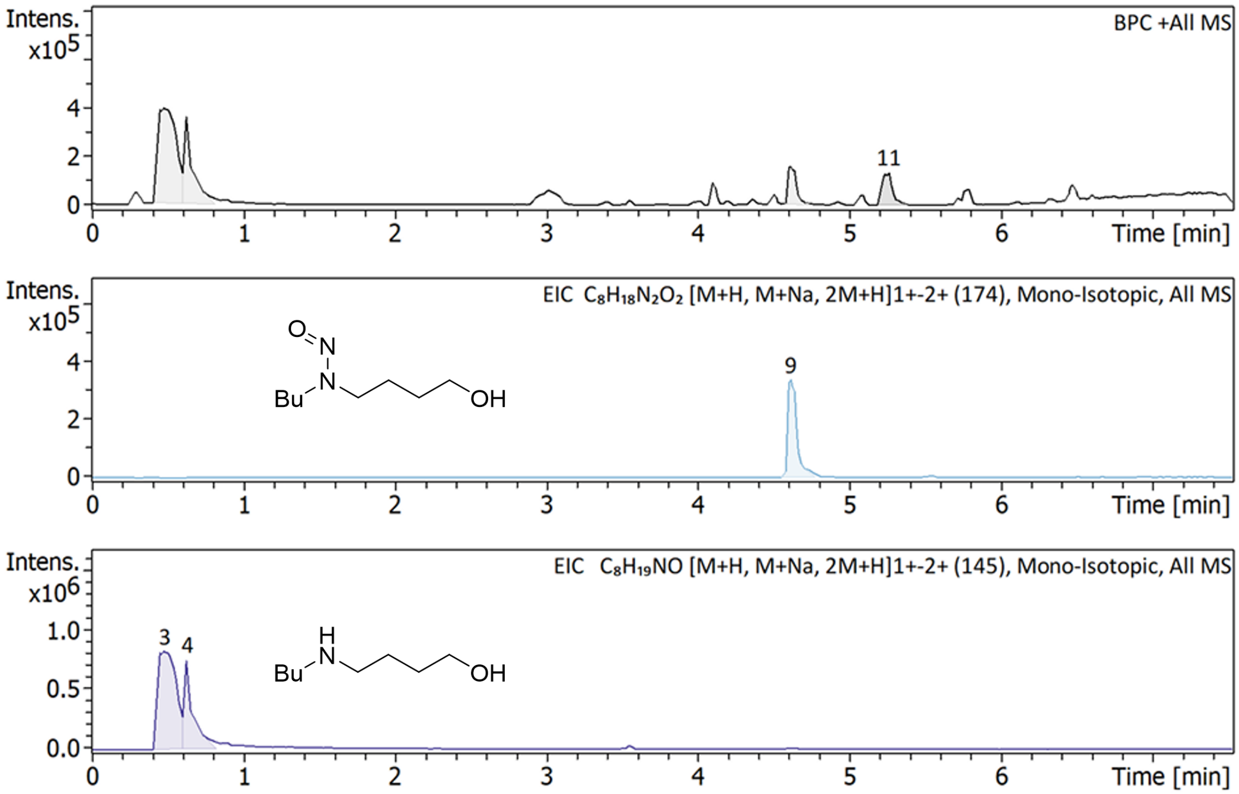

Summary of Results

Name	RT	BPC Area(%)	UV Area(%)	Confirm Formula Results
Cmpd 3, 0.5 min	0.48	59.0	30.6	C8H19NO
Cmpd 4, 0.6 min	0.63	22.3	no peak	C8H19NO
Cmpd 9, 4.6 min	4.61	9.1	35.9	C8H18N2O2
Cmpd 11, 5.3 min	5.26	9.6	no peak	

Figure S126: LC-MS/MS experiment chromatogram for *N*-butyl-*N*-(4-hydroxybutyl)nitrosamine (N7) in reaction condition 7. Referenced in Section 3.3.

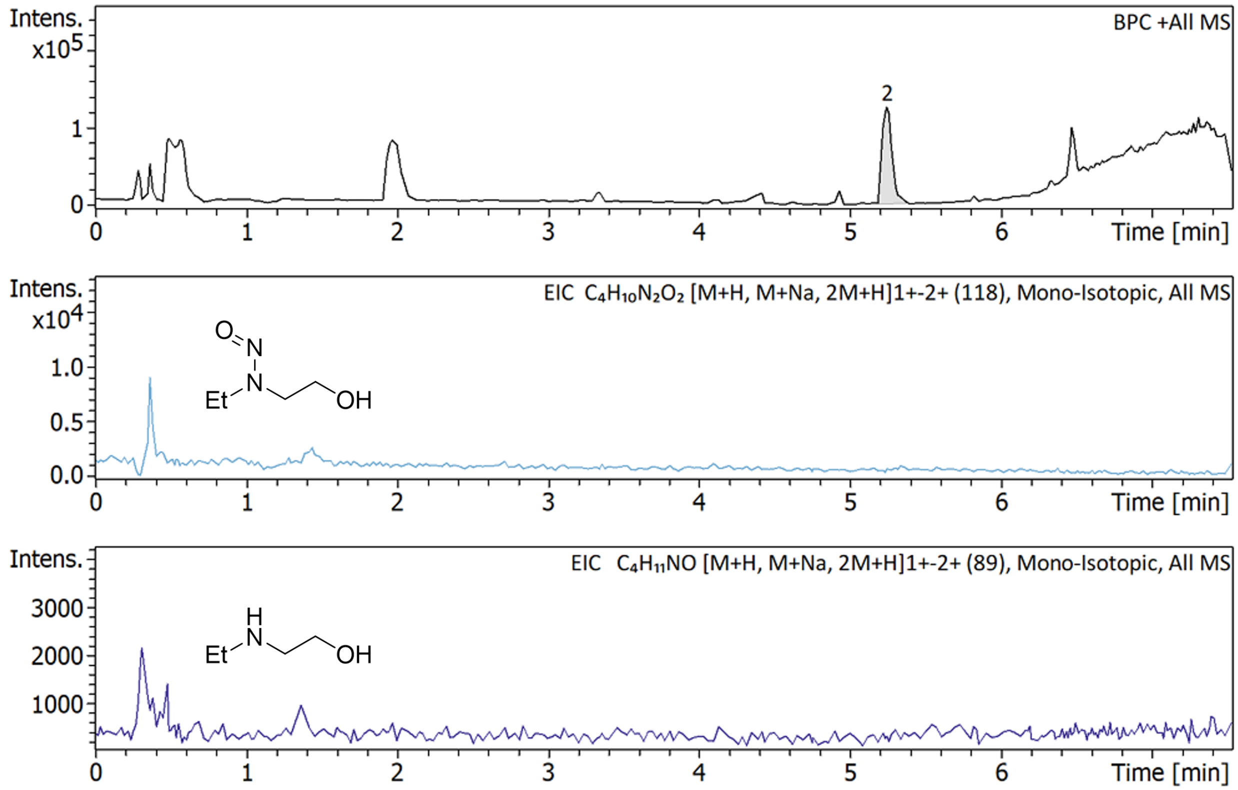

Summary of Results

Name	RT	BPC Area(%)	UV Area(%)	Confirm Formula Results
Cmpd 2, 5.2 min	5.24	no peak	no peak	

Figure S127: LC-MS/MS experiment chromatogram for *N*-ethyl-*N*-(2-hydroxyethyl)nitrosamine (N8) in reaction condition 7. Referenced in Section 3.3.

9.4 Hydride reductant conditions (8-10):

a)

Key:	0	50	100
conversion (%)			
No data			

No.	Class	Reagents	Solvent	Temp. (°C)	Reaction time (h)	N1	N2	N3	N4	N5	N6	N7	N8
8	Reductant	$NaBH_4$ 16 eq	EtOH	22	24	3	0	3	23	-2	101	5	0
9	Reductant	DiBAL-H 5 eq.	THF/Hexane	22	0.5	72	66	61	76	101	100	51	69
10	Reductant	DiBAL-H 5 eq.	THF/Hexane	22	1	85	86	75	85	101	100	90	101

b)

Key:	
Found	
Absent	
No Data	

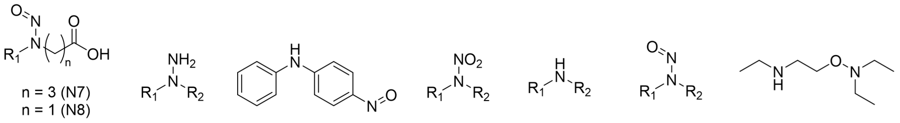

CA H P O A N D

	N1				N2				N3				N4					N5			N6		N7				N8				
No.	N	H	A	O	N	H	A	O	N	H	A	O	N	H	A	P	O	N	H	A	N	A	N	H	A	CA	N	H	A	CA	D
8	Found	Absent	Absent	Absent	Found	Absent	Absent	Absent	Found	Absent	Absent	Absent	Found	Absent	Found	Absent	Absent	Found	Absent	Absent	Absent	Absent	Found	Absent	Absent	Absent	Found	Absent	Absent	Absent	Absent
9	Found	Absent	Absent	Absent	Found	Found	Found	Absent	Found	Found	Found	Absent	Found	Found	Found	Absent	Absent	Absent	Found	Found	Absent	Absent	Found	Found	Found	Absent	Found	Found	Found	Absent	Found
10	Found	Absent	Absent	Absent	Found	Found	Found	Absent	Found	Found	Found	Absent	Found	Found	Found	Absent	Absent	Absent	Found	Found	Absent	Absent	Found	Found	Absent	Absent	Found	Found	Absent	Absent	Found

Figure S128: Reactivity of *N*-nitrosamines with hydride reductants in conditions 8-10. a) Conversions with respect to the nitrosamine and b) Products of the reactions determined by GC-MS/MS and LC-MS/MS.

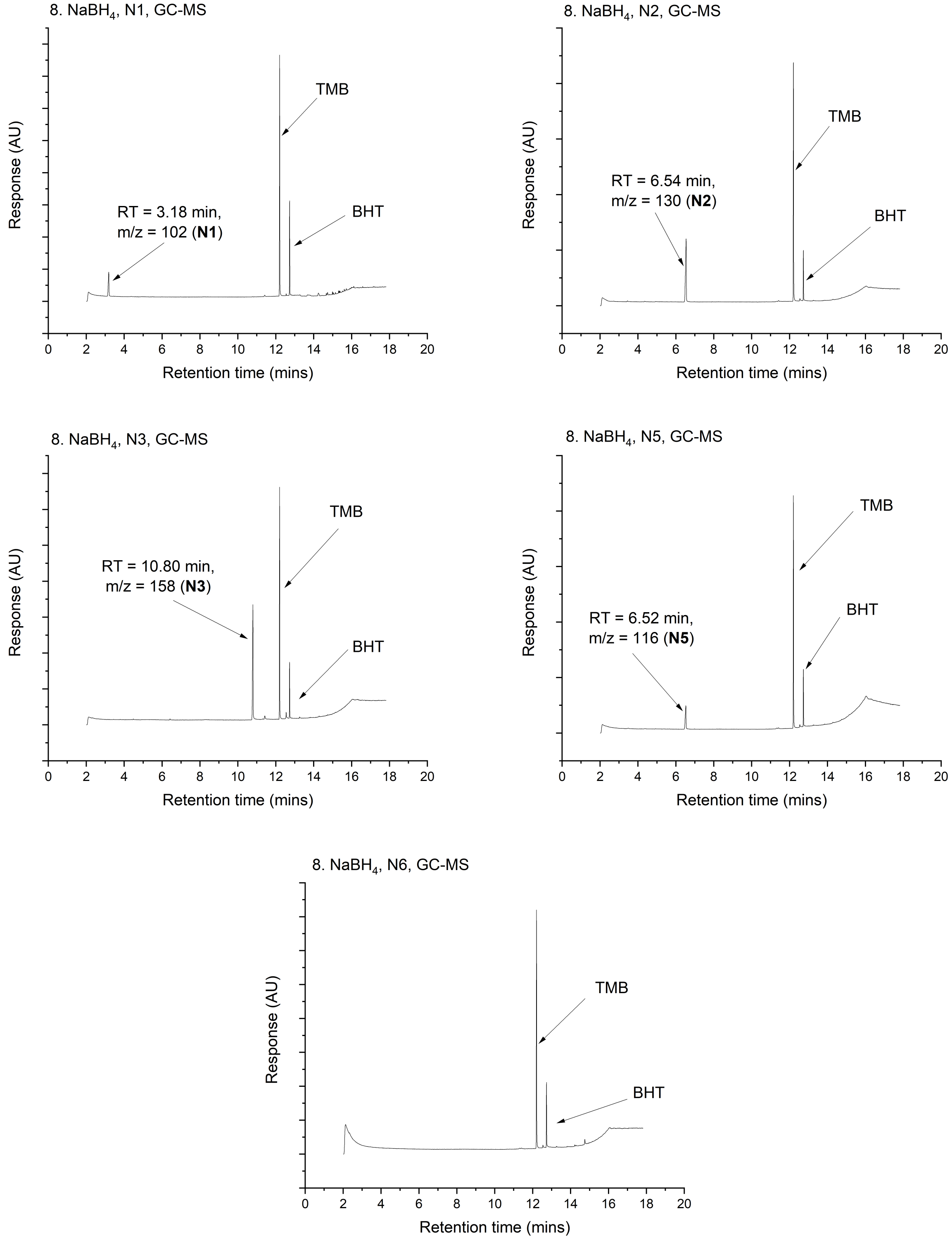

Figure S129: GC-MS/MS plots for N1, N2, N3, N5 and N6 for condition 8. As referenced in Section 3.3

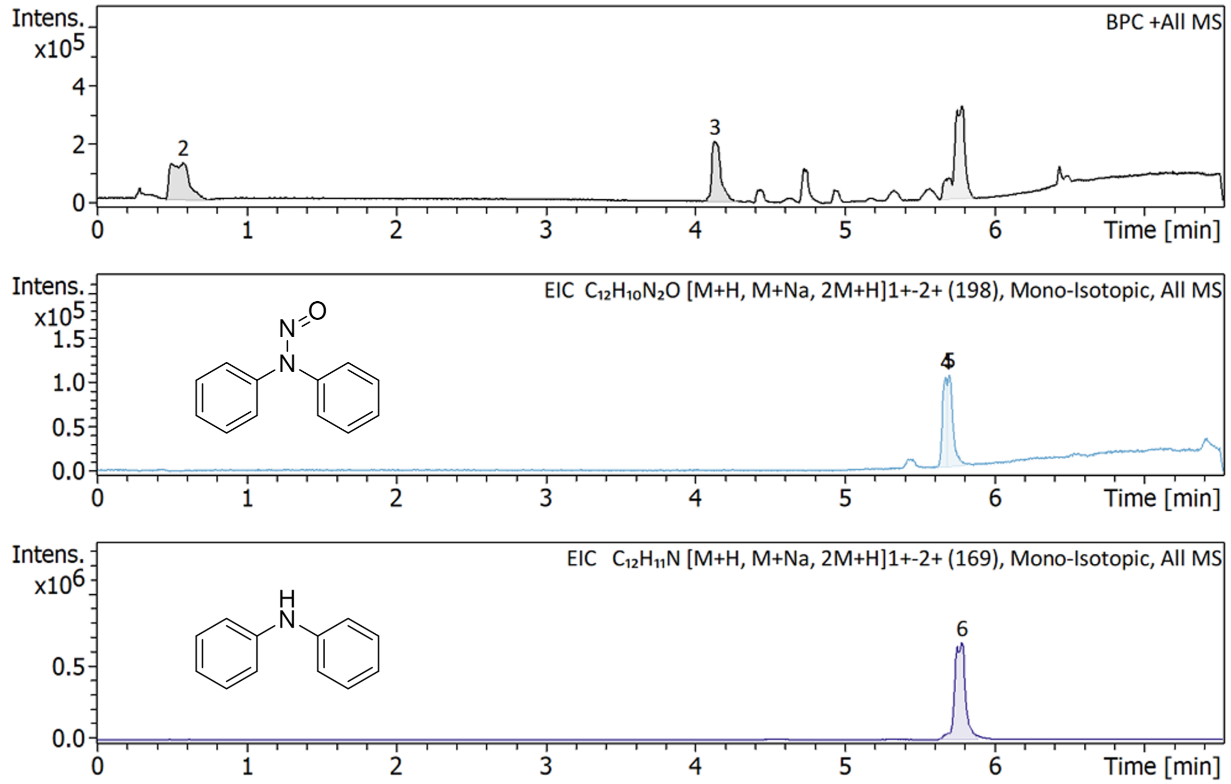

Summary of Results

Name	RT	BPC Area(%)	UV Area(%)	Confirm Formula Results
Cmpd 2, 0.6 min	0.58	30.7	no peak	
Cmpd 3, 4.1 min	4.13	24.5	no peak	
Cmpd 4, 5.7 min	5.67	no peak	no peak	C12H10N2O
Cmpd 5, 5.7 min	5.70	no peak	no peak	C12H10N2O
Cmpd 6, 5.8 min	5.78	44.8	no peak	C12H11N

Figure S130: LC-MS/MS experiment chromatogram for *N*-nitrosodiphenylamine (N4) in reaction condition 8. Referenced in Section 3.3.

.

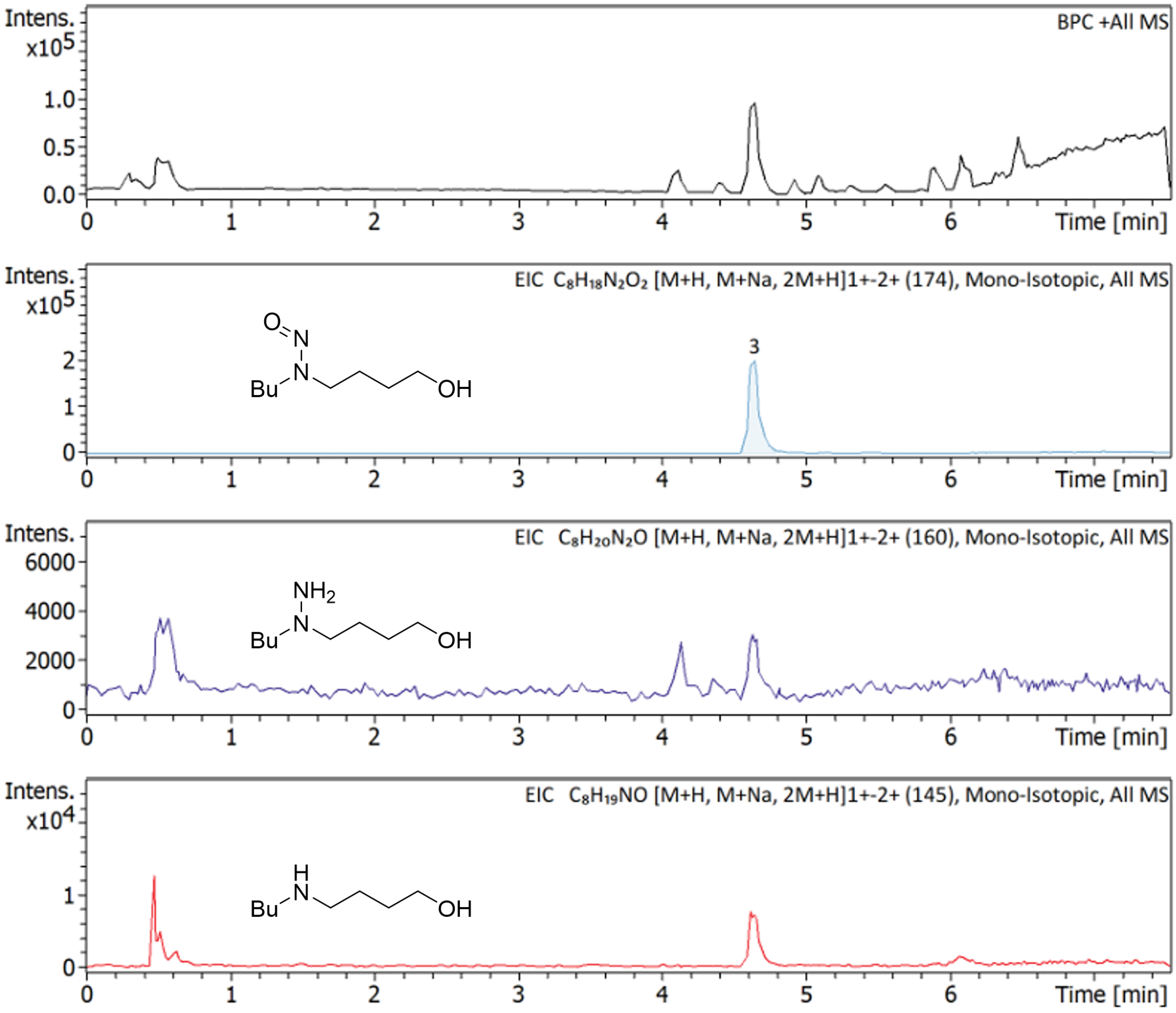

Summary of Results

Name	RT	BPC Area(%)	UV Area(%)	Confirm Formula Results
Cmpd 3, 4.6 min	4.64	no peak	88.0	C8H18N2O2

Figure S131: LC-MS/MS experiment chromatogram for *N*-butyl-*N*-(4-hydroxybutyl)nitrosamine (N7) in reaction condition 8. Referenced in Section 3.3.

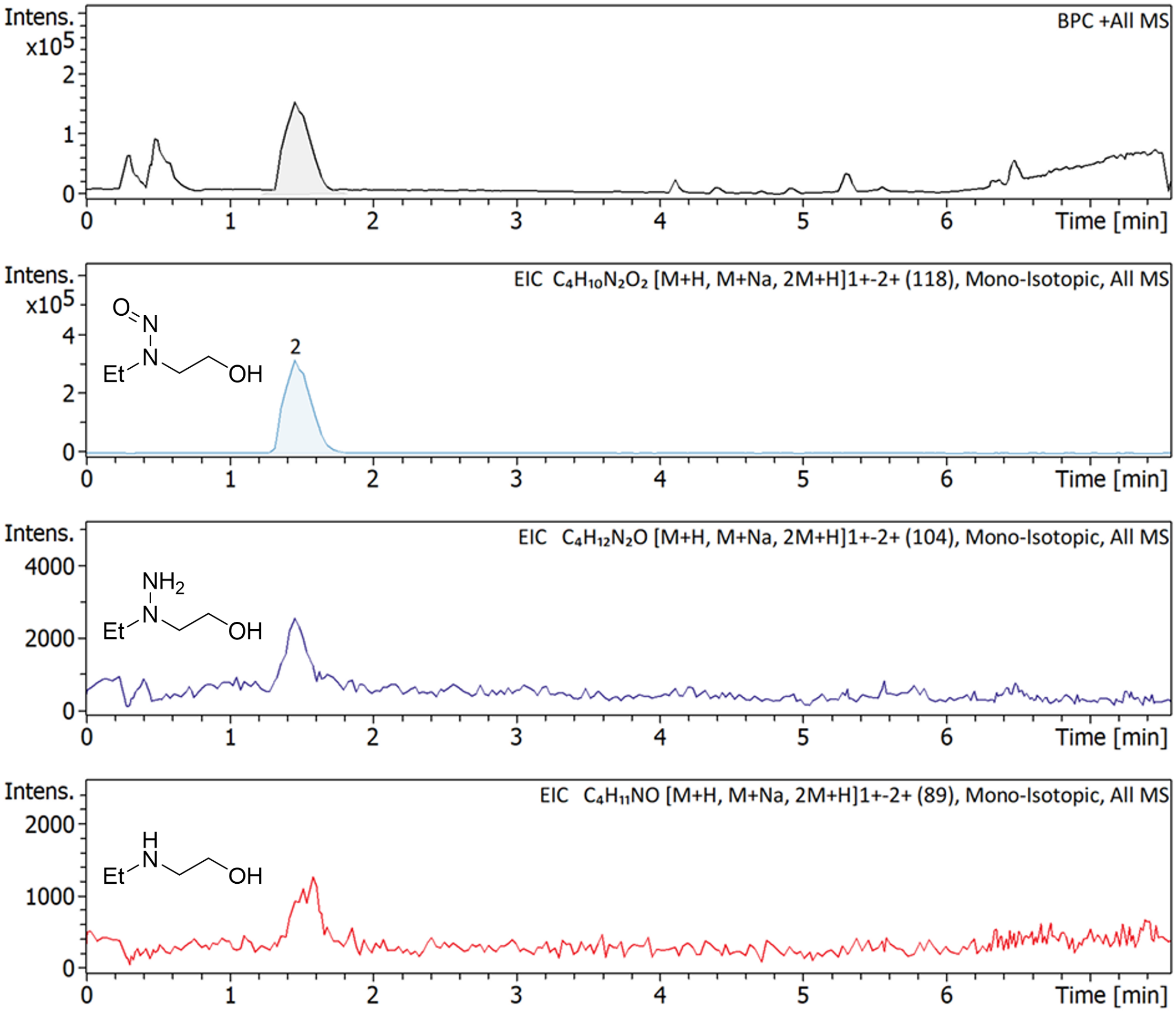

Summary of Results

Name	RT	BPC Area(%)	UV Area(%)	Confirm Formula Results
Cmpd 2, 1.5 min	1.46	100.0	63.3	C4H10N2O2,C4H10.5N2O2

Figure S132: LC-MS/MS experiment chromatogram for *N*-ethyl-*N*-(2-hydroxyethyl)nitrosamine (N8) in reaction condition 8. Referenced in Section 3.3.

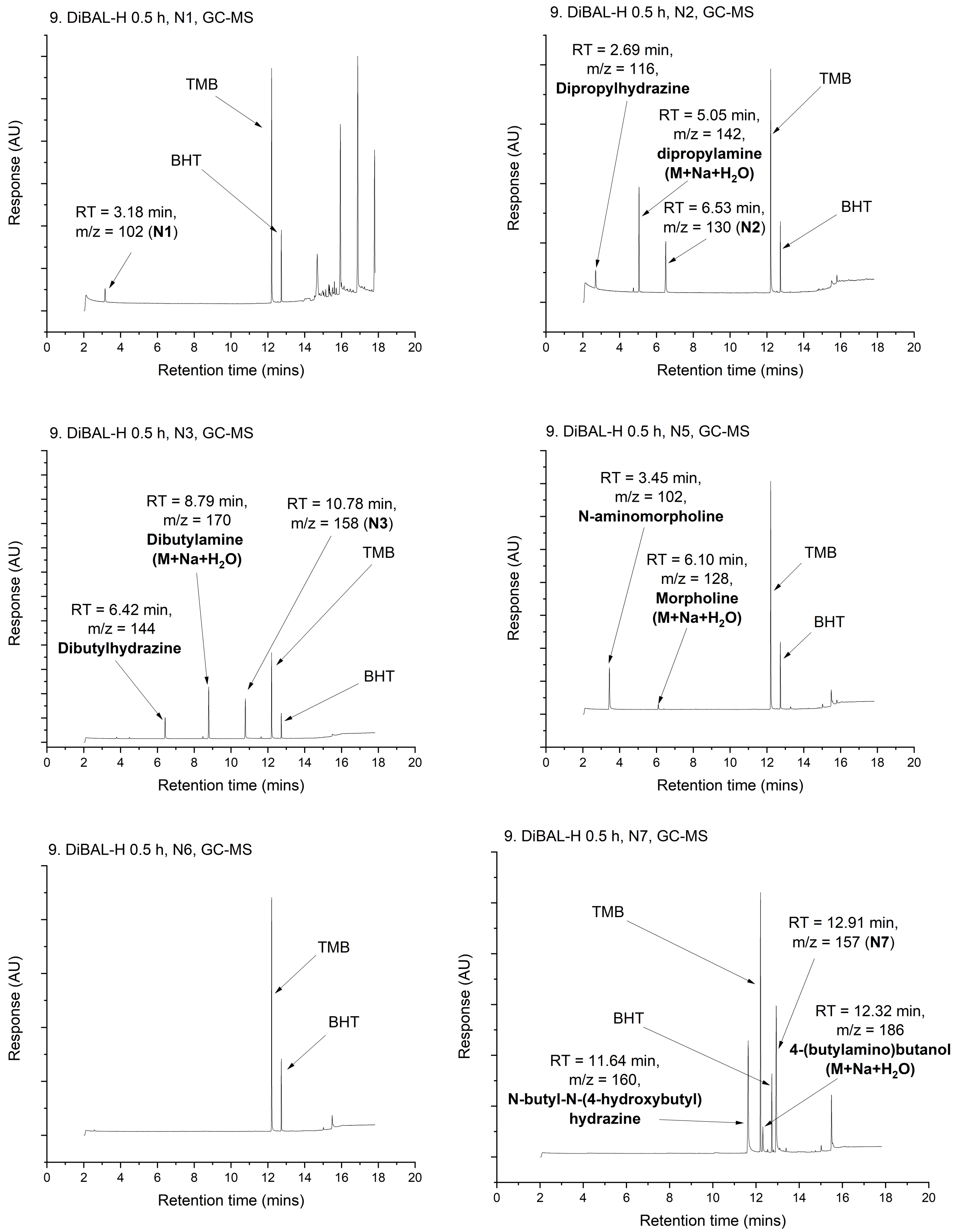

Figure S133: GC-MS/MS plots for N1, N2, N3 and N5-7 for condition 9. As referenced in Section 3.3

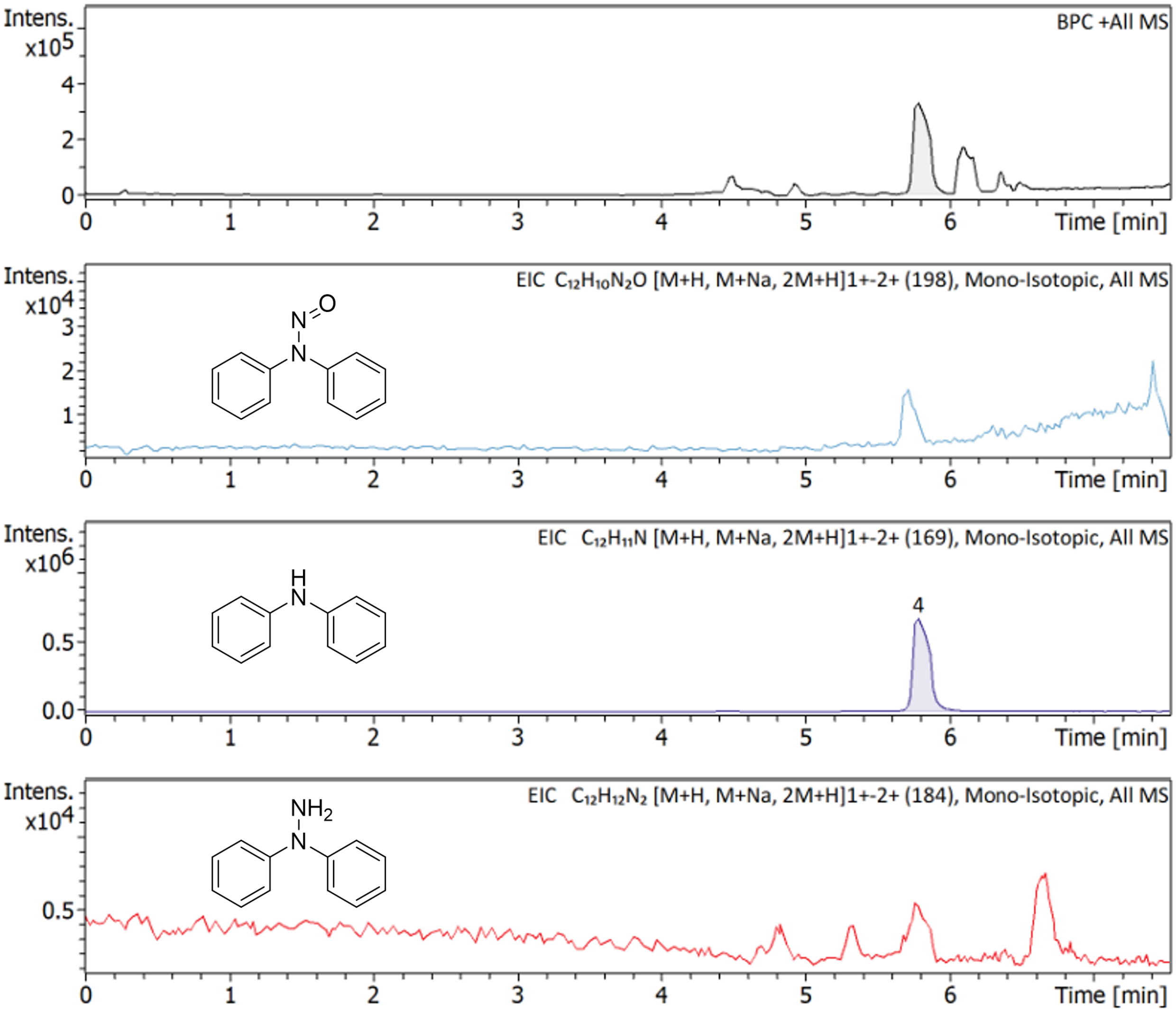

Summary of Results

Name	RT	BPC Area(%)	UV Area(%)	Confirm Formula Results
Cmpd 4, 5.8 min	5.78	100.0	28.5	C12H11N

Figure S134: LC-MS/MS experiment chromatogram for *N*-nitrosodiphenylamine (N4) in reaction condition 9. Referenced in Section 3.3.

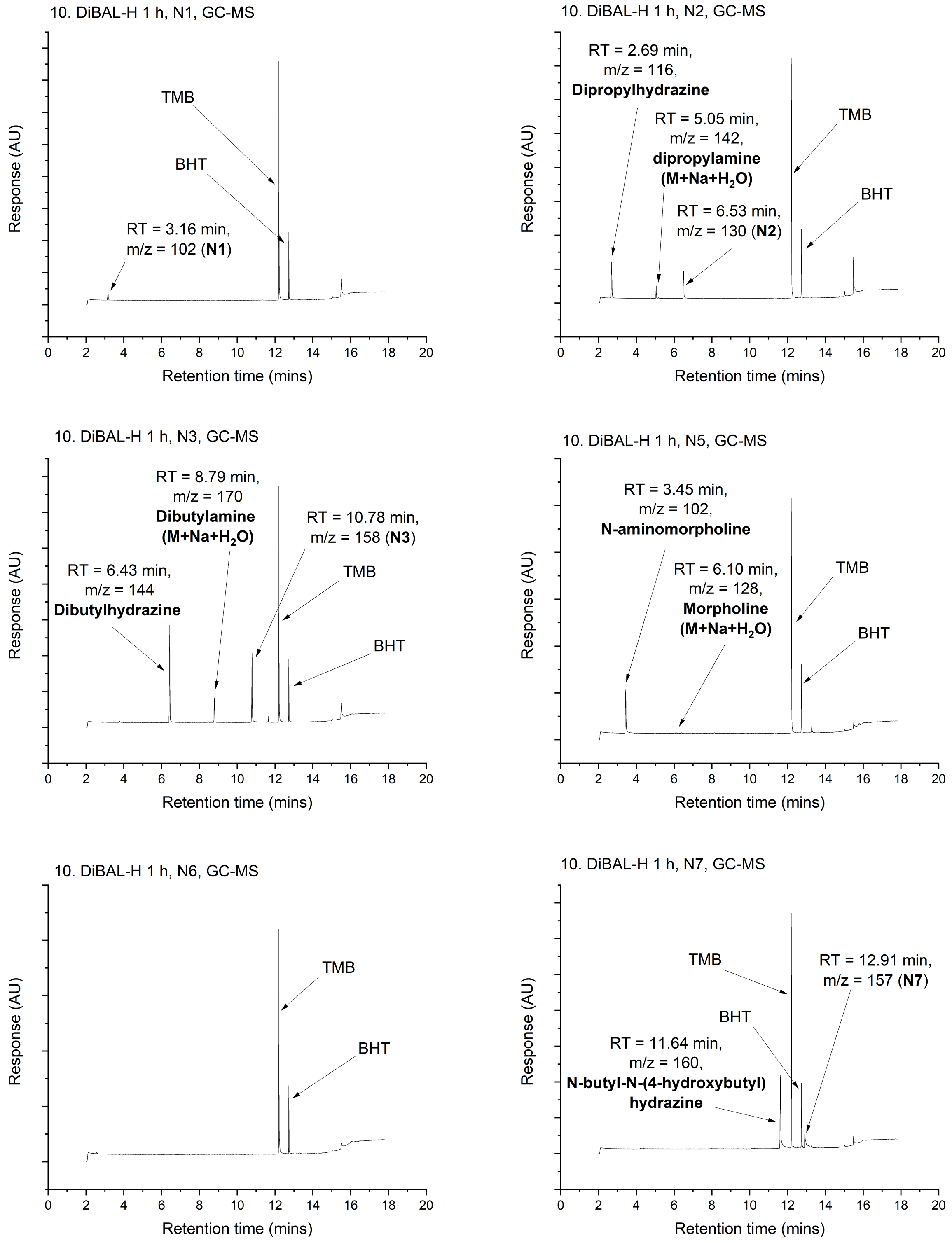

Figure S135: GC-MS/MS plots for N1, N2, N3 and N5-7 for condition 10. As referenced in Section 3.3

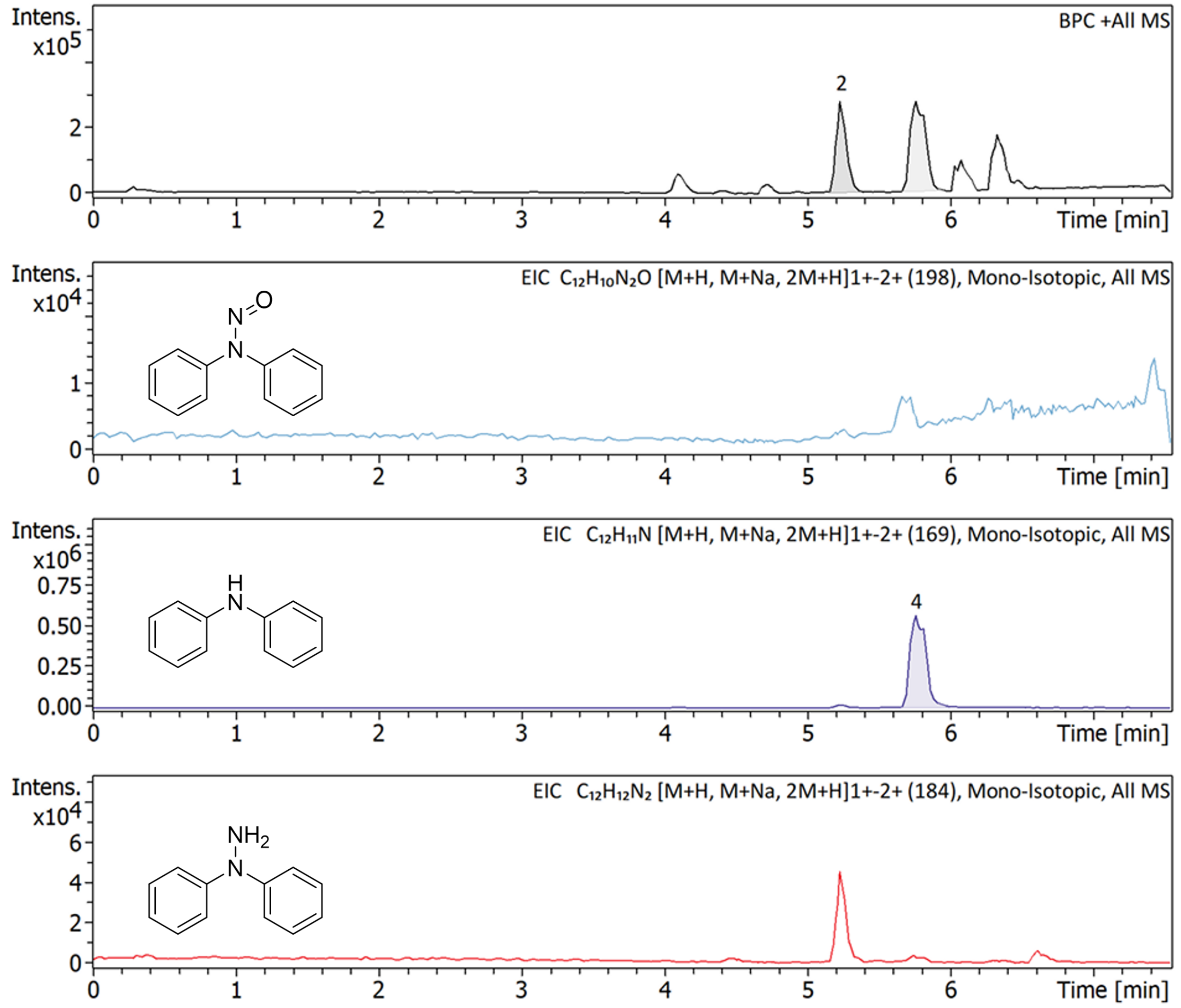

Summary of Results

Name	RT	BPC Area(%)	UV Area(%)	Confirm Formula Results
Cmpd 2, 5.2 min	5.23	39.9	no peak	C12H12N2
Cmpd 4, 5.8 min	5.76	60.1	32.6	C12H11N

Figure S136: LC-MS/MS experiment chromatogram for N-nitrosodiphenylamine (N4) in reaction condition 10. Referenced in Section 3.3.

9.5 Analysis of the products of N8 in conditions 9 and 10

The product determination analysis of the reaction between N8 and DiBAL-H in conditions 9 and 10 revealed an unexpected product, produced alongside the amine and hydrazine

products as shown in Figures S138 and S139 at m/z= 160.1. Further analysis of the reaction mixture by LC-MS confirmed the presence of the peak, which was not representative of the starting material, amine, or hydrazine. Based on the m/z of 160.1 and the fragment ion peaks we assign this peak to the dimer of the amine product of N8 (structure D in the product analysis summary tables) (Figure S137).

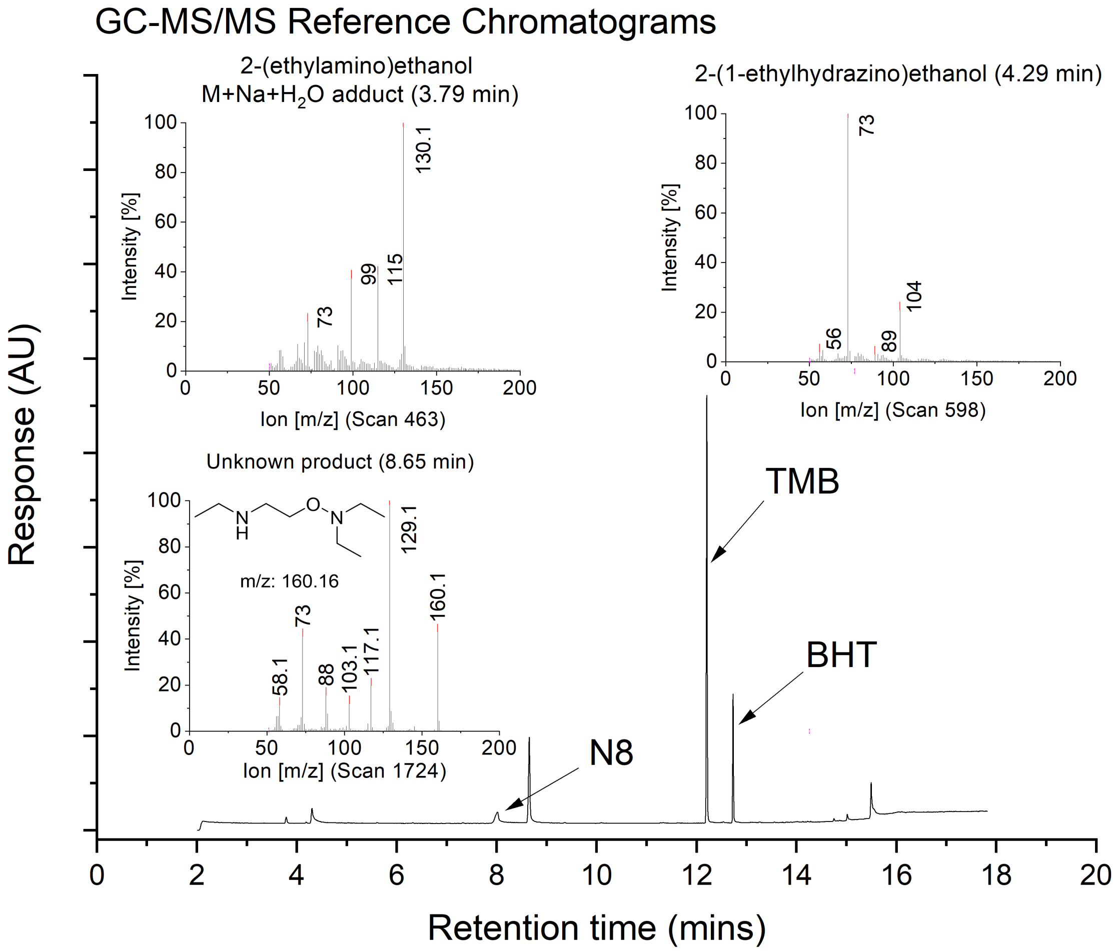

Figure S137: GC-MS/MS reference for 2-(ethylamino)ethanol and 2-(1-ethylhydrazino) ethanol, and unknown product from reaction of N8 with DiBAL-H from condition 9. Referenced in Table S7

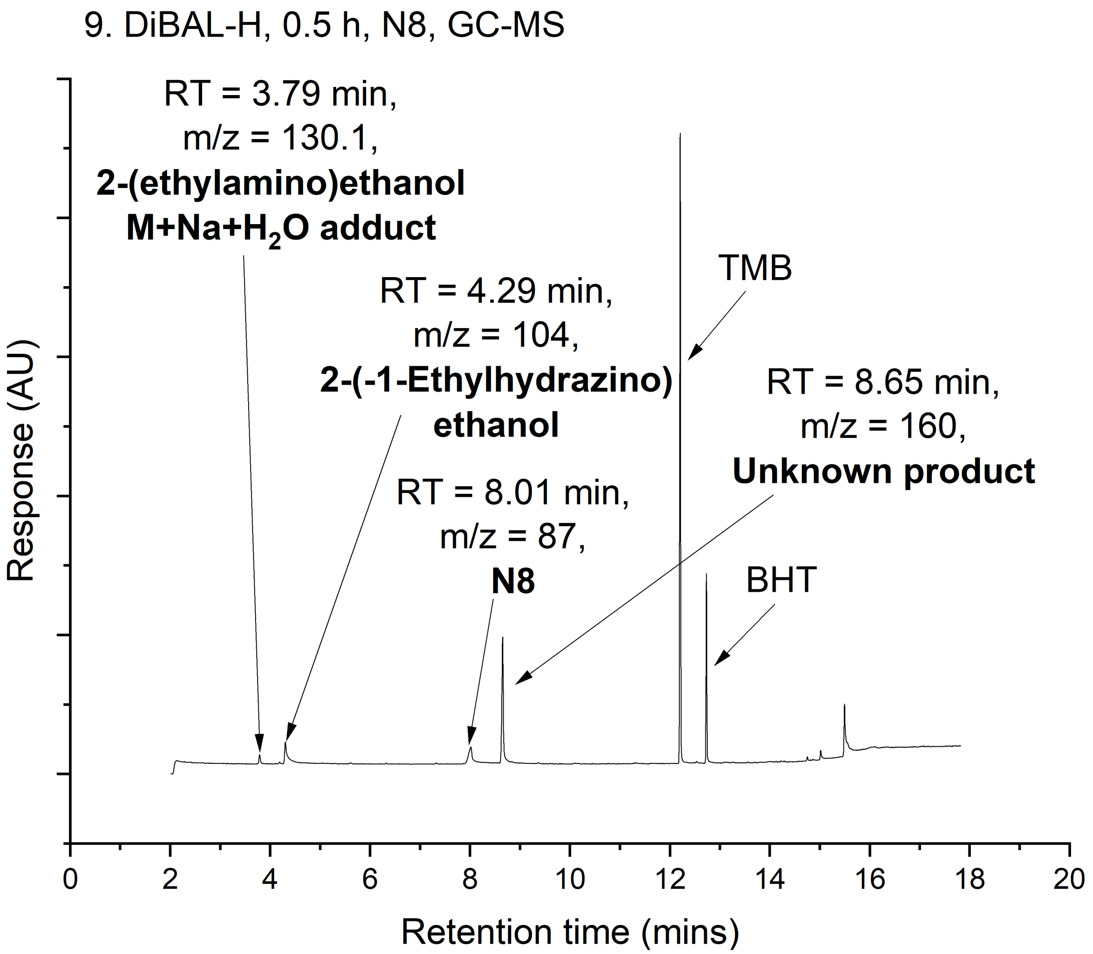

Figure S138: GC-MS/MS experiment chromatogram for N-ethyl-N-(2-hydroxyethyl)nitrosamine (N8) in reaction condition 9. Referenced in Section 3.3.

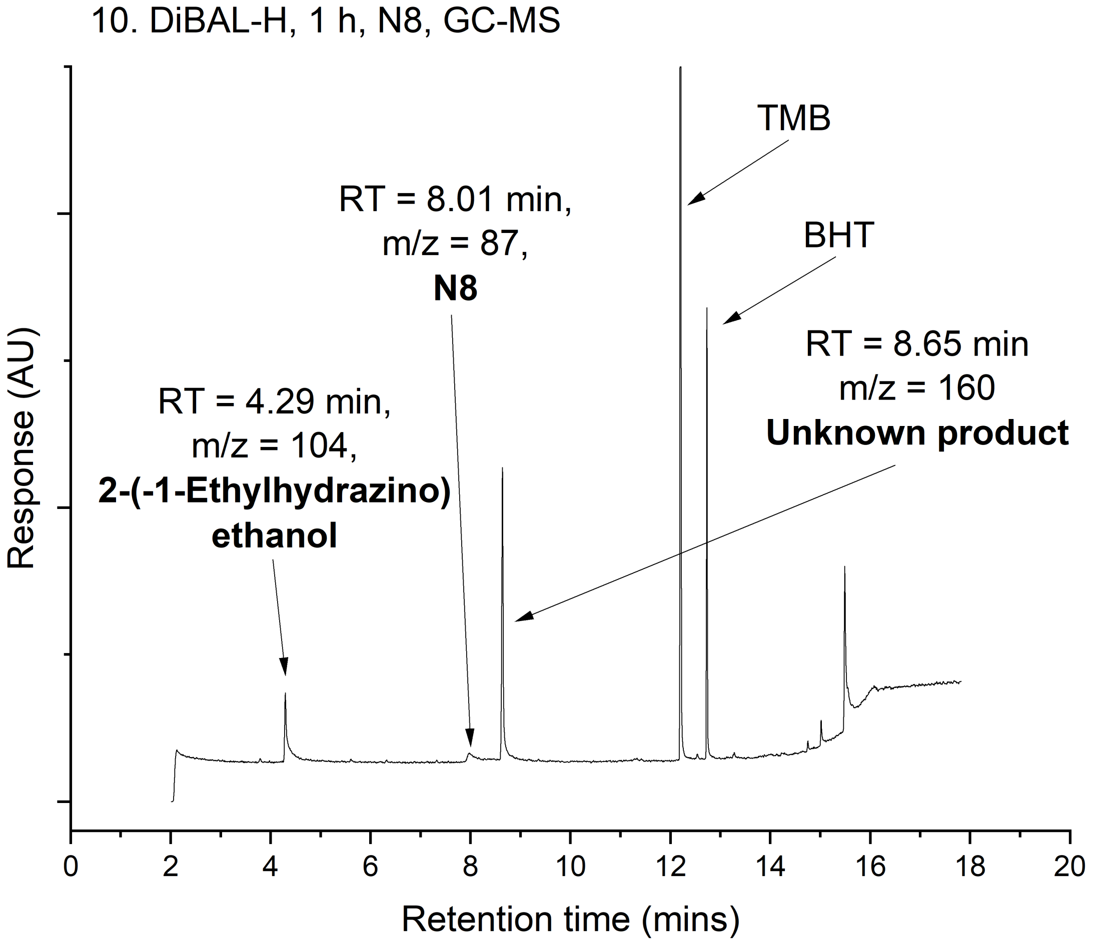

Figure S139: GC-MS/MS experiment chromatogram for *N*-ethyl-*N*-(2-hydroxyethyl)nitrosamine (N8) in reaction condition 10. Referenced in Section 3.3.

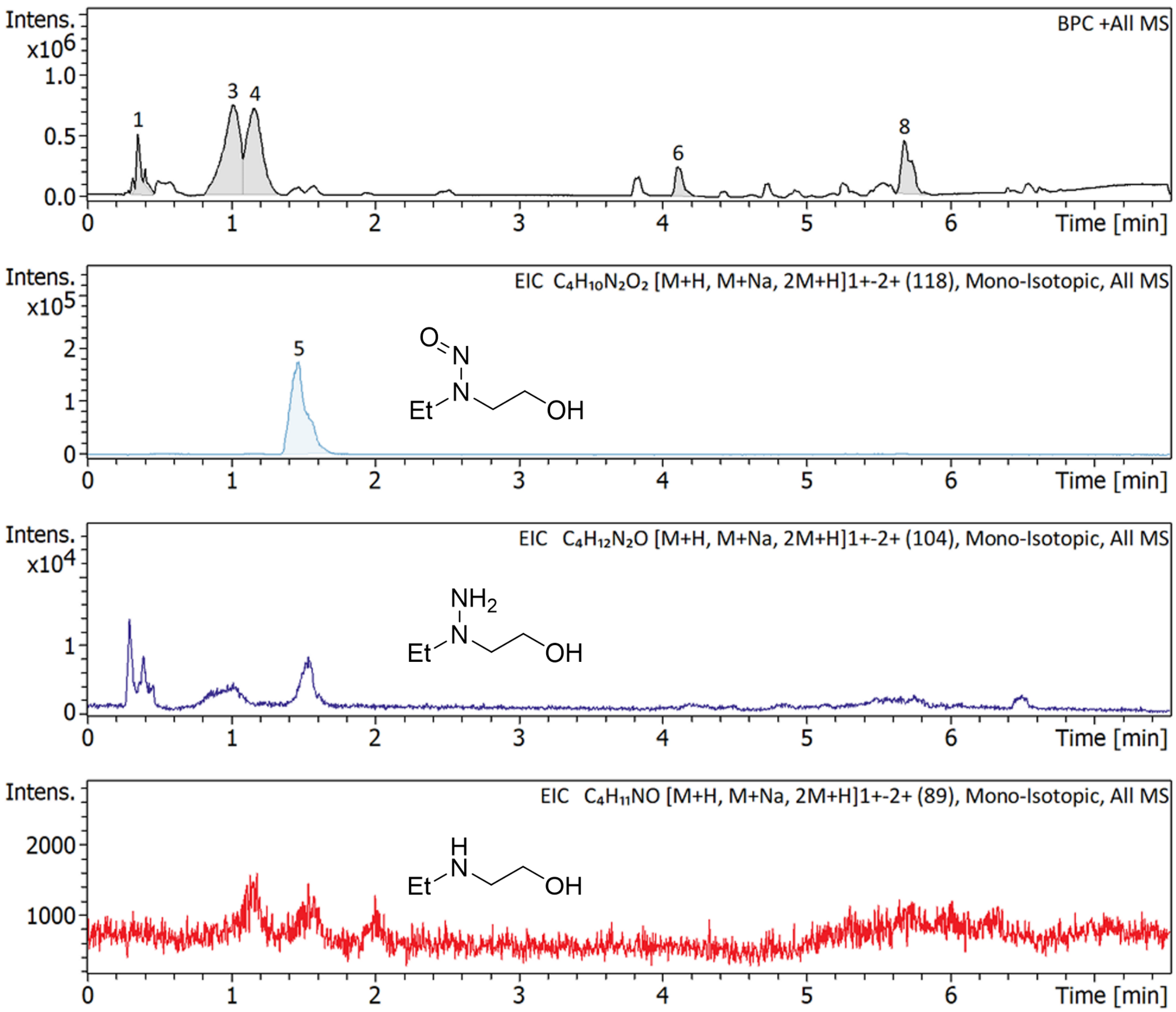

Summary of Results

Name	RT	BPC Area(%)	UV Area(%)	Confirm Formula Results
Cmpd 1, 0.4 min	0.36	7.9	no peak	
Cmpd 3, 1.0 min	1.01	37.9	no peak	
Cmpd 4, 1.2 min	1.17	34.3	no peak	
Cmpd 5, 1.5 min	1.47	no peak	no peak	C4H10N2O2,C4H10.5N2O2
Cmpd 6, 4.1 min	4.11	6.0	no peak	
Cmpd 8, 5.7 min	5.68	13.9	48.8	

Figure S140: LC-MS/MS experiment chromatogram for ethyl-*N*-(2-hydroxyethyl)nitrosamine (N8) in reaction condition 9. Referenced in Section 3.3.

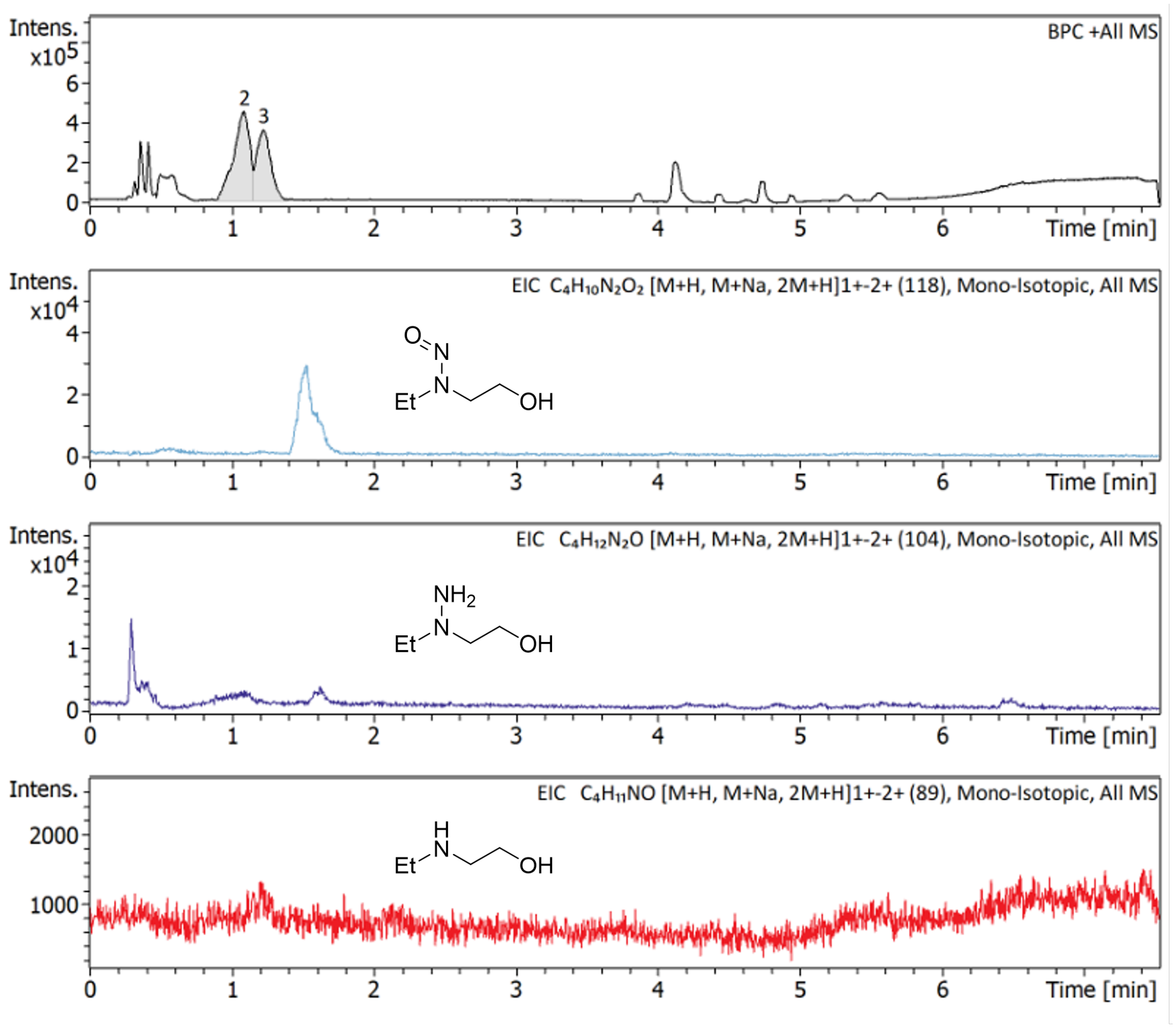

Summary of Results

Name	RT	BPC Area(%)	UV Area(%)	Confirm Formula Results
Cmpd 2, 1.1 min	1.09	58.3	no peak	
Cmpd 3, 1.2 min	1.22	41.7	no peak	

Figure S141: LC-MS/MS experiment chromatogram for ethyl-N-(2-hydroxyethyl)nitrosamine (N8) in reaction condition 10. Referenced in Section 3.3.

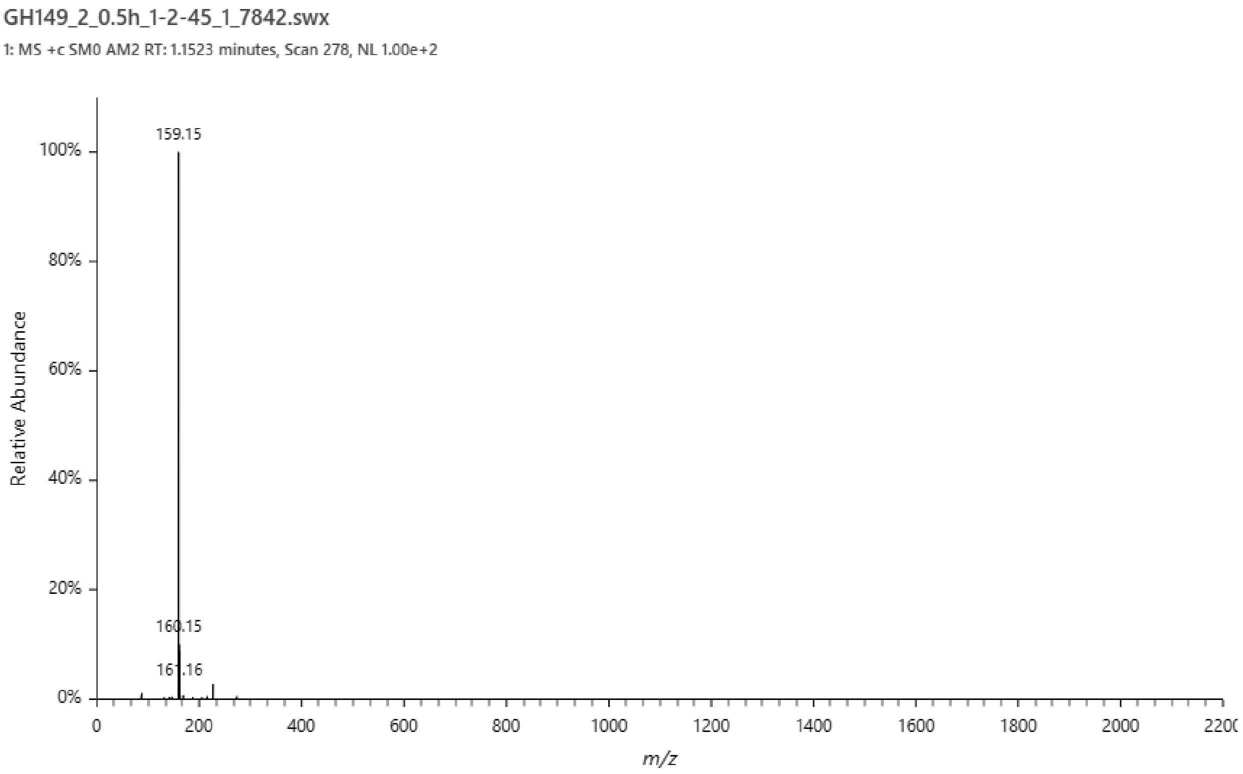

Figure S142: LC-MS/MS reference mass peaks for the products of the N8 in condition 9 (Compounds 3+4). As referenced in Table S9.

9.6 Sulfur-based reductants conditions (11-19):

a)

Key:	0	50	100
conversion (%)			
No data			

No.	Class	Reagents	Solvent	Temp. (°C)	Reaction time (h)	N1	N2	N3	N4	N5	N6	N7	N8
11	Reductant	$Na_2S_2O_4$	H_2O/EtOH	22	24	9	3	9	87	25	63	-5	5
12	Reductant	$Na_2S_2O_4$ + NaOH 0.1M	H_2O/EtOH	22	24	-4	2	9	87	32	100	-9	2
13	Reductant	$Na_2S_2O_4$ + NaOH 0.1M	H_2O/EtOH	50	16	18	7	11	99	67	101	6	40
14	Reductant	$Na_2S_2O_4$ + NaOH 1M	H_2O/EtOH	50	16	78	57	56	99	99	101	87	99
15	Reductant	$Na_2S_2O_4$ + NaOH 1M	H_2O/EtOH	50	3	18	1	11	99	75	101	14	32
16	Reductant	$Na_2S_2O_4$ + NaOH 1M	H_2O/EtOH	50	1	18	2	12	97	44	101	-4	17
17	Reductant	$Na_2S_2O_4$ + 20% NaOH	H_2O/EtOH	50	16	106	104	104	95	96	100	107	101
18	Reductant	Na_2SO_3	H_2O/EtOH	22	24	4	-2	6	22	-2	102	-6	3
19	Reductant	Na_2SO_3 + 1M NaOH	H_2O/EtOH	50	16	2	1	7	75	1	101	-3	-1

b)

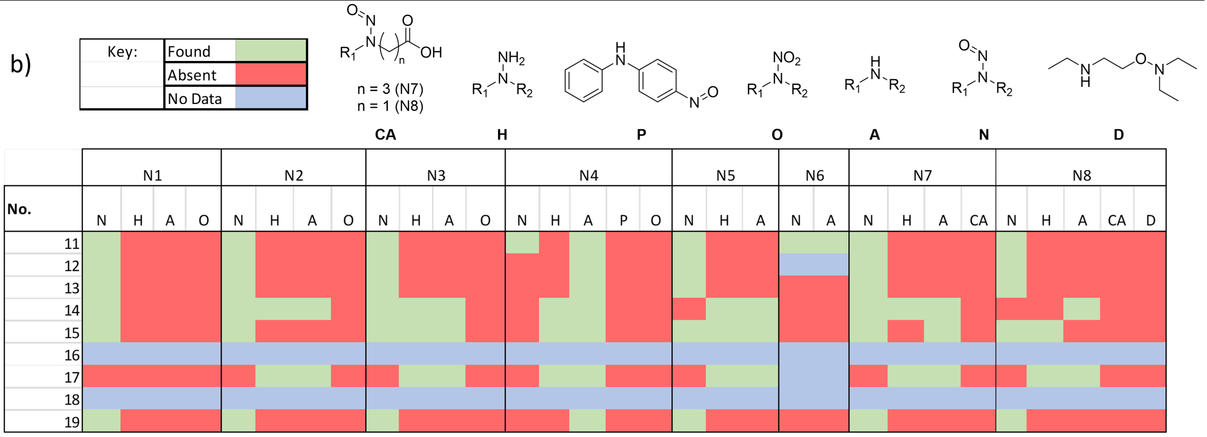

Figure S143: Reactivity of N-nitrosamines with sulfur-based reductants in conditions 11-19. a) Conversions with respect to the nitrosamine and b) Products of the reactions determined by GC-MS/MS and LC-MS/MS.

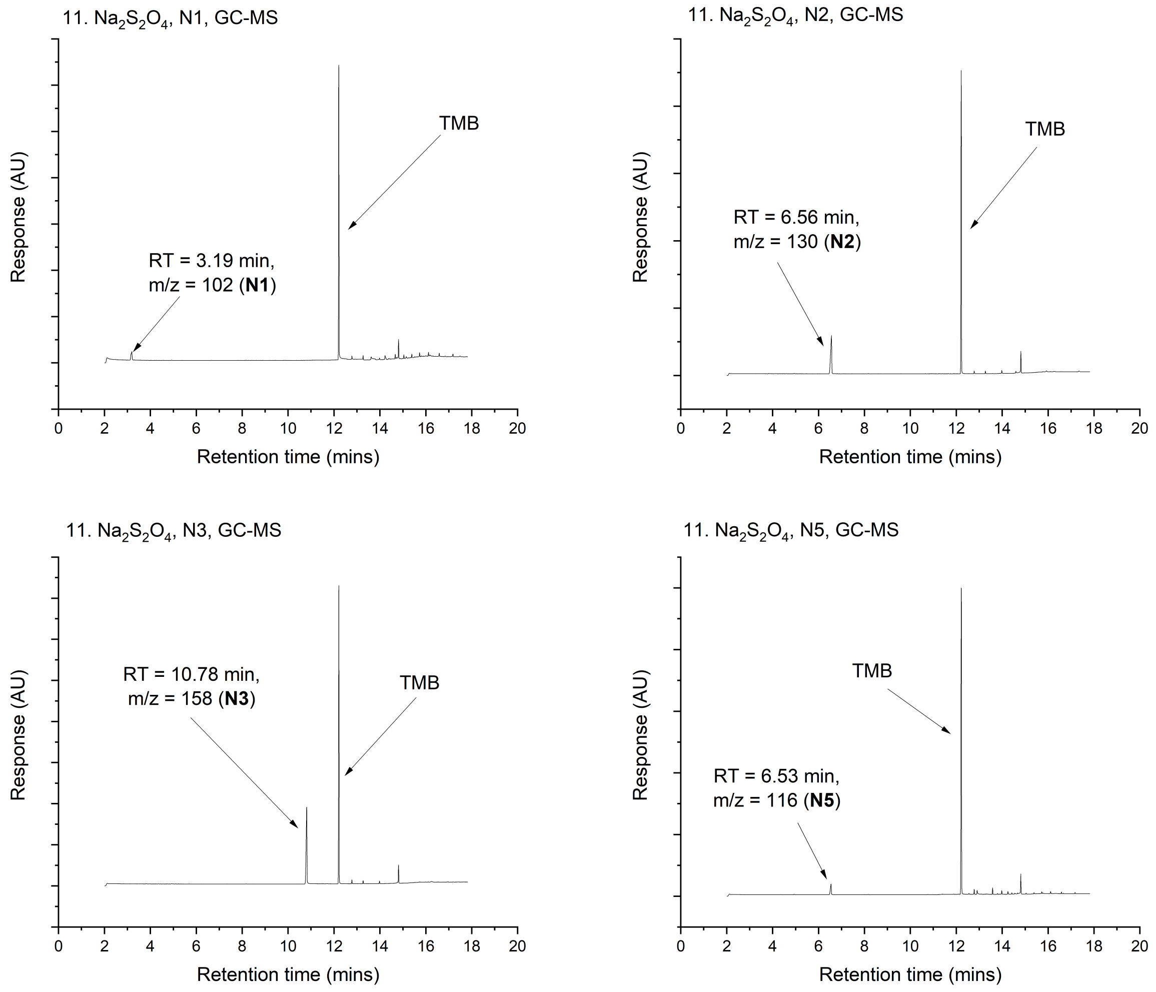

Figure S144: GC-MS/MS plots for N1, N2, N3 and N5 condition 11. As referenced in Section 3.3

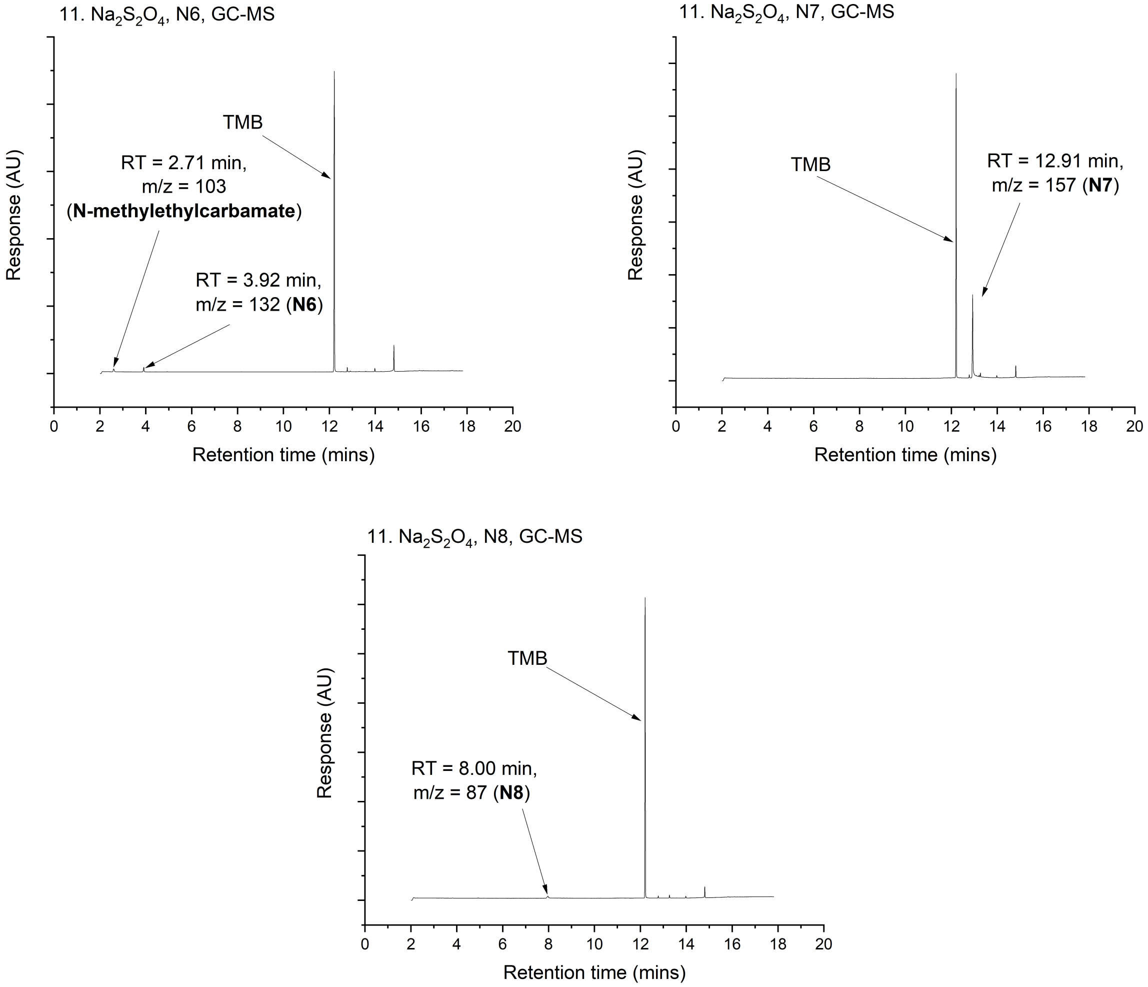

Figure S145: GC-MS/MS plots for N6-8 for condition 11. As referenced in Section 3.3

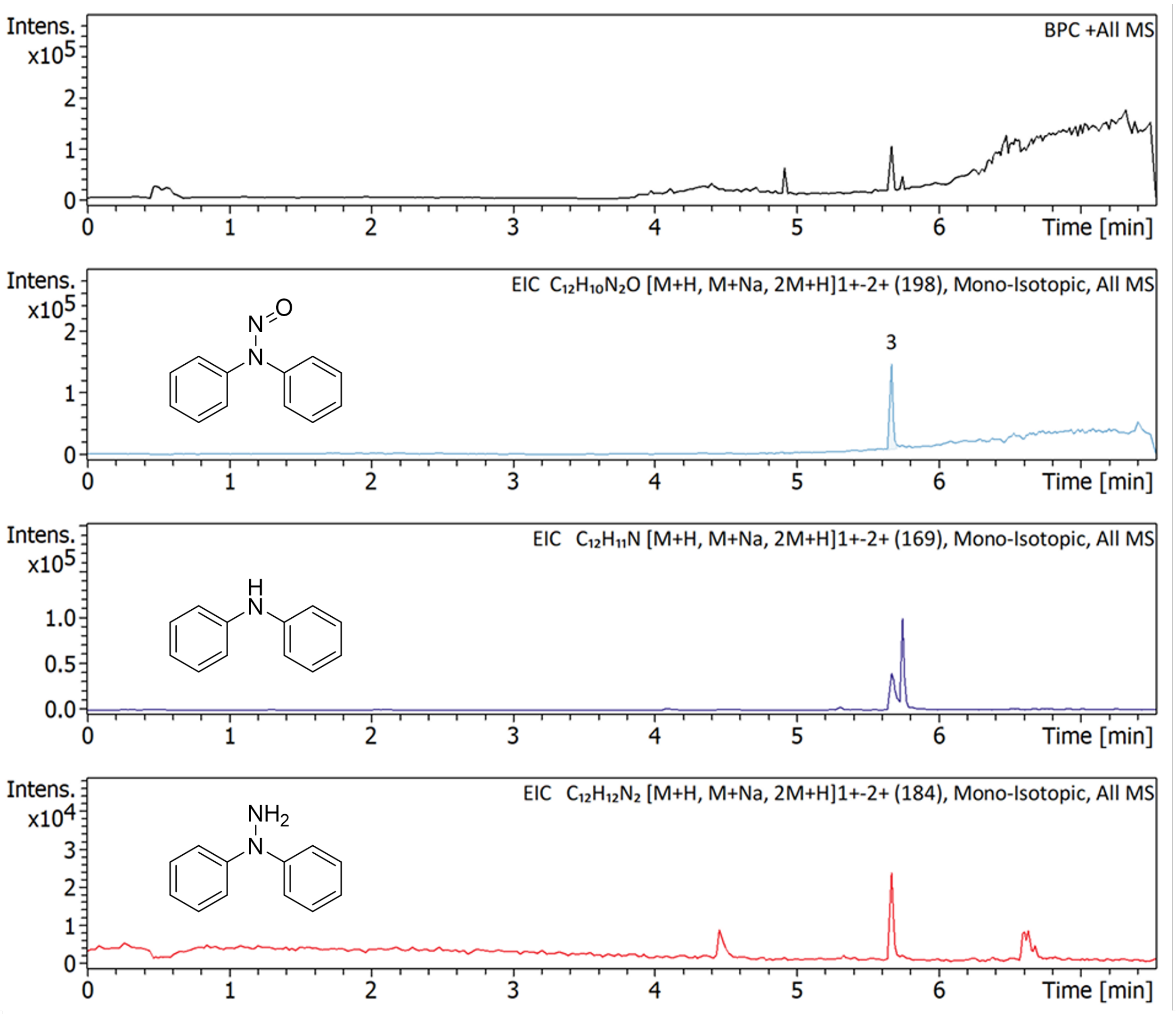

Summary of Results

Name	RT	BPC Area(%)	UV Area(%)	Confirm Formula Results
Cmpd 3, 5.7 min	5.66	no peak	62.4	C12H10N2O

Figure S146: LC-MS/MS experiment chromatogram for *N*-nitrosodiphenylamine (N4) in reaction condition 11. Referenced in Section 3.3.

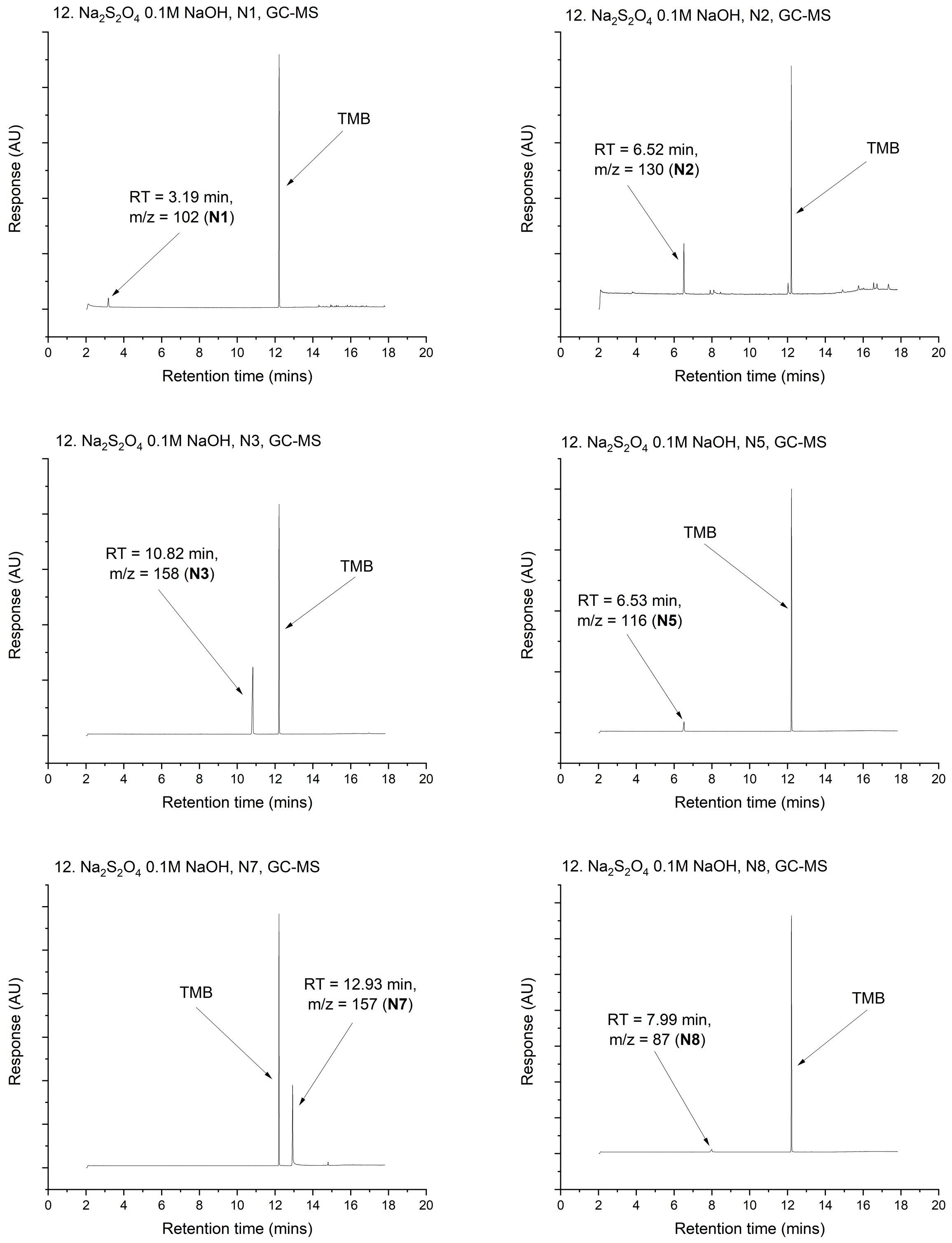

Figure S147: GC-MS/MS plots for N1-3, N5, N7 and N8 for condition 12. As referenced in Section 3.3

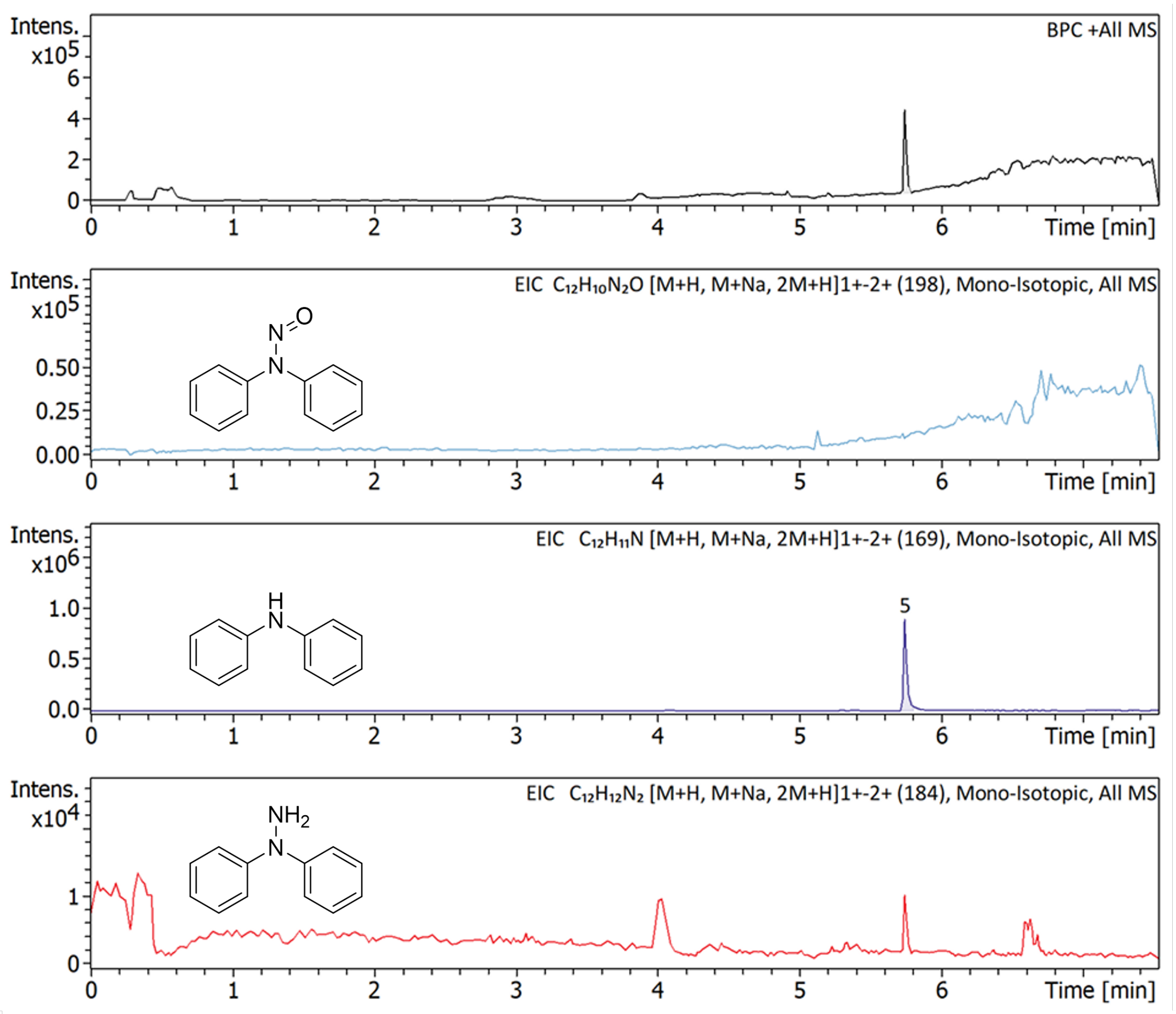

Summary of Results

Name	RT	BPC Area(%)	UV Area(%)	Confirm Formula Results
Cmpd 5, 5.7 min	5.74	100.0	70.0	C12H11N

Figure S148: LC-MS/MS experiment chromatogram for *N*-nitrosodiphenylamine (N4) in reaction condition 12. Referenced in Section 3.3.

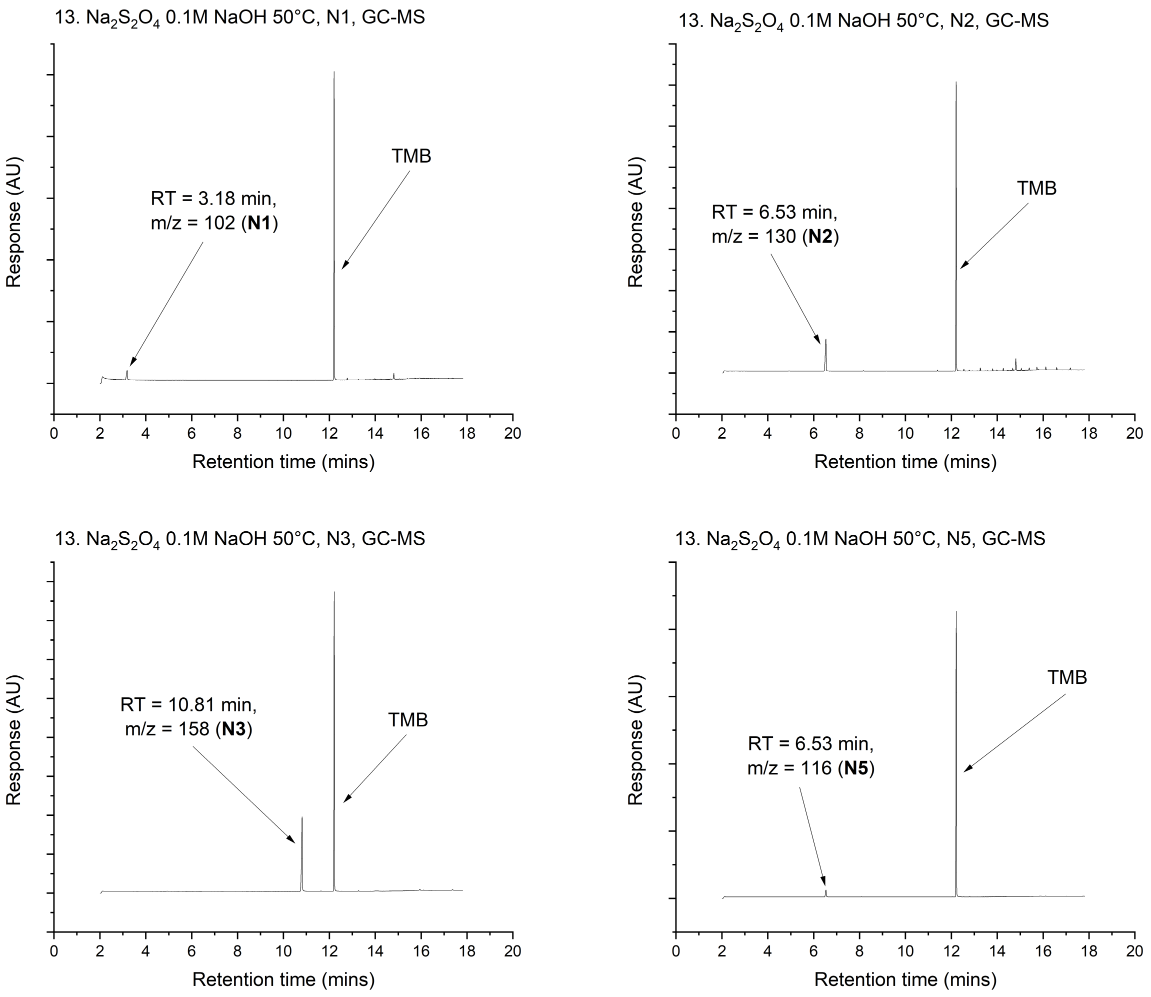

Figure S149: GC-MS/MS plots for N1, N2, N3 and N5 condition 13. As referenced in Section 3.3

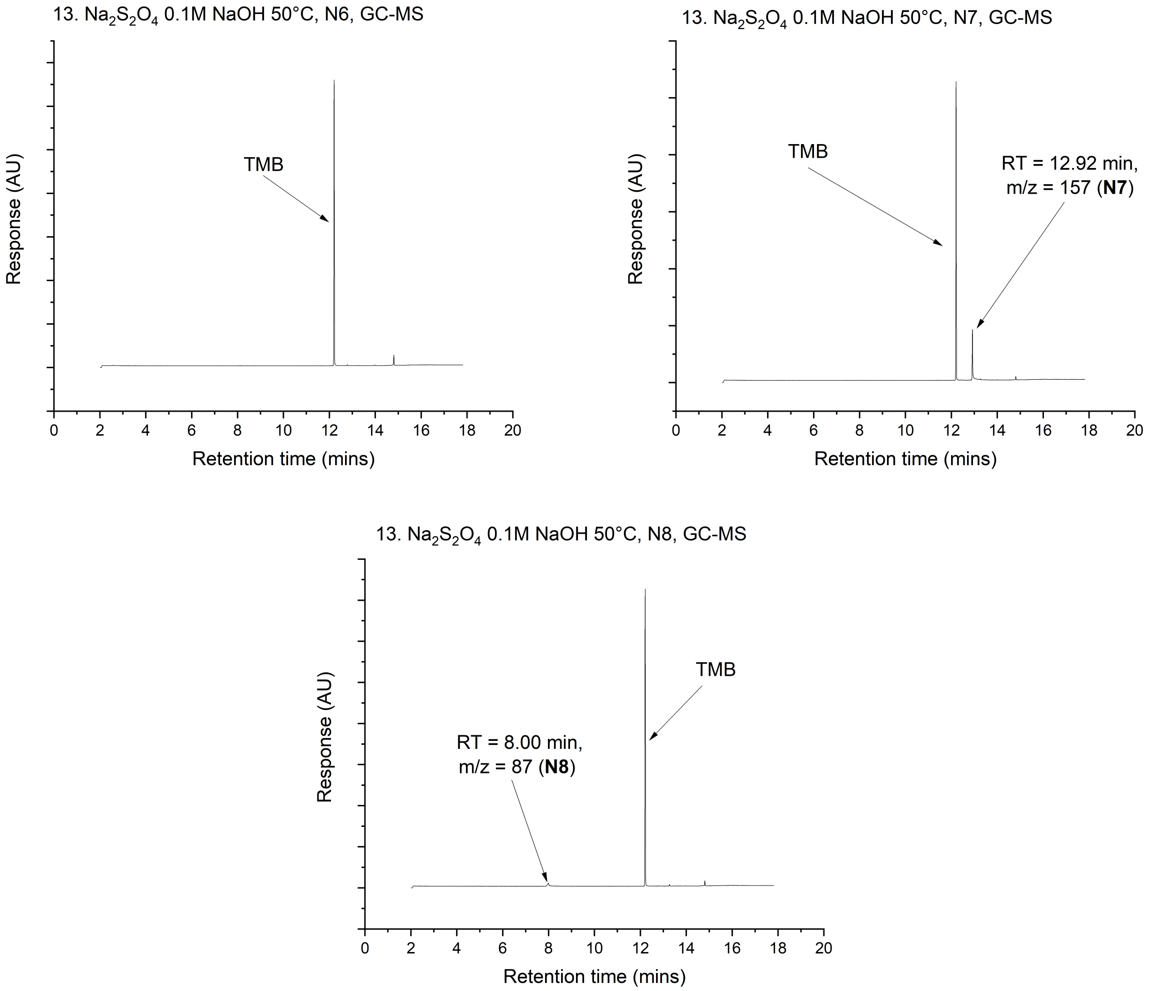

Figure S150: GC-MS/MS plots for N6-N8 for condition 13. As referenced in Section 3.3

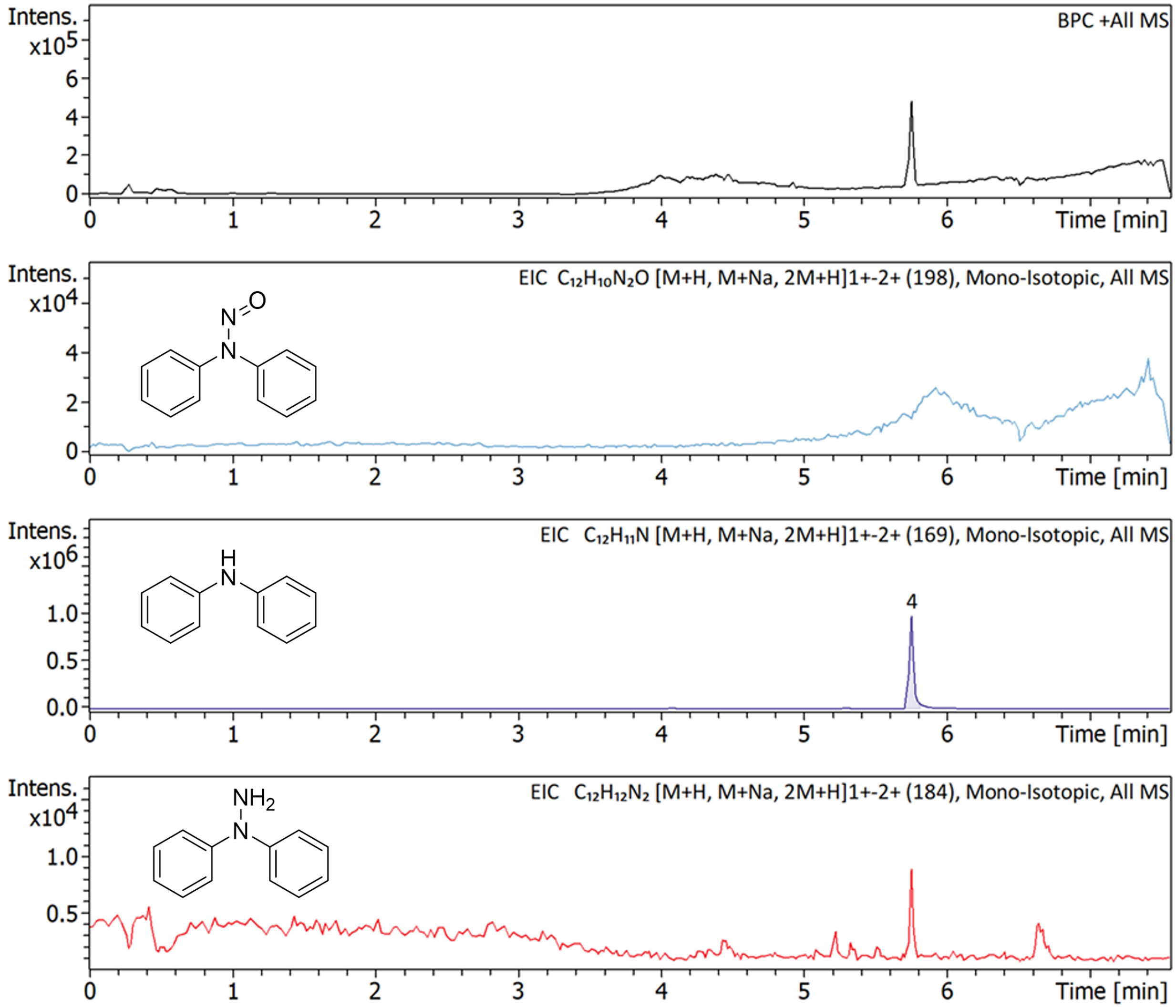

Summary of Results

Name	RT	BPC Area(%)	UV Area(%)	Confirm Formula Results
Cmpd 4, 5.7 min	5.75	no peak	80.9	C12H11N

Figure S151: LC-MS/MS experiment chromatogram for *N*-nitrosodiphenylamine (N4) in reaction condition 13. Referenced in Section 3.3.

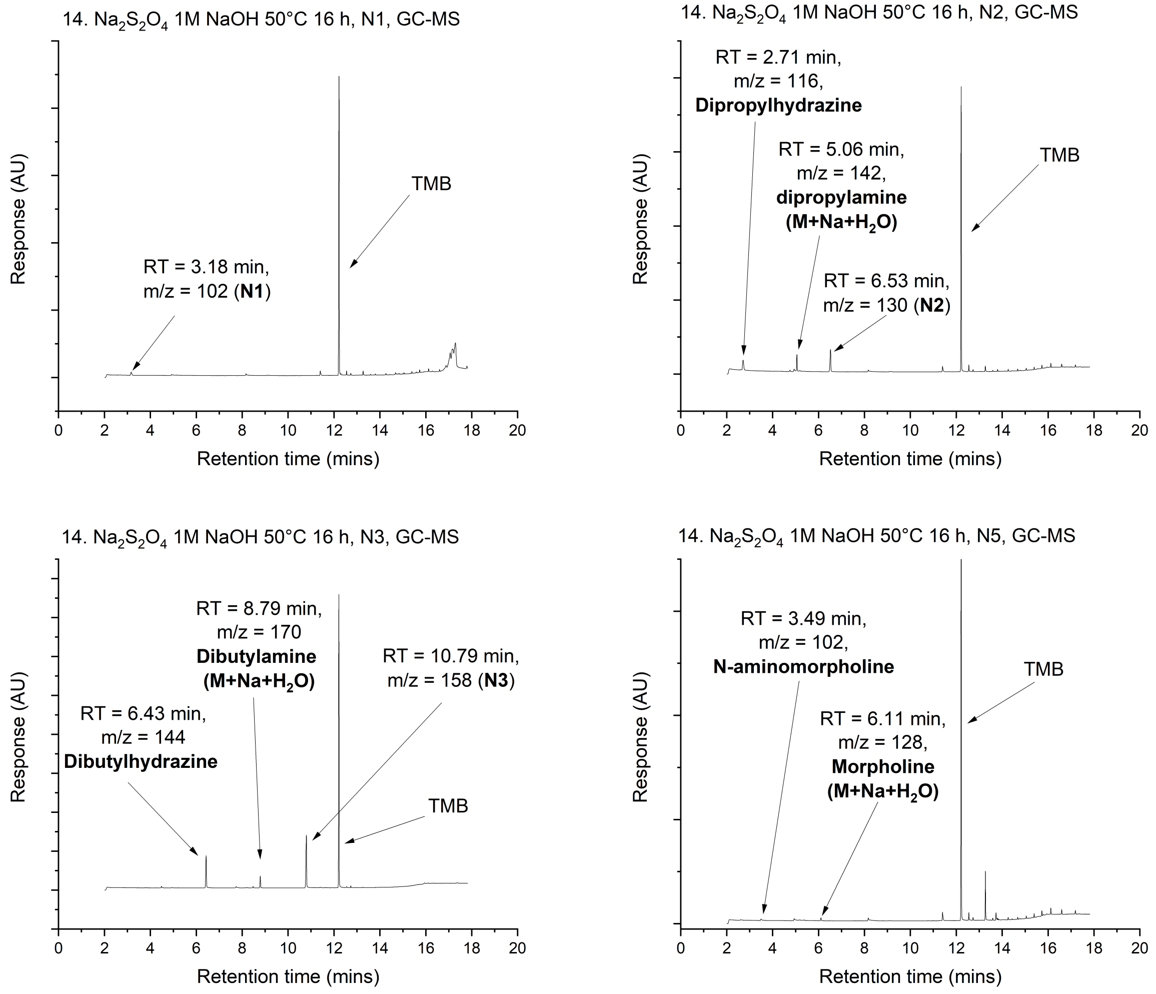

Figure S152: GC-MS/MS plots for N1, N2, N3 and N5 condition 14. As referenced in Section 3.3

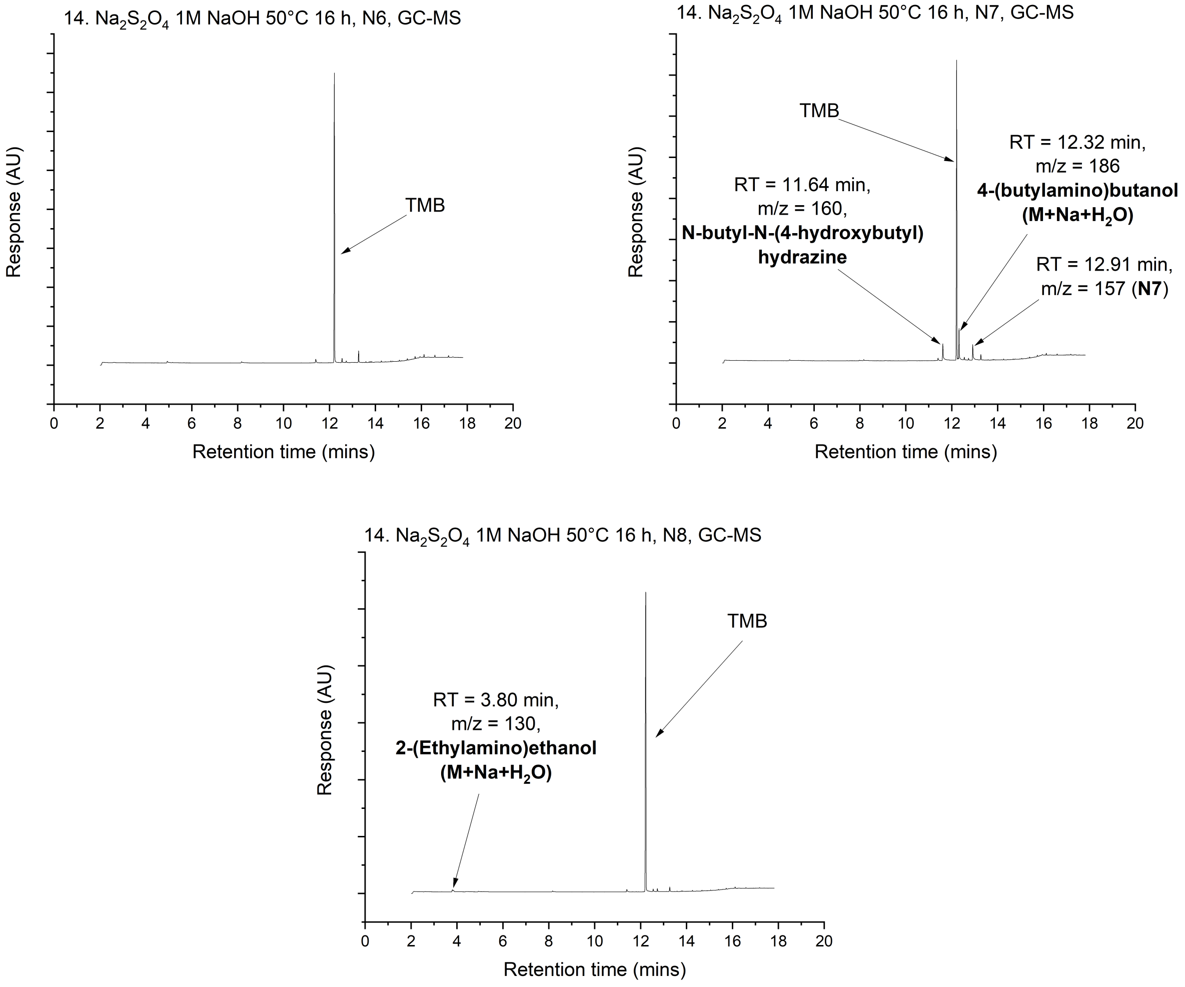

Figure S153: GC-MS/MS plots for N6-N8 for condition 14. As referenced in Section 3.3

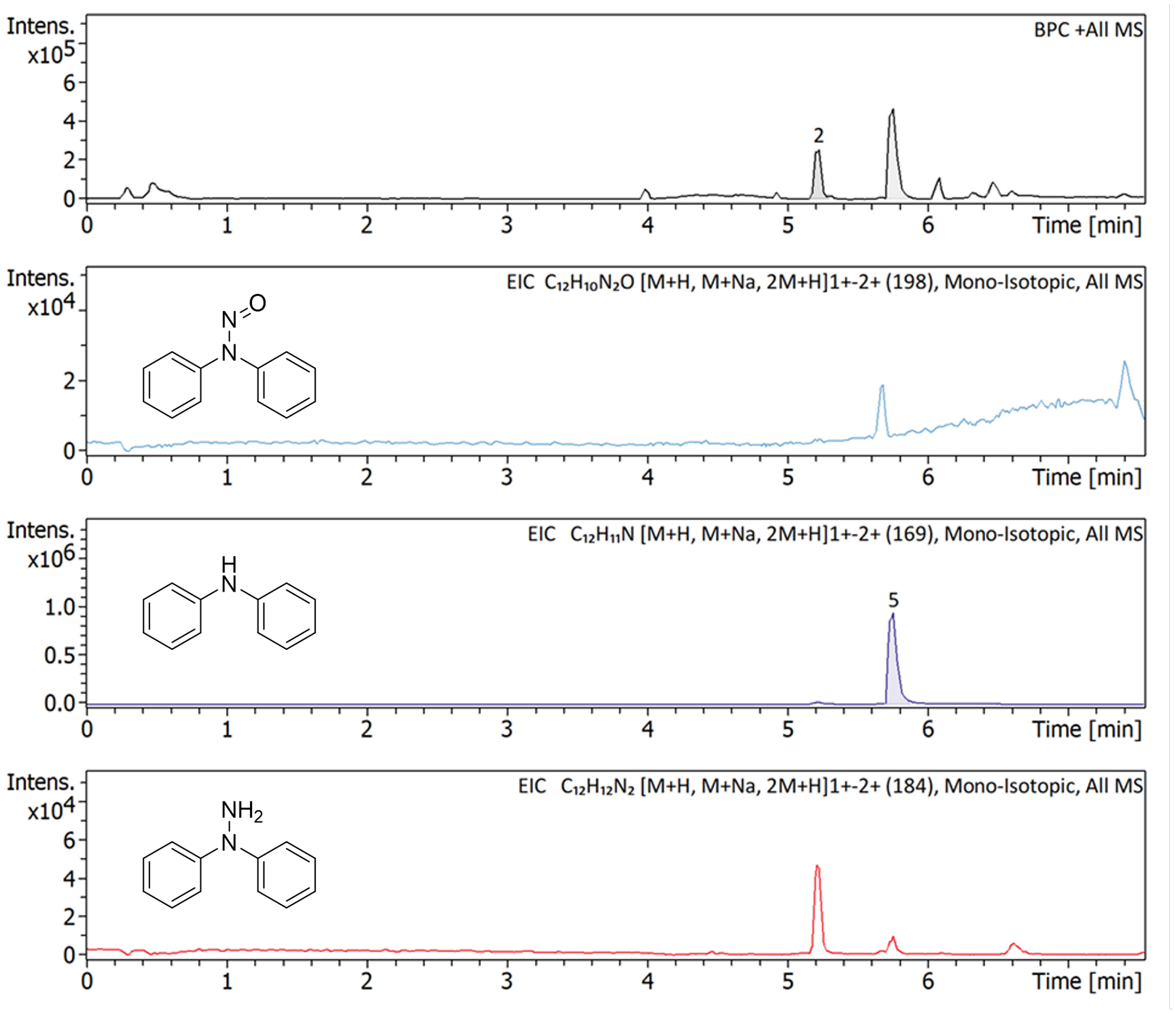

Summary of Results

Name	RT	BPC Area(%)	UV Area(%)	Confirm Formula Results
Cmpd 2, 5.2 min	5.22	30.8	no peak	C12H12N2
Cmpd 5, 5.8 min	5.75	69.2	25.6	C12H11N

Figure S154: LC-MS/MS experiment chromatogram for *N*-nitrosodiphenylamine (N4) in reaction condition 14. Referenced in Section 3.3

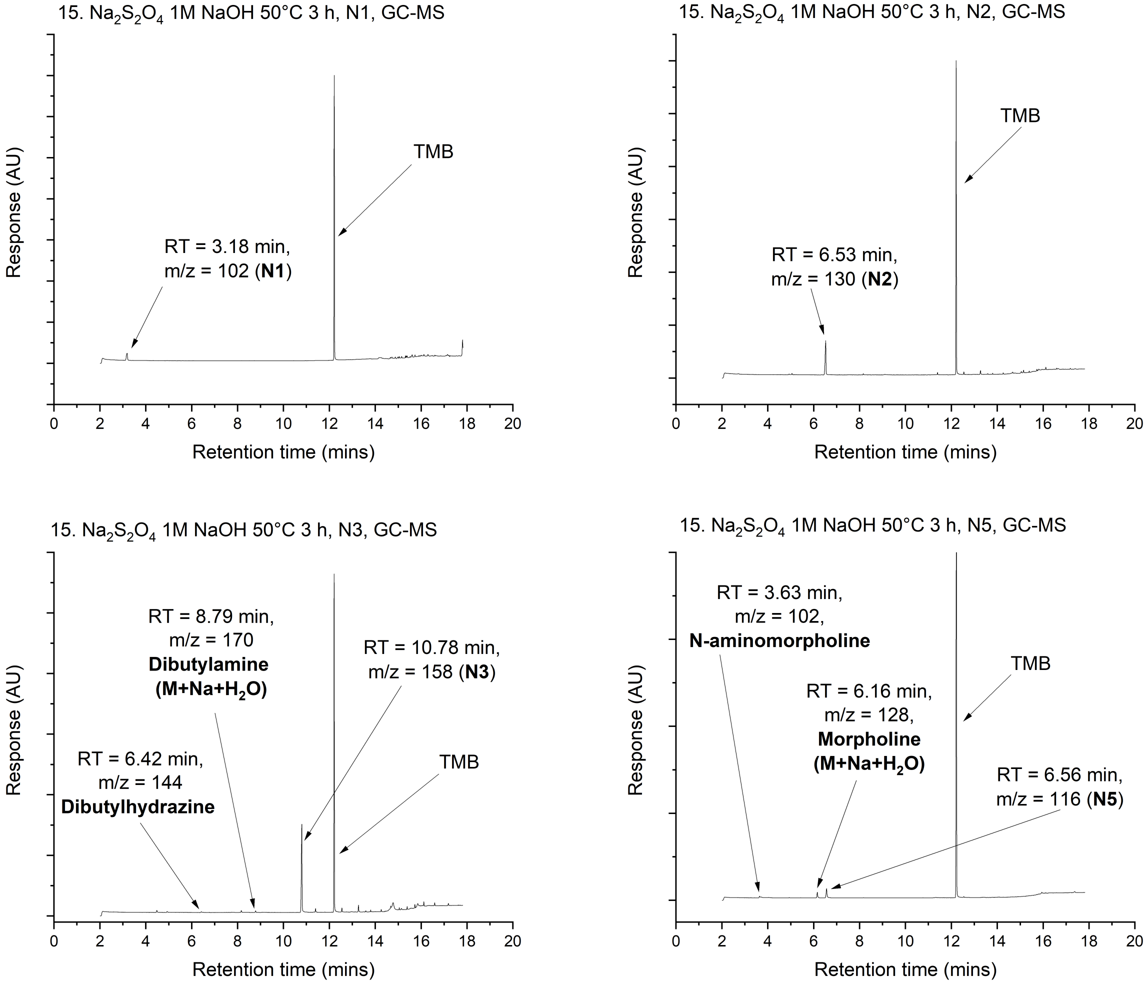

Figure S155: GC-MS/MS plots for N1, N2, N3 and N5 condition 15. As referenced in Section 3.3

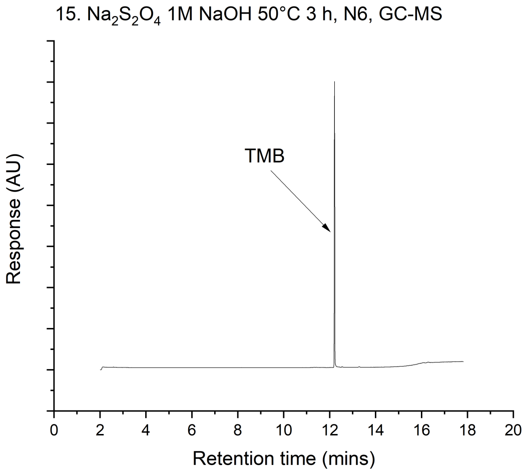

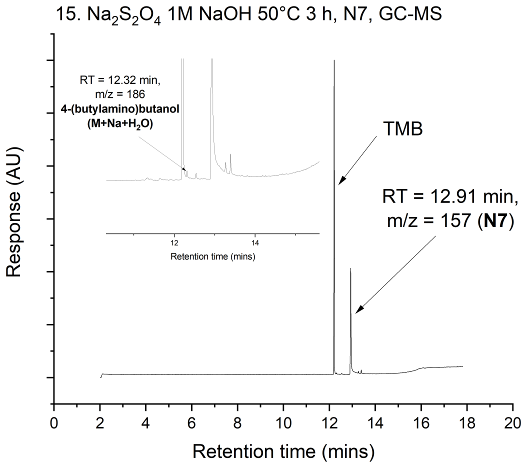

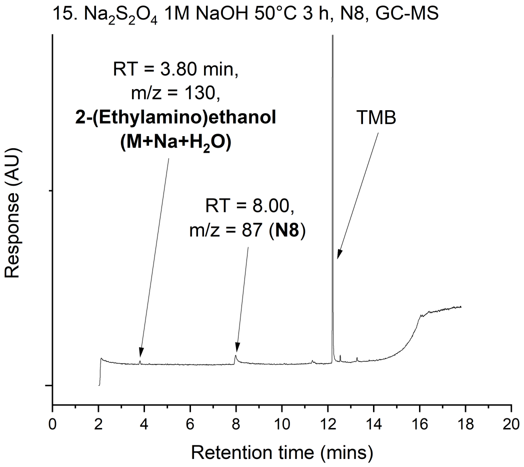

Figure S156: GC-MS/MS plots for N6-N8 for condition 15. As referenced in Section 3.3

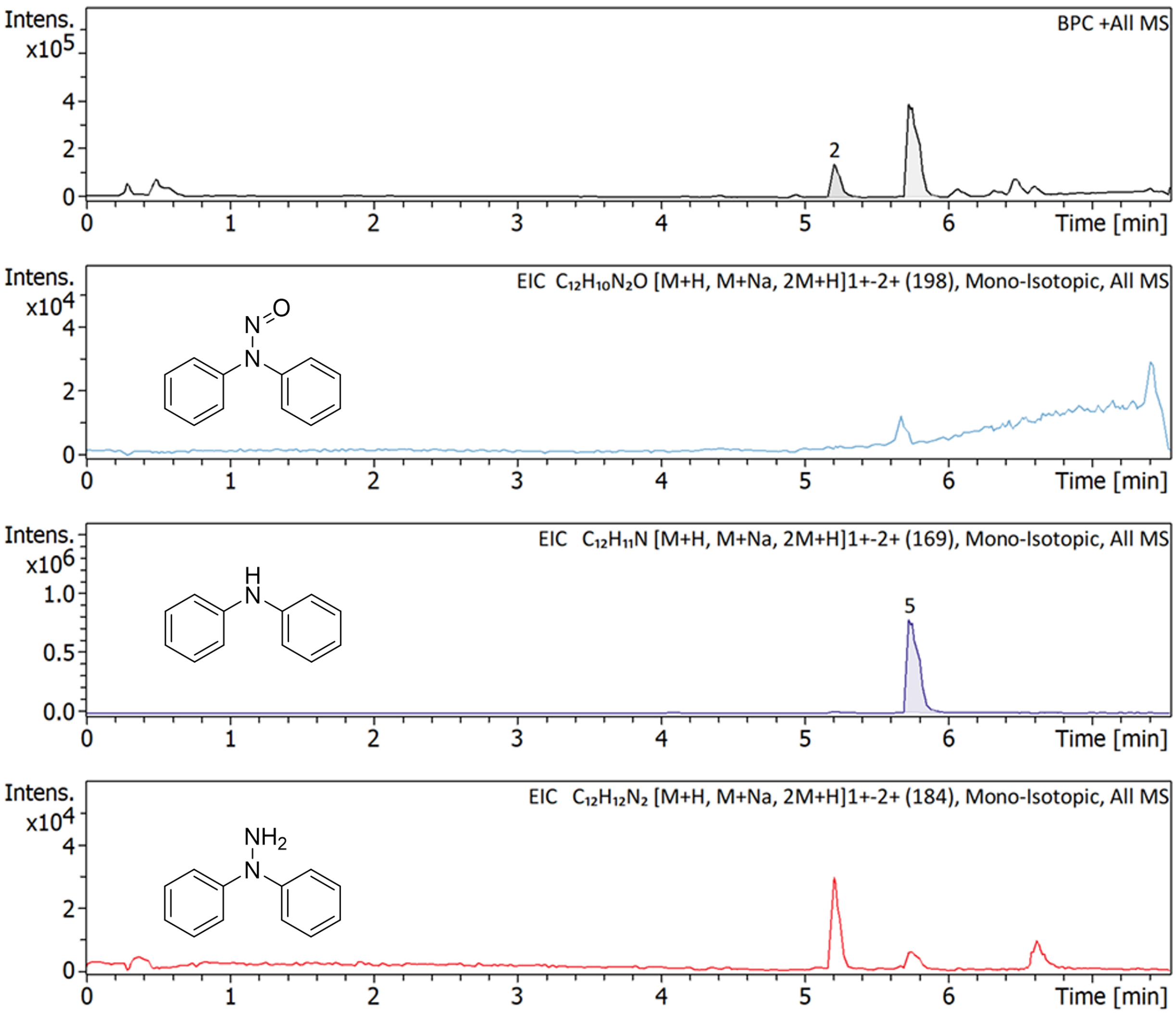

Summary of Results

Name	RT	BPC Area(%)	UV Area(%)	Confirm Formula Results
Cmpd 2, 5.2 min	5.21	19.1	no peak	C12H12N2
Cmpd 5, 5.7 min	5.73	80.9	89.1	C12H11N

Figure S157: LC-MS/MS experiment chromatogram for N-nitrosodiphenylamine (N4) in reaction condition 15. Referenced in Section 3.3

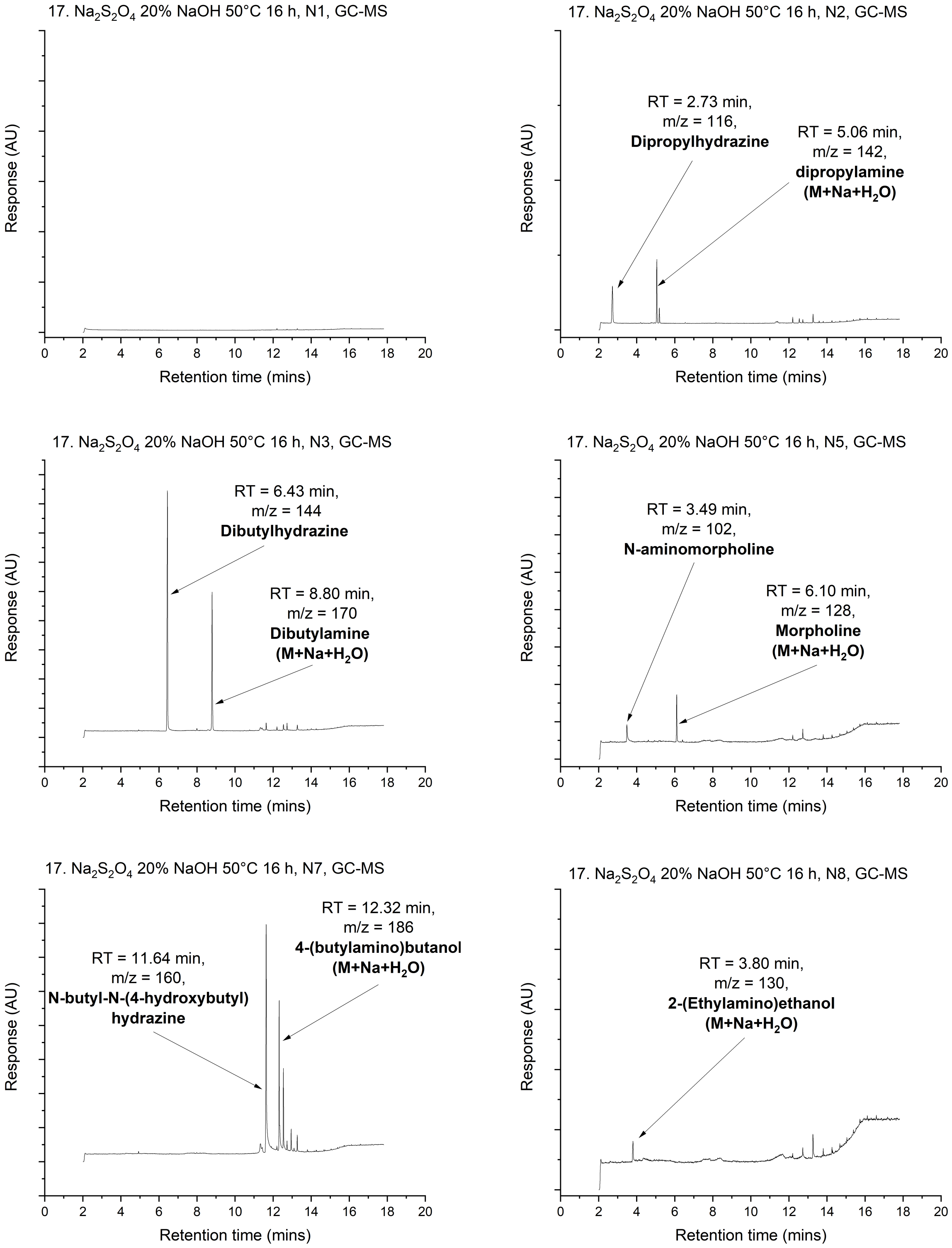

Figure S158: GC-MS/MS plots for N1-3, N5, N7 and N8 for condition 17. As referenced in Section 3.3

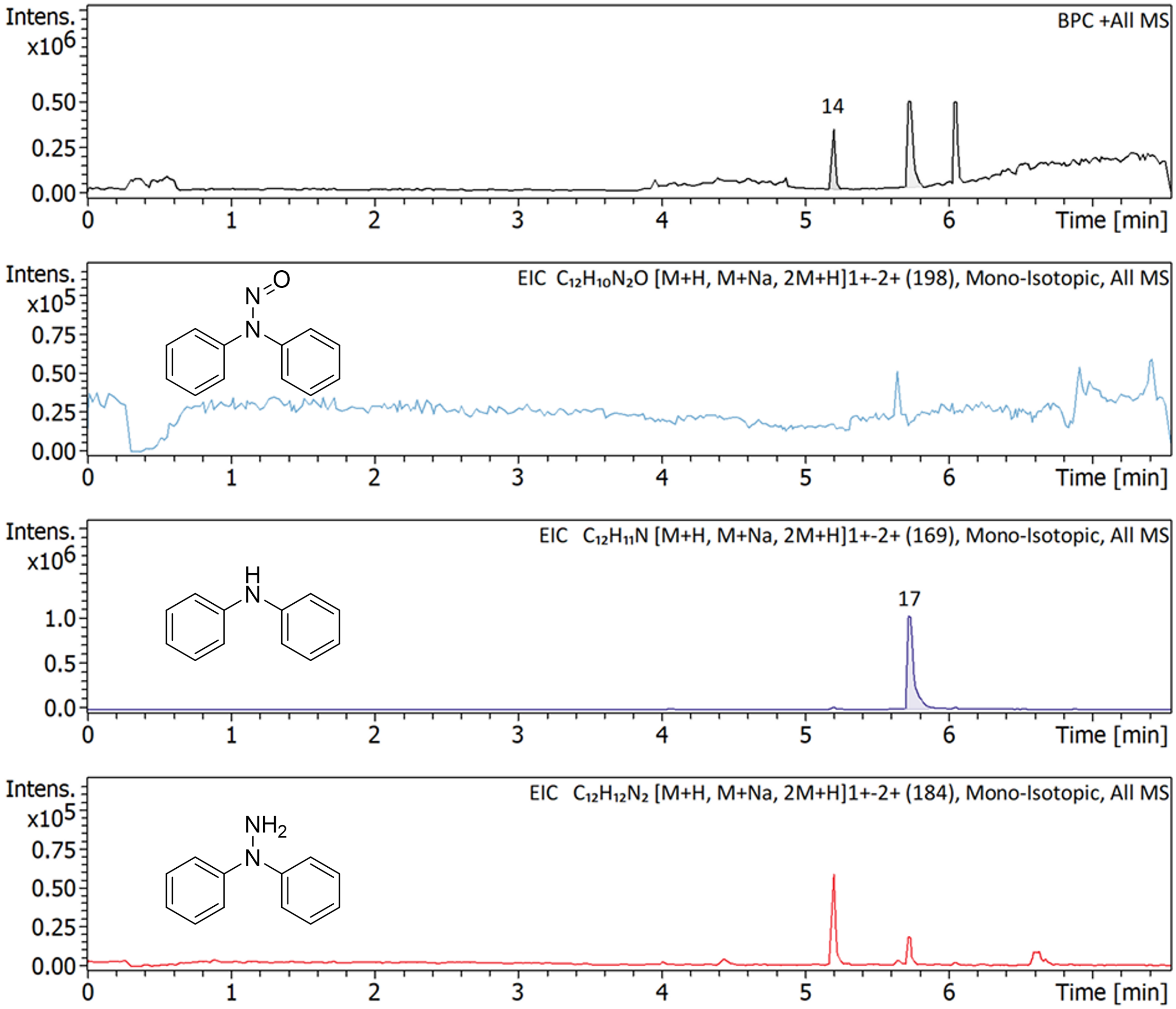

Summary of Results

Name	RT	BPC Area(%)	UV Area(%)	Confirm Formula Results
Cmpd 14, 5.2 min	5.19	40.5	1.8	C12H12N2
Cmpd 17, 5.7 min	5.73	59.5	78.0	C12H11N

Figure S159: LC-MS/MS experiment chromatogram for *N*-nitrosodiphenylamine (N4) in reaction condition 17. Referenced in Section 3.3

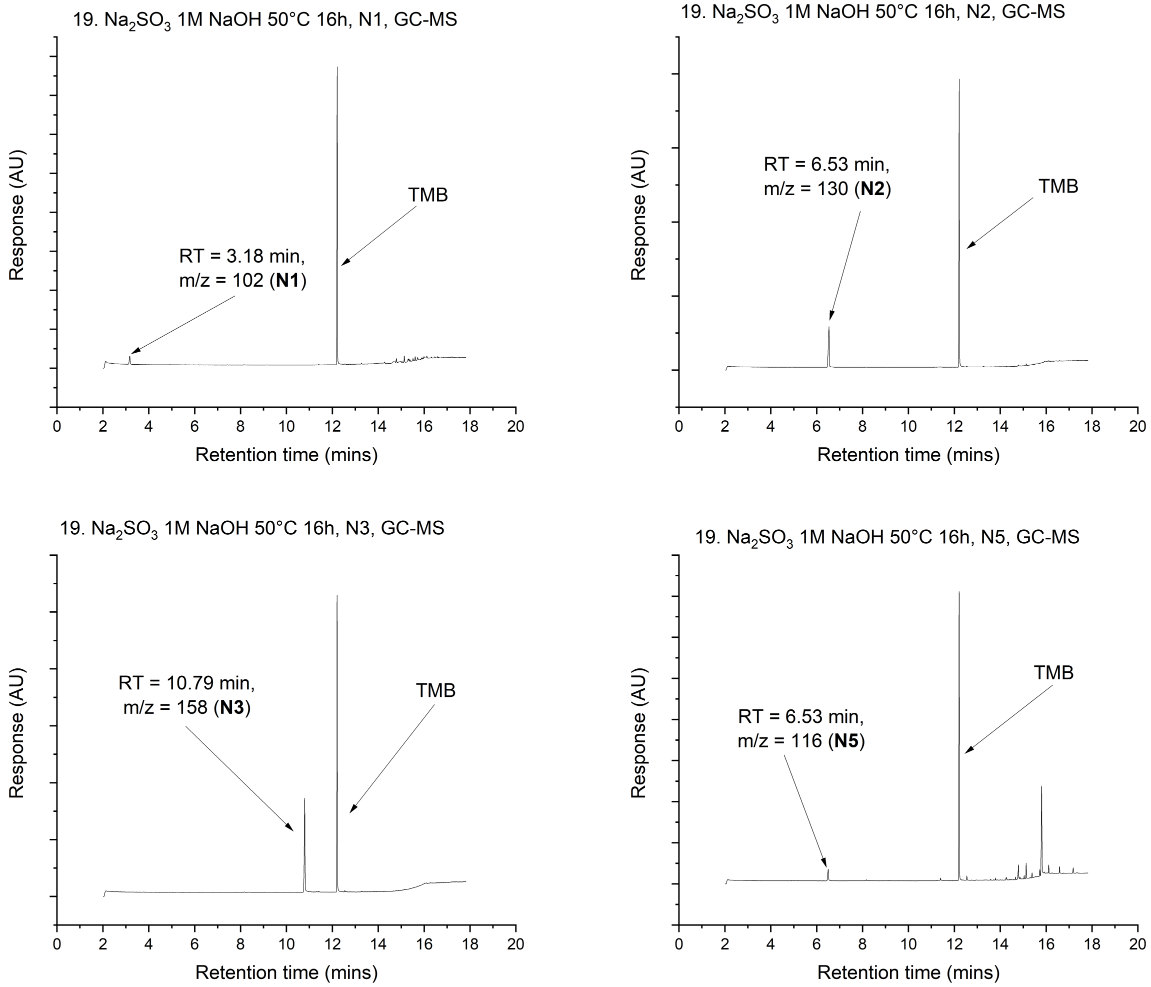

Figure S160: GC-MS/MS plots for N1, N2, N3 and N5 condition 19. As referenced in Section 3.3

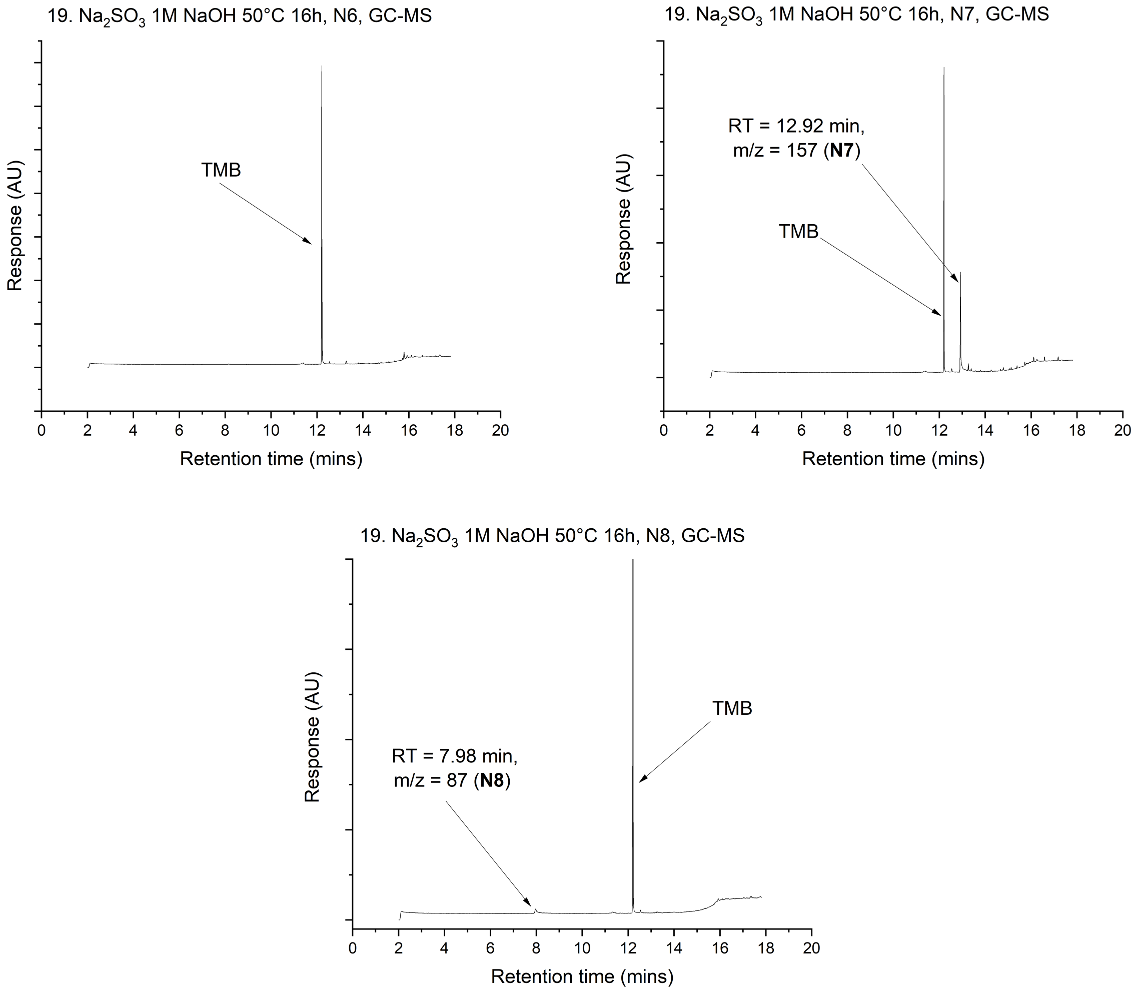

Figure S161: GC-MS/MS plots for N6-N8 for condition 19. As referenced in Section 3.3

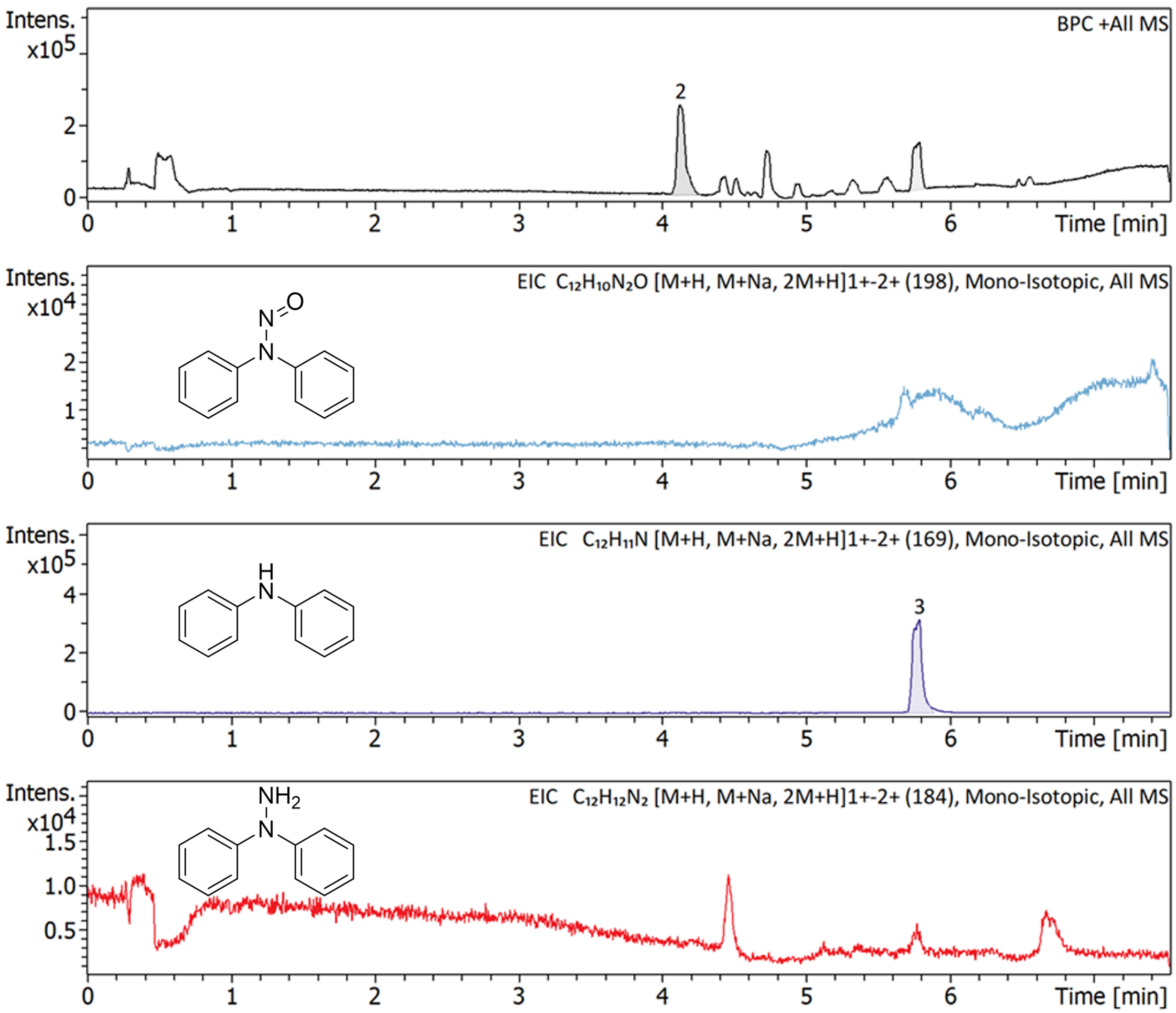

Summary of Results

Name	RT	BPC Area(%)	UV Area(%)	Confirm Formula Results
Cmpd 2, 4.1 min	4.13	66.7	no peak	
Cmpd 3, 5.8 min	5.79	33.3	no peak	C12H11N

Figure S162: LC-MS/MS experiment chromatogram for *N*-nitrosodiphenylamine (N4) in reaction condition 19. Referenced in Section 3.3

9.7 Basic conditions (20-21):

a)

Key:	0	50	100
conversion (%)			
No data			

No.	Class	Reagents	Solvent	Temp. (°C)	Reaction time (h)	N1	N2	N3	N4	N5	N6	N7	N8
20	Base	NaOH 2M	H_2O/EtOH	22	24	0	-5	3	1	0	101	6	6
21	Base	NaOEt 21% w/v	EtOH	22	24	-7	1	6	-3	3	101	2	7

b)

Key:		
	Found	
	Absent	
	No Data	

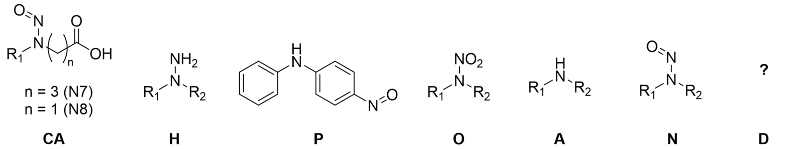

	N1				N2				N3				N4					N5			N6		N7				N8				
No.	N	H	A	O	N	H	A	O	N	H	A	O	N	H	A	P	O	N	H	A	N	A	N	H	A	CA	N	H	A	CA	D
20																															
21																															

Figure S163: Reactivity of *N*-nitrosamines under acidic conditions 20-21. a) Conversions with respect to the *N*-nitrosamine and b) Products of the reactions determined by GC-MS/MS and LC-MS/MS.

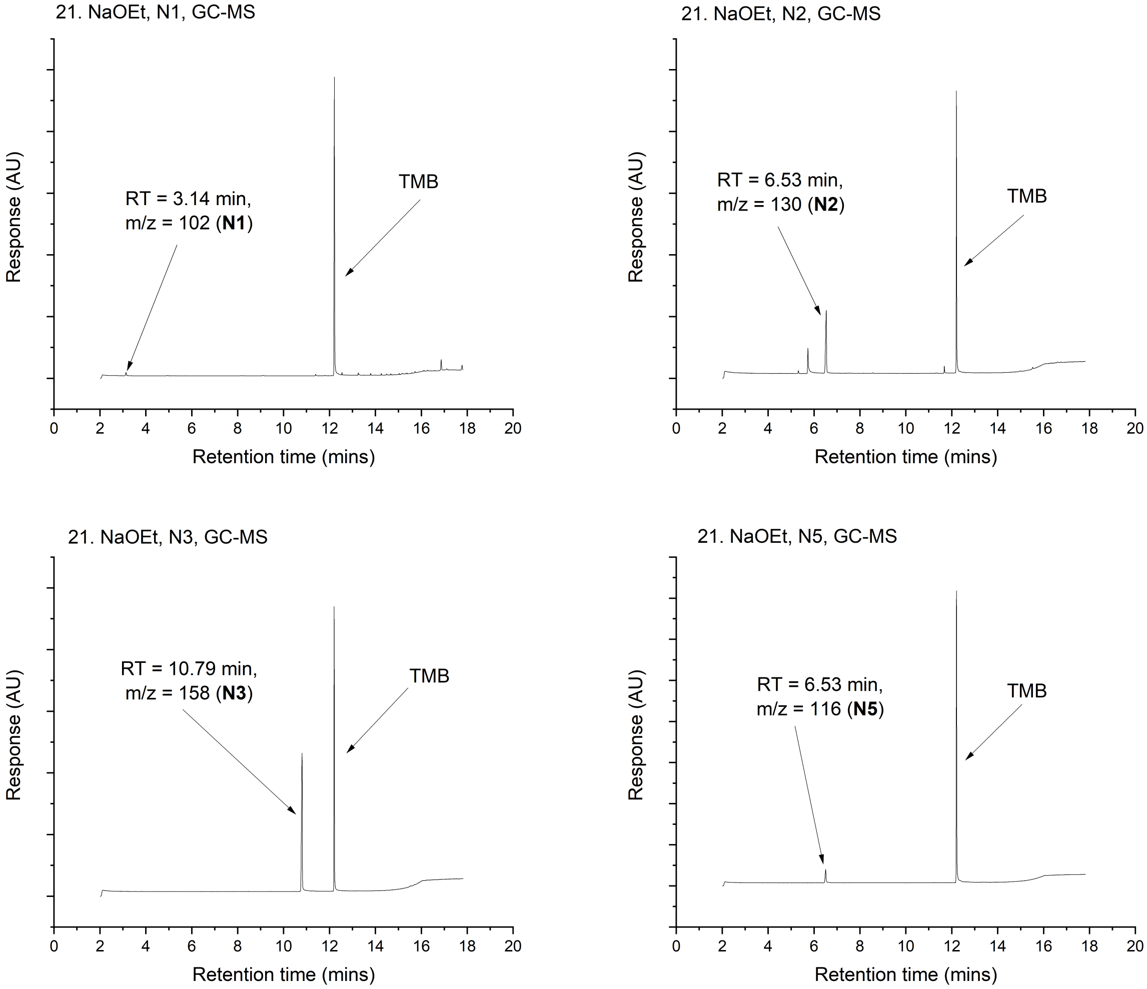

Figure S164: GC-MS/MS plots for N1, N2, N3 and N5 condition 21. As referenced in Section 3.3

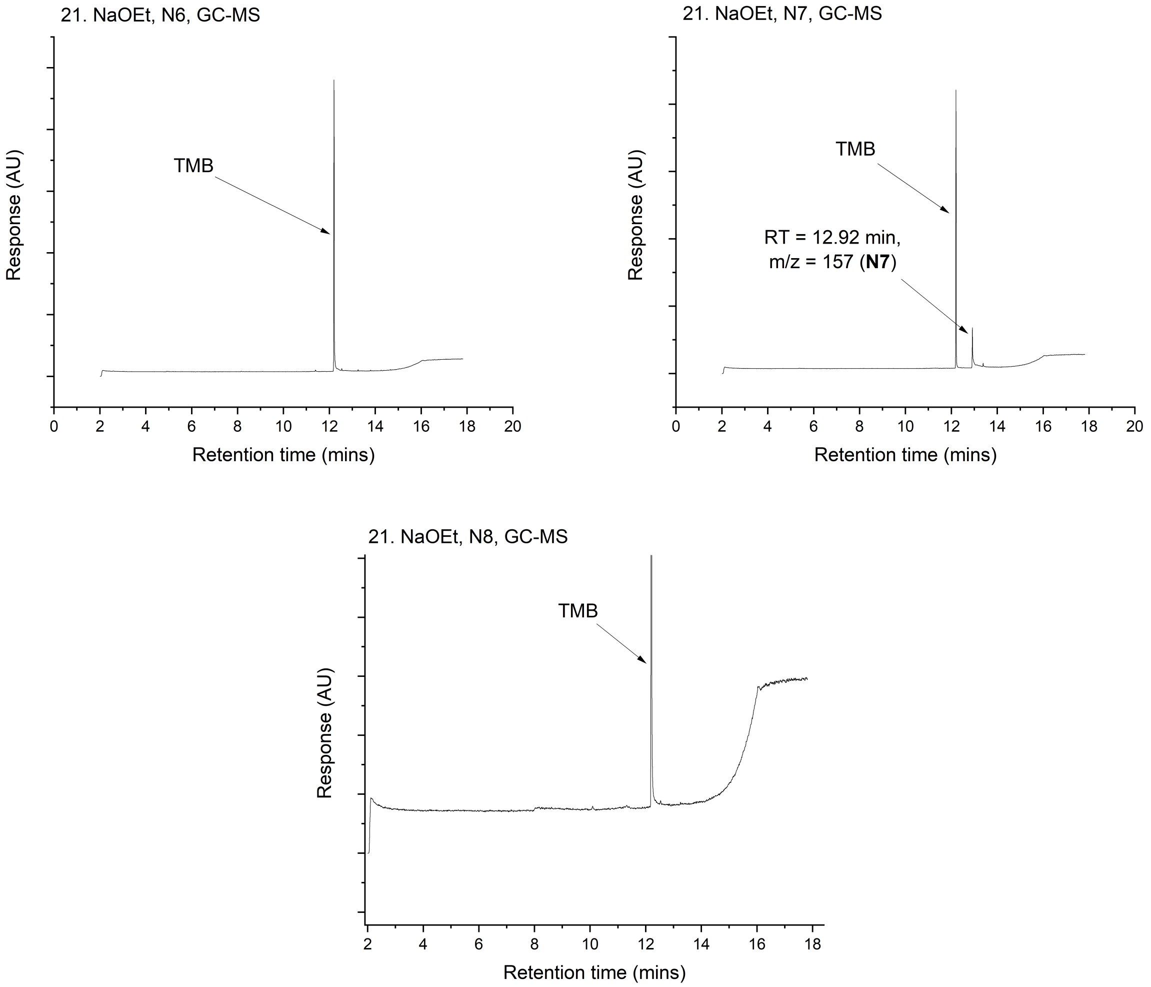

Figure S165: GC-MS/MS plots for N6-N8 for condition 21. As referenced in Section 3.3

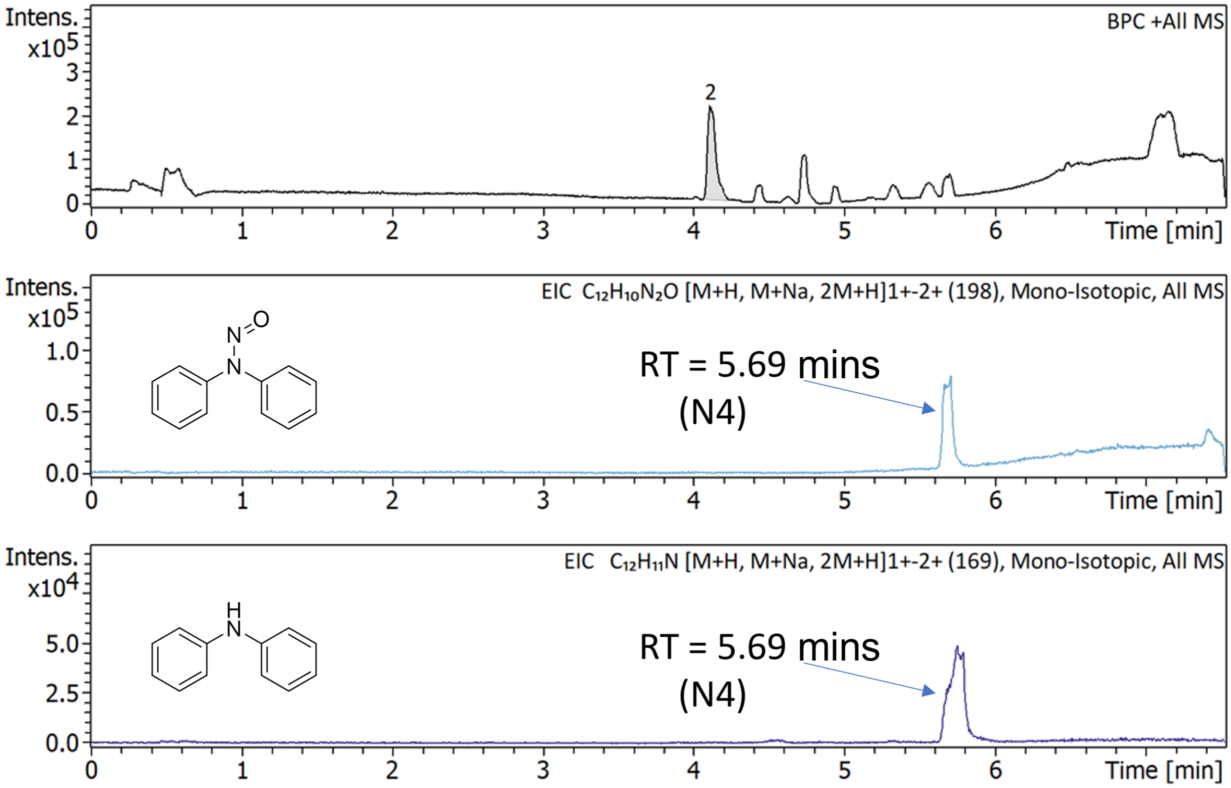

Figure S166: LC-MS/MS experiment chromatogram for *N*-nitrosodiphenylamine (N4) in reaction condition 21. Referenced in Section 3.3

10 Spectroscopic data of reaction products

10.1 NMR spectra

10.1.1 Diphenylamine ^{1}H NMR

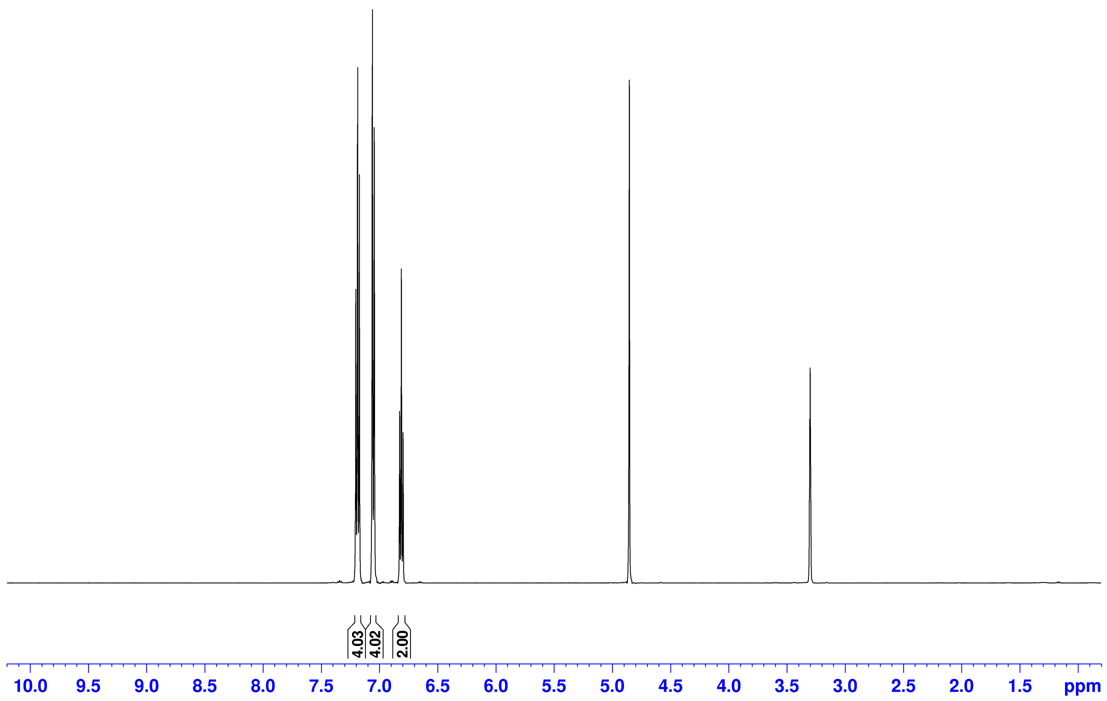

Figure S167: ^{1}H NMR spectrum of diphenylamine

^{1}H NMR δ(MeOD, 500 MHz): 7.21-7.17 (4H, m), 7.06-7.04 (4H, m), 6.83-6.79 (2H, m)

10.1.2 Diphenylamine ^{13}C NMR

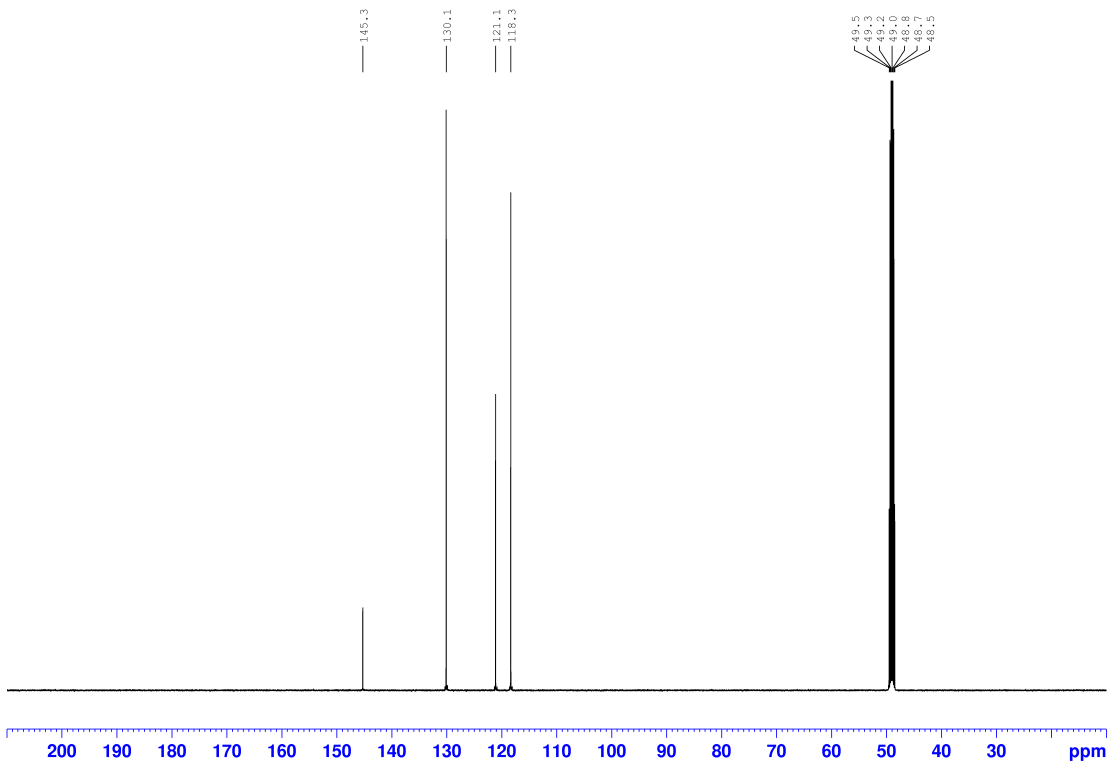

Figure S168: ^{13}C NMR spectrum of diphenylamine

^{13}C NMR, δ (MeOD, 125 MHz): 145.3, 130.1, 121.1, 118.3

10.1.3 *N*-nitrosodiphenylamine (N4) ^{1}H NMR

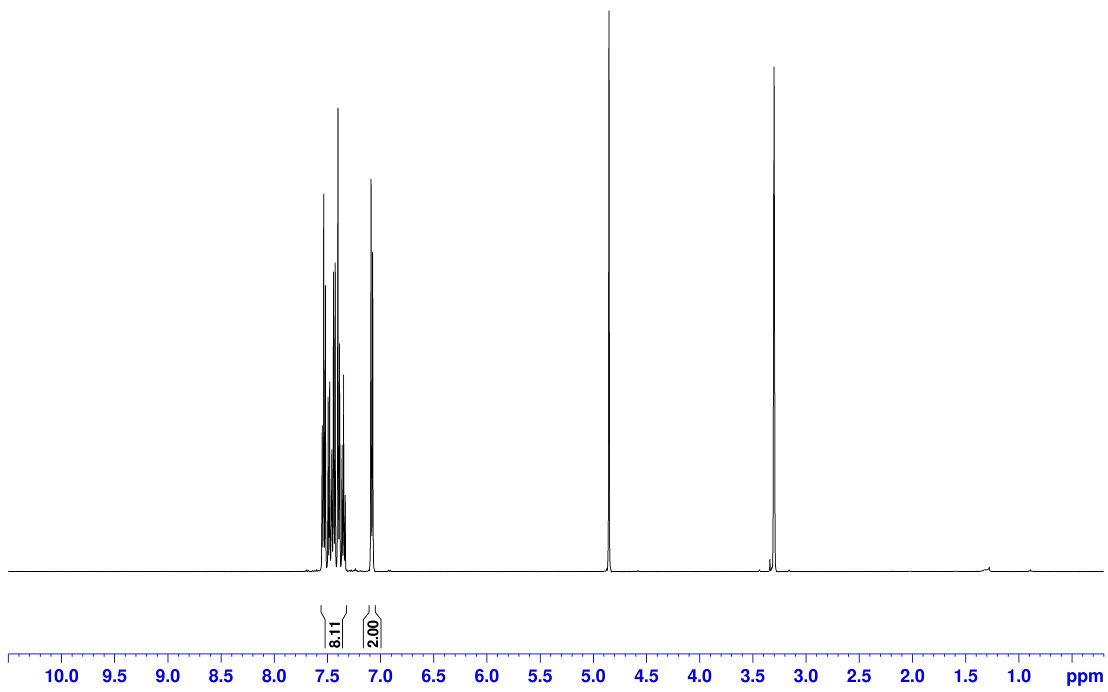

Figure S169: ^{1}H NMR spectrum of *N*-nitrosodiphenylamine (N4).

^{1}H NMR δ(MeOD, 500 MHz): 7.55-7.33 (8H, m), 7.09-7.07 (2H, m)

10.1.4 *N*-nitrosodiphenylamine (N4) ^{13}C NMR

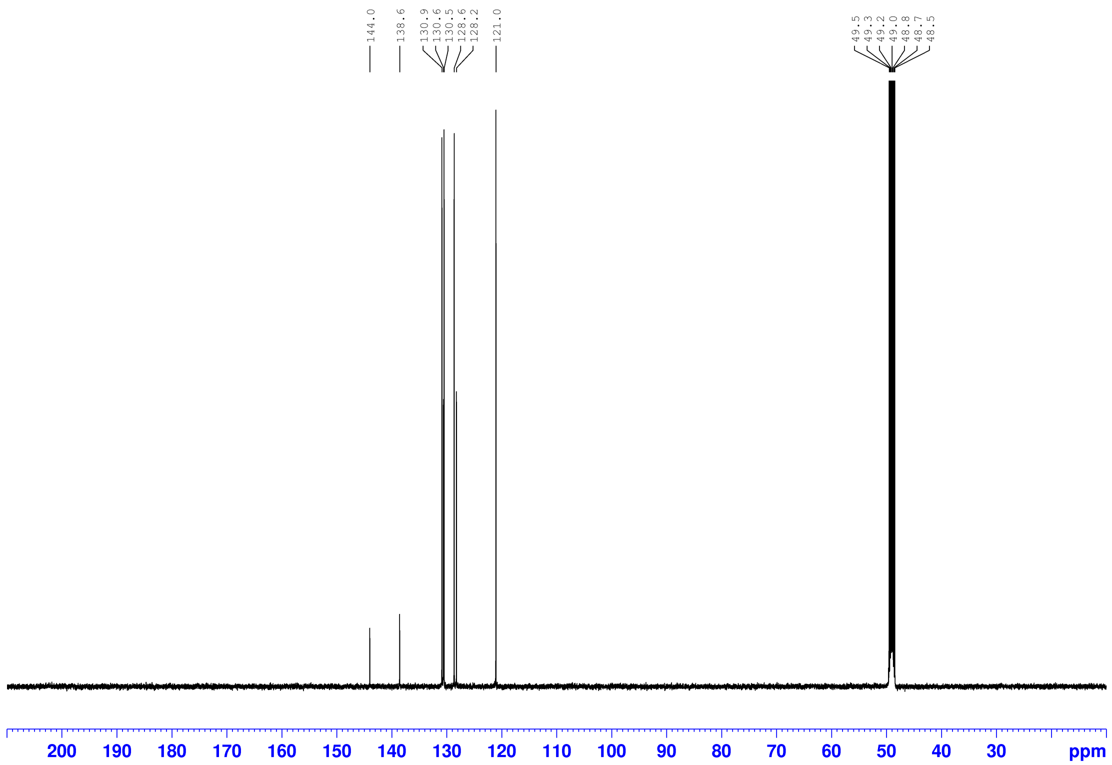

Figure S170: ^{13}C NMR spectrum of *N*-nitrosodiphenylamine (N4).

^{13}C NMR, δ (MeOD, 125 MHz): 144.0, 138.6, 130.9, 130.6, 130.5, 128.6, 128.2, 121.0

10.1.5 Condition 1 N4 ^{1}H NMR overlay

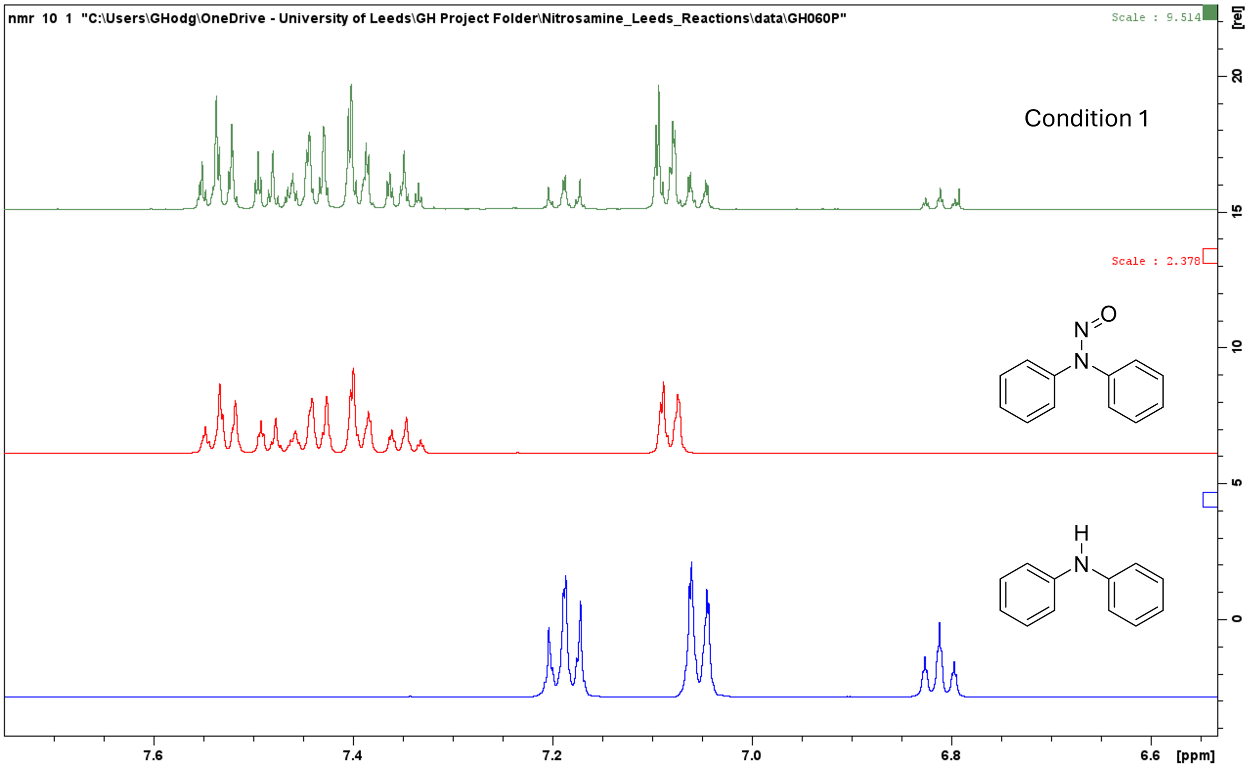

Figure S171: Overlay ^{1}H NMR spectra of *N*-nitrosodiphenylamine (N4), diphenylamine and the crude reaction NMR of N4 in condition 1.

10.1.6 Condition 1 N4 ^{13}C NMR overlay

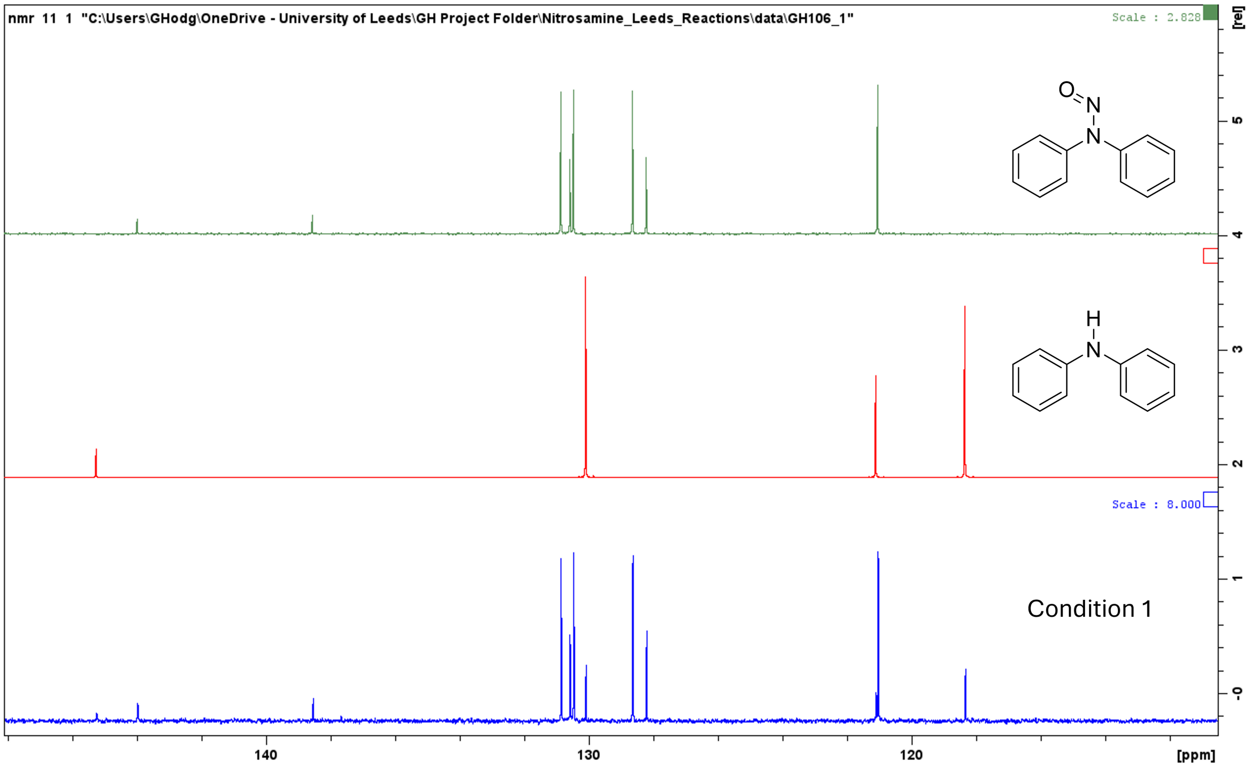

Figure S172: Overlay ^{13}H NMR spectra of *N*-nitrosodiphenylamine (N4), diphenylamine and the crude reaction NMR of N4 in condition 1.

10.1.7 Condition 1 N4 ^{1}H NMR (Full)

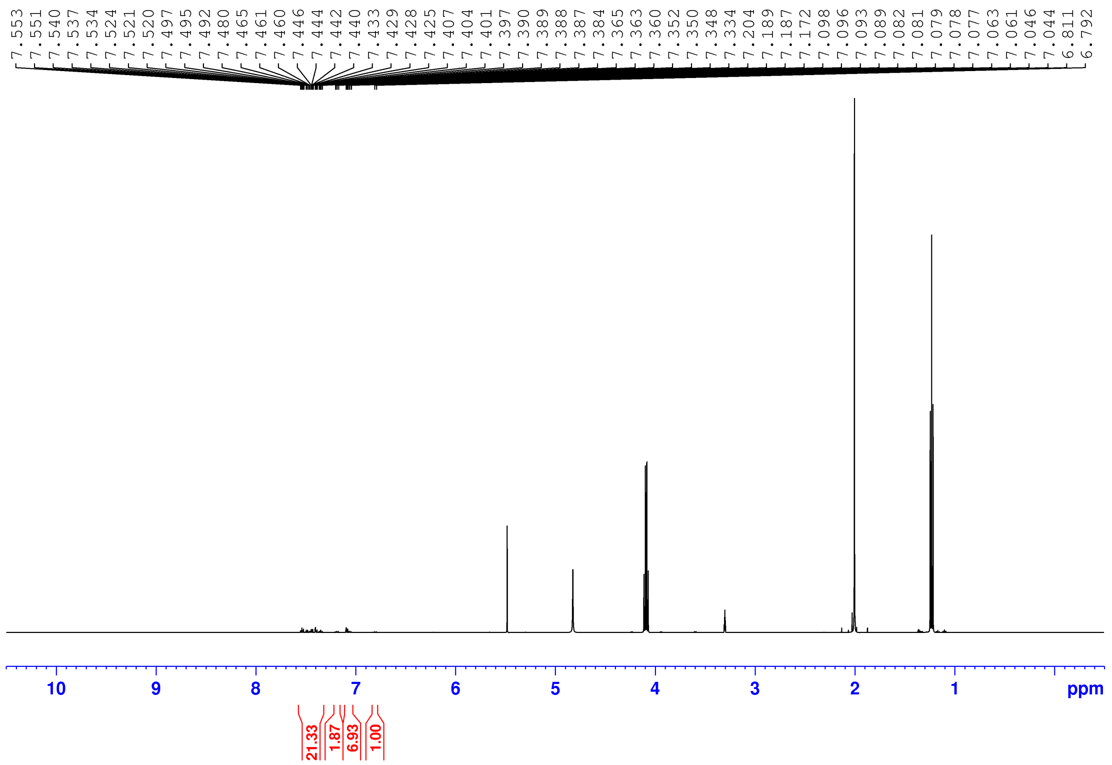

Figure S173: ^{1}H NMR spectrum of the crude reaction NMR of N4 in condition 1, showing the full spectrum.

10.1.8 Condition 1 N4 ^{1}H NMR (Zoomed)

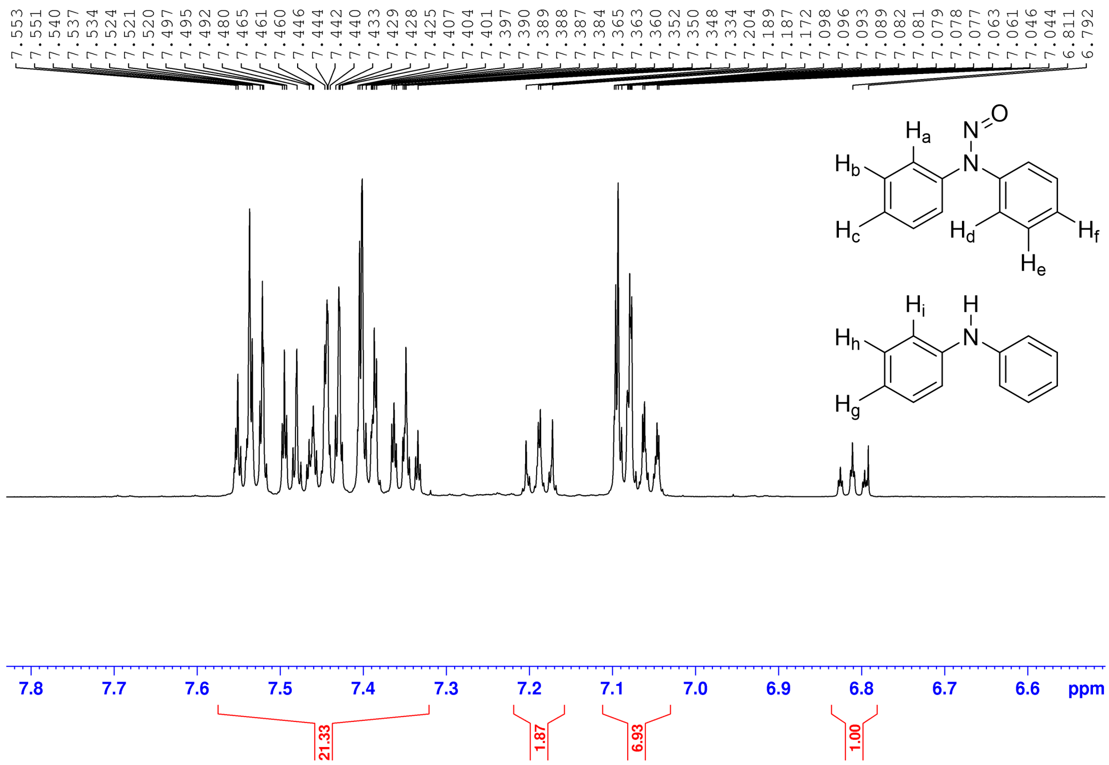

Figure S174: ^{1}H NMR spectrum of the crude reaction NMR of N4 in condition 1, showing the important peaks.

^{1}H NMR δ(MeOD, 500 MHz): 7.56-7.33 (8H, m, H*a, b, d, e*), 7.21-7.17 (2H, m, H*h*), 7.09-7.07 (2H, m, H*c*) 7.06-7.04 (2H, m, H*i*), 6.82-6.79 (2H, m, H*g*).

10.1.9 Condition 1 N4 ^{13}C NMR (Full)

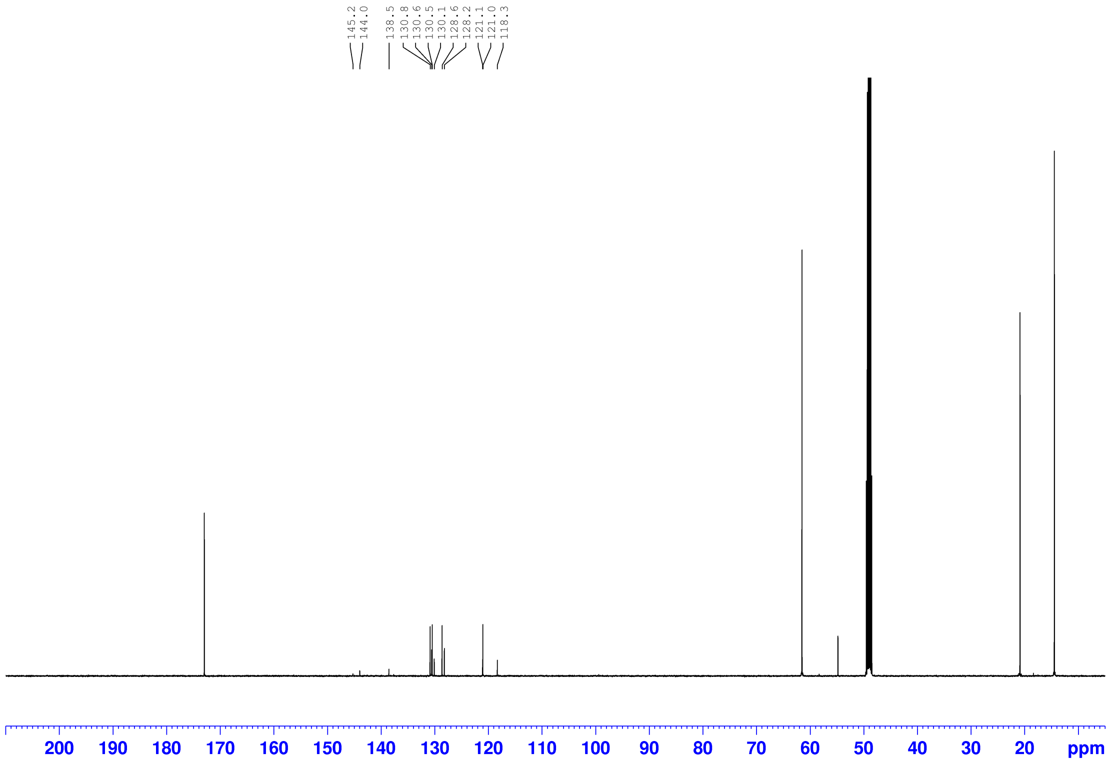

Figure S175: ^{13}C NMR spectrum of the crude reaction NMR of N4 in condition 1, showing the full spectrum.

10.1.10 Condition 1 N4 ^{13}C NMR (Zoomed)

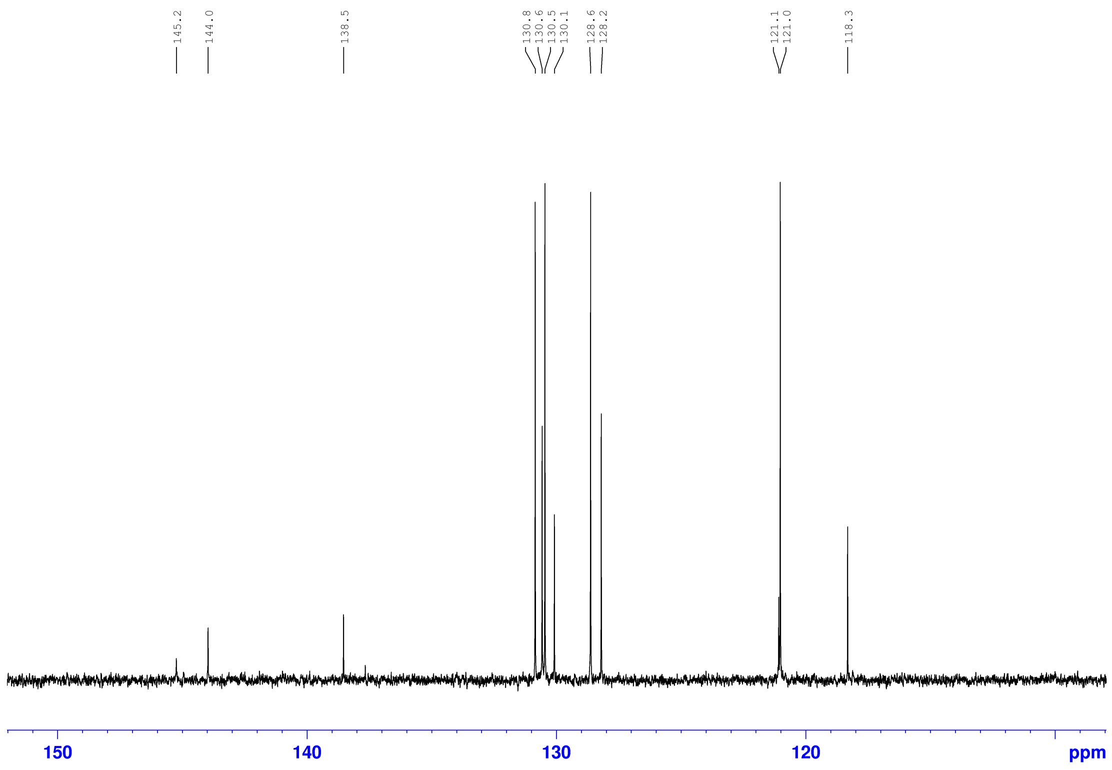

Figure S176: ^{13}C NMR spectrum of the crude reaction NMR of N4 in condition 1, showing the important peaks.

NDPA = *N*-nitrosodiphenylamine, DPA = diphenylamine

^{13}C NMR, δ (MeOD, 125 MHz): 145.2 (DPA), 144.0 (NDPA), 138.5 (NDPA), 130.9 (NDPA), 130.6 (NDPA), 130.5 (NDPA), 130.1 (DPA), 128.6 (NDPA), 128.2 (NDPA), 121.1 (DPA), 121.0 (NDPA), 118.3 (DPA)

10.1.11 Morpholine ^{1}H NMR analysis

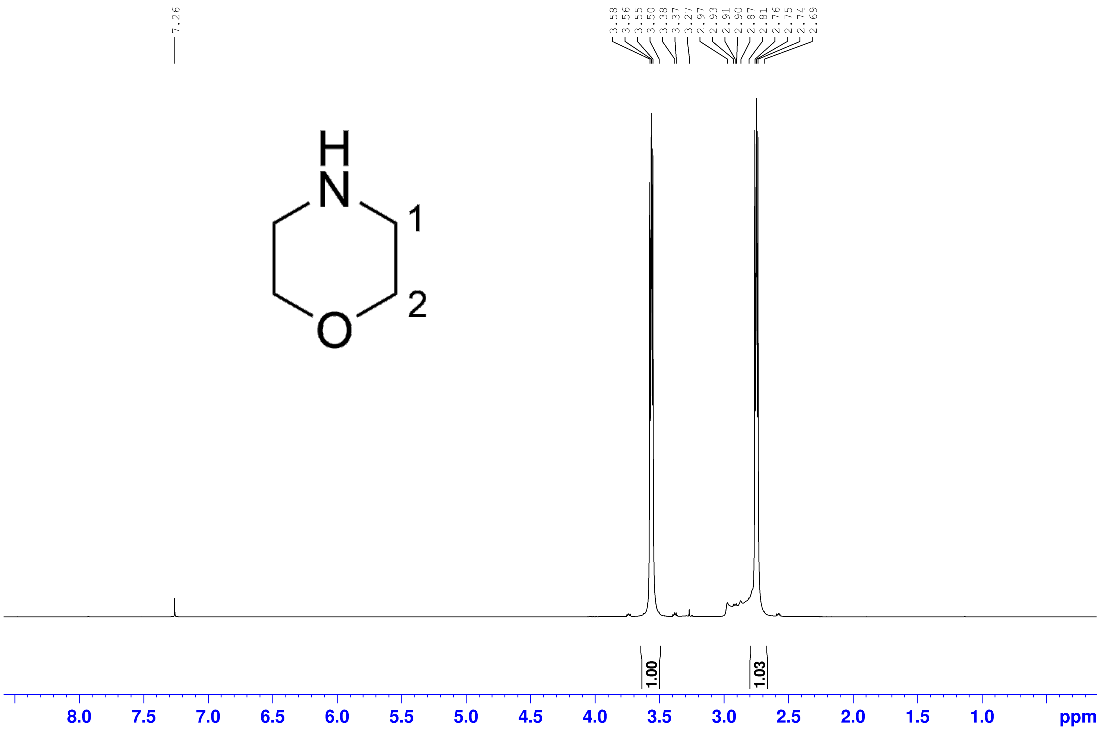

Figure S177: ^{1}H NMR spectrum of morpholine.

^{1}H NMR δ($CDCl_3$, 500 MHz): 3.56 (4H, t, J = 4.9 Hz, H2), 2.75 (4H, t, J = 4.8 Hz, H1)

10.1.12 Morpholine ^{13}C NMR analysis

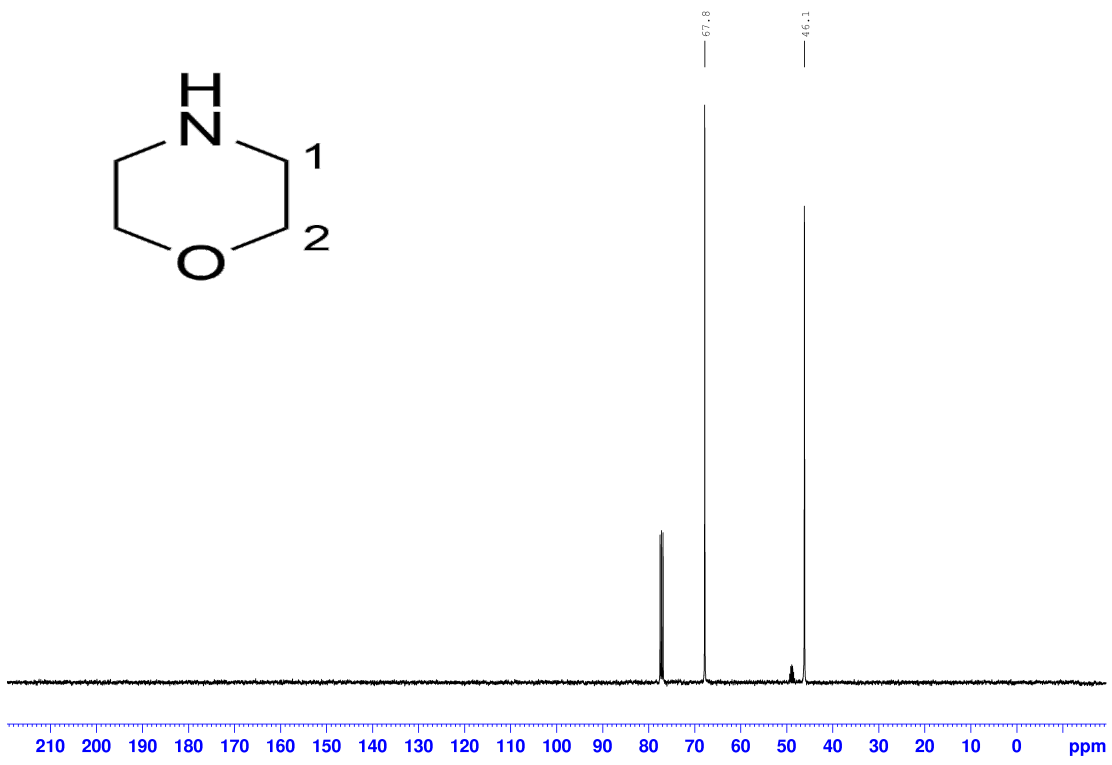

Figure S178: ^{13}C NMR spectrum of morpholine.

^{13}C NMR, δ ($CDCl_3$, 125 MHz): 67.8 (C2), 46.1 (C1)

10.1.13 *N*-nitrosmorpholine (N5) ^{1}H NMR analysis

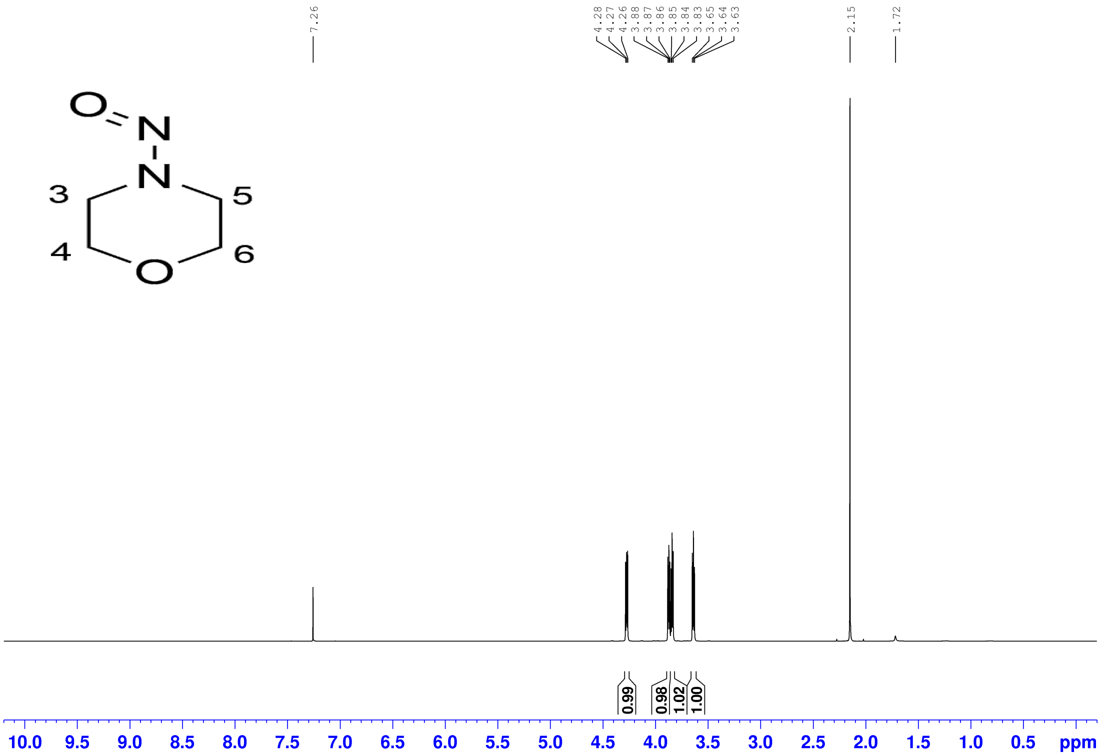

Figure S179: ^{1}H NMR spectrum of *N*-nitrosomorpholine.

^{1}H NMR δ($CDCl_3$, 500 MHz): 4.27 (2H, t, J = 5.0 Hz, H6), 3.87 (2H, t, J = 5.0 Hz, H4), 3.84 (2H, t, J = 5.2 Hz, H5), 3.64 (2H, t, J = 5.2 Hz, H3), 2.15 (s, starting material impurity)

10.1.14 *N*-nitrosomorpholine (N5) ^{13}C NMR analysis

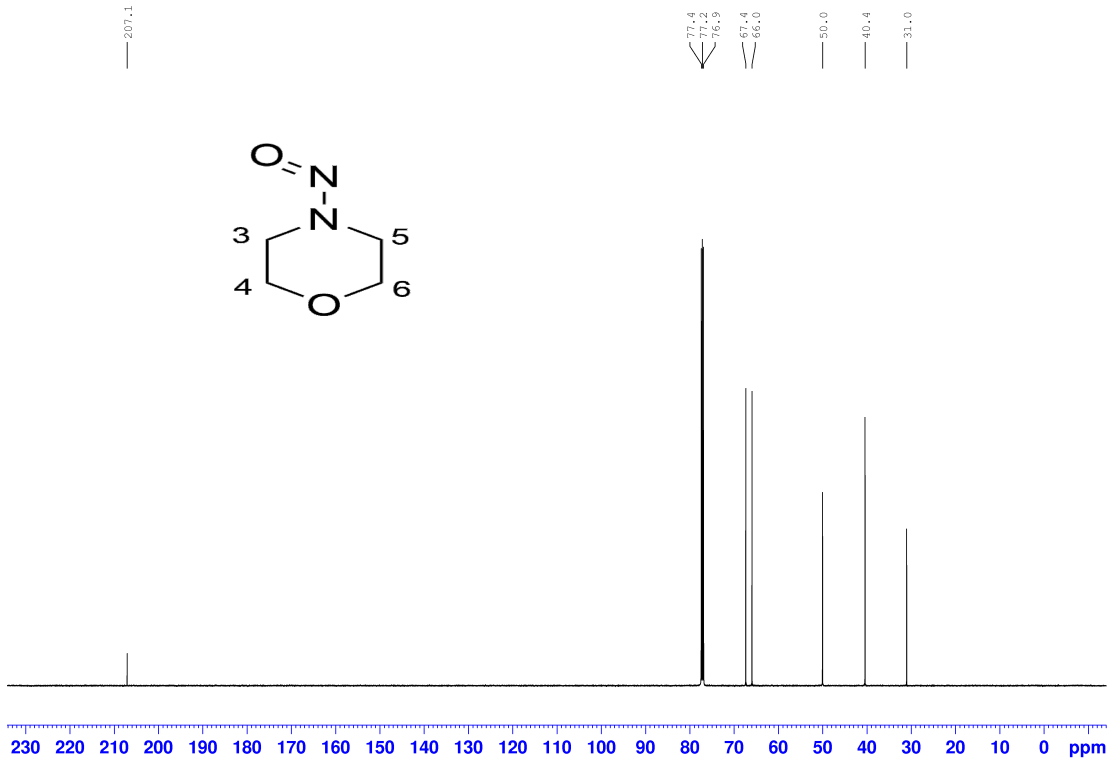

Figure S180: ^{13}C NMR spectrum of *N*-nitrosomorpholine (N5).

^{13}C NMR, δ ($CDCl_3$, 125 MHz): 207.1 (starting material impurity), 67.4 (C6), 66.0 (C4), 50.0 (C5), 40.4 (C3), 31 (starting material impurity)

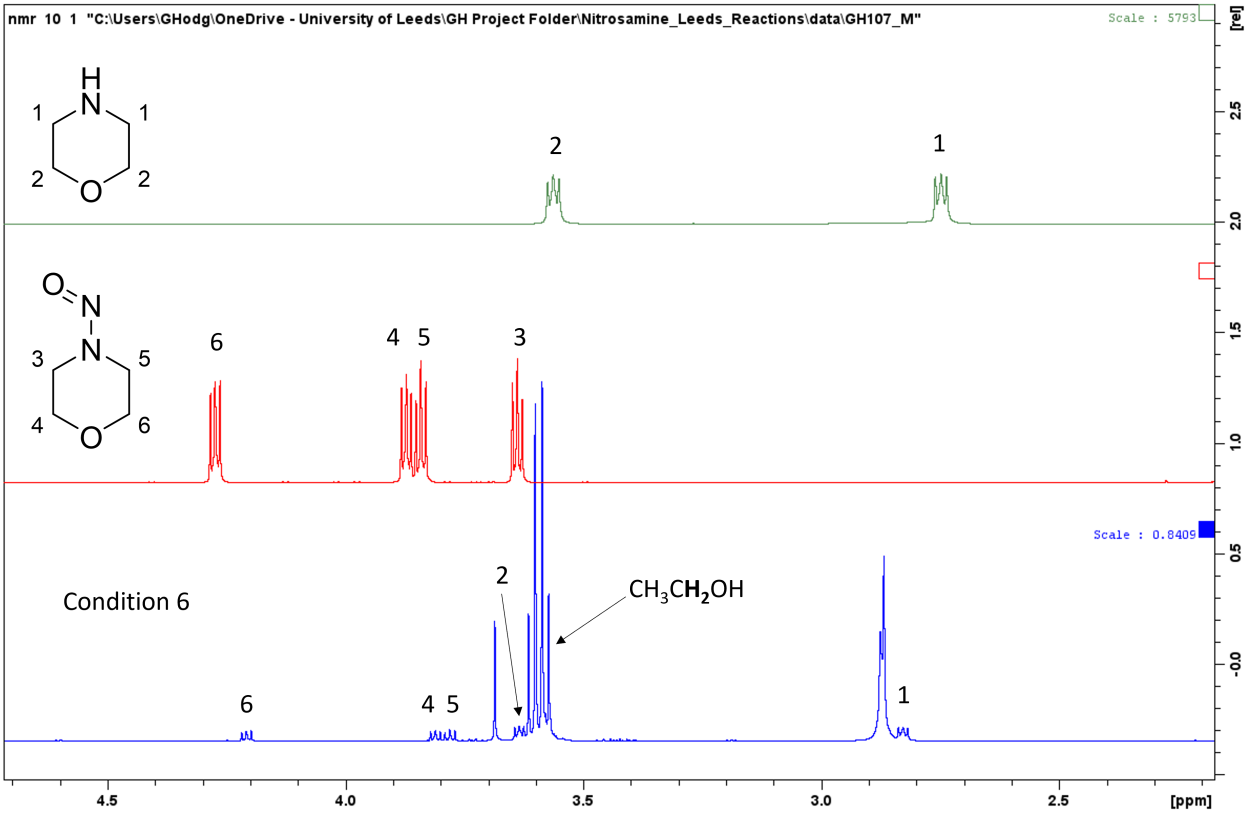

Figure S181: ^{1}H NMR overlay of the starting material (*N*-nitrosomorpholine - red), the product (morpholine - green) and the crude ^{1}H NMR spectrum of N5 in condition 6 - blue.

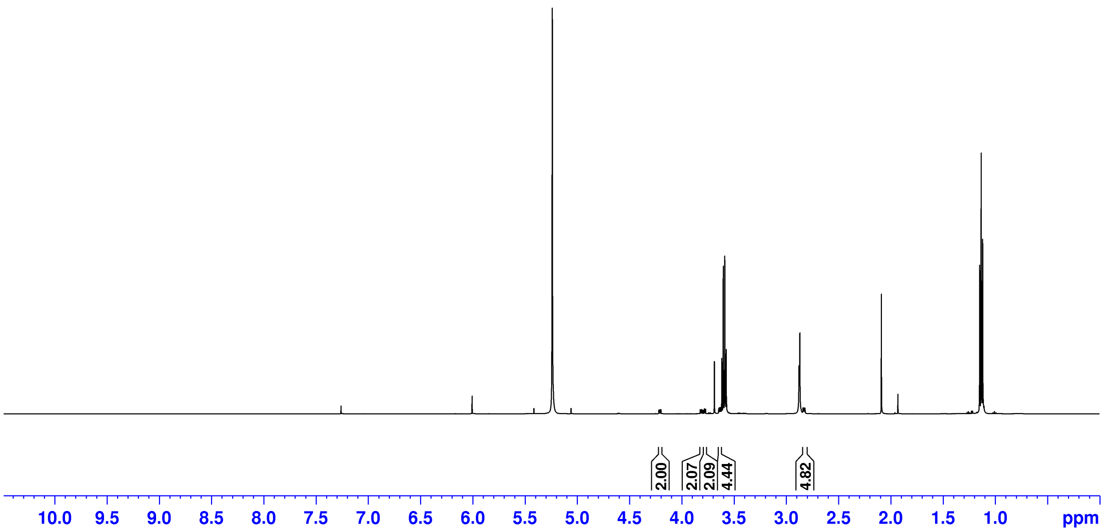

Figure S182: ^{1}H NMR analysis of crude reaction mixture for N5 in condition 6.

^{1}H NMR δ($CDCl_3$, 500 MHz): 4.21 (2H, t, J = 5.1 Hz, H6), 3.81 (2H, t, J = 5.1 Hz, H4), 3.81 (2H, t, J = 5.1 Hz, H5), 3.63 (4H, t, J = 4.7 Hz, H2), 2.28 (4H, t, J = 4.7 Hz, H1)

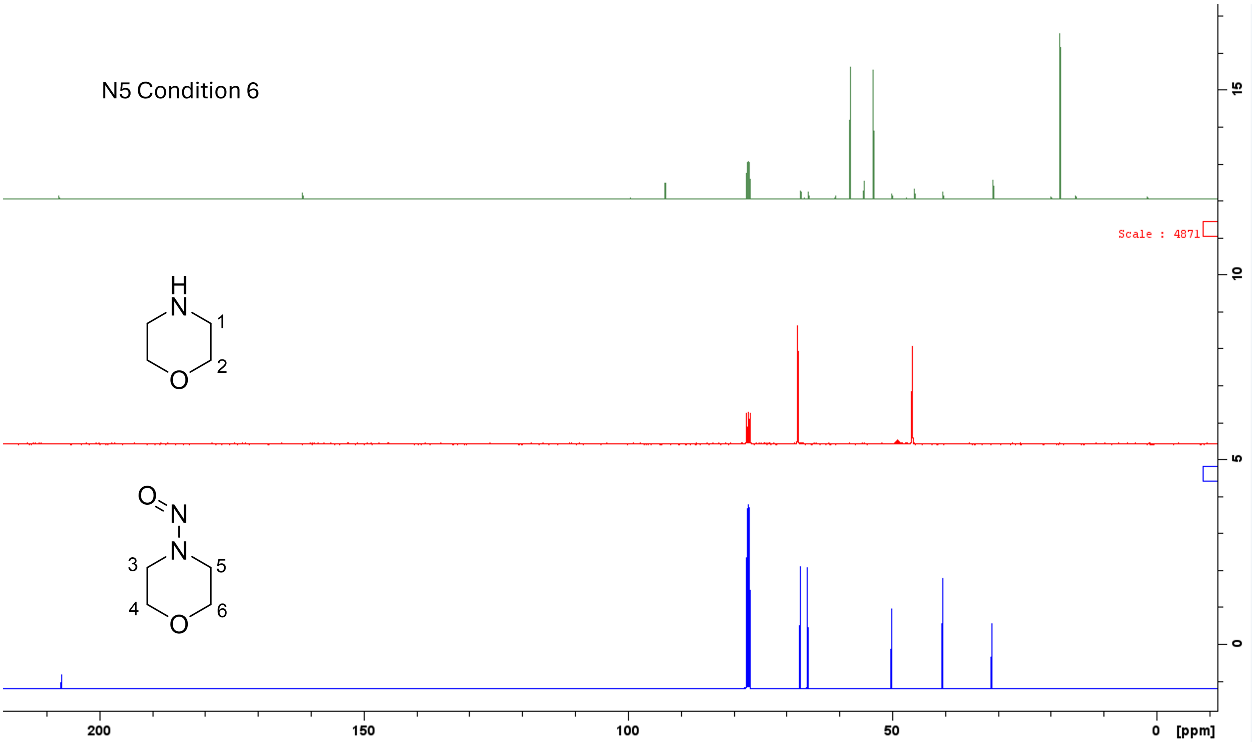

Figure S183: ^{13}C NMR overlay of the starting material (*N*-nitrosomorpholine - blue), the product (morpholine - red) and the crude ^{13}C NMR spectrum of N5 in condition 6 - green.

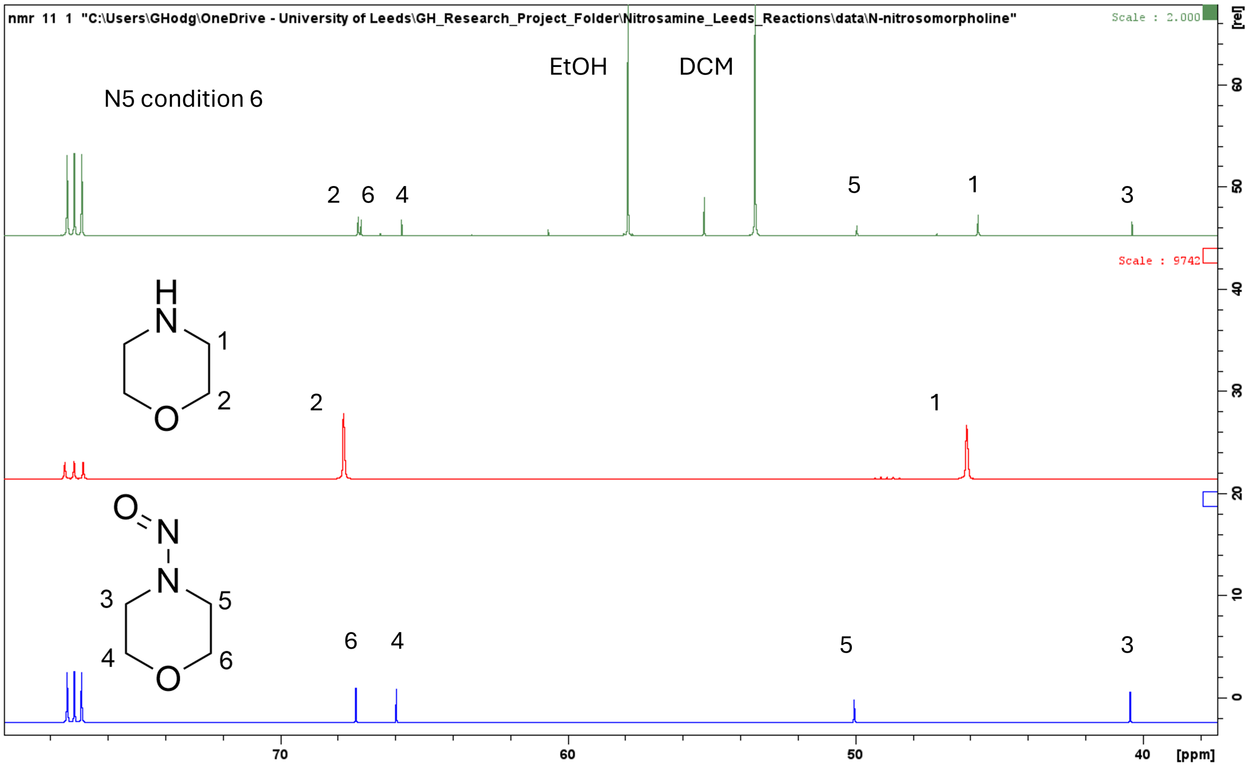

Figure S184: Zoomed ^{13}C NMR overlay of the starting material (*N*-nitrosomorpholine - blue), the product (morpholine - red) and the crude ^{13}C NMR spectrum of N5 in condition 6 - green.

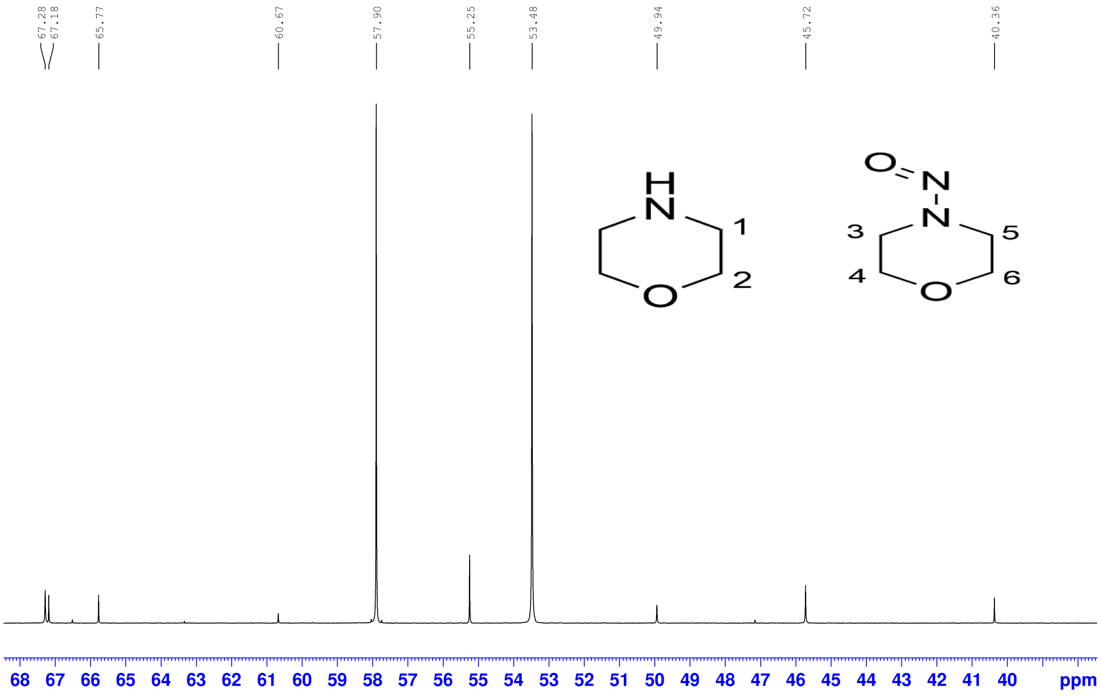

Figure S185: Zoomed ^{13}C NMR spectrum of the crude reaction mixture for N5 in condition 6.

^{13}C NMR, δ ($CDCl_3$, 125 MHz): 67.3 (C2), 67.2 (C6), 65.8 (C4), 60.7, 57.9 (EtOH), 55.3, 53.5 (DCM), 49.9 (C5), 45.7 (C1), 40.4 (C3)

References

(1) Rahman, S. A.; Torrance, G.; Baldacci, L.; Martínez Cuesta, S.; Fenninger, F.; Gopal, N.; Choudhary, S.; May, J. W.; Holliday, G. L.; Steinbeck, C.; Thornton, J. M. Reaction Decoder Tool (RDT): extracting features from chemical reactions. *Bioinformatics* **2016**, *32*, 2065–2066.

(2) Delannée, V.; Nicklaus, M. C. ReactionCode: format for reaction searching, analysis, classification, transform, and encoding/decoding. *Journal of Cheminformatics* **2020**, *12*, 1–13.

(3) Borths, C. J.; Burns, M.; Curran, T.; Ide, N. D. Nitrosamine reactivity: a survey of reactions and purge processes. *Organic Process Research & Development* **2021**, *25*, 1788–1801.

(4) Chow, Y.; Tam, N.; Lee, A. Photochemistry of nitroso compounds in solution. X. The reaction pattern of photo-excited nitrosamides. *Canadian Journal of Chemistry* **1969**, *47*, 2441–2448.

(5) Oscarsson, K.; Classon, B.; Kvarnström, I.; Hallberg, A.; Samuelsson, B. Solid phase assisted synthesis of HIV-1 protease inhibitors. Expedient entry to unsymmetrical substitution of a C 2 symmetric template. *Canadian Journal of Chemistry* **2000**, *78*, 829–837.

(6) MASUI, M.; NOSE, K.; TANAKA, A.; YAMAKAWA, E.; OHMORI, H. Electrochemical Transformation of N-Nitrosodialkylamines into N-Nitramines and β-Ketonitrosamines. *Chemical and Pharmaceutical Bulletin* **1981**, *29*, 3758–3760.

(7) Gowenlock, B.; Young, V.; others Photolysis of some N-nitroso-and N-nitro-anilines in solution. *Journal of the Chemical Society, Perkin Transactions 2* **1997**, 915–920.

(8) Chow, Y. L.; Richard, H.; Snyder, R. W.; Lockhart, R. W. Generation of aminyl and aminium radicals by photolysis of N-nitrodialkylamines in solution. *Canadian Journal of Chemistry* **1979**, *57*, 2936–2943.

(9) TERAO, Y.; MATSUNAGA, K.; SEKIYA, M. Synthesis of α-thio, α-sulfinyl, and α-sulfonyl-substituted nitrosamines. *Chemical and Pharmaceutical Bulletin* **1977**, *25*, 2964–2968.

(10) France, H.; Heilbron, I.; Hey, D. 256. Studies in the terphenyl series. Part I. p-Terphenyl, nitro-, amino-, and halogeno-p-terphenyls. *Journal of the Chemical Society (Resumed)* **1938**, 1364–1375.

(11) Emmons, W. D. Peroxytrifluoroacetic Acid. II. The Oxidation of Anilines to Nitrobenzenes1. *Journal of the American Chemical Society* **1954**, *76*, 3470–3472.

(12) Singh, M.; Reddy, A.; Singh, R.; Micetich, R. Synthesis and in vitro antibacterial activity of 2β and 2α-(substituted methyl) phenoxymethylpenicillins and an oxidation product. *European journal of medicinal chemistry* **1991**, *26*, 791–797.

(13) KROPF, H.; BALL, M.; HJ, F. 1, 2-BIS (ALKYLPEROXY)-ALKANE AUS 3-NITROSO-2-OXO-1, 3-OXAZOLIDINEN UND ALKYL-HYDROPEROXIDEN. **1975**,

(14) Beard, J. C.; Swager, T. M. An organic chemist's guide to N-nitrosamines: their structure, reactivity, and role as contaminants. *The Journal of organic chemistry* **2021**, *86*, 2037–2057.
